# Novel *N*,*N*′-Disubstituted Acylselenoureas as Potential Antioxidant and Cytotoxic Agents

**DOI:** 10.3390/antiox9010055

**Published:** 2020-01-08

**Authors:** Ana Carolina Ruberte, Sandra Ramos-Inza, Carlos Aydillo, Irene Talavera, Ignacio Encío, Daniel Plano, Carmen Sanmartín

**Affiliations:** 1 Departamento de Tecnología y Química Farmacéuticas, Universidad de Navarra, Irunlarrea 1, E-31008 Pamplona, Spain; aruberte@alumni.unav.es (A.C.R.); sramos.2@alumni.unav.es (S.R.-I.); caydillo@unav.es (C.A.); italavera@alumni.unav.es (I.T.); dplano@unav.es (D.P.); 2 Instituto de Investigación Sanitaria de Navarra (IdiSNA), Irunlarrea, 3, 31008 Pamplona, Spain; ignacio.encio@unavarra.es; 3 Departamento de Ciencias de la Salud, Universidad Pública de Navarra, Avda. Barañain s/n, 31008 Pamplona, Spain

**Keywords:** acylselenoureas, antioxidant, cytotoxicity, oxidative stress, radical scavenging, selenium

## Abstract

Selenium compounds are pivotal in medicinal chemistry for their antitumoral and antioxidant properties. Forty seven acylselenoureas have been designed and synthesized following a fragment-based approach. Different scaffolds, including carbo- and hetero-cycles, along with mono- and bi-cyclic moieties, have been linked to the selenium containing skeleton. The dose- and time-dependent radical scavenging activity for all of the compounds were assessed using the in vitro 2,2-diphenyl-1-picrylhydrazyl (DPPH) and 2,2′-azino-bis(3-ethylbenzthiazoline-6-sulfonic acid) (ABTS) assays. Some of them showed a greater radical scavenging capacity at low doses and shorter times than ascorbic acid. Therefore, four compounds were evaluated to test their protective effects against H_2_O_2_-induced oxidative stress. One derivative protected cells against H_2_O_2_-induced damage, increasing cell survival by up to 3.6-fold. Additionally, in vitro cytotoxic activity of all compounds was screened against several cancer cells. Eight compounds were selected to determine their half maximal inhibitory concentration (IC_50_) values towards breast and lung cancer cells, along with their selectivity indexes. The breast cancer cells turned out to be much more sensitive than the lung. Two compounds (**5d** and **10a**) stood out with IC_50_ values between 4.2 μM and 8.0 μM towards MCF-7 and T47D cells, with selectivity indexes greater than 22.9. In addition, compound **10b** exhibited dual antioxidant and cytotoxic activities. Although further evidence is needed, the acylselenourea scaffold could be a feasible frame to develop new dual agents.

## 1. Introduction

Cancer is a major health problem worldwide, being the second leading cause of death globally. According to the World Health Organization, the cancers with the highest incidence are lung and breast, with almost 3 million cases in the world. Of the available treatments for cancer, chemotherapy is still one of the most used nowadays. However, the poor selectivity of chemotherapy agents as well as drug resistance are still an issue; therefore, the development of new anticancer drugs is still needed.

Although the connection among reactive oxygen species (ROS) and cancer was suggested in the 1980s [1], in the last years, the scientific community has driven their attention to cellular oxidative stress for treating cancer [2,3,4]. ROS homeostasis is required for normal cell survival and proper cell signaling, and the excessive production of ROS is considered as an important molecular hallmark for carcinogenesis [5,6,7]. Typically, endogenous radicals usually arise from certain ROS, such as the hydroxyl radical (^−^OH), hydrogen peroxide (H_2_O_2_), the superoxide anion (^•^O_2_^−^), and singlet oxygen (^1^O_2_). In this case, the antioxidants can counteract and neutralize these and other reactive oxygen and nitrogen species (RONS). Among the defensive strategies, endogenous antioxidants enzymatic systems, like glutathione peroxidase, superoxide dismutase, and thioredoxin reductase, or different minerals that have an antioxidative function, such as selenium (Se), can be considered relevant [8]. Notably, the aforementioned enzymatic systems are selenoproteins, and in fact Se exerts its antioxidant actions primarily through its role in selenoproteins [9,10].

Several epidemiological studies have entailed low plasma Se levels with cancer incidence, mainly gastrointestinal and prostate [11,12]. However, contradictory results have been obtained among the follow-up supplementation clinical trials and laboratory studies [13,14,15]. Meanwhile, the Nutrition Prevention of Cancer (NPC) trial, a double-blind, placebo-controlled, and randomized trial, showed evidence of the chemopreventive efficacy of Se [16,17,18], and the subsequent Selenium and Vitamin E Cancer Prevention Trial (SELECT) failed to demonstrate this efficacy [19]. In fact, the use of certain Se species, mainly inorganic forms, may increase cancer risk [20]. Considered together, the clinical trials conducted to date reflect the pivotal role of Se speciation on the chemoprevention, anticancer, or carcinogenic effects of Se, along with the basal plasmatic levels of Se of the patients. Nowadays, it is still unclear whether Se supplementation, particularly if sustained, which results in a higher Se plasma concentration than 125 µg/L—where the concentration of selenoprotein P, and probably of all selenoproteins, reaches a plateau—has any beneficial, or indeed detrimental, effects [21].

The scientific community is therefore taking advantage of Se properties, and research on the development of new chemotherapy agents containing Se is very promising [15]. Among all of the types of species, organic molecules bearing Se have the advantage of being less toxic and more active than inorganic. Compounds as methylseleninic acid, methylselenocysteine, and selenomethionine have been widely studied and demonstrated an anticancer activity in vitro [22]. Other functional groups with Se are continuously being reported as cytotoxic for cancerous cell lines, such as selenocyanates [23], selenides, diselenides [24], and heterocyclic selenazo compounds [25]. 

**Figure 1:**
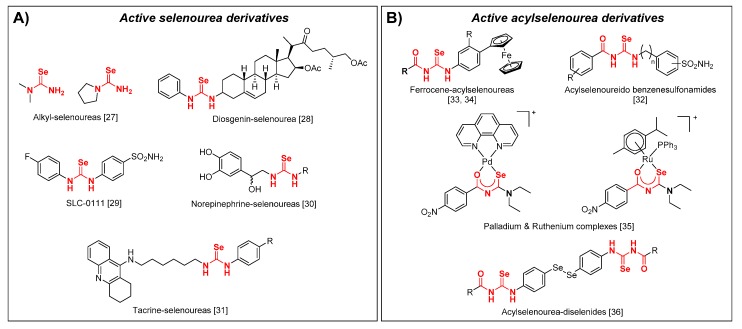
Selenourea (**A**) and acylselenourea (**B**) derivatives with an antioxidant and/or anticancer activity.

During the implementation of our scientific program based in the synthesis and biological evaluation of Se compounds, we have synthetized and observed the antioxidant and anticancer activity of closely related imidoselenocarbamates [26,27,28,29,30,31,32,33,34], seleno-quinazoline, and pyrido[2,3-*d*]pyrimidine derivatives [33,35,36,37]. Some evidence has pointed out that relatively simple selenoureas (Figure 1A) might possess a superoxide radical scavenging activity accompanied by a low toxicity in human cells [38]. More recently, selenourea derivatives have presented anticancer and antioxidant properties, such as diosgenin derived selonourea [39], selenourea based carbonic anhydrase (hCA) inhibitor SLC-0111 [40], selenourea mimic of norepinephrine [41], and selenourea linked to tacrine [42]. Acylselenoureas have also been reported (Figure 1B) as hCA inhibitors [43], and are conjugated with ferrocenyl moieties as antiproliferative agents [44,45]. Palladium and ruthenium complexes of acylselenoureas have also been reported as antiproliferative against leukemia cell lines [46]. Our research group also confirmed that acylselenourea derivatives in combination with diselenide motifs present a potent and selective antitumoral activity [47].

**Figure 2:**
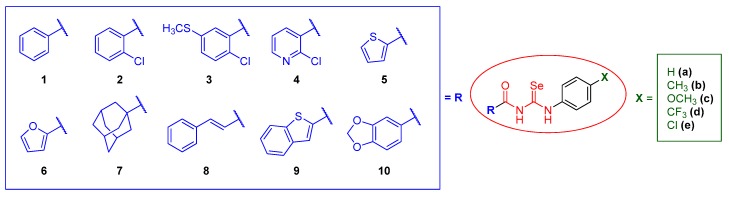
Structural design of the acylselenoureas presented in this work.

Keeping all this previous experience in mind, in this work, we planned the design of a new library of forty seven acylselenoureas with both a potential anticancer and antioxidant activity following a fragment-based approach for drug design [48], varying the two sides of the molecule, searching for a synergistic effect between the fragments and the acylselenourea itself. In the acyl moiety, we envisioned the introduction of different small carbocyclic and heterocyclic systems able to promote H-bonding, and with a reported antitumor and/or antioxidant activity, such as 2-furyl, 2-thienyl [49], 2-chloronicotinyl, benzo[*b*]thienyl [50], benzo[*d*][1,3]dioxolyl [51], and *trans*-cinnamyl [52]. In the opposite location of the molecule, the substitution in the para position of aniline was encompassed with substituents of a different nature (H, CH_3_, OCH_3_, CF_3_, and Cl), in order to modulate the available electron density on the system. The general structure of the designed compounds is depicted in Figure 2, with carbo- and hetero-cyclic moieties (in blue) and variability on aniline substitution (in green). The proposed synthesis of these compounds was similar to the protocols reported previously for analogous compounds [43,53,54,55].

The dose- and time-dependent radical scavenging activity of all of the synthesized compounds were assessed in vitro using the colorimetric assays of 2,2-diphenyl-1-picrylhydrazyl (DPPH) and 2,2′-azino-bis(3-ethylbenzthiazoline-6-sulfonic acid) (ABTS). Additionally, four of them were selected according to their antioxidant capacity for the evaluation of their protective effects on HT-29 cells towards oxidative damage induced by H_2_O_2_ treatment. Likewise, the cytotoxic activity of all of the compounds was screened in vitro at two doses (10 and 50 μM) against a panel of cancer cell lines derived from breast (MCF-7), lung (HTB-54), and colon (HT-29), using the colorimetric assay of 3-(4,5-dimethyl-2-thiazolyl)-2,5-diphenyl-2*H*-tetrazolium bromide (MTT). Eight compounds were selected according to their efficacy, and their IC_50_ values were determined towards breast (MCF-7 and T47D) and lung (HTB-54 and H1299) cancer cells, along with a breast non-malignant derived cell line (184B5), so as to determine their selectivity.

## 2. Materials and Methods

### 2.1. Chemistry

#### 2.1.1. General Information

Reagents, starting materials, and anhydrous solvents were purchased from commercial suppliers, and were used as received. Reaction courses were monitorized by thin-layer chromatography (TLC) on precoated silica gel 60 F_254_ aluminum sheets (Merck, Darmstadt, Germany), and the spots were visualized under UV light. The crude reaction products were purified by silica gel column chromatography using silica gel 60 Å (0.040–0.063 mm, Merck, Darmstadt, Germany), with hexane/ethyl acetate as the elution solvent, by the recrystallization of methanol or methanol:water (50:50), or by washing the crude with hexane, diethyl ether, or ethanol of 70%. ^1^H-, ^13^C-, and ^77^Se-NMR spectra were recorded on a Bruker Avance Neo 400 MHz in CDCl_3_ and DMSO-d_6_ operating at 400, 100, and 76 MHz, respectively. Chemical shifts were reported in δ values (ppm) and *J* values were reported in hertz (Hz).

#### 2.1.2. General Procedure for the Preparation of Acylselenourea Derivatives

It was necessary to synthesize some acyl chloride reagents. These compounds were obtained by treatment with an excess of thionyl chloride (10 mL) at reflux (150 °C) for 1.5 h. The resulting product was isolated by the rotatory evaporation of the thionyl chloride under vacuum, and the excess of thionyl chloride was removed with three fractions of toluene (3 × 50 mL). The resulting acyl chlorides were used without further purification.

The isolated acyl chloride (1 equivalent) was reacted with potassium selenocyanate (1 equivalent) in anhydrous acetone as solvent for 1 h at room temperature. Then, the corresponding aniline derivate (1 equivalent) was added in situ and allowed to stir for 0.5–24 h. The reactions were quenched by cold water addition. The resulting crude was then filtered under a vacuum or extracted with dichloromethane. The crude product was purified by the methods described previously.

### 2.2. Radical Scavenging Activity

The antioxidant activity of the natural and novel synthesized compounds is usually quantified in vitro by measuring either their efficacy in quenching a known concentration of preformed radicals, DPPH, and ABTS radical cations [56], or the enzymatic activity of superoxide dismutase (SOD), glutathione peroxidase (GPx), and catalase (CAT) [57]. Herein, the antioxidant properties of the novel acylselenoureas were determined by DPPH^•^ and ABTS^+^^•^ colorimetric assays. These antioxidant assays evaluate the capacity of the compounds for scavenging radicals in vitro. The measurements were recorded on a BioTeck PowerWave XS spectrophotometer (BioTeck Instruments, Winooski, VT, USA) and the data were collected using KCJunior v.1.41. software (BioTeck Instruments, Winooski, VT, USA). Compounds with a potent activity were selected and their protective effect against cell death induced by ROS was determined.

#### 2.2.1. DPPH Radical Scavenging Assay

DPPH assay was performed as described by Svinyarov [58]. Briefly, each compound was initially dissolved in absolute methanol at a concentration of 1 mg/mL, and serial dilutions were prepared. Ascorbic acid (Asc), a well-known and potent antioxidant, was used as a positive control. A methanolic solution (100 µM) of DPPH^•^ was prepared daily and protected from the dark. The absorbance measurements of this solution were needed in order to check the stability of the radical through the time of analysis. Then, 100 µL of DPPH in methanol were added to 100 µL of the compounds’ solutions, and the decolorization of the purple radical to the yellow reduced form was followed at 517 nm. Determinations were recorded at several time intervals. All of the measurements were carried out in triplicate. The results were expressed as the percentage of the radical scavenged, calculated using the following formula:

(1) $$\%\;\mathrm{DPPH}\;\mathrm{radical}\;\mathrm{scavenging}\;=\;\frac{A_{\mathrm{control}}-A_{\mathrm{sample}}}{A_{\mathrm{control}}}\;\times \;100$$

where A_control_ refers to the absorbance of the negative control and A_sample_ refers to the absorbance of the tested compounds. The results are expressed as percentage of DPPH radical scavenging ± standard deviation (SD).

#### 2.2.2. ABTS Radical Scavenging Assay

ABTS radical scavenging was assayed with a colorimetric test as well [59]. Briefly, ABTS was dissolved in deionized water at 1 mg/mL and oxidized to ABTS^+•^ with potassium persulfate (2.45 mM final concentration). This reaction mixture was kept overnight at room temperature in the dark. After radical generation, ABTS^+•^ was diluted with 50% ethanol until an absorbance of 0.70 ± 0.02 at 734 nm for measurements. The tested compounds were solved in absolute ethanol at a concentration of 1 mg/mL, and 20 µL of this solution were added to 180 µL of the diluted ABTS^+•^. After 6 min of incubation, the absorbance at 734 nm was recorded using 50% ethanol as the blank. Trolox and Asc were used as the positive controls. All of the determinations were carried out in triplicate. The same time intervals as in the DPPH assay were also measured. The ability to scavenge ABTS^+•^ was calculated using the following formula:

(2) $$\%\;\mathrm{ABTS}\;\mathrm{radical}\;\mathrm{scavenging}=\frac{A_{\mathrm{control}}-A_{\mathrm{sample}}}{A_{\mathrm{control}}}\;\times \;100$$

where A_control_ refers to the absorbance of the negative control and A_sample_ refers to the absorbance of the tested compounds. The results are expressed as percentage of ABTS radical scavenging ± SD.

### 2.3. Biological Evaluation

#### 2.3.1. Cell Culture Conditions

The cell lines were obtained from the American Type Culture Collection (ATCC, Manassas, VA, USA). Five tumor cell lines, including breast (MCF-7 and T47D), lung (H1299 and HTB-54), and colorectal (HT-29), were grown in an Roswell Park Memorial Institute (RPMI) 1640 medium (Gibco, Thermo Fisher, Waltham, MA, USA) supplemented with 10% fetal bovine serum (FBS; Gibco, Thermo Fisher, Waltham, MA, USA) and 1% antibiotics (10.00 units/mL penicillin and 10.00 µg/mL streptomycin; Gibco, Thermo Fisher, Waltham, MA, USA). Non-tumorigenic cells derived from breast tissue (184B5) were grown in DMEM/F12 (1:1) (1X) + GlutaMAX medium (Gibco, Thermo Fisher, Waltham, MA, USA) supplemented with 5% FBS; 1% antibiotics; and a supplement cocktail containing 1 mL of hydrocortisone (100 nm; Aldrich, Darmstadt, Germany), 1xITS (Lonza, Basel, Switzerland), 10 mL of sodium pyruvate (2 mM; Lonza, Basel, Switzerland), 10 µL of EGF (20 ng/mL; Aldrich, Darmstadt, Germany), and 150 µL of *trans*-retinoic acid (0.3 nM; Aldrich, Darmstadt, Germany). The culture medium was replaced every three days.

#### 2.3.2. Cell Viability Assay

The cell viability assay of each compound was carried out using the MTT assay [32]. Each compound was dissolved in dimethyl sulfoxide (DMSO) at a concentration of 0.01 M. Serial dilutions were prepared with a non-supplemented culture medium. The effect of each compound on the cell viability was determined at two different concentrations (10 and 50 µM) in MCF-7, HTB-54, and HT-29 cells. In addition, the selected compounds were tested at six different concentrations between 0.1–100 µM in MCF-7, T47D, H1299, HTB-54, and 184B5 cells.

Briefly, 1 × 10^4^ tumor cells/well or 2 × 10^4^ non-tumorigenic cells/well were grown in 96-well plates for 12 h. Then, these cells were incubated with either DMSO (control) or a different concentration of each of the tested compounds for 48 h. Three hours before the termination point, 20 µL of MTT were added to measure the cellular viability. The resultant formazan crystals were dissolved in 50 µL of DMSO, and the absorbance was measured at 570 and 630 nm. The results are expressed as IC_50_, the half maximal inhibitory concentration. This value was calculated using OriginPro 8.5.1. software (OriginLab, Northampton, MA, USA) by nonlinear curve fitting. Furthermore, the selectivity index (SI) was calculated as the ratio of the IC_50_ values determined for the non-malignant and the tumor cells (IC_50_ (184B5)/IC_50_ (MCF-7). The data were obtained from at least three independent experiments performed in quadruplicates.

#### 2.3.3. Protective Effects against H_2_O_2_-Induced Oxidative Stress in HT-29 Cells

An H_2_O_2_-induced oxidative damage model on HT-29 cells was established, and the cell viability was evaluated by MTT assay, as a measurement of the antioxidant activity of the compounds. Asc was used as a positive control. Briefly, 1 × 10^4^ HT-29 cells per well were grown in 96-well plates. After 12 h of incubation, DMSO; **4b**, **8c**, **9c**, and **10b** compounds; or Asc were added to each well at concentrations of 0.03 and 0.003 mg/mL, and incubated for 1 h. Subsequently, H_2_O_2_ (200, 250, and 300 µM) was added and the cells were incubated for 12 h to induce cell damage. An MTT assay was performed as mentioned previously. For each treatment, the mean cell viability was calculated from three independent experiments. The DMSO-treated controls were assigned with a cell viability value of 100% (V_control_) [60,61]. The results are expressed as the increase fold of cell survival, as follows:

(3) $$\mathrm{Increase}\;\mathrm{fold}\;\mathrm{of}\;\mathrm{cell}\;\mathrm{survival}\;=\;\frac{\left(V_{\mathrm{sample}}-V_{\mathrm{sample}\;+\;H2O2}\right)}{V_{\mathrm{Control}}-V_{H2O2}}$$

where V_sample_ refers to the viability of the cells only treated with the corresponding compound, V_H2O2_ refers to the viability of the HT-29 cells exposed to H_2_O_2_, and V_sample + H2O2_ refers to the cell survival of each treatment with a compound and H_2_O_2_. The data are expressed as increase fold of cell survival ± standard error of the mean (SEM) in at least three independent experiments performed in quadruplicates.

## 3. Results and Discussion

### 3.1. Chemistry

**Scheme 1:**
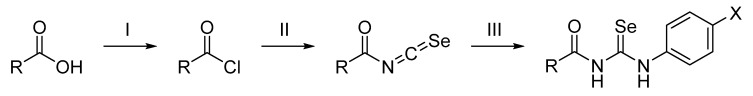
Synthesis of the acylselenourea derivatives. Reagent and conditions: (**I**) SOCl_2_, 150 °C, 1.5 h; (**II**) KSeCN, acetone (dry), room temperature, 1 h; (**III**) corresponding aniline, room temperature, 0.5–24 h.

The synthetic route of the final compounds is outlined in Scheme 1 and was carried out in two steps. First, the acyl isoselenocyanates were prepared by a reaction between the commercially available acyl chlorides, or prepared from corresponding carboxylic acids, and potassium selenocyanate. Second, the resulting acyl isoselenocyanates were not isolated from the mixture and were converted into the corresponding acylselenoureas by adding different anilines in dried acetone as a solvent. The reaction was carried out at room temperature with stirring for 0.5–24 h. The targeted compounds were obtained with yields ranging from 10% to 95%, and all of the structures of the newly synthesized compounds were elucidated on the basis of their spectroscopic data.

The characterization of all forty-seven acylselenoureas is included in the Supplementary material, along with the ^1^H-, ^13^C-, and ^77^Se-NMR spectra recorded for each compound (Figures S28–S168). An inspection of the ^1^H-NMR spectra revealed two characteristic peaks common to all structures. The signal most shifted downfield appears in the range 13.20–9.75 ppm, and corresponds to the proton linked to both C=O and C=Se. The other peak appears in the interval of 12.37–9.10 ppm, corresponding to the proton linked to the aniline. Interestingly, a chemical shift increase was observed because of the presence of trifluoromethyl group, which is the most electron-withdrawing substituent, causing a deshielding over these protons. Likewise, this behavior was also observed in the ^77^Se-NMR spectra because of the effect of the electron density nature of the different substituents in the para position of aniline. For each nucleus, these substituents increased the chemical shift of Se as their electron-withdrawing ability did (CF_3_ > Cl > H > CH_3_ > OCH_3_). Overall, the Se signal for the acylselenoureas appears in a range of 576–366 ppm.

### 3.2. Antioxidant Activity

As the chemopreventive activity of Se has been related with its ability to interact with the redox status of the cell [62], we decided to determine the radical scavenging activity of the synthesized compounds. With this purpose, the ability of the compounds to scavenge both DPPH^•^ and ABTS^+•^ radicals was measured. Compounds displaying interesting radical scavenging activities were also tested in a cancer cell line (HT-29) so as to determine their ability to prevent cell death induced by ROS.

#### 3.2.1. DPPH Radical Scavenging Assay

The DPPH assay was used as a first experimental approach to determine the radical scavenging of the new acylselenourea derivatives. Measurements were performed at four different concentrations ranging from 0.0003 to 0.03 mg/mL, and collected at different time lengths (Figures S1–S17). The results obtained at two concentrations (Figure 3A,B) and two time-points (Figure 3A–C) are presented as the percentage of inhibition for the DPPH radical scavenging activity.

**Figure 3:**
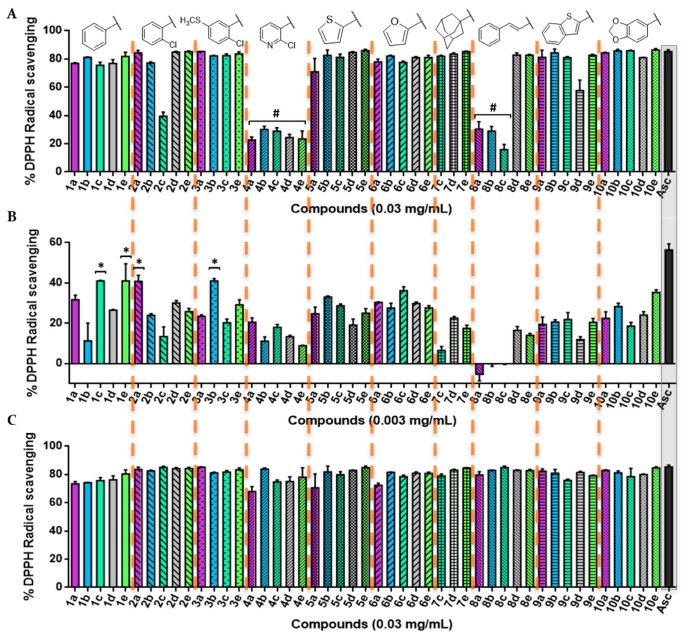
2,2-diphenyl-1-picrylhydrazyl (DPPH) radical scavenging activity of the novel compounds synthetized in this work. (**A**) Percentage of DPPH^•^ scavenging after 0 min at the highest concentration tested (0.03 mg/mL); (**B**) percentage of DPPH^•^ scavenging after 0 min at 0.003 mg/mL; (**C**) percentage of DPPH^•^ scavenging at 0.03 mg/mL after 30 min for all of the 47 compounds. Significance: * *p* < 0.05 compared with ascorbic acid (Asc). The results obtained for lower concentrations and longer times are included in the Supplementary Material.

The scavenging activity was dose-dependent, as illustrated in Figure 3. Overall, most of the compounds showed a potent radical scavenging activity at 0.03 mg/mL, even similar to the positive control with no significant differences (*p* < 0.05) through a very fast kinetic manner, reaching the highest DPPH activity inhibition after treatment (0 min; Figure 3A). On the contrary, most of the 2-chloropyridyl (**4a**–**e**) and *trans*-cinnamyl (**8a**–**c**) derivatives demonstrated a lower kinetic profile, with 30 min being necessary to achieve maximum inhibition (Figure 3C). Likewise, all of the synthesized compounds maintained their radical scavenging effect throughout the time, up to the highest time-point tested (120 min; Figure S3).

At a lower concentration (0.003 mg/mL), compounds **1c**, **1e**, **2a**, and **3b** were able to scavenge the DPPH^•^ with values around 40%, although none of them showed an activity significantly comparable (*p* < 0.05) to that of Asc. Overall, the compounds with a *trans*-cinnamyl moiety (series 8) are less antioxidant (with an average radical inhibition of 5.01%) than the rest of the derivatives, whereas compounds from series **1** (phenyl) and **6** (furyl) are globally the most active ones (average DPPH inhibition of 30.32%).

Interestingly, compound 1c presented a greater inhibition compared with Asc (*p* < 0.05) at a lower dose (0.0006 mg/mL) for all of the time-points tested under our experimental conditions (Figures S8–S12), while other compounds, such as **4b**, **10b**, and **10e**, showed a similar activity statistically (*p* < 0.05) to the positive control. In addition, even at the lowest concentration tested (0.0003 mg/mL, Figure S13), several acylselenoureas showed an analogous behavior as the Asc (*p* < 0.05), which were not time-dependent (Figures S13–S17).

Additionally, the radical scavenging profile showed by these acylselenoureas substantially differed from the one observed for sodium selenite (Na_2_SeO_3_), a well-known antioxidant Se compound [63,64], which only showed DPPH radical scavenging activity at pH 3.0 [65] (conditions not tested in our experimental setup).

#### 3.2.2. ABTS Radical Scavenging Assay

**Figure 4:**
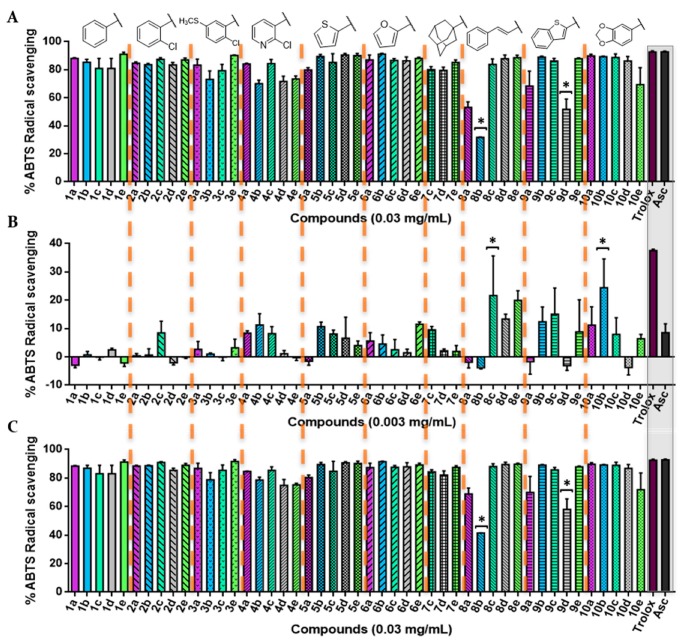
2,2′-azino-bis(3-ethylbenzthiazoline-6-sulfonic acid) (ABTS) radical scavenging activity of the novel acylselenourea compounds. (**A**) Percentage of ABTS^+•^ scavenging after 0 min at the highest concentration tested (0.03 mg/mL); (**B**) percentage of ABTS^+•^ scavenging after 0 min at 0.003 mg/mL; (**C**) percentage of ABTS^+•^ scavenging at 0.03 mg/mL after 6 min of incubation for all the 47 compounds. Significance: * *p* < 0.05 compare with Asc and Trolox. The results obtained for longer times are included in the Supplementary Material.

To further confirm the radical scavenging activity of the compounds, we determined their capacity to reduce ABTS^+•^. Given that the compounds exerted their scavenging activity in the DPPH assay mainly at the two highest concentrations tested (0.03 and 0.003 mg/mL), we decided to perform this assay at those same concentrations. The measurements were recorded after 6 min of incubation with ABTS^+•^**,** and at the same time intervals measured in the previous assay (Figures S18–S26). Trolox and Asc were used as the positive controls for this assay. The results obtained are expressed as the percentage of scavenged ABTS^+^**^•^,** and are shown in Figure 4A–C.

The evidence found confirmed the radical scavenging capacity of the acylselenourea derivatives. All of the compounds displayed an activity at the highest concentration, comparable to both of the positive controls (*p* < 0.05), whereas only two compounds (**8b** and **9d**) showed values below 60% at 0.03 mg/mL after the incubation time (Figure 4A,C).

As shown in Figure 4A–C, the new compounds exhibited concentration-dependent, but not time-dependent radical scavenging activity in vitro. At 0.003 mg/mL, several compounds decreased their scavenging activity considerably, although most of them were still more potent than Asc. In addition, acylselenoureas with methyl (**b**) or methoxy (**c**) substituent groups in the aromatic ring had a tendency of being slightly more active than the rest of their corresponding series at this concentration (Figure 4B and Figures S23 and S24). In this context, two of them (**8c** and **10b**) showed a potent radical scavenging activity similar to Trolox, with no significant differences (*p* < 0.05; Figure 4B).

Moreover, the average of radical scavenging activities of all of the acylselenoureas, containing cinnamyl (**8**), benzothienyl (**9**), or benzodioxyl (**10**) groups showed more of an antioxidant capacity than the rest of acylselenoureas at low concentrations.

Finally, taking into account both of the radical scavenging assays, we selected those acylselenourea derivatives that showed not only a potent activity at high concentration, but also displayed a radical scavenging capacity at low concentrations. Therefore, compounds **4b**, **8c**, **9c**, and **10b** were selected to further test their ability to prevent ex vivo cell death caused by an oxidizing agent (H_2_O_2_).

#### 3.2.3. Protective Effects against H_2_O_2_-Induced Cell Damage in HT-29 Cells

**Figure 5:**
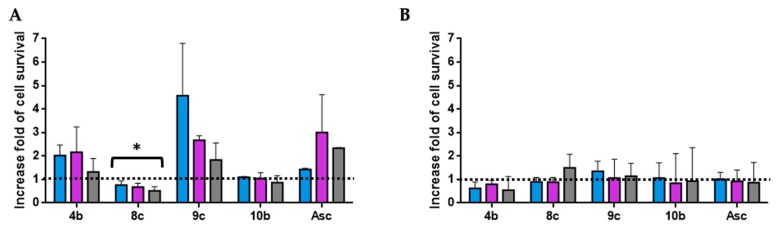
Antioxidant activity of hit compounds at 0.03 mg/mL (**A**) and 0.003 mg/mL (**B**) was evaluated using the H_2_O_2_-induced HT-29 cells oxidative damage model at 300 (blue), 250 (violet), and 200 µM (grey). The results are expressed as an increase fold of cell survival. Significance: * *p* < 0.05 compared with Asc.

The antioxidant activity of the selected compounds (**4b**, **8c**, **9c**, and **10b**) and Asc (positive control) was evaluated in H_2_O_2_-damaged HT-29 cells at the two highest concentrations used previously in the radical scavenging assays (0.03 and 0.003 mg/mL), using the MTT method (Figure 5). The cell viability with H_2_O_2_ oxidation was evaluated at three different doses, namely: 300, 250, and 200 µM. In this context, the cell death of the HT-29 cells increased by 29.3%, 31.5%, and 21.5% after 12 h of exposure to 300, 250, and 200 µM of H_2_O_2_, respectively. Whilst these hydrogen peroxide concentrations might not be relevant for mimicking the intracellular ROS levels on in vivo models, they were chosen to induce ample oxidative stress for inducing reasonable levels of cell death.

Three compounds (**4b**, **9c**, and **10b**) show a similar antioxidant capacity to the positive control at a high dose (0.03 mg/mL). However, the protective effect against H_2_O_2_-induced oxidative stress in the cell for each compound, as well as Asc, disappeared at a low dose (0.003 mg/mL). Moreover, compound **9c**, which contains the benzothienyl group, was the most antioxidant derivative at 0.03 mg/mL. This derivative protected the HT-29 cells against H_2_O_2_ oxidation (300, 250, and 200 µM), with up to 3.6-, 1.7-, and 0.8-fold of cell survival, respectively (Figure 5).

### 3.3. Biological Evaluation

As it has been previously reported, ROS homeostasis is necessary for the normal function of the cell, whereas its excessive production is related with carcinogenesis. In this context, our results show that these acylselenoureas could act as chemopreventive agents, and therefore we decided to evaluate their cytotoxicity in several cancer cell lines as a first approach to test their potential activity as chemotherapeutic agents.

#### Cytotoxic Activity

All of the compounds were screened for their cytotoxic activity against a panel of three human tumor cell lines, namely: lung carcinoma (HTB-54), colon carcinoma (HT-29), and breast adenocarcinoma (MCF-7). The in vitro inhibition of the cell viability was analyzed by a colorimetric microassay based on the reduction of MTT [66]. The three cell lines were treated with each compound for 48 h at two concentrations (50 and 10 μM). The data are expressed as the percentage of cell growth ± SEM in at least three independent experiments performed in quadruplicates (Table S1).

Most of the compounds were active under our experimental conditions, with MCF-7 and HTB-54 cells being the most sensitive cells. This profile was quite unexpected considering that, as previously mentioned, most of the Se compounds demonstrated an anticancer activity on gastrointestinal and prostate cancers. Nonetheless, the newly synthesized acylselenoureas demonstrated the greatest activity towards breast cancer cells, making these analogs worth further evaluating against this specific cancer. As shown in Table S1, compounds **5d**, **6a**, **9e**, **10a**, **10b**, **10c**, and **10e** were the most active, with a reduction of cell growth by at least 45% after treatment with 10 μM, and by more than 85% after treatments at 50 μM after 48 h, in at least one of the cell lines. These results allowed us to determine some preliminary structure–activity relationships among the different small carbocyclic and heterocyclic systems substituted in the acyl region. Thus, acylselenourea derivatives with benzodioxyl, a well-known chemotherapeutic heterocycle [55,67,68,69] (**10a**–**c** and **10e**), seemed to be more active than all of the other synthesized acylselenourea derivatives. Nevertheless, among the different substituents (H, Me, OCH_3_, CF_3_, and Cl) on the aniline ring, these groups seemed not to be important for the inhibition of cell viability.

Compounds **5d**, **6a**, and **9e**, and all of the derivatives with benzodioxyl heterocycle (**10a**–**d**) were further tested at six different concentrations (0.1, 1, 5, 10, 25, 50, and 100 µM), after 48 h of treatment, in order to establish their dose–response curve. A panel of two different lung human cancer cells (HTB-54 and H1299) and two different breast carcinoma cells (MCF-7 and T47D) were used. The potential selectivity of these compounds was further examined in one cell line derived from normal breast tissue (184B5). The results are expressed as IC_50_ and the selectivity index (SI), calculated as the ratio of the IC_50_ values determined for the non-malignant and the tumor cells, respectively (IC_50_ (184B5)/IC_50_ (MCF-7)). The obtained results are shown in Table 1.

As shown in Table 1, the selected cancer cell lines present different sensitivity profiles to the action of these derivatives, with the breast cancer cells being much more sensitive than lung ones. In this context, breast cancer cells were more sensitive to **5d**, an acylselenourea with the thienyl group, with 6.1 and 4.2 μM IC_50_ values on T47D and MCF-7, respectively. This derivative also showed a high selectivity index (>23.7) towards malignant breast cells (Table 1). In addition, compounds **10b** and **10c** were the most active on both lung cancer cells (HTB-54 and H1299) with IC_50_ values ranging from 1.5 to 7.3 μM, and also exhibited selective cytotoxicity towards breast cancer cells. Both derivatives have the same benzodioxyl heterocycle, but different substituents on the aniline ring (CH_3_ and OCH_3_, respectively). Interestingly, benzodioxyl derivative **10b** also displayed a potent radical scavenging activity in both the DPPH and ABTS assays, and showed a similar protective effect against oxidative stress on cells to Asc. Thus, we have discovered a compound with dual activity.

## 4. Conclusions

To sum up, results demonstrated that acylselenourea derivatives are excellent radical scavenging agents, which present a fast kinetic behavior and antioxidant effects similar to the positive controls, Asc and Trolox. Furthermore, **9c** protected cells against H_2_O_2_ oxidation (300 µM), achieving an increase on cell survival up to 3.6-fold. In addition, eight acylselenoureas, five of them containing the benzodioxyl group, exhibited a potent and selective cytotoxic activity, with IC_50_ values below 17.4 µM on breast cancer cells, together with selectivity indexes greater than 6.2. Thus, these results evoke interest in obtaining new acylselenourea derivatives with the benzodioxyl group for the development of therapeutic products with dual functions, such as **10b**, involving antioxidant and selective cytotoxic activity. This dual activity observed for this compound might suggest the further discovery of drugs effective as both chemopreventive and chemotherapeutic. Notwithstanding, further studies in order to characterize the signaling pathways by which these compounds exert their anticancer effect are vital. Likewise, the elucidation of the in vitro interaction with the radical species used is also needed. Finally, the possible interaction of these acylselenourea with the different endogenous intracellular antioxidant networks, including glutathione peroxidases, could encourage the development of these Se compounds.

## Figures and Tables

**Table 1:** Cytotoxic activity, expressed as average IC_50_ values (µM) and selectivity indexes (SI) calculated of derivatives **5d**, **6a**, **9e**, **10a**, **10b**, **10c**, **10d**, and **10e**.

Compound	IC_50_ (µM) ^1^					
	Cell Lines					
	HTB-54	H1299	T47D	MCF-7	184B5	SI ^2^
**5d**	14.6 ± 6.3	7.5 ± 0.2	6.1 ± 0.4	4.2 ± 0.2	>100	>23.7
**6a**	26.1 ± 2.3	30.2 ± 7.2	7.8 ± 1.0	3.5 ± 0.6	52.2 ± 3.8	14.7
**9e**	>100	8.7 ± 1.4	7.1 ± 0.6	9.8 ± 0.9	>100	>10.2
**10a**	>100	11.2 ± 0.8	8.0 ± 1.0	4.4 ± 1.0	>100	>22.9
**10b**	1.5 ± 1.8	5.9 ± 1.4	11.9 ± 1.0	7.3 ± 0.3	>100	>13.6
**10c**	5.2 ± 4.0	7.3 ± 0.2	12.9 ± 1.5	8.5 ± 0.6	53.8 ± 10.5	6.3
**10d**	23.4 ± 3.2	5.7 ± 0.1	11.8 ± 1.9	7.9 ± 0.7	>100	>12.7
**10e**	2.9 ± 3.2	48.6 ± 0.9	17.4 ± 3.1	11.7 ± 1.5	73.1 ± 5.2	6.2

^1^ IC_50_: half maximal inhibitory concentration; ^2^ SI: Selectivity index was calculated as the ratio of the IC_50_ values determined for the non-malignant and the tumor cells (IC_50_ (184B5)/IC_50_ (MCF-7)).

## Supplementary Materials

The following are available online at https://www.mdpi.com/2076-3921/9/1/55/s1, Table S1: Cytotoxic activity of all acylselenourea derivatives synthesized. Figures S1–S17: Supplementary data for DPPH assays. Figures S18–S26: Supplementary data for ABTS assays. Figure S27: General structure used to assign the chemical shifts in NMR spectroscopy. Figures S28–S168: NMR spectra (^1^H, ^13^C and ^77^Se) of final products.
