# Supplementary material for: Novel N,N′-Disubstituted Acylselenoureas as Potential Antioxidant and Cytotoxic Agents
Source: Antioxidants (Basel). 2020 Jan 8;9(1):55. doi: 10.3390/antiox9010055 (PMC7023466; doi:10.3390/antiox9010055)
Supplement: Supplementary file 1 [file antioxidants-09-00055-s001.pdf]

Article

Novel *N,N'*-Disubstituted Acylselenoureas as Potential Antioxidant and Cytotoxic Agents

Ana Carolina Ruberte [1,2,†], Sandra Ramos-Inza [1,2,†], Carlos Aydillo [1,2], Irene Talavera [1,2], Ignacio Encío [2,3], Daniel Plano [1,2] and Carmen Sanmartín [1,2,*]

1 Departamento de Tecnología y Química Farmacéuticas, Universidad de Navarra, Irunlarrea 1, E-31008 Pamplona, Spain; aruberte@alumni.unav.es (A.C.R.); sramos.2@alumni.unav.es (S.R.-I.); caydillo@unav.es (C.A.); italavera@alumni.unav.es (I.T.); dplano@unav.es (D.P.)

2 Instituto de Investigación Sanitaria de Navarra (IdiSNA), Irunlarrea, 3, 31008 Pamplona, Spain; ignacio.encio@unavarra.es

3 Departamento de Ciencias de la Salud, Universidad Pública de Navarra, Avda. Barañain s/n, 31008, Pamplona, Spain;

* Correspondence: sanmartin@unav.es; Tel.: +34-948425600 ext. 806388

† These authors have contributed equally to the manuscript

Contents:

1. Cytotoxicity screening assay of all derivatives synthesized (**Table S1**)
2. Supplementary data for DPPH (**Figures S1-S17**) and ABTS (**Figures S18-S26**) assays
3. General structure used to assign the chemical shifts in NMR spectroscopy (**Figure S27**). Characterization of the compounds synthetized in this work
4. NMR spectra (^{1}H, ^{13}C and ^{77}Se) of final products (**Figures S28-S168**)

1. CYTOTOXICITY SCREENING ASSAY

Table S1. Cytotoxic activity of all acylselenourea derivatives synthesized. The data are expressed as percentage of cell growth ± SEM in at least 3 independent experiments performed in quadruplicates.

		MCF-7		HT-29		HTB-54	
**Comp.**	**Dose (μM)**	**Growth (%)**	**SEM**	**Growth (%)**	**SEM**	**Growth (%)**	**SEM**
**1a**	10	70.5	7.0	56.9	2.5	67.8	4.7
	50	6.6	1.5	23.4	2.3	57.1	2.8
**1b**	10	64.2	5.8	60.4	3.5	75.8	4.5
	50	12.6	2.8	28.0	2.1	61.1	4.9
**1c**	10	67.8	1.7	74.9	6.6	64.4	6.6
	50	13.9	1.8	31.9	0.2	57.2	1.7
**1d**	10	73.6	7.7	97.3	8.8	84.9	7.3
	50	21.9	1.1	25.1	1.0	38.7	2.7
**1e**	10	74.0	3.9	77.9	4.2	82.3	5.1
	50	13.9	3.9	33.8	2.5	57.2	0.9
**2a**	10	93.6	8.4	79.1	9.4	91.3	6.0
	50	30.5	3.1	37.8	3.6	65.6	3.5
**2b**	10	60.1	14.0	80.2	8.0	87.9	4.1
	50	24.7	1.1	46.1	2.0	66.6	9.7
**2c**	10	62.6	10.1	76.3	8.3	84.9	7.5
	50	26.4	1.6	44.8	6.6	66.7	2.0
**2d**	10	94.2	0.7	87.1	10.9	100.4	0.2
	50	10.4	2.2	51.7	6.4	37.6	2.0
**2e**	10	118.1	9.2	77.8	5.3	96.7	7.0
	50	16.0	9.2	57.9	3.3	18.6	1.8
**3a**	10	84.1	6.2	71.1	5.1	88.8	5.5
	50	23.1	6.4	42.7	1.7	49.9	10.1
**3b**	10	64.2	4.9	64.7	4.0	45.1	13.0
	50	24.5	2.5	42.5	2.0	22.2	3.5
**3c**	10	73.9	6.5	67.9	2.2	79.0	7.6
	50	23.7	1.5	44.4	1.3	27.6	2.8
**3e**	10	105.1	5.6	76.6	1.4	79.4	10.7
	50	32.4	5.2	73.0	1.7	35.8	15.4
**4a**	10	68.8	11.5	99.1	5.5	83.1	6.4
	50	29.3	4.9	34.1	1.1	67.6	6.7
**4b**	10	77.6	5.1	99.2	2.5	87.0	2.2
	50	32.4	5.5	51.7	2.1	64.8	3.9
**4c**	10	86.6	7.3	99.1	7.2	88.9	5.0
	50	33.0	2.9	51.6	6.4	68.5	2.5
**4d**	10	67.1	2.9	103.2	11.8	90.5	0.8
	50	48.9	5.8	62.2	5.2	62.2	5.6
**4e**	10	90.6	6.7	93.4	8.7	92.1	7.8

	50	60.2	5.1	67.0	6.1	63.5	2.3
**5a**	10	70.3	4.6	73.5	2.2	76.8	4.4
	50	27.4	2.6	39.0	2.7	43.7	11.6
**5b**	10	64.2	4.4	82.8	7.0	66.8	2.2
	50	17.5	2.1	36.8	2.3	66.7	4.7
**5c**	10	90.7	4.1	57.5	4.5	85.0	6.0
	50	11.8	2.2	34.6	2.6	60.2	8.5
**5d**	10	46.7	9.9	60.6	4.7	76.2	1.0
	50	23.5	3.4	29.3	2.0	4.3	0.2
**5e**	10	77.6	2.4	78.1	1.3	73.2	4.2
	50	20.8	4.4	37.8	3.6	58.1	2.2
**6a**	10	39.9	1.5	72.6	3.0	72.8	5.6
	50	5.0	0.9	35.5	1.9	0.7	1.3
**6b**	10	64.9	2.4	87.6	1.1	73.0	8.0
	50	5.8	2.0	48.4	1.7	1.6	1.0
**6c**	10	70.0	4.7	74.5	2.9	74.2	4.2
	50	13.1	2.8	35.9	4.3	3.8	1.1
**6d**	10	71.7	3.7	81.5	8.0	59.4	3.7
	50	11.9	0.3	18.7	1.9	4.6	1.9
**6e**	10	62.7	7.0	75.2	2.6	84.1	2.0
	50	11.2	1.6	31.1	3.1	4.0	1.5
**7c**	10	91.8	5.3	59.6	4.2	98.4	5.1
	50	24.3	8.7	34.0	3.7	48.3	9.5
**7d**	10	87.9	8.2	49.5	2.8	92.9	5.3
	50	25.3	9.2	29.6	5.8	79.8	6.6
**7e**	10	83.9	6.4	53.5	2.8	110.7	7.7
	50	39.0	8.2	45.9	2.1	36.4	5.4
**8a**	10	75.8	7.2	98.6	12.4	65.2	7.3
	50	15.3	2.1	34.2	2.5	49.7	4.9
**8b**	10	95.5	1.4	95.3	7.3	85.9	7.3
	50	82.3	3.5	76.9	9.4	72.8	2.8
**8c**	10	70.2	5.8	73.4	3.9	64.1	3.5
	50	5.7	1.0	42.5	6.6	19.3	2.8
**8d**	10	91.1	11.1	76.0	5.7	90.0	5.6
	50	18.5	3.4	37.3	8.1	41.1	8.4
**8e**	10	69.6	9.2	85.2	13.3	76.1	4.0
	50	11.8	2.1	40.6	3.2	31.2	4.8
**9a**	10	64.9	6.8	62.0	4.1	66.9	6.6
	50	43.4	5.2	28.6	2.3	3.3	1.2
**9b**	10	76.8	18.1	64.3	6.7	79.0	11.8
	50	62.8	6.2	36.2	1.7	19.0	6.1
**9c**	10	63.0	11.1	47.9	8.2	64.2	1.3
	50	49.2	7.3	37.3	4.1	7.1	1.3

**9d**	10	91.1	9.2	61.2	8.2	84.5	6.4
	50	70.2	0.4	36.0	6.2	79.3	7.2
**9e**	10	54.8	7.8	45.4	7.5	32.9	6.7
	50	44.4	11.6	33.5	5.5	9.0	2.4
**10a**	10	37.8	7.7	57.3	4.2	44.0	7.9
	50	22.4	5.7	49.5	5.1	13.1	4.5
**10b**	10	63.0	3.3	63.3	9.1	16.4	3.2
	50	7.4	0.5	43.9	3.9	12.0	2.3
**10c**	10	52.7	5.4	55.7	6.7	78.2	4.2
	50	15.6	3.1	35.5	4.3	5.1	0.7
**10d**	10	60.6	1.6	66.5	4.5	79.6	5.1
	50	8.4	1.0	13.9	1.4	5.1	0.7
**10e**	10	55.6	1.9	54.4	4.0	45.5	8.9
	50	16.9	4.5	21.4	2.0	6.5	1.4

2. SUPPLEMENTARY DATA FOR DPPH AND ABTS ASSAYS

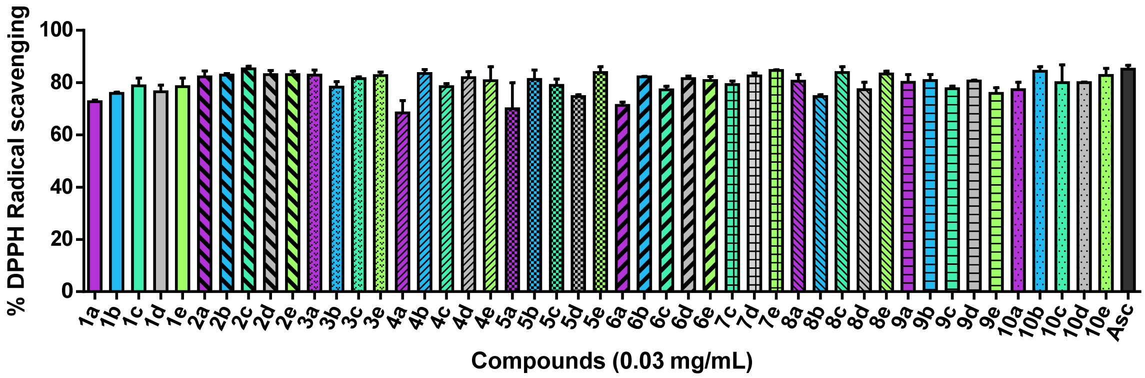

Figure S1. DPPH scavenging activity at a concentration of 0.03 mg/mL and after 60 minutes.

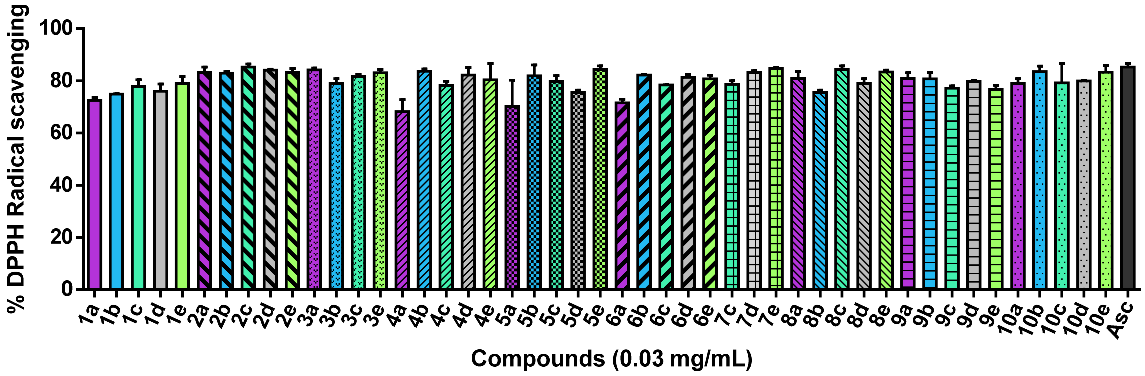

Figure S2. DPPH scavenging activity at a concentration of 0.03 mg/mL and after 90 minutes.

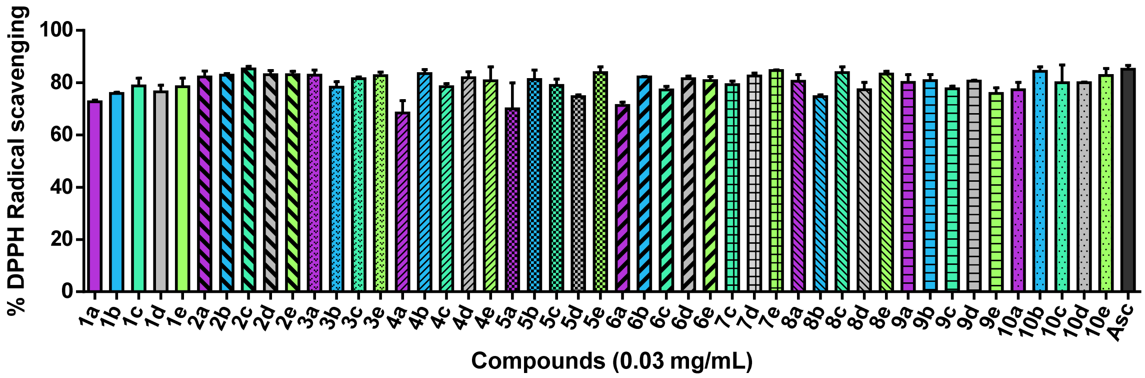

Figure S3. DPPH scavenging activity at a concentration of 0.03 mg/mL and after 120 minutes.

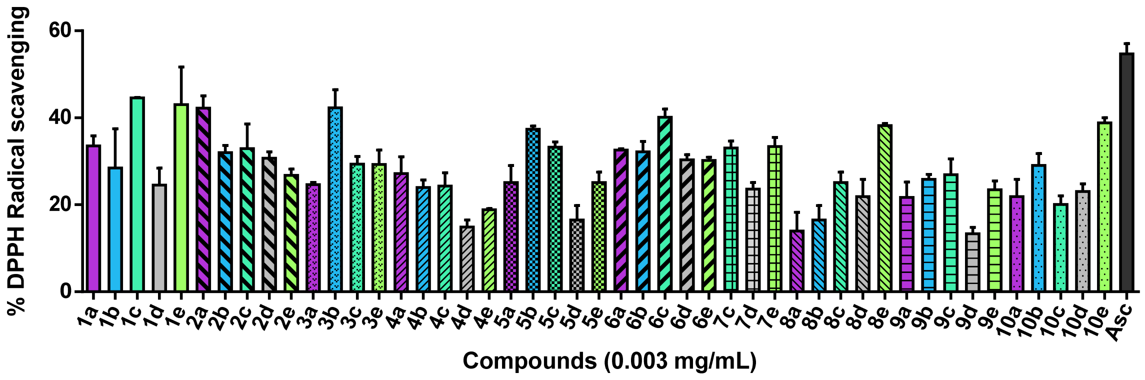

Figure S4. DPPH scavenging activity at a concentration of 0.003 mg/mL and after 30 minutes.

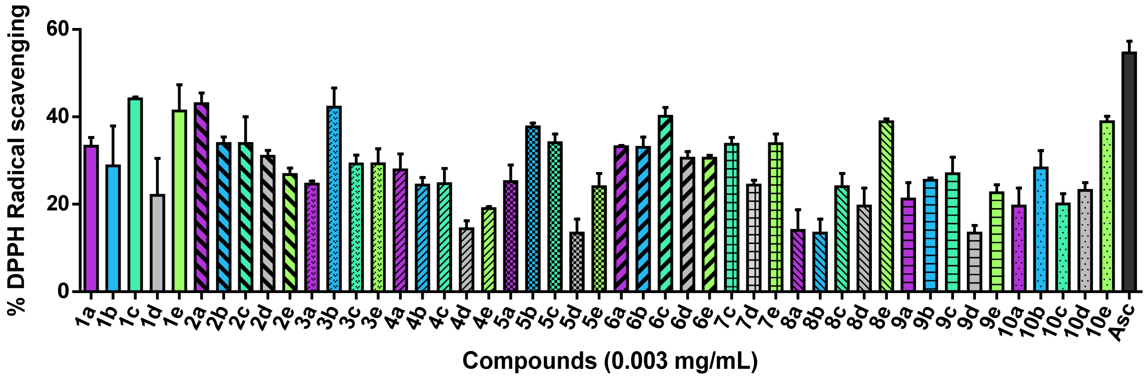

Figure S5. DPPH scavenging activity at a concentration of 0.003 mg/mL and after 60 minutes.

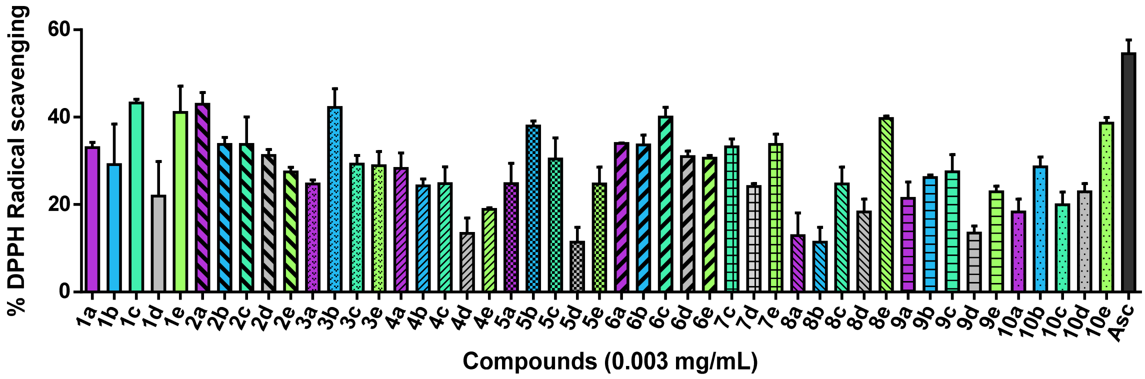

Figure S6. DPPH scavenging activity at a concentration of 0.003 mg/mL and after 90 minutes.

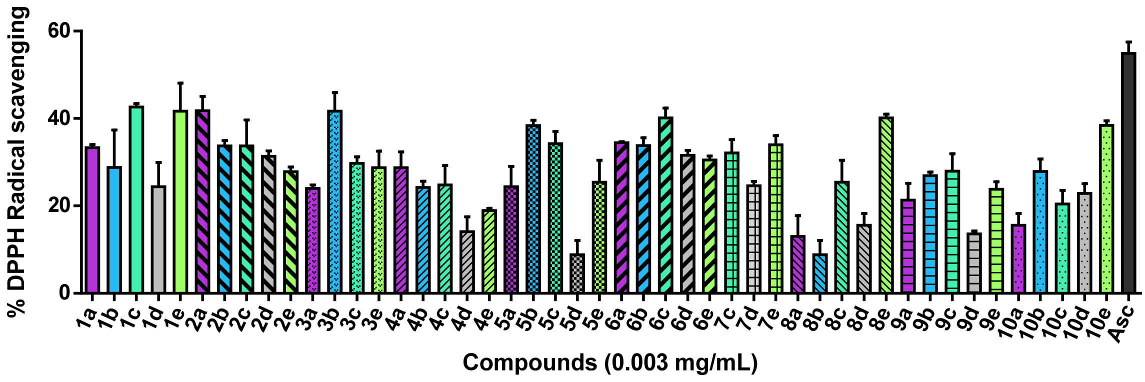

Figure S7. DPPH scavenging activity at a concentration of 0.003 mg/mL and after 120 minutes.

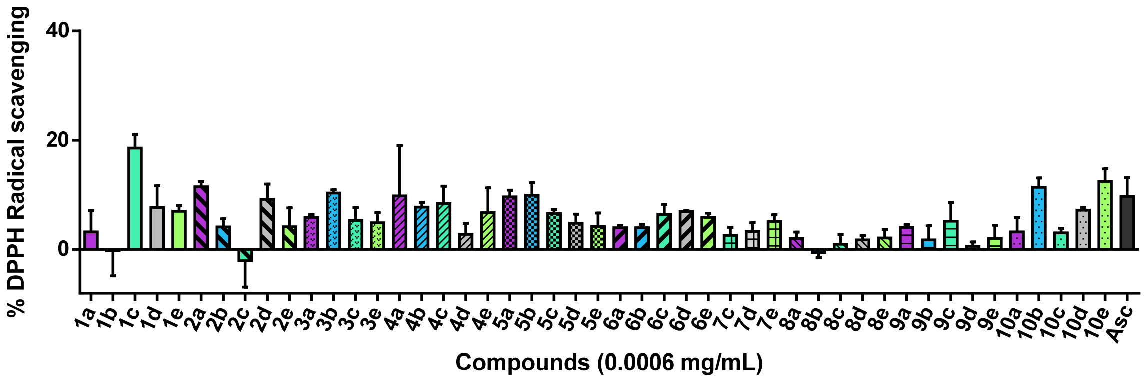

Figure S8. DPPH scavenging activity at a concentration of 0.0006 mg/mL and 0 minutes.

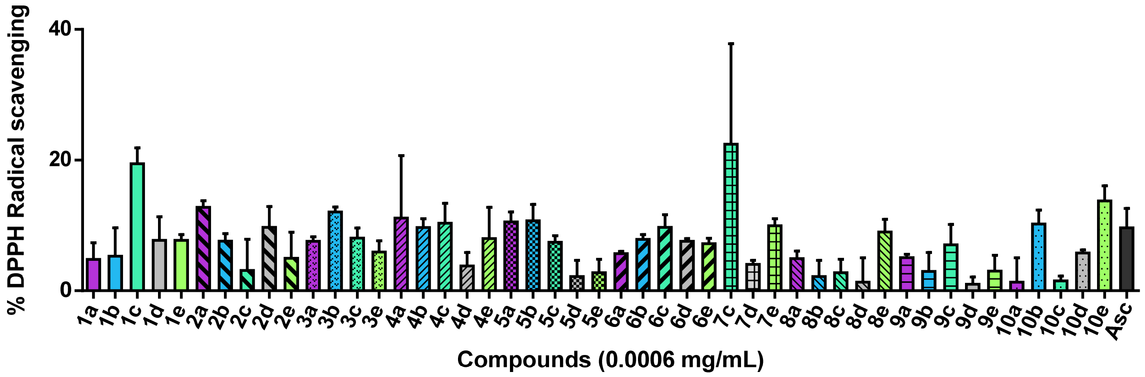

Figure S9. DPPH scavenging activity at a concentration of 0.0006 mg/mL and after 30 minutes.

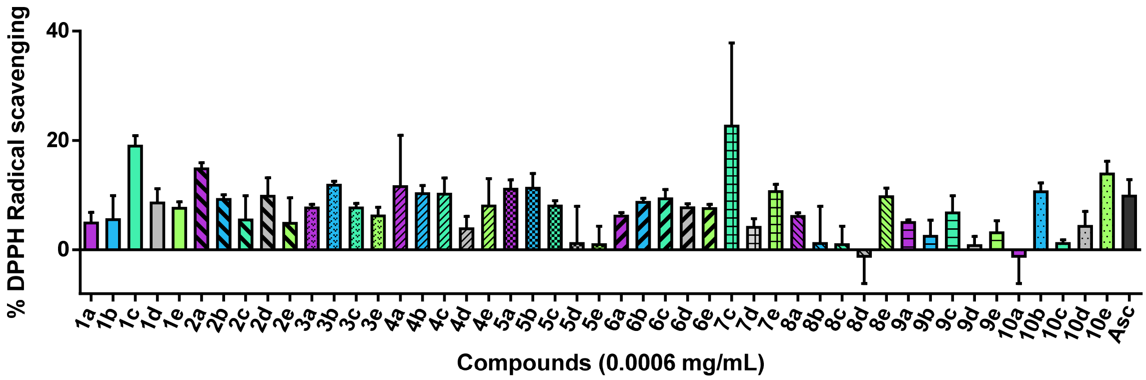

Figure S10. DPPH scavenging activity at a concentration of 0.0006 mg/mL and after 60 minutes.

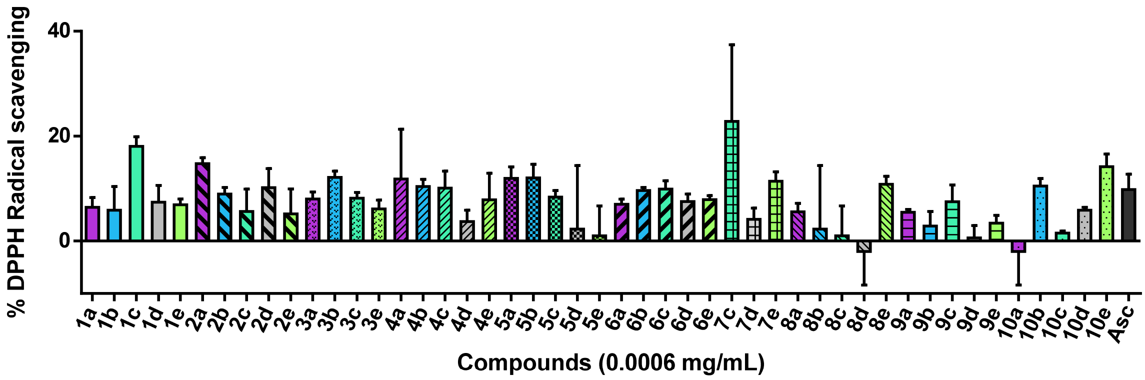

Figure S11. DPPH scavenging activity at a concentration of 0.0006 mg/mL and after 90 minutes.

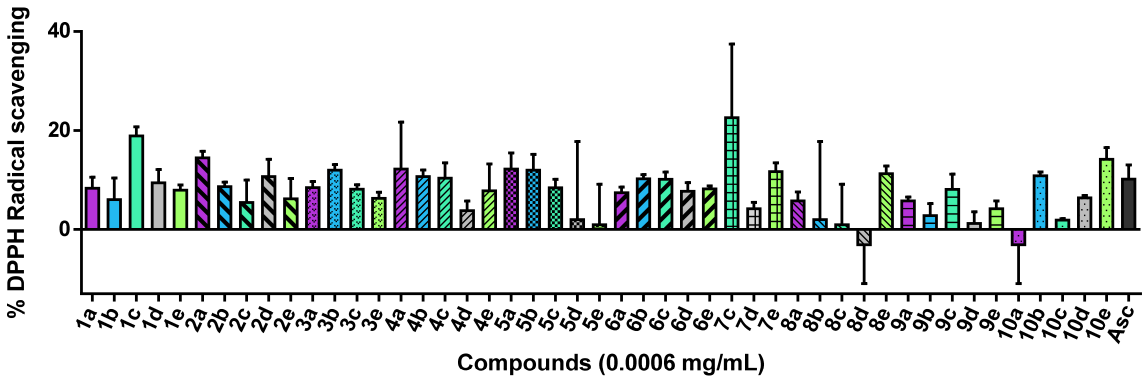

Figure S12. DPPH scavenging activity at a concentration of 0.0006 mg/mL and after 120 minutes.

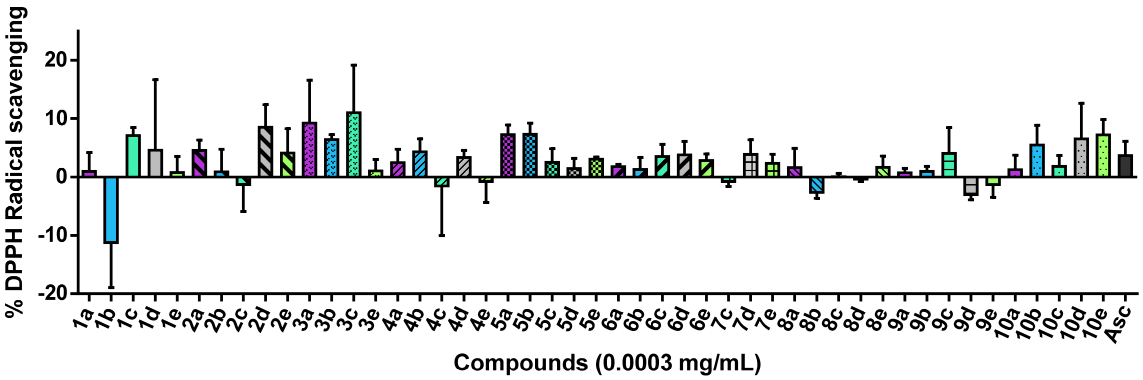

Figure S13. DPPH scavenging activity at a concentration of 0.0003 mg/mL and 0 minutes.

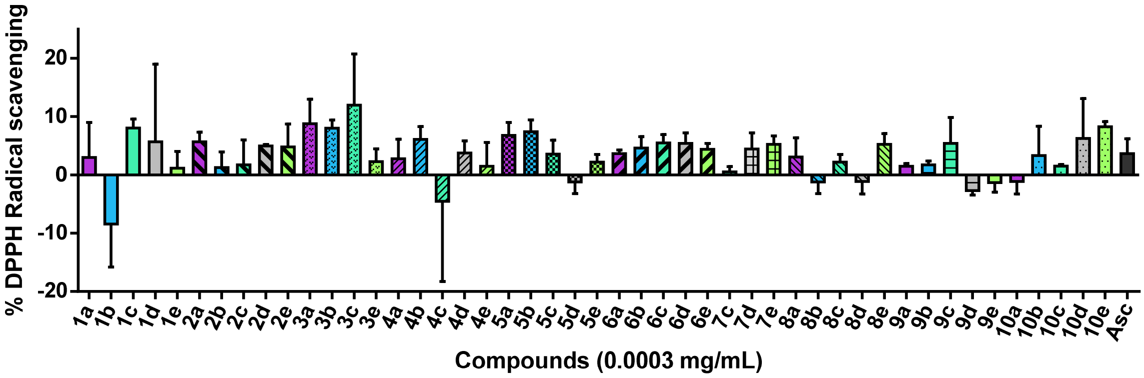

Figure S14. DPPH scavenging activity at a concentration of 0.0003 mg/mL and after 30 minutes.

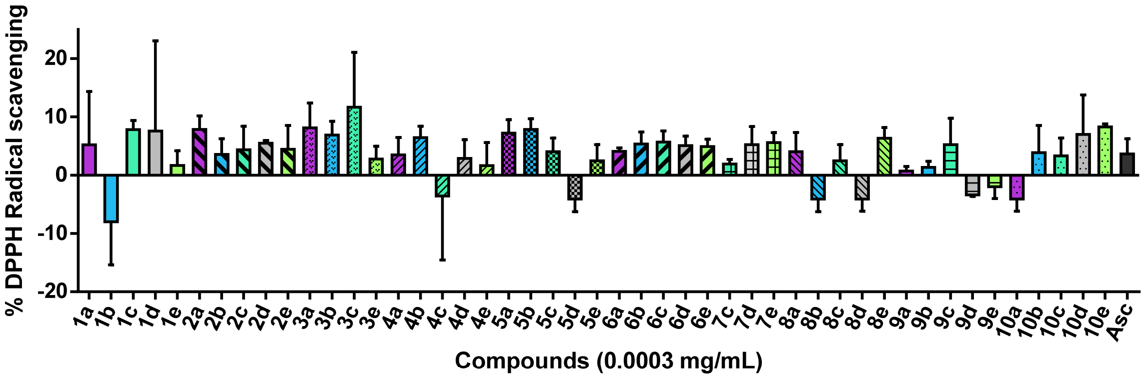

Figure S15. DPPH scavenging activity at a concentration of 0.0003 mg/mL and after 60 minutes.

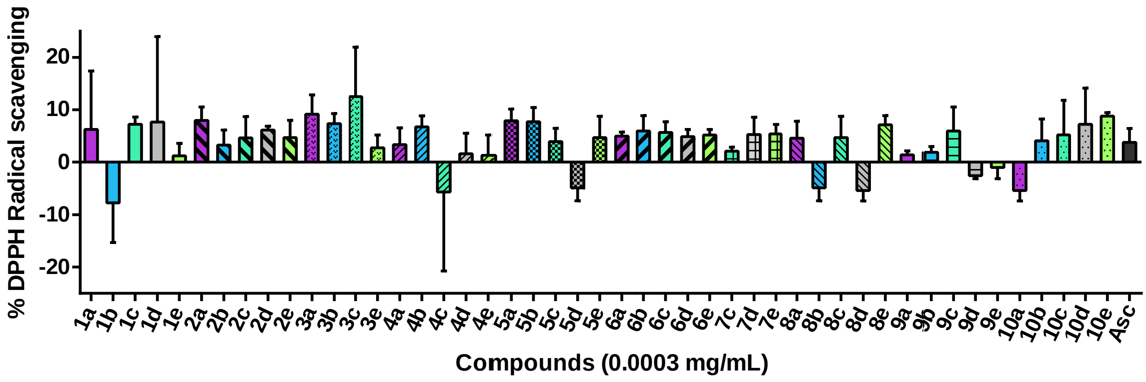

Figure S16. DPPH scavenging activity at a concentration of 0.0003 mg/mL and after 90 minutes.

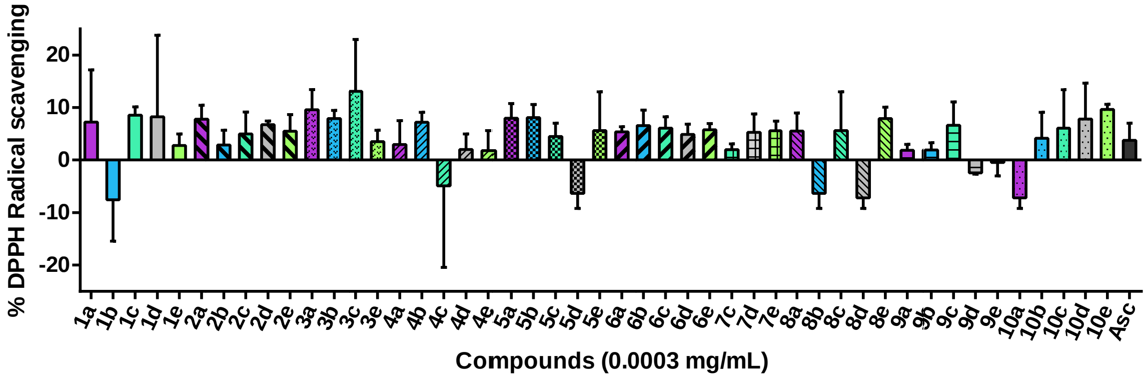

Figure S17. DPPH scavenging activity at a concentration of 0.0003 mg/mL and after 120 minutes.

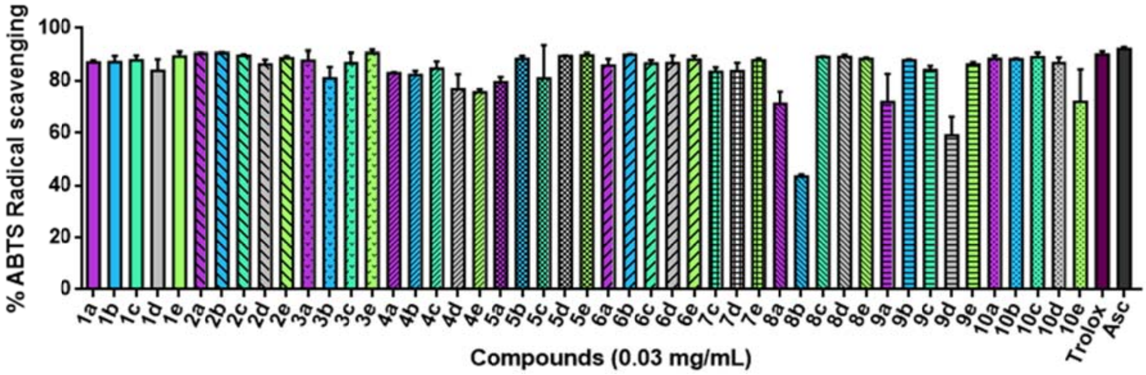

Figure S18. ABTS scavenging activity at a concentration of 0.03 mg/m**L** and after 30 minutes.

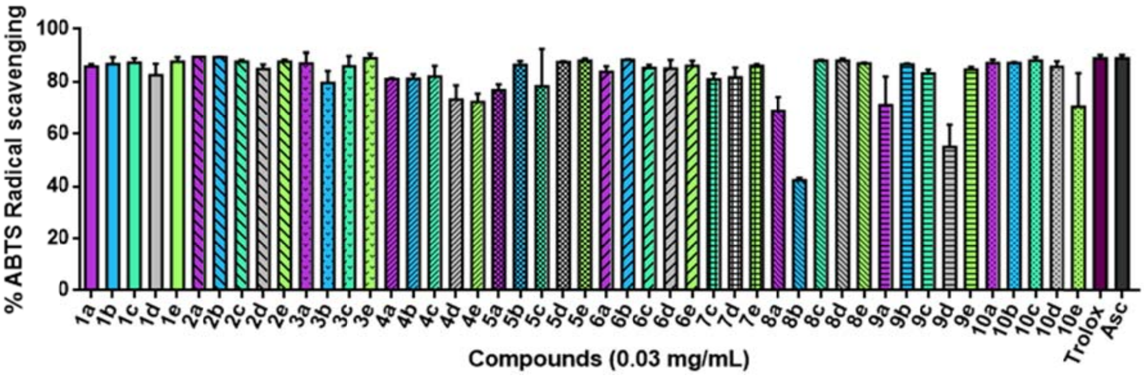

Figure S19. ABTS scavenging activity at a concentration of 0.03 mg/m**L** and after 60 minutes.

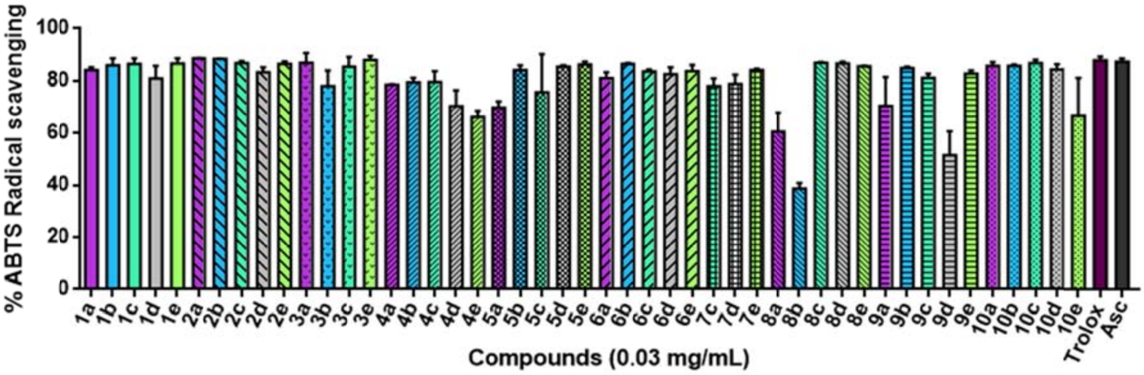

Figure S20. ABTS scavenging activity at a concentration of 0.03 mg/m**L** and after 90 minutes.

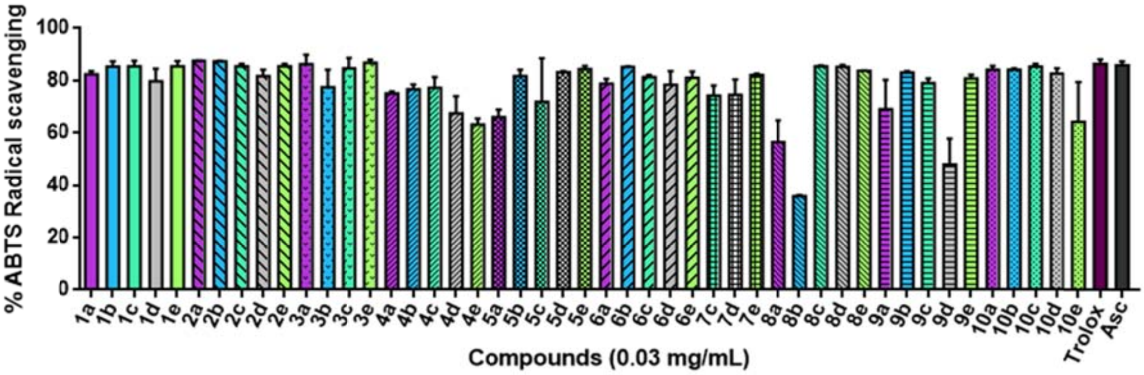

Figure S21. ABTS scavenging activity at a concentration of 0.03 mg/mL and after 120 minutes.

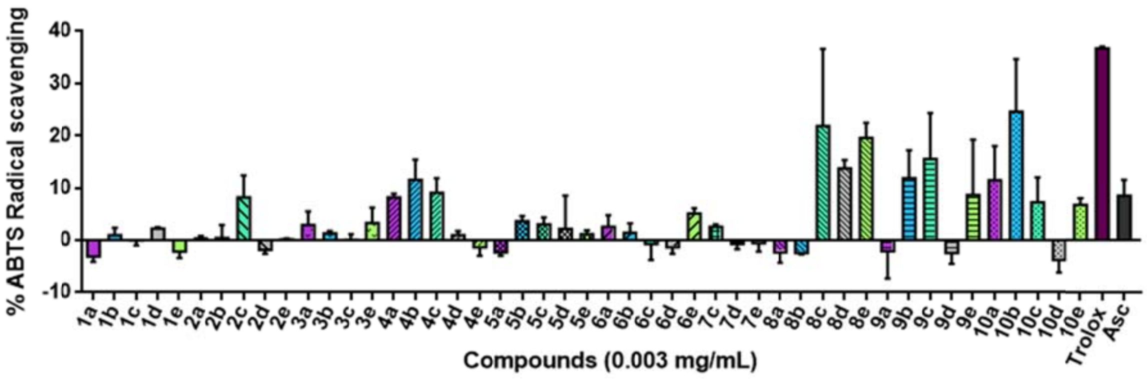

Figure S22. ABTS scavenging activity at a concentration of 0.003 mg/mL and after 6 minutes of incubation.

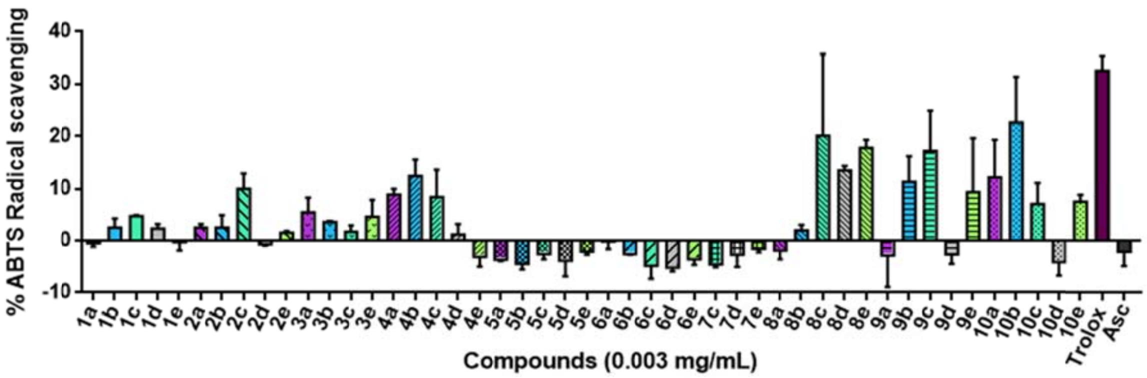

Figure S23. ABTS scavenging activity at a concentration of 0.003 mg/mL and after 30 minutes.

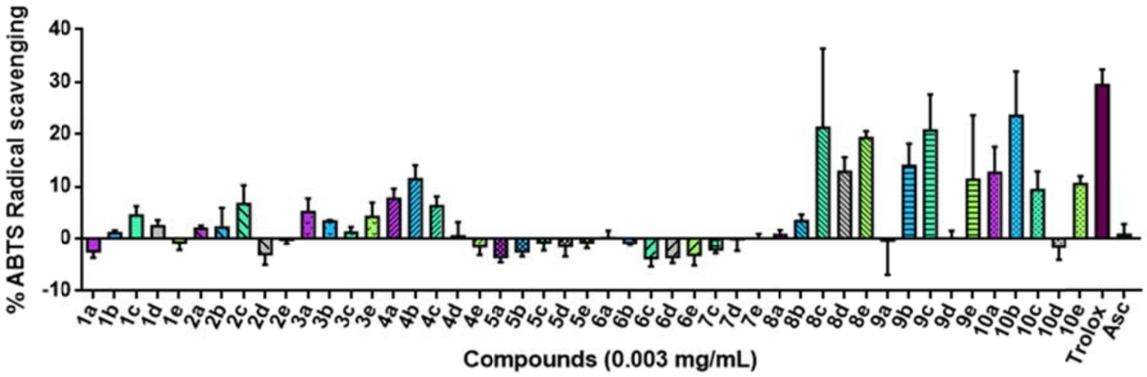

Figure S24. ABTS scavenging activity at a concentration of 0.003 mg/mL and after 60 minutes.

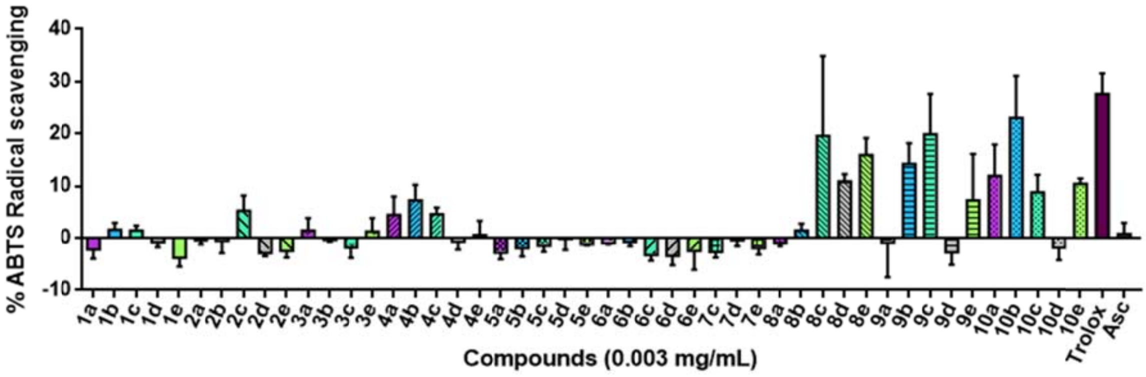

Figure S25. ABTS scavenging activity at a concentration of 0.003 mg/mL and after 90 minutes.

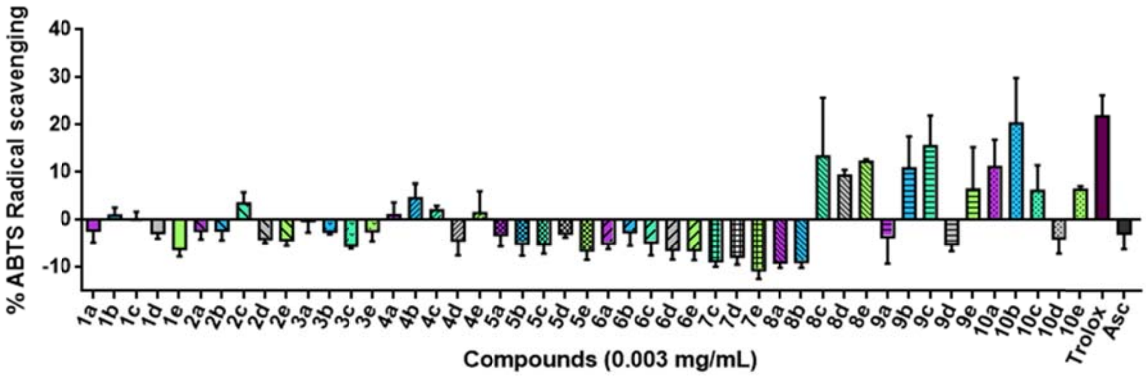

Figure S26. ABTS scavenging activity at a concentration of 0.003 mg/mL and after 120 minutes.

3. CHARACTERIZATION

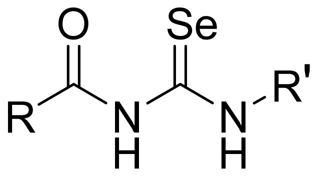

Figure S27. General structure of the new acylselenoureas.

N-(phenylcarbamoselenoyl)benzamide **(1a)**. Brown solid; yield: 45%. ^{1}H-NMR (400 MHz, $CDCl_3$) δ: 12.98 (1H, s, CONH), 11.80 (1H, s, Ph-NH), 7.99 (2H, d, *J* = 7.6 Hz, R-H), 7.68 (2H, d, *J* = 7.7 Hz, R′-H), 7.67-7.65 (1H, m, R-H), 7.55 (2H, t, *J* = 7.6 Hz, R-H), 7.45 (2H, t, *J* = 7.7 Hz, R′-H), 7.33 (1H, t, *J* = 7.7 Hz, R′-H). ^{13}C-NMR (100 MHz, $CDCl_3$) δ: 181.0, 168.6, 139.4 133.7, 132.4, 129.2, 129.2, 128.9, 127.4, 125.6. ^{77}Se-NMR (75 MHz, $CDCl_3$) δ: 416.

N-[(4-methylphenyl)carbamoselenoyl]benzamide **(1b)**. Brown solid; yield: 55%. ^{1}H-NMR (400 MHz, $CDCl_3$) δ: 12.94 (1H, s, CONH), 11.76 (1H, s, Ph-NH), 7.98 (2H, d, *J* = 7.3 Hz, R-H), 7.68 (1H, t, *J* = 7.3 Hz, R-H), 7.56-7.52 (4H, m, R′-H+R-H), 7.24 (2H, d, *J* = 8.1 Hz, R′-H), 2.33 (3H, s, CH_3). ^{13}C-NMR (100 MHz, $CDCl_3$) δ: 180.8, 168.6, 136.9, 136.8, 133.7, 132.4, 129.6, 129.2, 128.9, 125.4, 21.1. ^{77}Se-NMR (76 MHz, $CDCl_3$) δ: 412.

N-[(4-methoxyphenyl)carbamoselenoyl]benzamide **(1c)**. Brown solid; yield: 66%. ^{1}H-NMR (400 MHz, $CDCl_3$) δ: 12.85 (1H, s, CONH), 11.75 (1H, s, Ph-NH), 7.98 (2H, d, *J* = 7.3 Hz, R-H), 7.68 (1H, t, *J* = 7.3 Hz, R-H), 7.56 (2H, d, *J* = 8.9 Hz, R′-H), 7.55-7.53 (2H, m, R-H), 6.99 (2H, d, *J* = 8.9 Hz, R′-H), 3.79 (3H, s, OCH_3). ^{13}C-NMR (100 MHz, $CDCl_3$) δ: 180.9, 168.6, 158.3, 133.7, 132.4, 132.3, 129.2, 128.9, 127.1, 114.3, 55.8. ^{77}Se-NMR (76 MHz, $CDCl_3$) δ: 405.

N-[(4-trifluoromethylphenyl)carbamoselenoyl]benzamide **(1d)**. Brown solid; yield: 90%. ^{1}H-NMR (400 MHz, $CDCl_3$) δ: 13.06 (1H, s, CONH), 11.98 (1H, s, Ph-NH), 7.99 (2H, d, *J* = 7.4 Hz, R-H), 7.95 (2H, d, *J* = 8.3 Hz, R′-H), 7.81 (2H, d, *J* = 8.3 Hz, R′-H), 7.69 (1H, t, *J* = 7.4Hz, R-H), 7.56 (2H, t, *J* = 7.4 Hz, R-H). ^{13}C-NMR (100 MHz, $CDCl_3$) δ: 181.6, 168.4, 143.2, 133.8, 132.3, 129.3, 128.9, 126.3. ^{77}Se-NMR (76 MHz, $CDCl_3$) δ: 436.

N-[(4-chlorophenyl)carbamoselenoyl]benzamide **(1e)**. Yellow solid; yield: 95%. ^{1}H-NMR (400 MHz, $CDCl_3$) δ: 12.92 (1H, s, CONH), 11.87 (1H, s, Ph-NH), 7.98 (2H, d, *J* = 7.5 Hz, R-H), 7.71 (2H, d, *J* = 8.5 Hz, R′-H), 7.69-7.65 (2H, m, R-H), 7.55 (1H, t, *J* = 7.5 Hz, R-H), 7.50 (2H, d, *J* = 8.5 Hz, R′-H). ^{13}C-NMR (100 MHz, $CDCl_3$) δ: 181.4, 168.5, 138.5, 133.7, 132.4, 131.5, 129.3, 129.1, 128.9, 127.7. ^{77}Se-NMR (76 MHz, $CDCl_3$) δ: 423.

2-chloro-N-(phenylcarbamoselenoyl)benzamide **(2a)**. Orange solid; yield: 11%. ^{1}H-NMR (400 MHz, $CDCl_3$) δ: 12.76 (1H, s, CONH), 12.31 (1H, s, Ph-NH), 7.70 (2H, d, *J* = 7.7 Hz, R′-H), 7.65 (1H, d, *J* = 7.5 Hz, R-H), 7.58-7.53 (2H, m, R′-H), 7.49-7.43 (3H, m, R-H), 7.34 (1H, t, *J* = 7.7 Hz, R′-H). ^{13}C-NMR (100 MHz, $CDCl_3$) δ: 180.6, 168.0, 139.3, 134.6, 132.6, 130.5, 130.0, 129.8, 129.2, 127.6, 127.5, 125.6. ^{77}Se-NMR (76 MHz, $CDCl_3$) δ: 423.

2-chloro-N-[(4-methylphenyl)carbamoselenoyl]benzamide **(2b)**. Orange solid; yield: 36%. ^{1}H-NMR (400 MHz, $CDCl_3$) δ: 12.71 (1H, s, CONH), 12.27 (1H, s, Ph-NH), 7.64 (1H, d, *J* = 7.6 Hz, R-H), 7.57 (2H, d, *J* = 3.2 Hz, R-H), 7.56-7.53 (2H, m, R′-H), 7.46 (1H, t, *J* = 7.6 Hz, R-H), 7.25 (2H, d, *J* = 8.1 Hz, R′-H), 2.33 (3H, s, CH_3). ^{13}C-NMR (100 MHz, $CDCl_3$) δ: 180.4, 168.0, 136.9, 136.8, 134.6, 132.6, 130.5, 130.0, 129.8, 129.6, 127.5, 125.4, 21.2. ^{77}Se-NMR (76 MHz, $CDCl_3$) δ: 419.

2-chloro-N-[(4-methoxyphenyl)carbamoselenoyl]benzamide **(2c)**. Orange solid; yield: 15%. ^{1}H-NMR (400 MHz, $CDCl_3$) δ: 12.63 (1H, s, CONH), 12.25 (1H, s, Ph-NH), 7.64 (1H, d, *J* = 7.5 Hz, R-H), 7.57 (2H, d, *J* = 7.5 Hz, R-H), 7.57-7.53 (2H, m, R′-H), 7.45 (1H, t, *J* = 7.5 Hz, R-H), 6.99 (2H, d, *J* = 8.9 Hz, R′-H), 3.80 (3H, s, OCH_3). ^{13}C-NMR (100 MHz, $CDCl_3$) δ: 180.6, 168.0, 158.4, 134.6, 132.6, 132.2, 130.5, 130.0, 129.8, 127.5, 127.1, 114.3, 55.8. ^{77}Se-NMR (76 MHz, $CDCl_3$) δ: 411.

2-chloro-N-[(4-trifluoromethylphenyl)carbamoselenoyl]benzamide **(2d)**. Orange solid; yield: 18%. ^{1}H-NMR (400 MHz, $CDCl_3$) δ: 13.02 (1H, s, CONH), 9.69 (1H, s, Ph-NH), 7.96 (2H, d, *J* = 8.5 Hz, R′-H), 7.80 (1H, dd, *J* = 7.8, 1.6 Hz, R-H), 7.71 (2H, d, *J* = 8.5 Hz, R′-H), 7.56-7.51 (2H, m, R-H), 7.49-7.43 (1H, m, R-H). ^{13}C-NMR (100 MHz, $CDCl_3$) δ: 179.8, 166.0, 133.8, 131.4, 131.3, 131.2, 130.8, 127.7, 126.3, 126.2, 124.4. ^{77}Se-NMR (76 MHz, $CDCl_3$) δ: 460.

2-chloro-N-[(4-chlorophenyl)carbamoselenoyl]benzamide **(2e)**. Pink solid; yield: 26%. ^{1}H-NMR (400 MHz, $CDCl_3$) δ: 12.69 (1H, s, CONH), 12.37 (1H, s, Ph-NH), 7.71 (2H, d, *J* = 8.3 Hz, R′-H), 7.64 (1H, d, *J* = 7.2 Hz, R-H), 7.59-7.53 (2H, m, R-H), 7.50 (2H, d, *J* = 8.3 Hz, R′-H), 7.50-7.45 (1H, m, R-H). ^{13}C-NMR (100 MHz, $CDCl_3$) δ: 181.0, 167.8, 138.4, 134.5, 132.7, 131.5, 130.5, 130.0, 129.8, 129.1, 127.7, 127.6. ^{77}Se-NMR (76 MHz, $CDCl_3$) δ: 430.

2-chloro-5-methylthio-N-(phenylcarbamoselenoyl)benzamide **(3a)**. Yellow solid; yield: 12%. ^{1}H-NMR (400 MHz, $CDCl_3$) δ: 12.74 (1H, s, CONH), 12.32 (1H, s, Ph-NH), 7.69 (2H, d, *J* = 7.8 Hz, R′-H), 7.53 (1H, d, *J* = 2.1 Hz, R-H), 7.46 (2H, d, *J* = 7.8 Hz, R′-H), 7.44-7.40 (2H, m, R-H), 7.34 (1H, t, *J* = 7.8 Hz, R′-H), 2,54 (3H, s, SCH_3). ^{13}C-NMR (100 MHz, $CDCl_3$) δ: 180.6, 167.5, 139.3, 138.2, 134.8, 130.3, 129.6, 129.2, 127.5, 126.6, 126.6, 125.6, 15.2. ^{77}Se-NMR (76 MHz, $CDCl_3$) δ: 425.

2-chloro-5-methylthio-N-[(4-methylphenyl)carbamoselenoyl]benzamide **(3b)**. Grey solid; yield: 12%. ^{1}H-NMR (400 MHz, $CDCl_3$) δ: 12.69 (1H, s, CONH), 12.28 (1H, s, Ph-NH), 7.56 (2H, d, *J* = 8.3 Hz, R′-H), 7.52 (1H, d, *J* = 2.3 Hz, R-H), 7.48 (1H, d, *J* = 8.5 Hz, R-H), 7.41 (1H, dd, *J* = 8.5, 2.3 Hz, R-H), 7.25 (2H, d, *J* = 8.3 Hz, R′-H), 2,54 (3H, s, SCH_3), 2.33 (3H, s, CH_3). ^{13}C-NMR (100 MHz, $CDCl_3$) δ: 180.4, 167.5, 138.2, 136.9, 136.7, 134.8, 130.3, 129.7, 129.6, 126.6, 126.5, 125.4, 21.2, 15.2. ^{77}Se-NMR (76 MHz, $CDCl_3$) δ: 421.

2-chloro-5-methylthio-N-[(4-methoxyphenyl)carbamoselenoyl]benzamide **(3c)**. Grey solid; yield: 11%. ^{1}H-NMR (400 MHz, $CDCl_3$) δ: 12.61 (1H, s, CONH), 12.26 (1H, s, Ph-NH), 7.56 (2H, d, *J* = 8.9 Hz, R′-H), 7.52 (1H, d, *J* = 2.3 Hz, R-H), 7.48 (1H, d, *J* = 8.5 Hz, R-H), 7.41 (1H, dd, *J* = 8.5, 2.3 Hz, R-H), 7.00 (2H, d, *J* = 8.9 Hz, R′-H), 3.80 (3H, s, OCH_3), 2,54 (3H, s, SCH_3). ^{13}C-NMR (100 MHz, $CDCl_3$) δ: 180.6, 167.5, 158.4, 138.2, 134.9, 130.3, 129.5, 127.1, 126.5, 114.3, 55.8, 15.2. ^{77}Se-NMR (76 MHz, $CDCl_3$) δ: 413.

2-chloro-5-methylthio-N-[(4-chlorophenyl)carbamoselenoyl]benzamide **(3e)**. Grey solid; yield: 24%. ^{1}H-NMR (400 MHz, $CDCl_3$) δ: 12.67 (1H, s, CONH), 12.37 (1H, s, Ph-NH), 7.70 (2H, d, *J* = 8.6 Hz, R′-H), 7.52-7.51 (2H, m, R-H), 7.50-7.47 (2H, m, R′-H), 7.42 (1H, dd, *J* = 8.5, 2.3 Hz, R-H), 2,54 (3H, s, SCH_3). ^{13}C-NMR (100 MHz, $CDCl_3$) δ: 181.1, 167.3, 138.4, 138.2, 134.8, 131.6, 130.3, 129.6, 129.1, 127.7, 126.6, 126.5, 15.2. ^{77}Se-NMR (76 MHz, $CDCl_3$) δ: 431.

2-chloro-N-(phenylcarbamoselenoyl)nicotinamide **(4a)**. Orange solid; yield: 28%. ^{1}H-NMR (400 MHz, $CDCl_3$) δ: 12.61 (1H, s, CONH), 9.79 (1H, s, Ph-NH), 8.55 (1H, dd, *J* = 4.7, 1.8 Hz, R-H), 8.12 (1H, dd, *J* = 7.7, 1.8 Hz, R-H), 7.66 (2H, d, *J* = 7.8 Hz, R′-H), 7.42-7.40 (1H, m, R-H), 7.38 (2H, d, *J* = 7.8 Hz, R′-H), 7.30 (1H, t, *J* = 7.8 Hz, R′-H). ^{13}C-NMR (100 MHz, $CDCl_3$) δ: 178.3, 163.2, 151.9, 146.3, 139.1, 137.2, 128.1, 127.4, 126.8, 123.6, 122.0. ^{77}Se-NMR (76 MHz, $CDCl_3$) δ: 443.

2-chloro-N-[(4-methylphenyl)carbamoselenoyl]nicotinamide **(4b)**. Yellow solid; yield: 43%. ^{1}H-NMR (400 MHz, $CDCl_3$) δ: 12.51 (1H, s, CONH), 9.75 (1H, s, Ph-NH), 8.55 (1H, dd, *J* = 4.8, 1.8 Hz, R-H), 8.12 (1H, dd, *J* = 7.7, 1.8 Hz, R-H), 7.51 (2H, d, *J* = 7.3 Hz, R′-H), 7.40 (1H, dd, *J* = 7.7, 4.8 Hz, R-H), 7.18

(2H, d, *J* = 7.3 Hz, R′-H), 2.32 (3H, s, CH_3). ^{13}C-NMR (100 MHz, $CDCl_3$) δ: 178.2, 163.1, 151.8, 139.0, 136.9, 134.6, 128.7, 127.4, 123.5, 122.0, 20.2. ^{77}Se-NMR (76 MHz, $CDCl_3$) δ: 576.

2-chloro-N-[(4-methoxyphenyl)carbamoselenoyl]nicotinamide **(4c)**. Yellow solid; yield: 40%. ^{1}H-NMR (400 MHz, $CDCl_3$) δ: 12.46 (1H, s, CONH), 9.84 (1H, s, Ph-NH), 8.54 (1H, d, *J* = 4.8 Hz, R-H), 8.10 (1H, d, *J* = 7.6 Hz, R-H), 7.52 (2H, d, *J* = 8.8 Hz, R′-H), 7.39 (1H, dd, *J* = 7.6, 4.8 Hz, R-H), 6.90 (2H, d, *J* = 8.8 Hz, R′-H), 3.78 (3H, s, OCH_3). ^{13}C-NMR (100 MHz, $CDCl_3$) δ: 179.5, 164.3, 158.9, 152.8, 147.4, 140.0, 131.1, 128.6, 126.2, 123.0, 114.3, 55.51. ^{77}Se-NMR (76 MHz, $CDCl_3$) δ: 424.

2-chloro-N-[(4-trifluoromethylphenyl)carbamoselenoyl]nicotinamide **(4d)**. Orange solid; yield: 51%. ^{1}H-NMR (400 MHz, $CDCl_3$) δ: 12.82 (1H, s, CONH), 9.87 (1H, s, Ph-NH), 8.56 (1H, dd, *J* = 4.8, 2.0 Hz, R-H), 8.14 (1H, dd, *J* = 7.7, 2.0 Hz, R-H), 7.88 (2H, d, *J* = 8.5 Hz, R′-H), 7.64 (2H, d, *J* = 8.5 Hz, R′-H), 7.41 (1H, dd, *J* = 7.7, 4.8 Hz, RH). ^{13}C-NMR (100 MHz, $CDCl_3$) δ: 178.6, 163.4, 152.1, 140.1, 139.1, 127.2, 125.3, 123.4, 122.1, 120.1, 118.8. ^{77}Se-NMR (76 MHz, $CDCl_3$) δ: 478.

2-chloro-N-[(4-chlorophenyl)carbamoselenoyl]nicotinamide **(4e)**. Orange solid; yield: 53%. ^{1}H-NMR (400 MHz, $CDCl_3$) δ: 12.62 (1H, s, CONH), 9.86 (1H, s, Ph-NH), 8.56 (1H, dd, *J* = 4.8, 1.6 Hz, R-H), 8.12 (1H, dd, *J* = 7.7, 1.6 Hz, R-H), 7.63 (2H, d, *J* = 8.7 Hz, R′-H), 7.41 (1H, dd, *J* = 7.7, 4.8 Hz, R-H), 7.35 (2H, d, *J* = 8.7 Hz, R′-H). ^{13}C-NMR (100 MHz, $CDCl_3$) δ: 179.6, 164.4, 153.0, 140.1, 136.7, 133.2, 129.3, 128.4, 128.3, 125.9, 123.1. ^{77}Se-NMR (76 MHz, $CDCl_3$) δ: 455.

N-(phenylcarbamoselenoyl)-2-thiophencarboxamide **(5a)**. Yellow crystals; yield: 35%. ^{1}H-NMR (400 MHz, $CDCl_3$) δ: 12.73 (1H, s, CONH), 9.24 (1H, s, Ph-NH), 7.68 (2H, dd, *J* = 4.6, 3.5 Hz, R-H), 7.62 (2H, d, *J* = 7.8 Hz, R′-H), 7.36 (2H, t, *J* = 7.8 Hz, R′-H), 7.26 (1H, t, *J* = 7.8 Hz, R′-H), 7.13 (1H, dd, *J* = 4.6, 3.5 Hz, R-H).^{13}C-NMR (100 MHz, $CDCl_3$) δ: 179.7, 161.1, 138.4, 135.6, 134.7, 131.1, 129.0, 128.7, 127.6, 124.7. ^{77}Se-NMR (76 MHz, $CDCl_3$) δ: 408.

N-[(4-methylphenyl)carbamoselenoyl]-2-thiophencarboxamide **(5b)**. Yellow solid; yield: 50%. ^{1}H-NMR (400 MHz, $CDCl_3$) δ: 12.63 (1H, s, CONH), 9.26 (1H, s, Ph-NH), 7.68 (1H, d, *J* = 4.5 Hz, R-H), 7.67 (1H, d, *J* = 4.5 Hz, R-H), 7.46 (2H, d, *J* = 8.3 Hz, R′-H), 7.15 (2H, d, *J* = 8.3 Hz, R′-H), 7.12 (1H, t, *J* = 4.5 Hz, R-H), 2.29 (3H, s, CH_3). ^{13}C-NMR (100 MHz, $CDCl_3$) δ: 178.7, 160.1, 136.6, 134.9, 134.6, 133.6, 130.0, 128.6, 127.6, 123.6, 20.2. ^{77}Se-NMR (76 MHz, $CDCl_3$) δ: 400.

N-[(4-methoxyphenyl)carbamoselenoyl]-2-thiophencarboxamide **(5c)**. Yellow solid; yield: 46%. ^{1}H-NMR (400 MHz, $CDCl_3$) δ: 12.56 (1H, s, CONH), 9.25 (1H, s, Ph-NH), 7.67 (2H, t, *J* = 4.0 Hz, R-H), 7.48 (2H, d, *J* = 8.9 Hz, R′-H), 7.12 (1H, dd, *J* = 5.0, 4.0 Hz, R-H), 6.87 (2H, d, *J* = 8.9 Hz, R′-H), 3.75 (3H, s, OCH_3). ^{13}C-NMR (100 MHz, $CDCl_3$) δ: 178.8, 160.1, 157.7, 134.6, 133.6, 130.4, 127.6, 125.2, 113.2, 54.5. ^{77}Se-NMR (76 MHz, $CDCl_3$) δ: 393.

N-[(4-trifluoromethylphenyl)carbamoselenoyl]-2-thiophencarboxamide **(5d)**. Yellow crystals; yield: 32%. ^{1}H-NMR (400 MHz, $CDCl_3$) δ: 12.95 (1H, s, CONH), 9.29 (1H, s, Ph-NH), 7.83 (2H, d, *J* = 7.3 Hz, R-H), 7.72-7.68 (2H, m, R′-H), 7.61 (2H, d, *J* = 7.0 Hz, R′-H), 7.16-7.11 (1H, m, R-H). ^{13}C-NMR (100 MHz, $CDCl_3$) δ: 179.0, 160.2, 140.3, 134.3, 134.0, 130.2, 127.7, 125.2, 123.4, 118.9. ^{77}Se-NMR (76 MHz, $CDCl_3$) δ: 438.

N-[(4-chlorophenyl)carbamoselenoyl]-2-thiophencarboxamide **(5e)**. Yellow solid; yield: 53%. ^{1}H-NMR (400 MHz, $CDCl_3$) δ: 12.74 (1H, s, CONH), 9.27 (1H, s, Ph-NH), 7.71-7.67 (2H, m, R′H), 7.58 (2H, d, *J* = 8.9 Hz, R′-H), 7.31 (2H, d, *J* = 8.9 Hz, R′-H), 7.12 (1H, dd, *J* = 4.8, 4.0 Hz, R′H). ^{13}C-NMR (100 MHz, $CDCl_3$) δ: 179.0, 160.2, 135.9, 134.4, 133.9, 132.0, 130.2, 128.1, 127.7, 124.9. ^{77}Se-NMR (76 MHz, $CDCl_3$) δ: 419.

N-(phenylcarbamoselenoyl)-2-furancarboxamide **(6a)**. White solid; yield: 39%. ^{1}H-NMR (400 MHz, $CDCl_3$) δ: 12.73 (1H, s, CONH), 11.43 (1H, s, Ph-NH), 8.10 (1H, d, *J* = 1.3 Hz, R-H), 7.90 (1H, d,

J = 3.6 Hz, R-H), 7.65 (2H, d, *J* = 7.8 Hz, R′-H), 7.44 (2H, t, *J* = 7.8 Hz, R′-H), 7.33 (1H, t, *J* = 7.8 Hz, R′-H), 6.78 (1H, dd, *J* = 3.6, 1.3 Hz, R-H). ^{13}C-NMR (100 MHz, $CDCl_3$) δ: 180.4, 157.7, 149.1, 144.9, 139.4, 129.2, 127.4, 125.7, 119.5, 113.3. ^{77}Se-NMR (76 MHz, $CDCl_3$) δ: 417.

N-[(4-methylphenyl)carbamoselenoyl]-2-furancarboxamide **(6b)**. Yellow solid; yield: 66%. ^{1}H-NMR (400 MHz, $CDCl_3$) δ: 12.67 (1H, s, CONH), 11.38 (1H, s, Ph-NH), 8.09 (1H, d, *J* = 1.4 Hz, R-H), 7.88 (1H, d, *J* = 3.6 Hz, R-H), 7.51 (2H, d, *J* = 8.3 Hz, R′-H), 7.23 (2H, d, *J* = 8.3 Hz, R′-H), 6.78 (1H, dd, *J* = 3.6, 1.4 Hz, R-H), 2.32 (3H, s, CH_3). ^{13}C-NMR (100 MHz, $CDCl_3$) δ: 180.2, 157.7, 149.1, 144.9, 136.9, 129.6, 125.5, 119.5, 113.3, 21.1. ^{77}Se-NMR (76 MHz, $CDCl_3$) δ: 413.

N-((4-methoxyphenyl)carbamoselenoyl)-2-furancarboxamide **(6c)**. Yellow solid; yield: 39%. ^{1}H-NMR (400 MHz, $CDCl_3$) δ: 12.59 (1H, s, CONH), 11.37 (1H, s, Ph-NH), 8.09 (1H, s, R-H), 7.88 (1H, d, *J* = 3.6 Hz, R-H), 7.52 (2H, d, *J* = 8.9 Hz, R′-H), 6.98 (2H, d, *J* = 8.9 Hz, R′-H), 6.78 (1H, dd, *J* = 3.6, 1.6 Hz, R-H), 3.79 (3H, s, OCH_3). ^{13}C-NMR (100 MHz, $CDCl_3$) δ: 180.4, 158.4, 157.7, 149.0, 145.0, 132.3, 127.1, 119.4, 114.3, 113.3, 55.8. ^{77}Se-NMR (76 MHz, $CDCl_3$) δ: 406.

N-[(4-trifluoromethylphenyl)carbamoselenoyl]-2-furancarboxamide **(6d)**. White solid; yield: 42%. ^{1}H-NMR (400 MHz, $CDCl_3$) δ: 12.81 (1H, s, CONH), 11.61 (1H, s, Ph-NH), 8.11 (1H, d, *J* = 1.4 Hz, R-H), 7.92 (1H, m, R-H), 7.91 (2H, d, *J* = 8.5 Hz, R′-H), 7.80 (2H, d, *J* = 8.5 Hz, R′-H), 6.79 (1H, dd, *J* = 3.6, 1.4 Hz, R-H). ^{13}C-NMR (100 MHz, $CDCl_3$) δ: 181.1, 156.9, 149.2, 147.5, 146.6, 144.9, 143.1, 142.7, 126.4, 120.6, 119.7, 116.0, 113.3, 112.8. ^{77}Se-NMR (76 MHz, $CDCl_3$) δ: 437.

N-[(4-chlorophenyl)carbamoselenoyl]-2-furancarboxamide **(6e)**. White solid; yield: 39%. ^{1}H-NMR (400 MHz, $CDCl_3$) δ: 12.67 (1H, s, CONH), 11.52 (1H, s, Ph-NH), 8.10 (1H, s, R-H), 7.89 (1H, d, *J* = 3.5 Hz, R-H), 7.67 (2H, d, *J* = 8.6 Hz, R′-H), 7.49 (2H, d, *J* = 8.6 Hz, R′-H), 6.78 (1H, dd, *J* = 3.5, 1.6 Hz, R-H). ^{13}C-NMR (100 MHz, $CDCl_3$) δ: 180.9, 157.6, 149.1, 144.9, 138.5, 131.5, 129.1, 127.7, 119.6, 113.3. ^{77}Se-NMR (76 MHz, $CDCl_3$) δ: 424.

(1S,3s)-N-[(4-methoxyphenyl)carbamoselenoyl]adamantane-1-carboxamide **(7c)**. Pink solid; yield: 28%. ^{1}H-NMR (400 MHz, $CDCl_3$) δ: 12.83 (1H, s, CONH), 10.65 (1H, s, Ph-NH), 7.48 (2H, d, *J* = 8.9 Hz, R′-H), 6.96 (2H, d, *J* = 8.9 Hz, R′-H), 3.78 (3H, s, OCH_3), 2.05-1.99 (3H, m, R-H), 1.98-1.93 (6H, m, R-H), 1.79-1.64 (6H, m, R-H). ^{13}C-NMR (100 MHz, $CDCl_3$) δ: 181.0, 179.9, 158.3, 132.3, 127.1, 114.2, 55.8, 42.1, 37.3, 36.0, 27.9. ^{77}Se-NMR (76 MHz, $CDCl_3$) δ: 381.

(1S,3s)-N-[(4-trifluoromethylphenyl)carbamoselenoyl]adamantane-1-carboxamide **(7d)**. Pink solid; yield: 31%. ^{1}H-NMR (400 MHz, $CDCl_3$) δ: 13.08 (1H, s, CONH), 10.91 (1H, s, Ph-NH), 7.87 (2H, d, *J* = 8.4 Hz, R′-H), 7.77 (2H, d, *J* = 8.4 Hz, R′-H), 2.05-1.99 (3H, m, R-H), 1.99-1.95 (6H, m, R-H), 1.76-1.65 (6H, m, R-H). ^{13}C-NMR (100 MHz, $CDCl_3$) δ: 181.7, 179.9, 143.1, 127.5, 127.2, 126.4, 126.2, 125.9, 123.2, 42.1, 37.2, 36.0, 27.9. ^{77}Se-NMR (76 MHz, $CDCl_3$) δ: 410.

(1S,3s)-N-((4-chlorophenyl)carbamoselenoyl)adamantane-1-carboxamide **(7e)**. Yellow solid; yield: 37%. ^{1}H-NMR (400 MHz, $CDCl_3$) δ: 12.91 (1H, s, CONH), 10.82 (1H, s, Ph-NH), 7.62 (2H, d, *J* = 8.7 Hz, R′-H), 7.46 (2H, d, *J* = 8.7 Hz, R′-H), 2.05-1.99 (3H, m, R-H), 1.98-1.94 (6H, m, R-H), 1.79-1.65 (6H, m, R-H). ^{13}C-NMR (100 MHz, $CDCl_3$) δ: 181.5, 179.9, 138.4, 131.4, 129.0, 127.8, 42.1, 37.2, 36.0, 27.9. ^{77}Se-NMR (76 MHz, $CDCl_3$) δ: 397.

N-(phenylcarbamoselenoyl)cinnamamide **(8a)**. Yellow solid; yield: 45%. ^{1}H-NMR (400 MHz, $CDCl_3$) δ: 12.97 (1H, s, CONH), 9.39 (1H, s, Ph-NH), 7.78 (1H, d, *J* = 15.5 Hz, C=C-H), 7.61 (2H, d, *J* = 7.7 Hz, R′-H), 7.49 (2H, d, *J* = 7.7 Hz, R′-H), 7.41-7.32 (5H, m, R-H), 7.26 (1H, t, *J* = 7.7 Hz, R′-H), 6.51 (1H, d, *J* = 15.5 Hz, C=C-H).^{13}C-NMR (100 MHz, $CDCl_3$) δ: 179.0, 164.8, 146.2, 137.4, 132.6, 130.3, 128.1, 128.0, 127.6, 126.5, 123.8, 117.0. ^{77}Se-NMR (76 MHz, $CDCl_3$) δ: 392.

N-[(4-methylphenyl)carbamoselenoyl]cinnamamide **(8b)**. Brown solid; yield: 42%. ^{1}H-NMR (400 MHz, $CDCl_3$) δ: 12.84 (1H, s, CONH), 9.24 (1H, s, Ph-NH), 7.78 (1H, d, *J* = 15.5 Hz, C=C-H), 7.51-7.48 (2H,

m, R-H), 7.47 (2H, d, *J* = 8.3 Hz, R′-H), 7.40-7.32 (3H, m, R-H), 7.15 (2H, d, *J* = 8.3 Hz, R′-H), 6.46 (1H, d, *J* = 15.5 Hz, C=C-H), 2.30 (3H, s, CH_3).^{13}C-NMR (100 MHz, $CDCl_3$) δ: 178.9, 164.6, 146.1, 136.5, 134.9, 132.6, 130.3, 128.6, 128.1, 127.6, 123.7, 117.0, 20.2. ^{77}Se-NMR (76 MHz, $CDCl_3$) δ: 386.

N-[(4-methoxyphenyl)carbamoselenoyl]cinnamamide **(8c)**. Yellow solid; yield: 48%. ^{1}H-NMR (400 MHz, $CDCl_3$) δ: 12.80 (1H, s, CONH), 9.47 (1H, s, Ph-NH), 7.77 (1H, d, *J* = 15.5 Hz, C=C-H), 7.50-7.47 (2H, m, R-H), 7.47 (2H, d, *J* = 8.5 Hz, R′-H), 7.39-7.31 (3H, m, R-H), 6.86 (2H, d, *J* = 8.5 Hz, R′-H), 6.52 (1H, d, *J* = 15.5 Hz, C=C-H), 3.76 (3H, s, OCH_3).^{13}C-NMR (100 MHz, $CDCl_3$) δ: 179.1, 164.8, 157.7, 146.0, 132.6, 130.4, 130.3, 128.1, 127.6, 125.3, 117.0, 113.1, 54.4. ^{77}Se-NMR (76 MHz, $CDCl_3$) δ: 376.

N-[(4-trifluoromethylphenyl)carbamoselenoyl]cinnamamide **(8d)**. Yellow solid; yield: 49%. ^{1}H-NMR (400 MHz, $CDCl_3$) δ: 13.20 (1H, s, CONH), 9.37 (1H, s, Ph-NH), 7.83 (2H, d, *J* = 8.3 Hz, R′-H), 7.79 (1H, m, C=C-H), 7.60 (2H, d, *J* = 8.3 Hz, R′-H), 7.50 (2H,d, *J* = 6.8 Hz, R-H), 7.41-7.31 (3H, m, R-H), 6.48 (1H, d, *J* = 15.5 Hz, C=C-H).^{13}C-NMR (100 MHz, $CDCl_3$) δ: 179.3, 164.9, 146.7, 140.3, 132.5, 130.5, 128.2, 127.6, 125.1, 123.5, 116.6. ^{77}Se-NMR (76 MHz, $CDCl_3$) δ: 421.

N-((4-chlorophenyl)carbamoselenoyl)cinnamamide **(8e)**. Yellow solid; yield: 46%. ^{1}H-NMR (400 MHz, $CDCl_3$) δ: 12.95 (1H, s, CONH), 9.10 (1H, s, Ph-NH), 7.79 (1H, d, *J* = 15.5 Hz, C=C-H), 7.59 (2H, d, *J* = 8.7 Hz, R′-H), 7.52-7.48 (2H, m, R-H), 7.42-7.35 (3H, m, R-H), 7.32 (2H, d, *J* = 8.7 Hz, R′-H), 6.41 (1H, d, *J* = 15.5 Hz, C=C-H).^{13}C-NMR (100 MHz, $CDCl_3$) δ: 180.3, 165.6, 147.5, 136.9, 133.5, 132.9, 131.5, 129.2, 128.6, 125.9, 117.7. ^{77}Se-NMR (76 MHz, $CDCl_3$) δ: 408.

N-(phenylcarbamoselenoyl)benzo[b]thiophene-2-carboxamide **(9a)**. Yellow solid; yield: 44%. ^{1}H-NMR (400 MHz, $CDCl_3$) δ: 12.72 (1H, s, CONH), 9.40 (1H, s, Ph-NH), 7.97 (1H, s, CH), 7.85 (2H, t, *J* = 8.7 Hz, R-H), 7.63 (2H, d, *J* = 7.8 Hz, R′-H), 7.46 (1H, t, *J* = 7.8 Hz, R′-H), 7.41-7.27 (4H, m, R-H+R′-H). ^{13}C-NMR (100 MHz, $CDCl_3$) δ: 179.5, 161.8, 142.4, 138.7, 138.4, 135.1, 129.1, 128.4, 128.0, 127.7, 126.1, 125.7, 124.7, 122.9. ^{77}Se-NMR (76 MHz, $CDCl_3$) δ: 415.

N-[(4-methylphenyl)carbamoselenoyl]benzo[b]thiophene-2-carboxamide **(9b)**. Yellow solid; yield: 62%. ^{1}H-NMR (400 MHz, $CDCl_3$) δ: 12.72 (1H, s, CONH), 9.40 (1H, s, Ph-NH), 7.96 (1H, s, CH), 7.84 (2H, t, *J* = 8.4 Hz, R-H), 7.48 (2H, d, *J* = 7.7 Hz, R′-H), 7.46-7.43 (1H, m, R-H), 7.39 (1H, t, *J* = 7.4 Hz, R-H), 7.16 (2H, d, *J* = 7.7 Hz, R′-H), 2.30 (3H, s, CH_3). ^{13}C-NMR (100 MHz, $CDCl_3$) δ: 179.5, 161.7, 142.4, 138.7, 137.7, 135.9, 135.2, 129.7, 128.3, 127.9, 126.0, 125.6, 124.6, 122.9, 21.2. ^{77}Se-NMR (76 MHz, $CDCl_3$) δ: 408.

N-[(4-methoxyphenyl)carbamoselenoyl]benzo[b]thiophene-2-carboxamide **(9c)**. Yellow solid; yield: 37%. ^{1}H-NMR (400 MHz, $CDCl_3$) δ: 12.56 (1H, s, CONH), 9.44 (1H, s, Ph-NH), 7.95 (1H, s, CH), 7.83 (2H, t, *J* = 7.8 Hz, R-H), 7.48 (2H, d, *J* = 8.4 Hz, R′-H), 7.45-7.36 (2H, m, R-H), 6.86 (2H, d, *J* = 8.4 Hz, R′-H), 3.75 (3H, s, OCH_3). ^{13}C-NMR (100 MHz, $CDCl_3$) δ: 179.6, 161.8, 158.8, 142.4, 138.7, 135.2, 131.4, 128.4, 127.9, 126.2, 126.0, 125.6, 122.9, 114.2, 55.5. ^{77}Se-NMR (76 MHz, $CDCl_3$) δ: 401.

N-[(4-trifluoromethylphenyl)carbamoselenoyl]benzo[b]thiophene-2-carboxamide **(9d)**. Yellow solid; yield: 51%. ^{1}H-NMR (400 MHz, $CDCl_3$) δ: 12.95 (1H, s, CONH), 9.45 (1H, s, Ph-NH), 7.98 (1H, s, CH), 7.86-7.83 (4H, m, R-H+R′-H), 7.61 (2H, d, *J* = 8.5 Hz, R′-H), 7.50-7.37 (2H, m, R-H). ^{13}C-NMR (100 MHz, $CDCl_3$) δ: 179.8, 161.9, 142.5, 141.3, 138.6, 134.7, 128.6, 128.1, 126.3, 126.2, 126.1, 125.7, 124.4, 122.9. ^{77}Se-NMR (76 MHz, $CDCl_3$) δ: 448.

N-((4-chlorophenyl)carbamoselenoyl)benzo[b]thiophene-2-carboxamide **(9e)**. Yellow solid; yield: 43%. ^{1}H-NMR (400 MHz, $CDCl_3$) δ: 12.73 (1H, s, CONH), 9.40 (1H, s, Ph-NH), 7.97 (1H, s, CH), 7.90-7.82 (2H, m, R-H), 7.61 (2H, d, *J* = 8.7 Hz, R′-H), 7.47 (1H, t, *J* = 7.0 Hz, R-H), 7.41 (1H, t, *J* = 7.0 Hz, R-H), 7.33 (2H, d, *J* = 8.7 Hz, R′-H). ^{13}C-NMR (100 MHz, $CDCl_3$) δ: 179.8, 161.8, 142.5, 138.7, 136.9, 134.9, 133.1, 129.2, 128.5, 128.1, 126.1, 125.9, 125.7, 122.9. ^{77}Se-NMR (76 MHz, $CDCl_3$) δ: 427.

N-(phenylcarbamoselenoyl)benzo[d][1,3]dioxole-5-carboxamide **(10a)**. Orange solid; yield: 66%. ^{1}H-NMR (400 MHz, $CDCl_3$) δ: 12.95 (1H, s, CONH), 9.38 (1H, s, Ph-NH), 7.62 (2H, d, *J* = 7.7 Hz, R′-H), 7.41 (1H, dd, *J* = 8.2, 1.9 Hz, R-H), 7.37 (2H, t, *J* = 7.7Hz, R′-H), 7.31 (1H, d, *J* = 1.9 Hz, R-H), 7.27 (1H, t, *J* = 7.7 Hz, R′-H), 6.85 (1H, d, *J* = 8.2 Hz, R-H), 6.03 (2H, s, CH_2). ^{13}C-NMR (100 MHz, $CDCl_3$) δ: 179.1, 165.1, 151.6, 147.7, 137.4, 128.0, 124.1, 123.7, 122.3, 107.6, 106.9, 101.4. ^{77}Se-NMR (76 MHz, $CDCl_3$) δ: 394.

N-[(4-methylphenyl)carbamoselenoyl]benzo[d][1,3]dioxole-5-carboxamide **(10b)**. Orange solid; yield: 68%. ^{1}H-NMR (400 MHz, $CDCl_3$) δ: 12.82 (1H, s, CONH), 9.29 (1H, s, Ph-NH), 7.47 (2H, d, *J* = 8.3 Hz, R′-H), 7.38 (1H, dd, *J* = 8.2, 1.8 Hz, R-H), 7.28 (1H, d, *J* = 1.8 Hz, R-H), 7.15 (2H, d, *J* = 8.3 Hz, R′-H), 6.83 (1H, d, *J* = 8.2 Hz, R-H), 6.01 (2H, s, CH_2), 2.29 (3H, s, CH_3). ^{13}C-NMR (100 MHz, $CDCl_3$) δ: 180.0, 166.1, 152.6, 148.7, 137.6, 136.0, 129.6, 125.2, 124.7, 123.2, 108.6, 107.9, 102.4, 21.2. ^{77}Se-NMR (76 MHz, $CDCl_3$) δ: 390.

N-[(4-methoxyphenyl)carbamoselenoyl]benzo[d][1,3]dioxole-5-carboxamide **(10c)**. Orange solid; yield: 62%. ^{1}H-NMR (400 MHz, $CDCl_3$) δ: 12.76 (1H, s, CONH), 9.31 (1H, s, Ph-NH), 7.48 (2H, d, *J* = 8.9 Hz, R′-H), 7.38 (1H, dd, *J* = 8.2, 1.6 Hz, R-H), 7.29 (1H, d, *J* = 1.6 Hz, R-H), 6.87 (2H, d, *J* = 8.9 Hz, R′-H), 6.84 (1H, d, *J* = 8.2 Hz, R-H), 6.03 (2H, s, CH_2), 3.76 (3H, s, OCH_3). ^{13}C-NMR (100 MHz, $CDCl_3$) δ: 180.2, 166.1, 158.7, 152.6, 148.7, 131.5, 126.3, 125.2, 123.2, 114.2, 108.6, 107.9, 102.4, 55.5. ^{77}Se-NMR (76 MHz, $CDCl_3$) δ: 383.

N-[(4-trifluoromethylphenyl)carbamoselenoyl]benzo[d][1,3]dioxole-5-carboxamide **(10d)**. Orange solid; yield: 56%. ^{1}H-NMR (400 MHz, $CDCl_3$) δ: 13.08 (1H, s, CONH), 11.72 (1H, s, Ph-NH), 7.93 (2H, d, *J* = 8.4 Hz, R′-H), 7.79 (2H, d, *J* = 8.4 Hz, R′-H), 7.66 (1H, dd, *J* = 8.2, 1.5 Hz, R-H), 7.55 (1H, d, *J* = 1.5 Hz, R-H), 7.07 (1H, d, *J* = 8.2 Hz, R-H), 6.17 (2H, s, CH_2). ^{13}C-NMR (100 MHz, $CDCl_3$) δ: 181.5, 152.2, 147.9, 143.2, 126.2, 125.7, 125.6, 120.5, 109.0, 108.5, 102.7, 102.4. ^{77}Se-NMR (76 MHz, $CDCl_3$) δ: 432.

N-((4-chlorophenyl)carbamoselenoyl)benzo[d][1,3]dioxole-5-carboxamide **(10e)**. Orange solid; yield: 67%. ^{1}H-NMR (400 MHz, $CDCl_3$) δ: 12.95 (1H, s, CONH), 9.33 (1H, s, Ph-NH), 7.59 (2H, d, *J* = 8.4 Hz, R′-H), 7.38 (1H, dd, *J* = 8.2, 1.9 Hz, R-H), 7.31 (2H, d, *J* = 8.4 Hz, R′-H), 7.28 (1H, d, *J* = 1.9 Hz, R-H), 6.84 (1H, d, *J* = 8.2 Hz, R-H), 6.03 (2H, s, CH_2). ^{13}C-NMR (100 MHz, $CDCl_3$) δ: 179.3, 165.1, 151.7, 147.7, 135.9, 131.9, 128.1, 124.9, 123.9, 122.2, 107.6, 106.8, 101.4. ^{77}Se-NMR (76 MHz, $CDCl_3$) δ: 408.

4. NMR SPECTRA (^{1}H, ^{13}C AND ^{77}Se) OF FINAL PRODUCTS

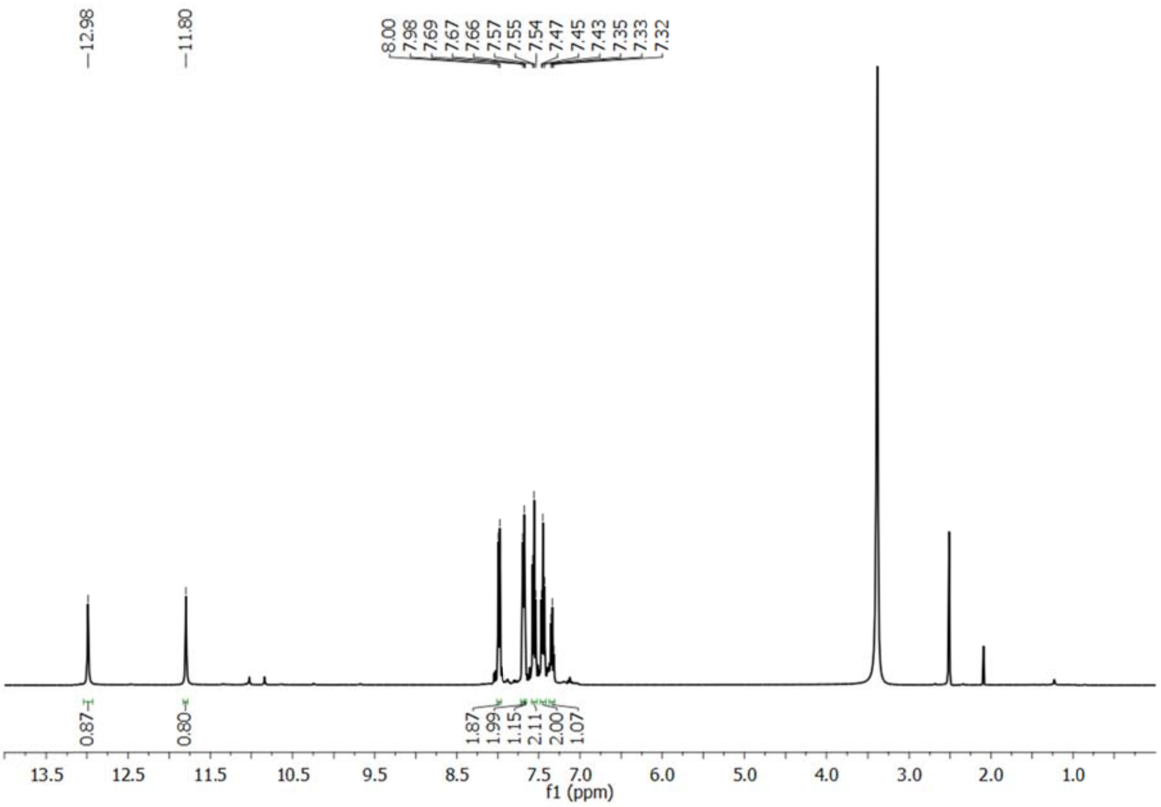

Figure S28. ^{1}H-NMR of compound **1a**.

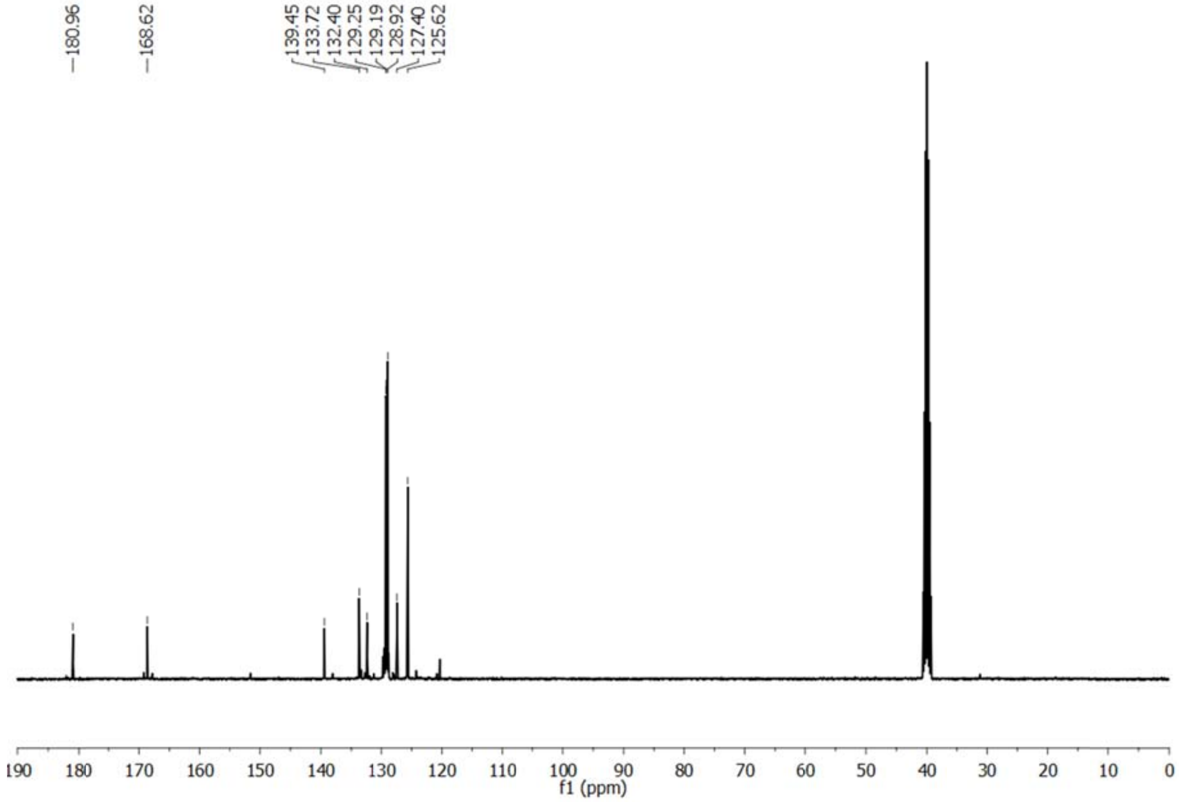

Figure S29. ^{13}C-NMR of compound **1a.**

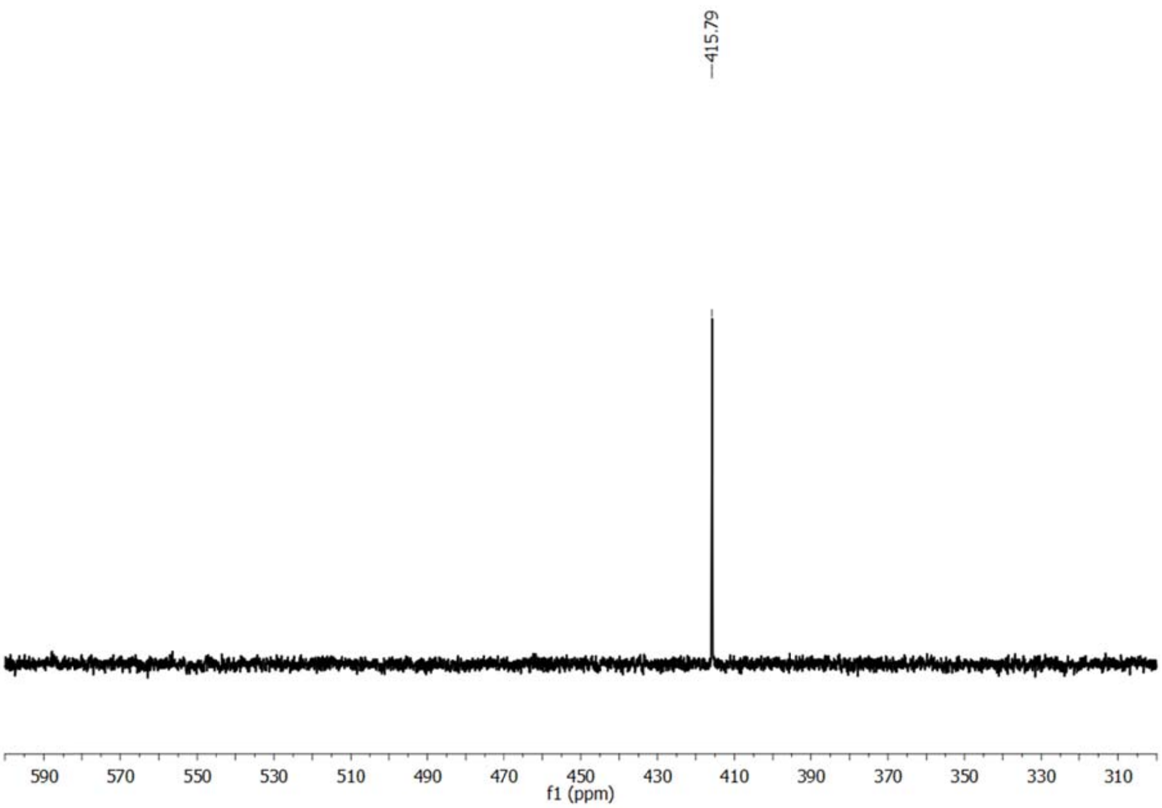

Figure S30. ^{77}Se-NMR of compound **1a**.

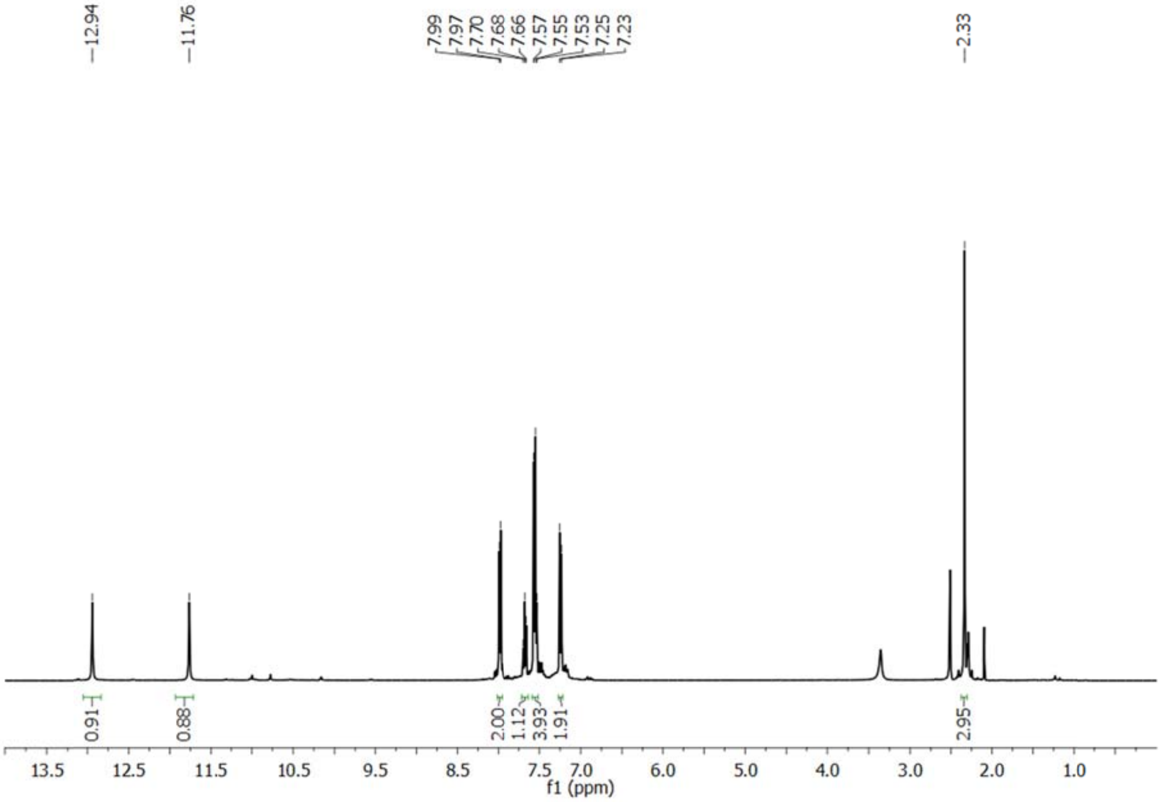

Figure S31. ^{1}H-NMR of compound **1b**.

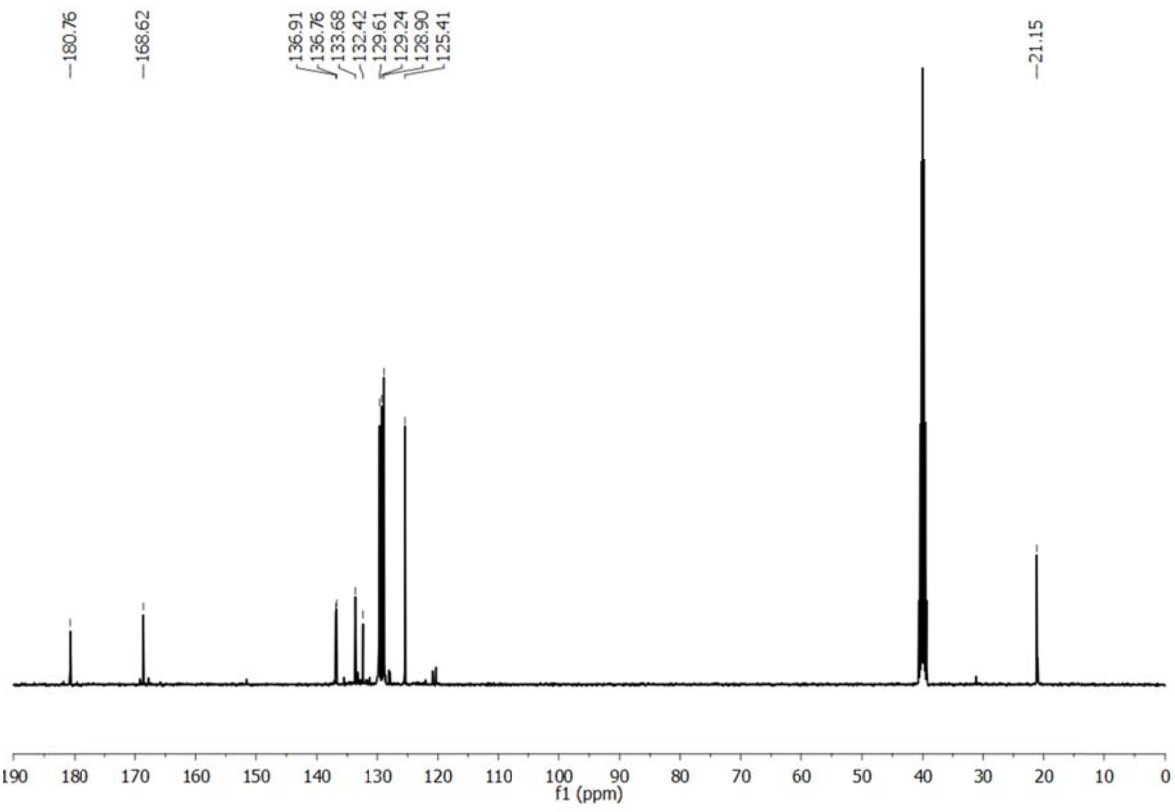

Figure S32. ^{13}C-NMR of compound **1b**.

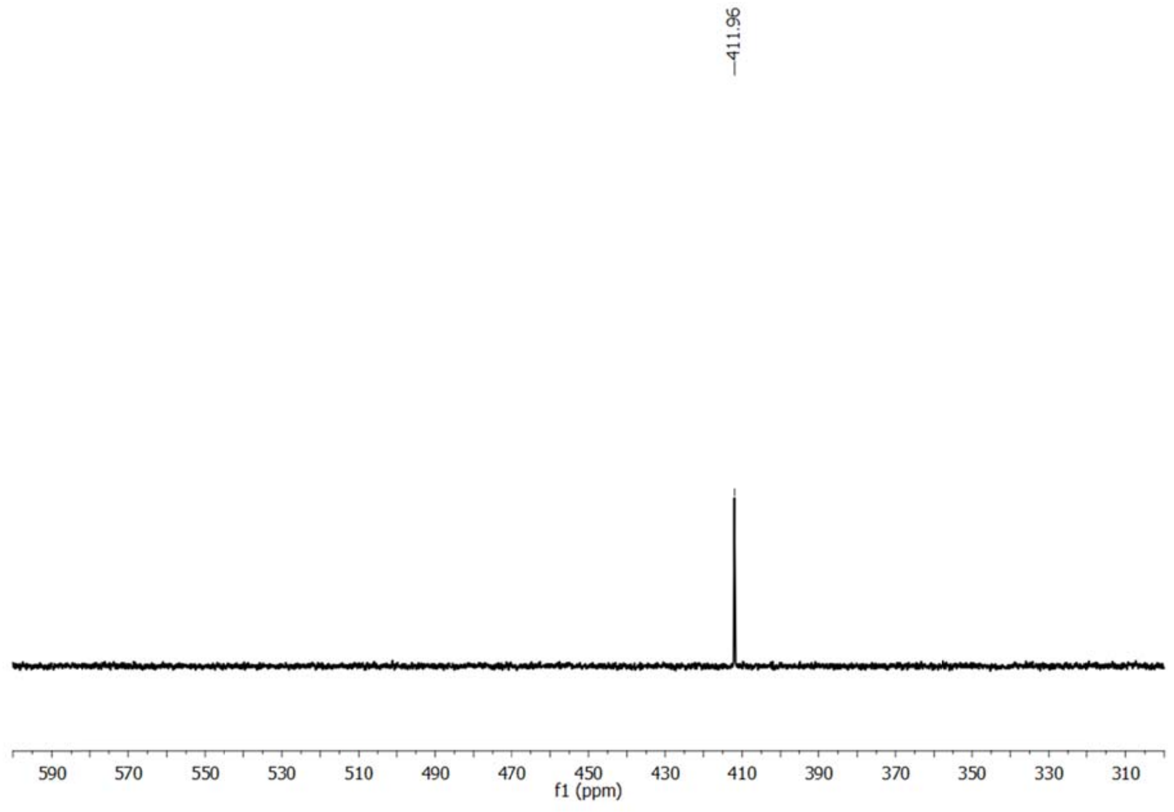

Figure S33. ^{77}Se-NMR of compound **1b**.

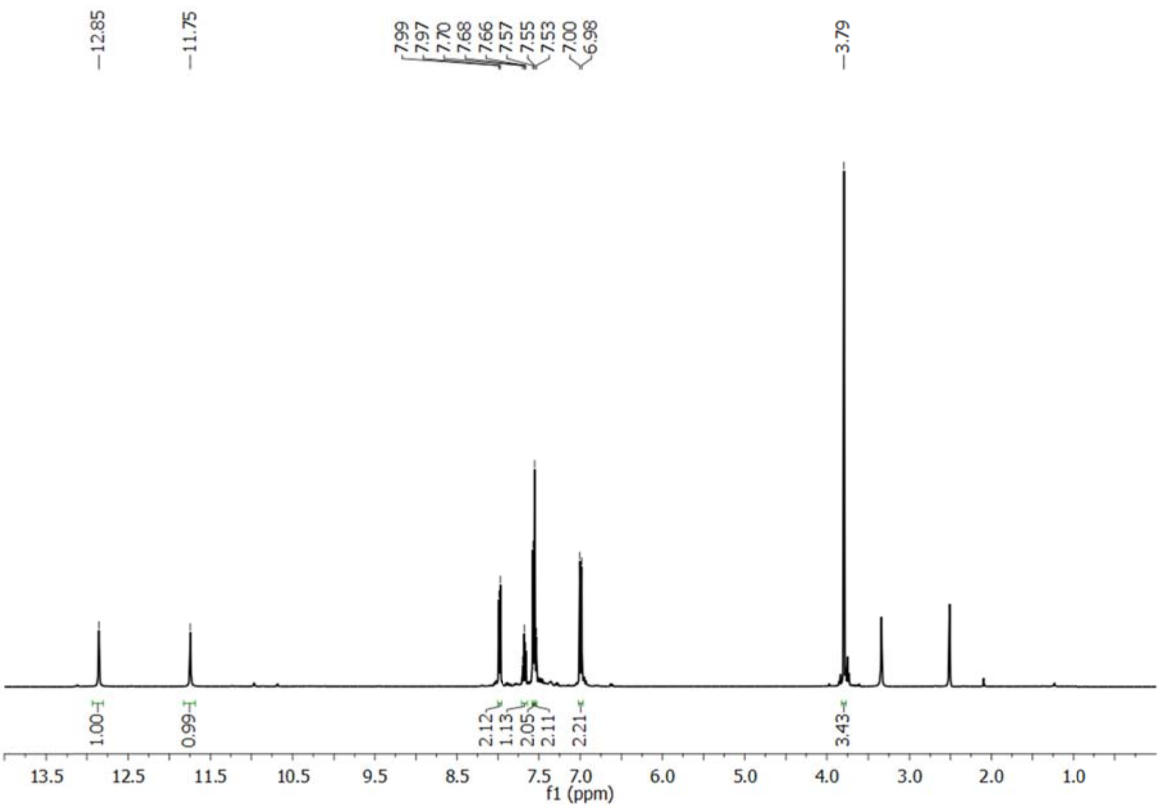

Figure S34. ^{1}H-NMR of compound **1c**.

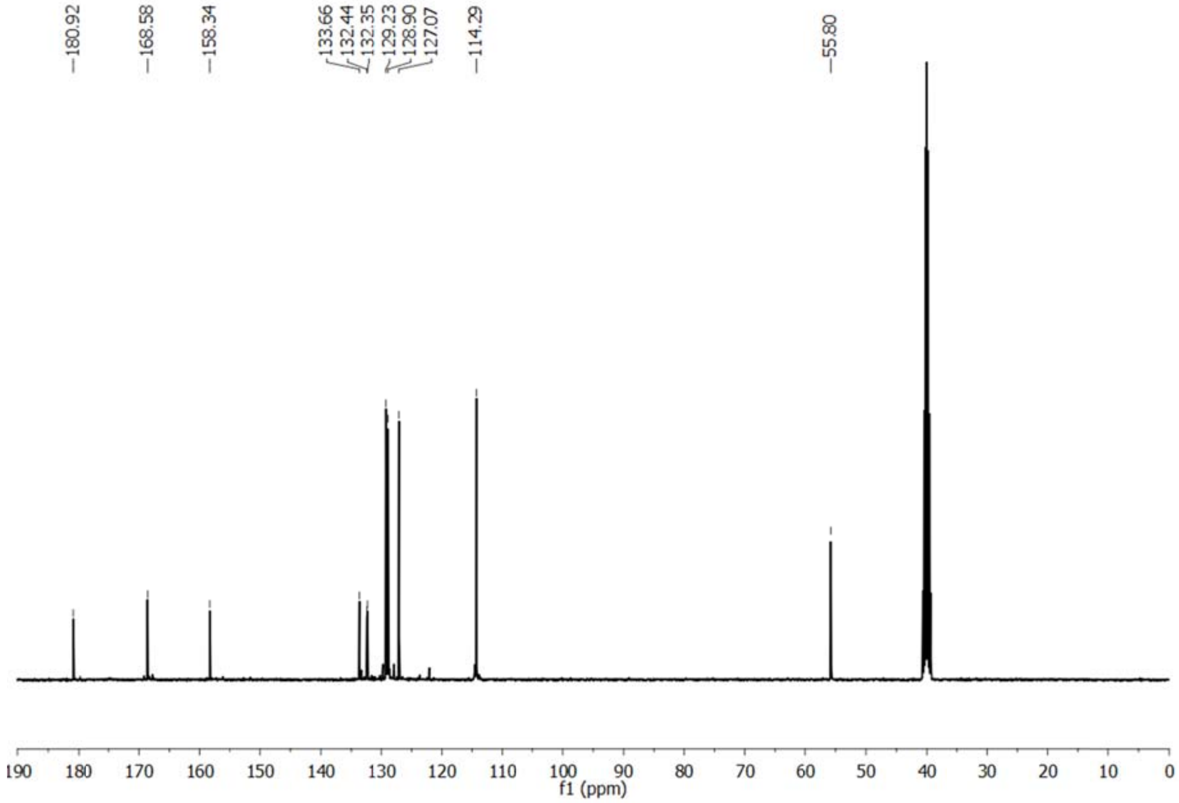

Figure S35. ^{13}C-NMR of compound **1c.**

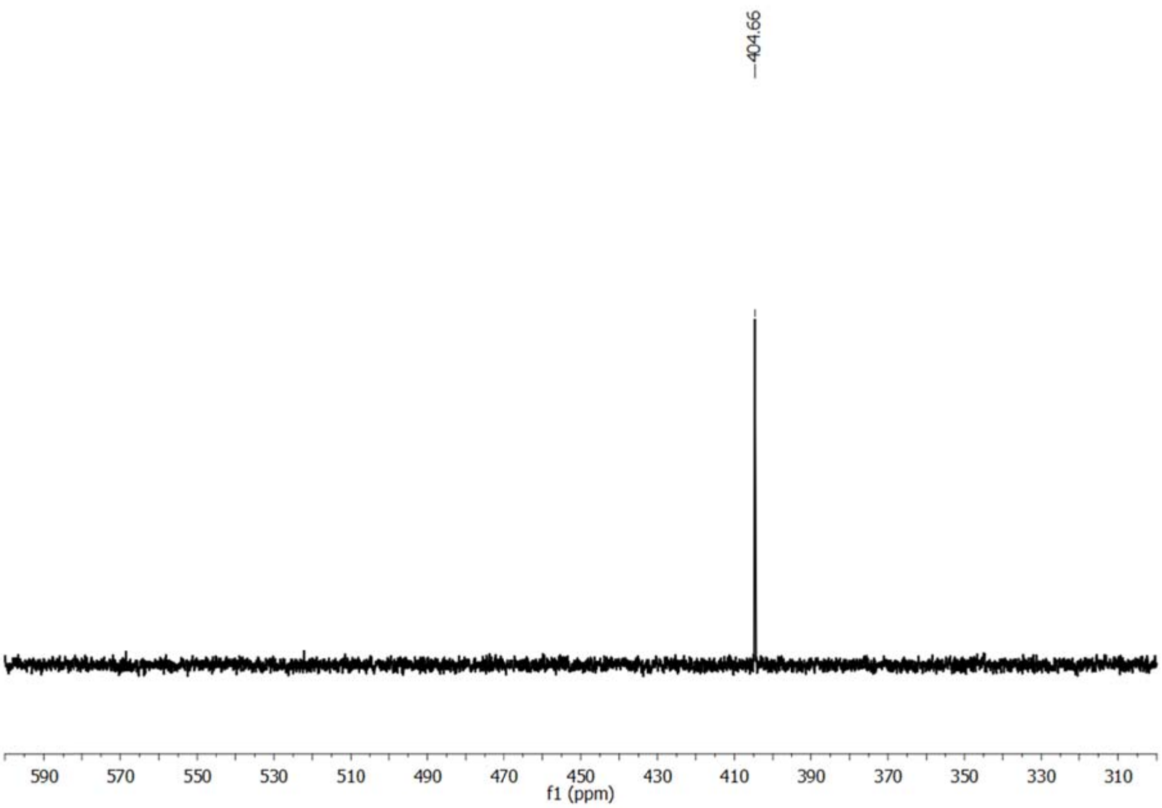

Figure S36. ^{77}Se-NMR of compound **1c**.

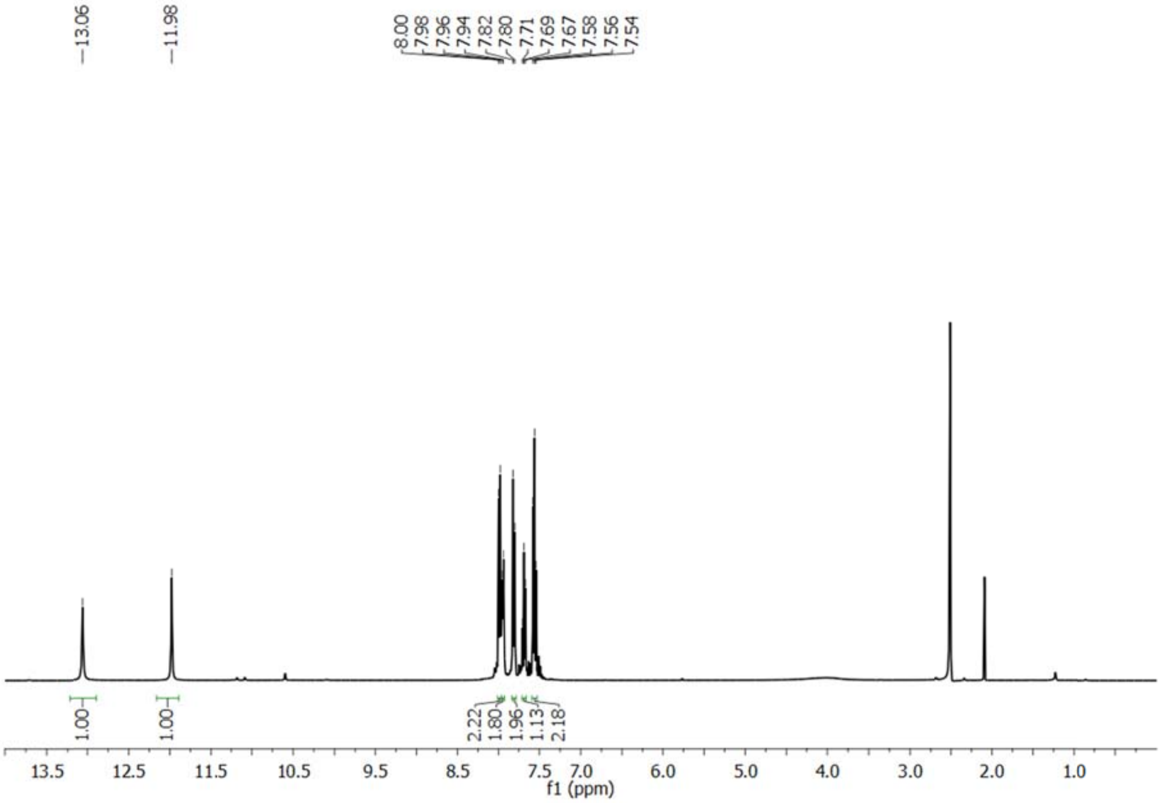

Figure S37. ^{1}H-NMR of compound **1d.**

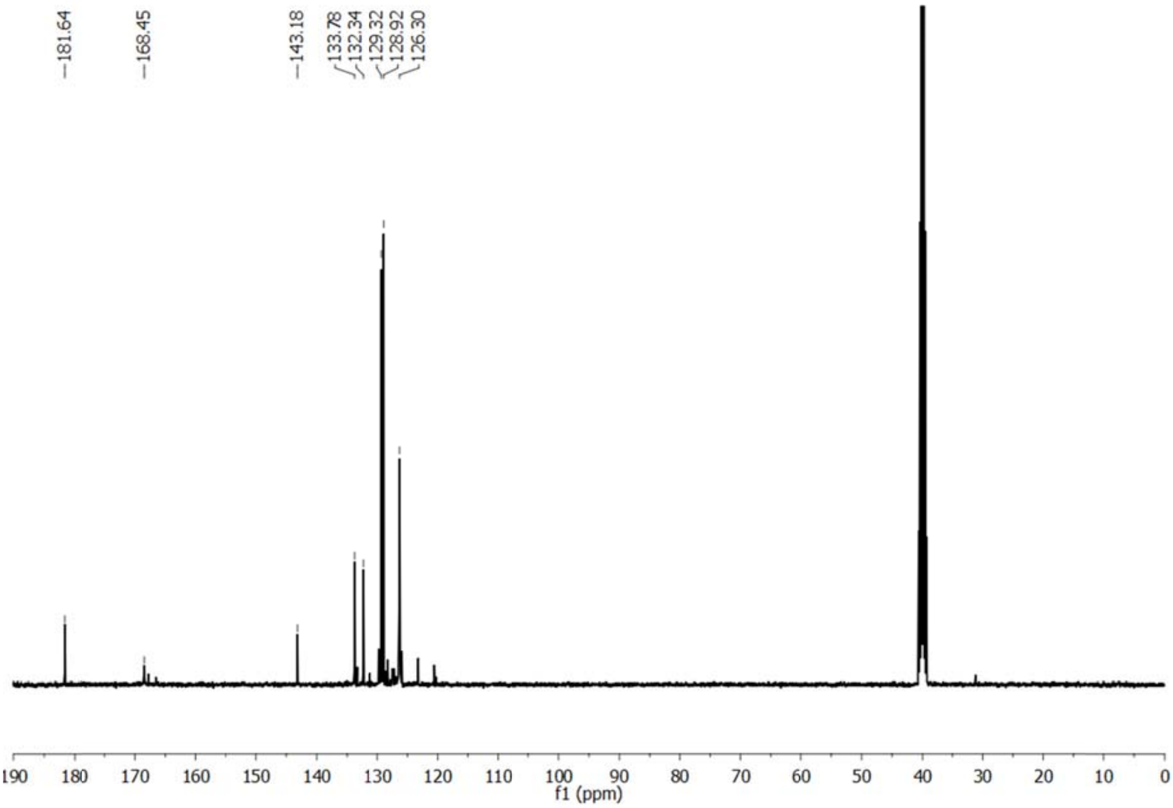

Figure S38. ^{13}C-NMR of compound **1d**.

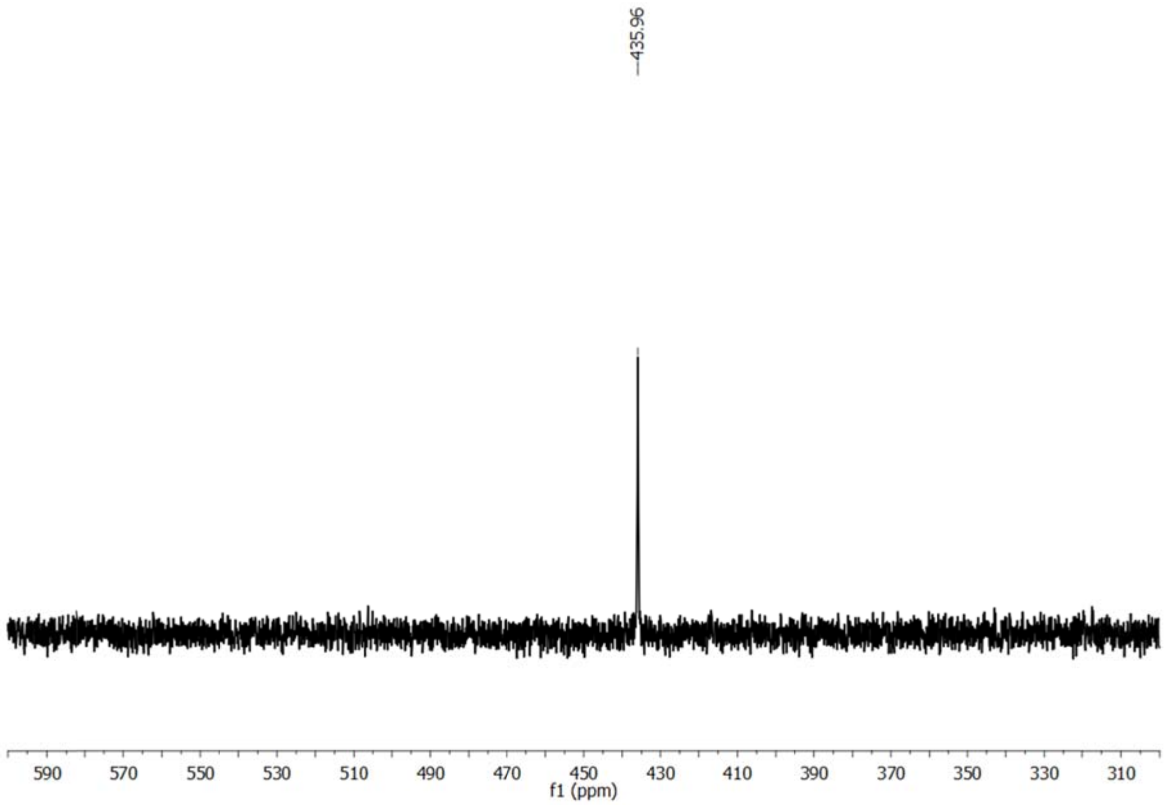

Figure S39. ^{77}Se-NMR of compound **1d**.

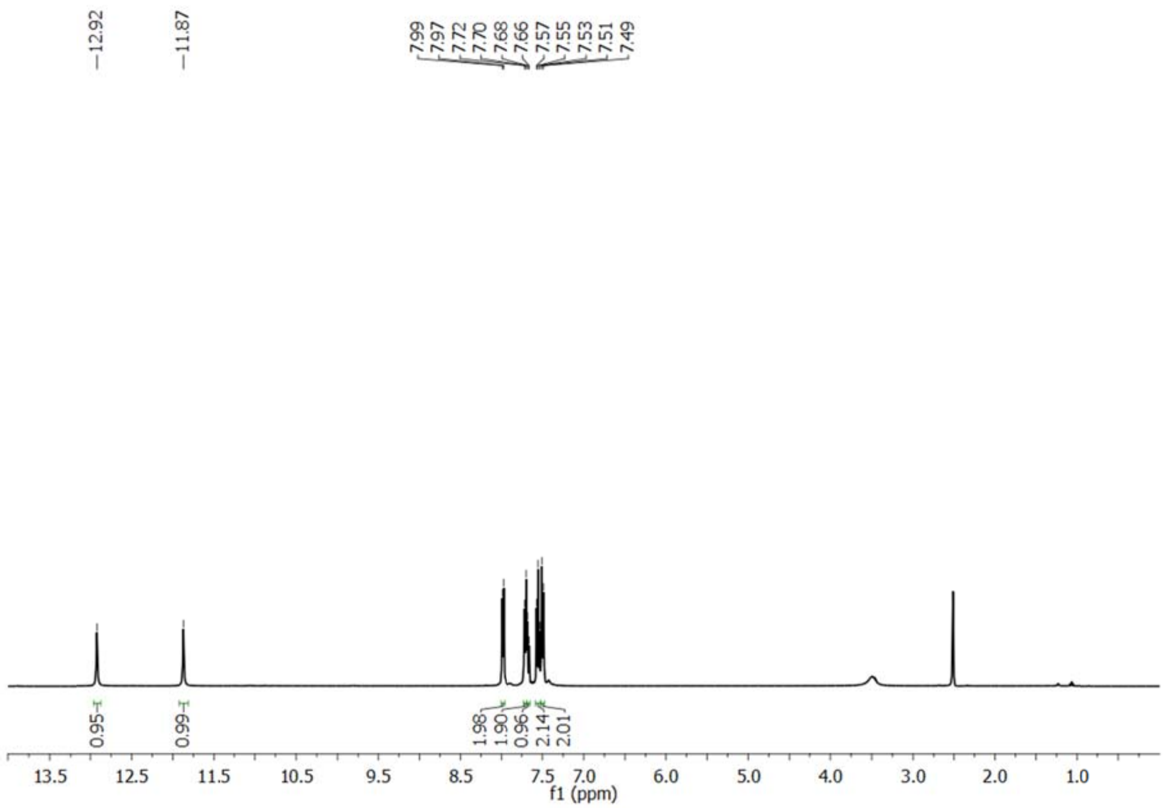

Figure S40. ^{1}H-NMR of compound **1e**.

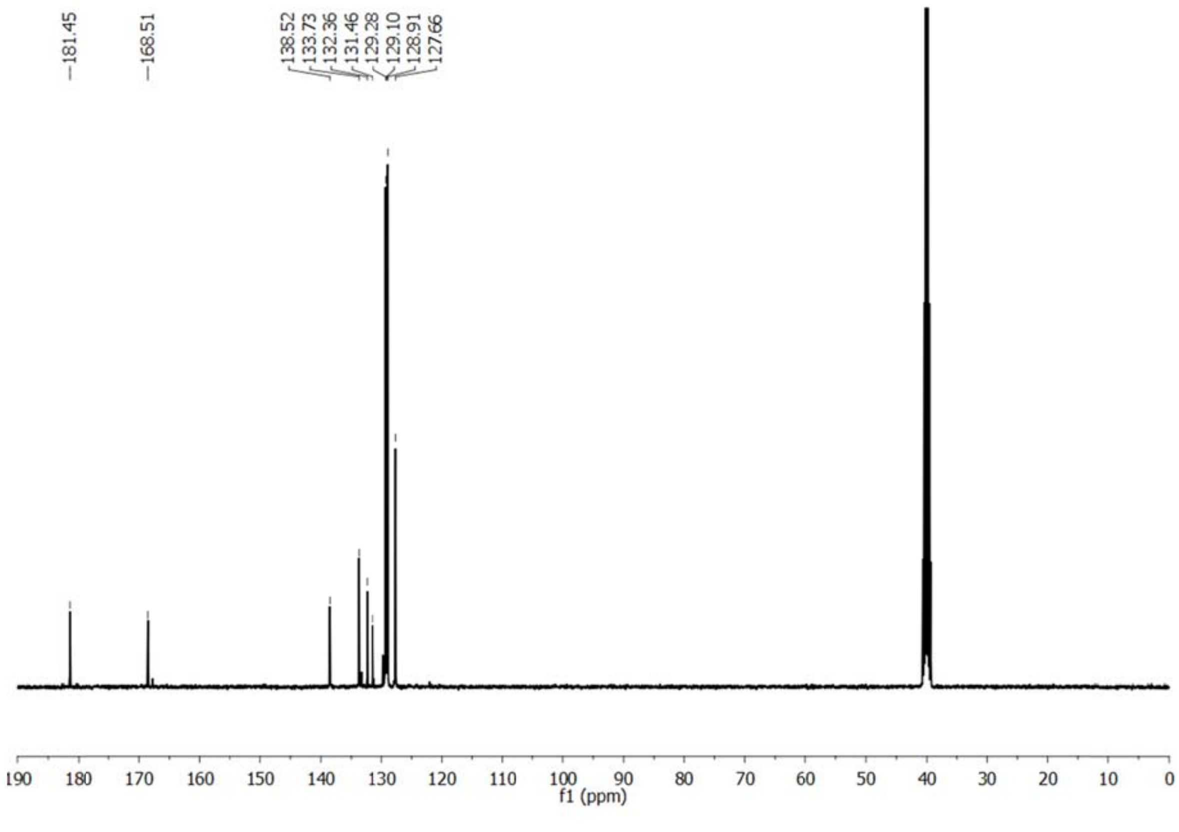

Figure S41. ^{13}C-NMR of compound **1e.**

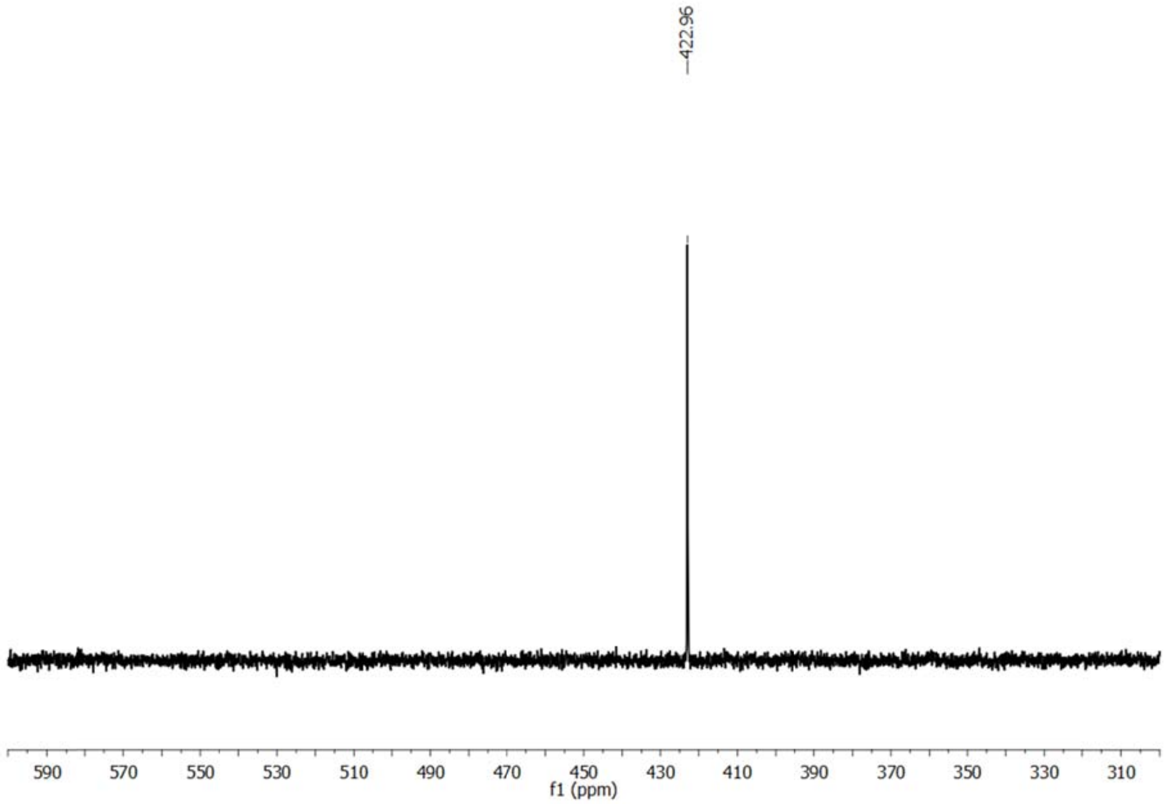

Figure S42. ^{77}Se-NMR of compound **1e**.

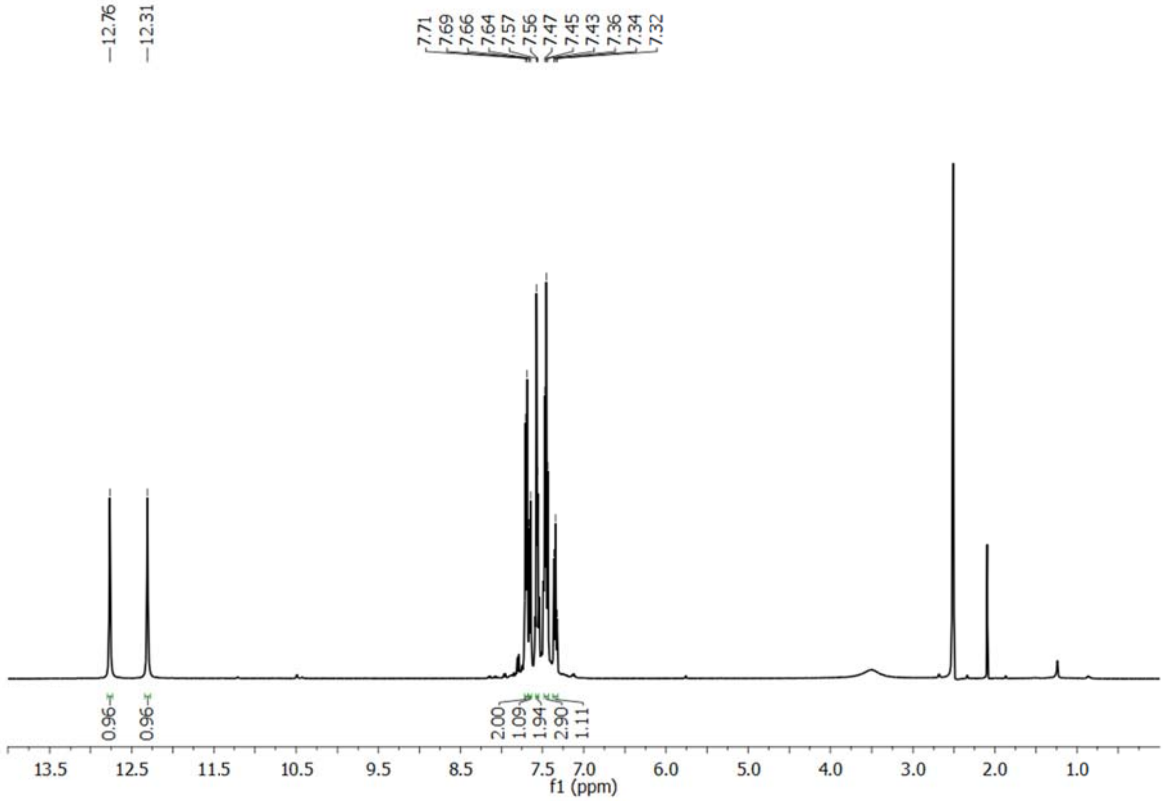

Figure S43. ^{1}H-NMR of compound **2a**.

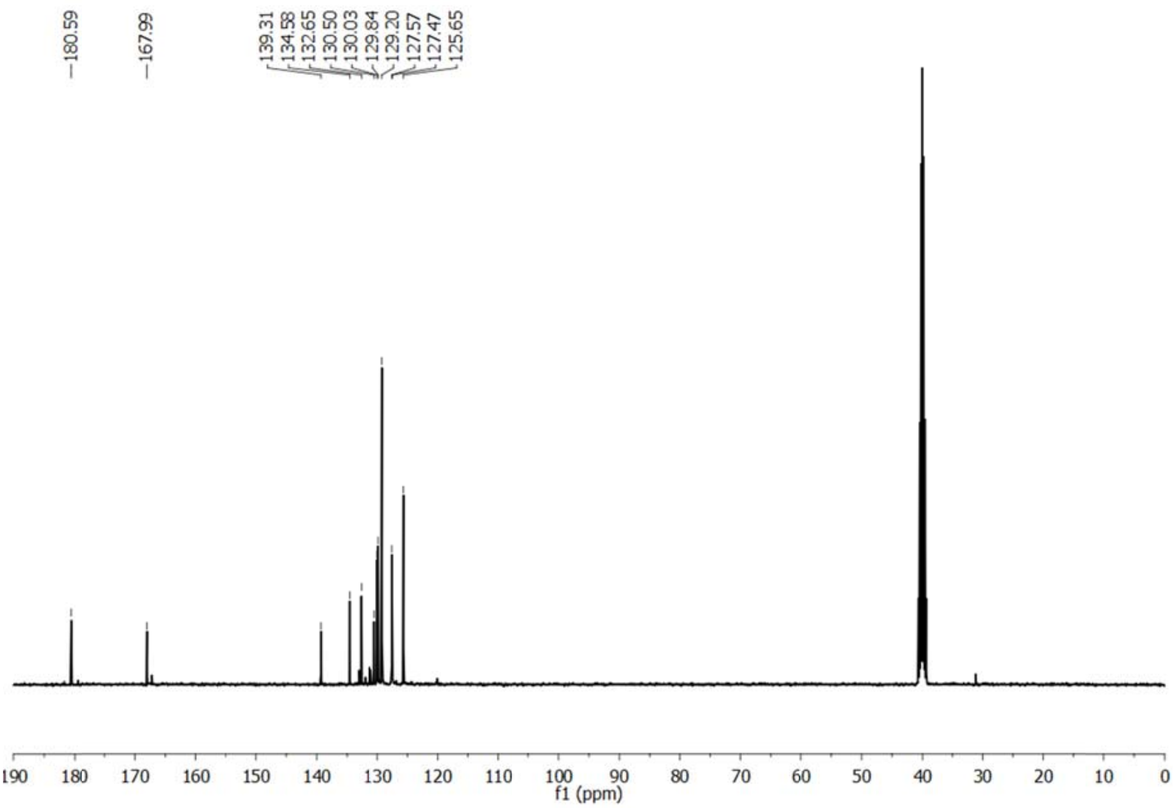

Figure S44. ^{13}C-NMR of compound **2a.**

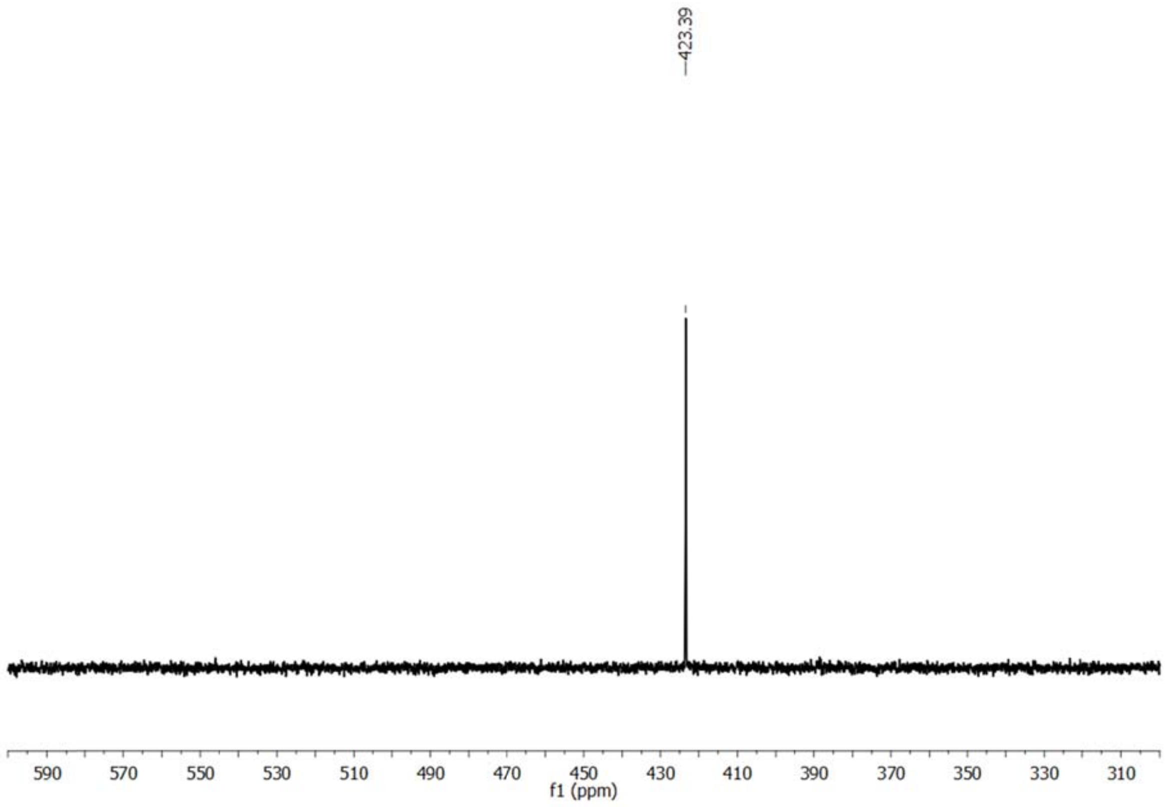

Figure S45. ^{77}Se-NMR of compound **2a**.

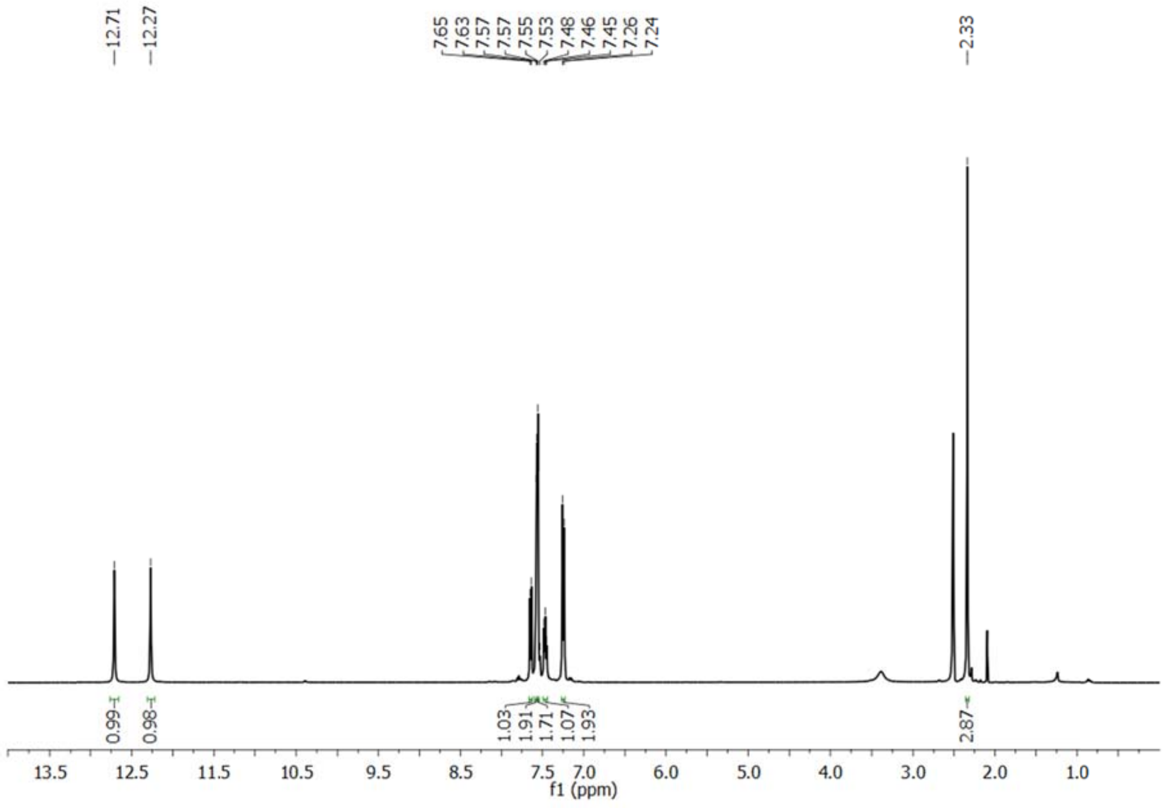

Figure S46. ¹H-NMR of compound **2b**.

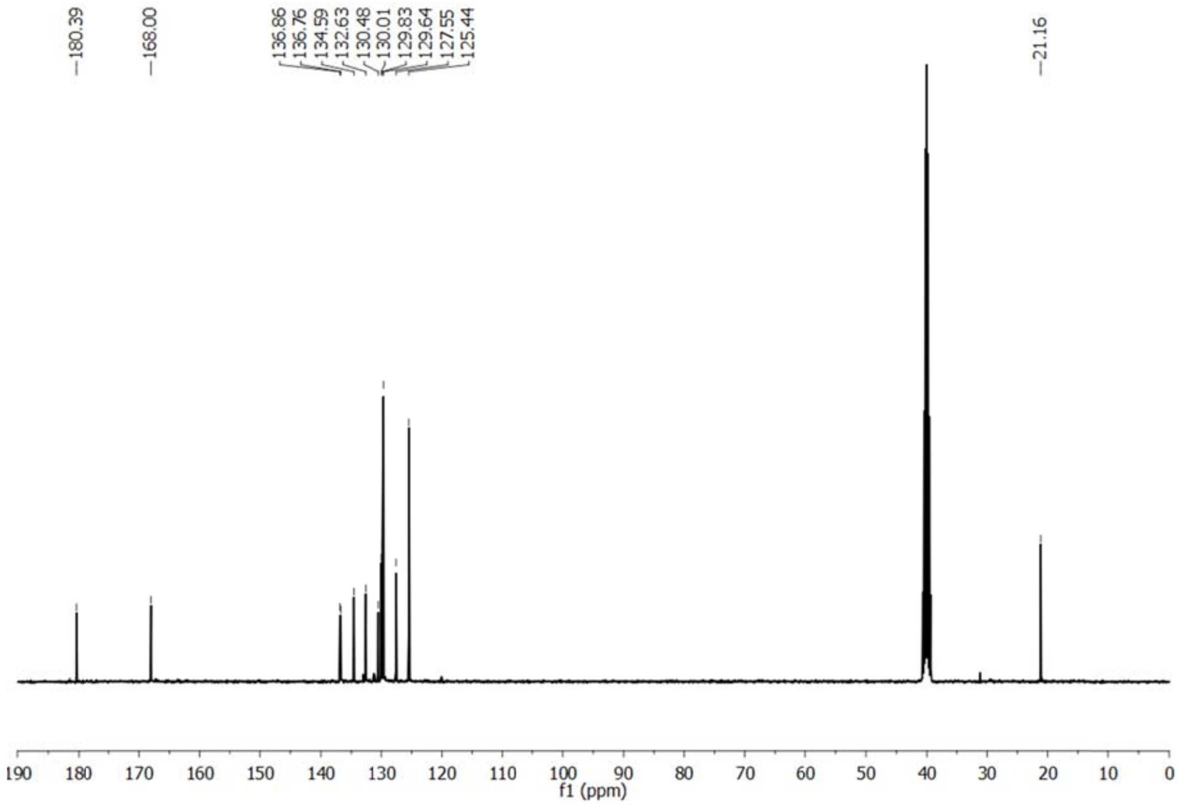

Figure S47. ¹³C-NMR of compound **2b**.

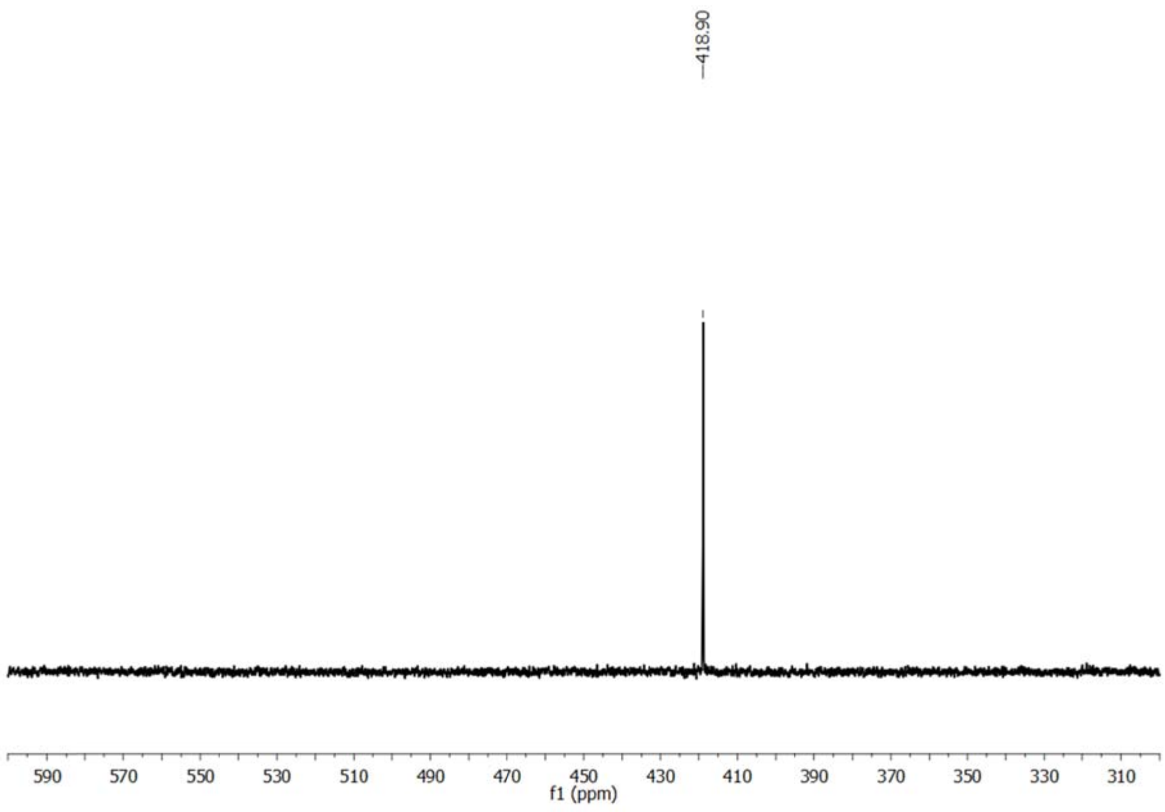

Figure S48. ^{77}Se-NMR of compound **2b**.

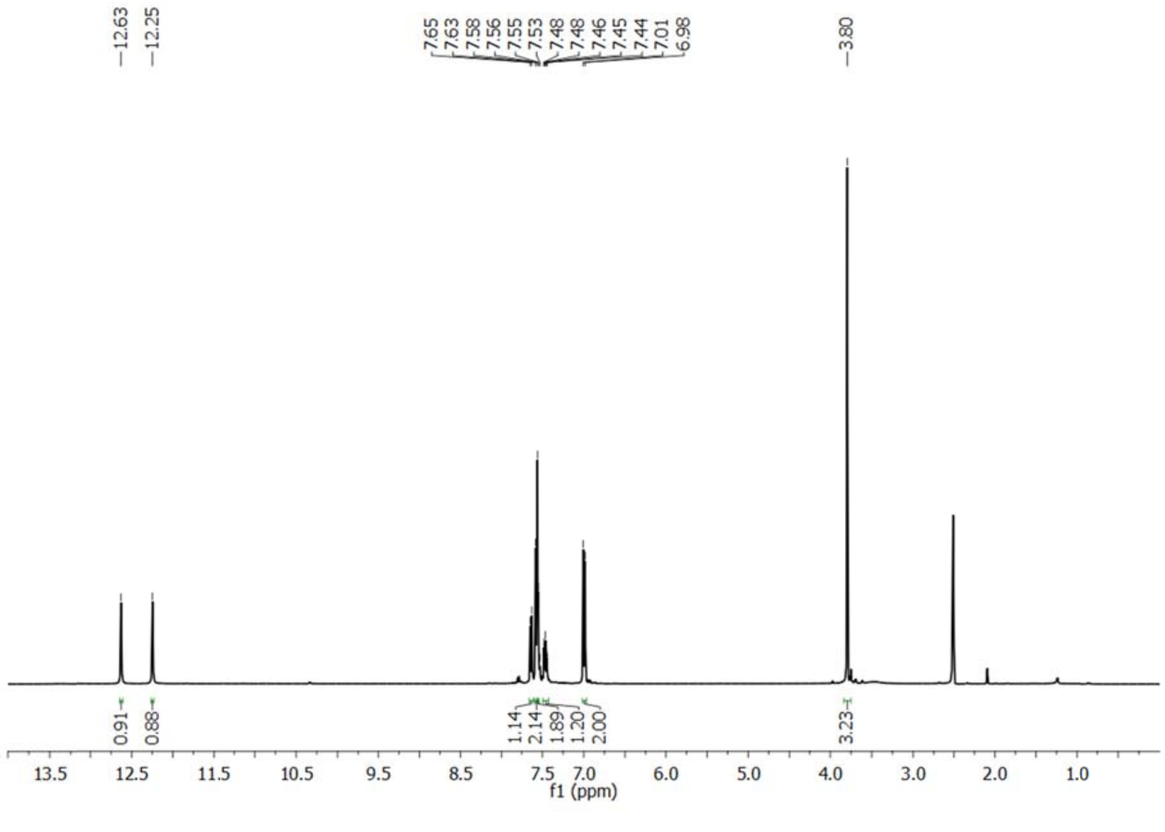

Figure S49. ^{1}H-NMR of compound **2c**.

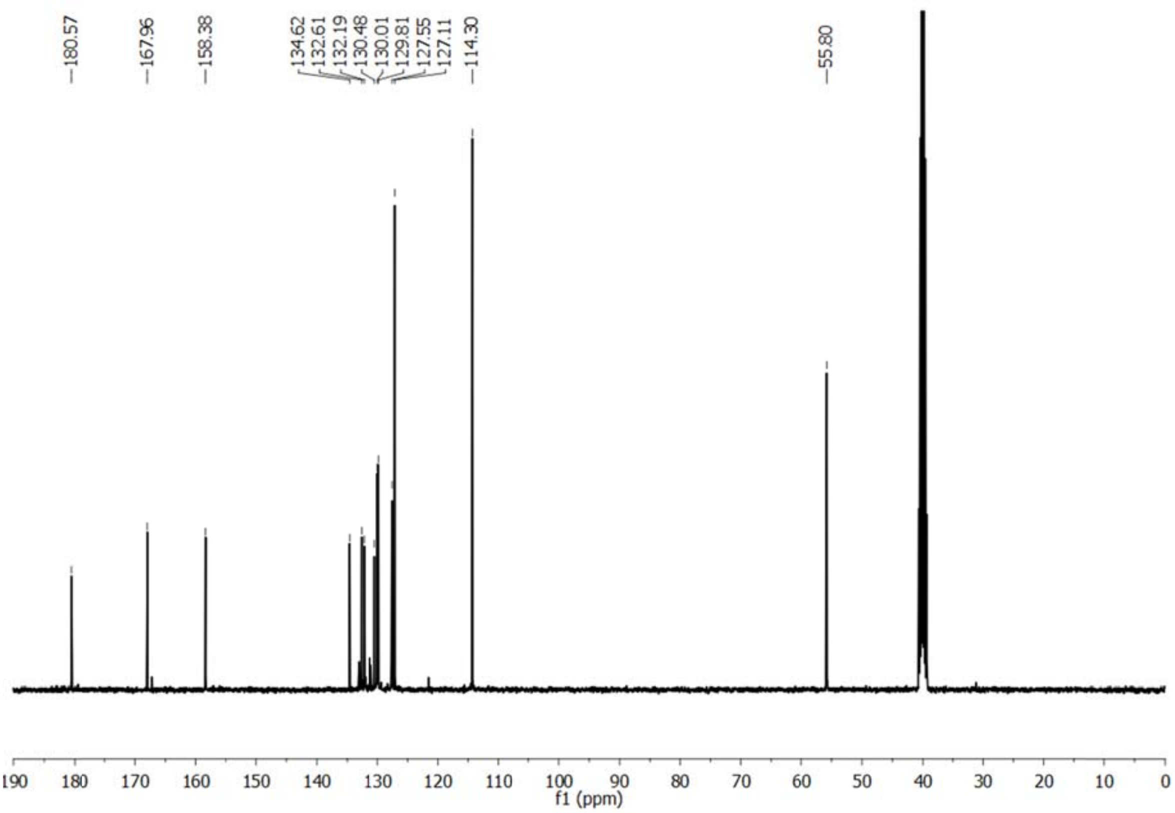

Figure S50. ^{13}C-NMR of compound **2c.**

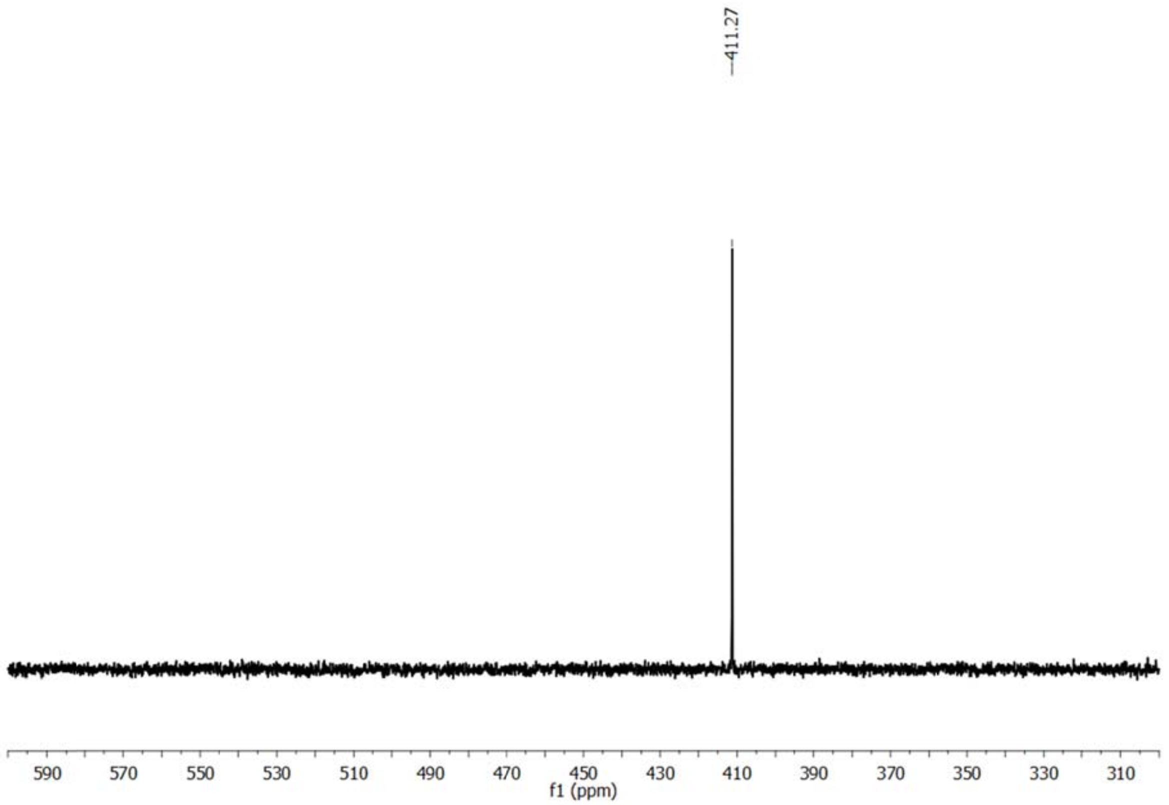

Figure S51. ^{77}Se-NMR of compound **2c**.

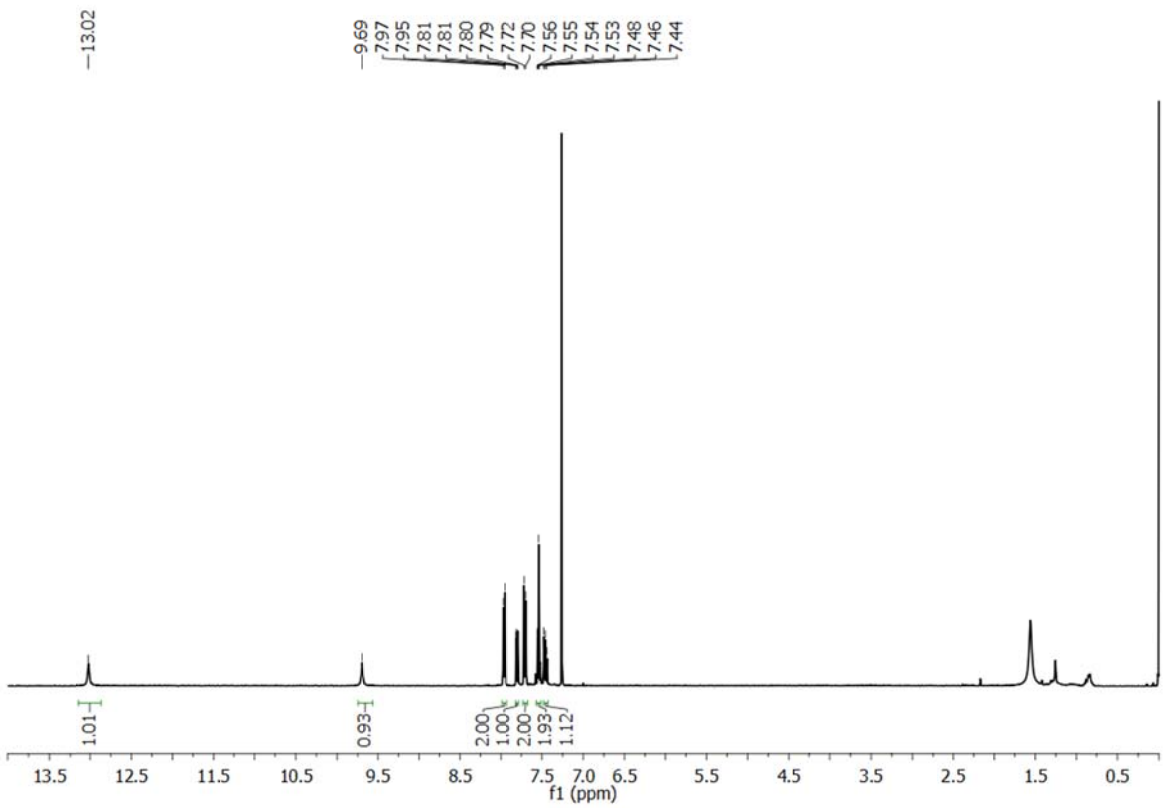

Figure S52. ^{1}H-NMR of compound **2d**.

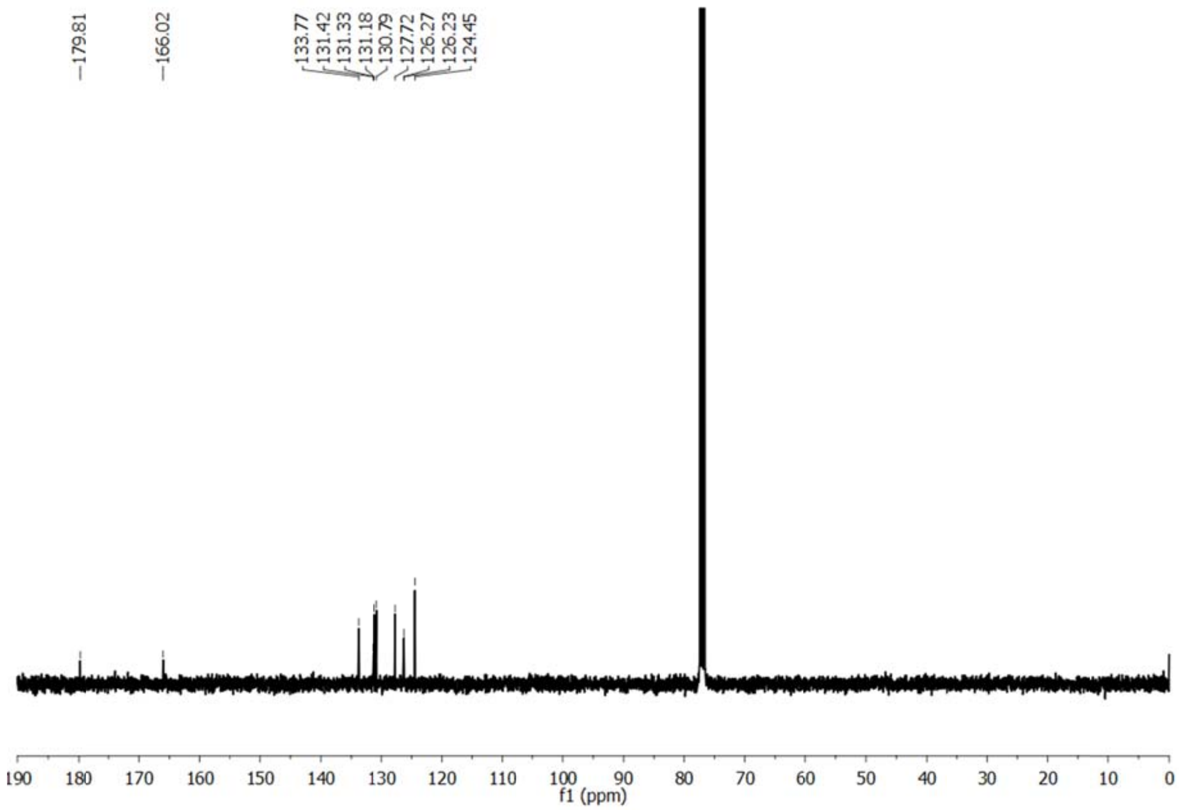

Figure S53. ^{13}C-NMR of compound **2d**.

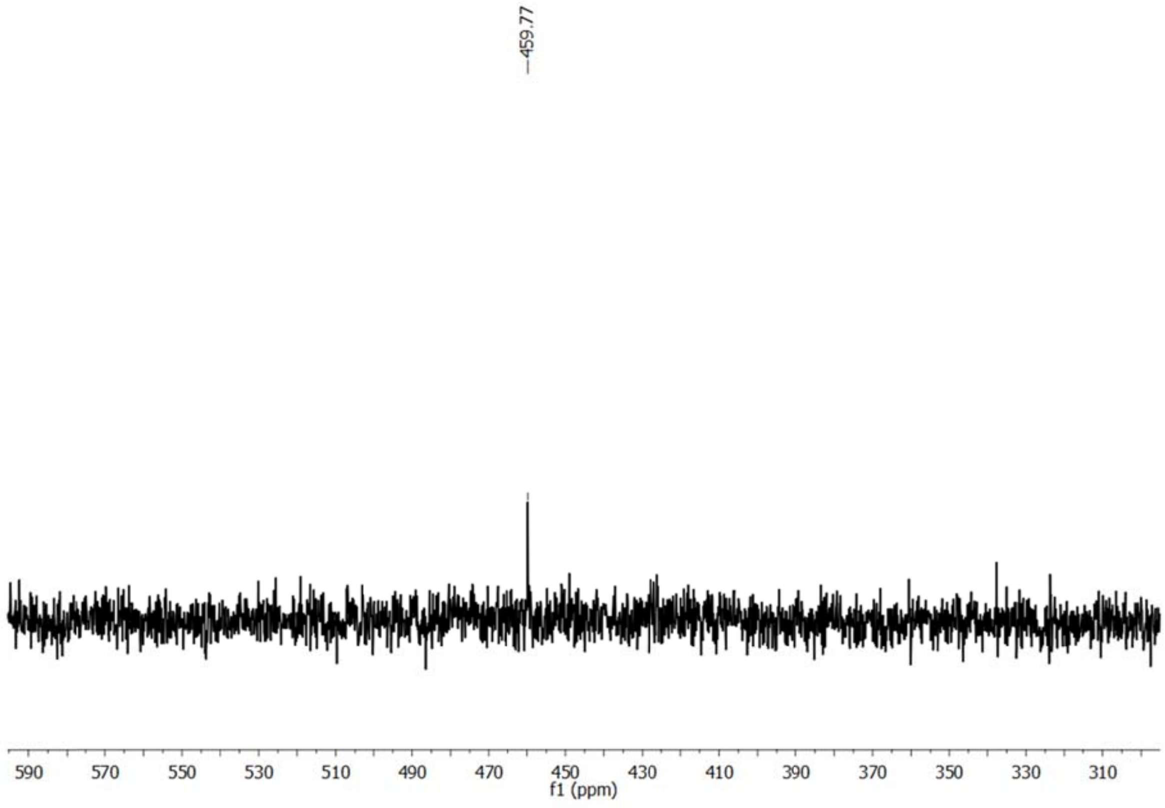

Figure S54. ^{77}Se-NMR of compound **2d**.

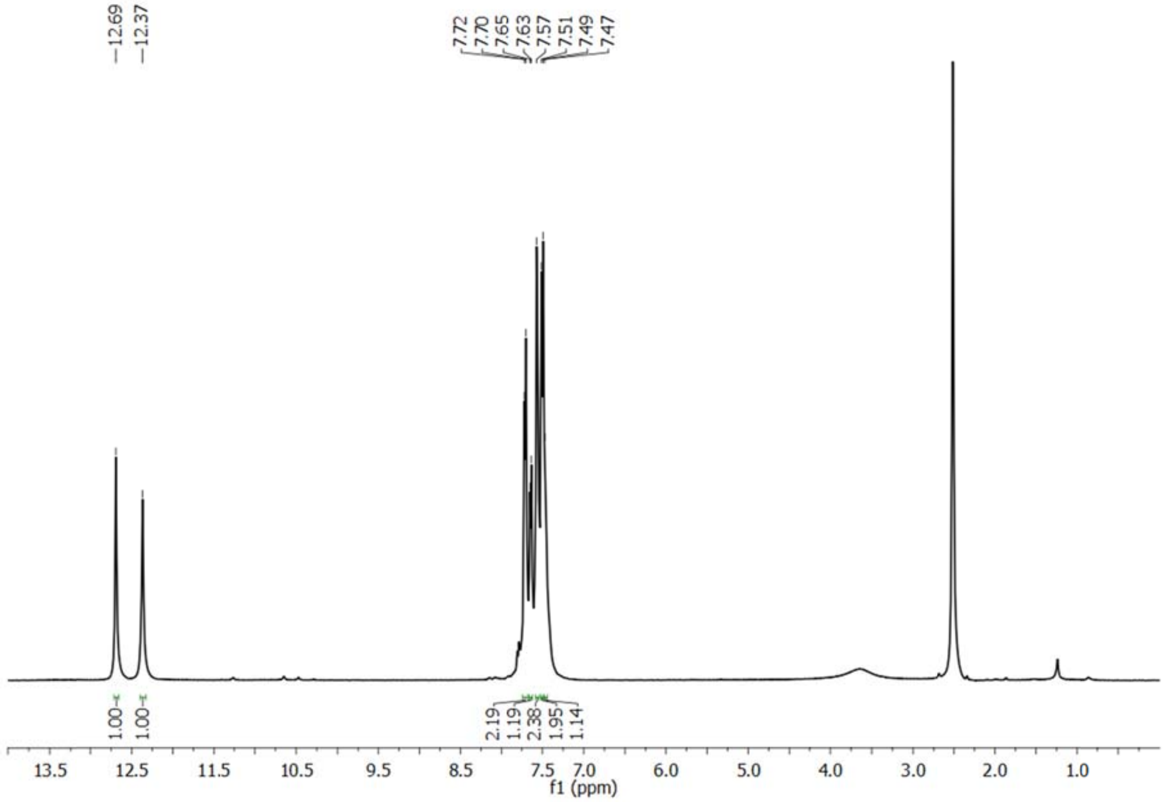

Figure S55. ^{1}H-NMR of compound **2e**.

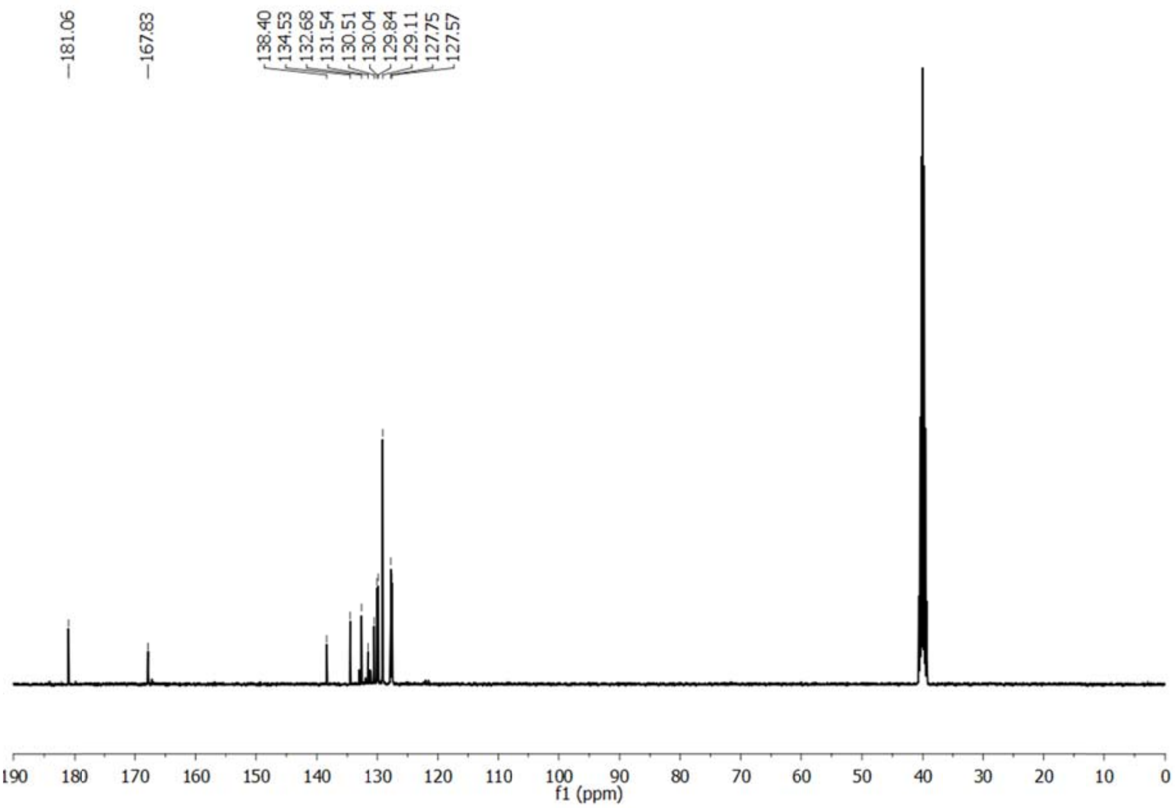

Figure S56. ^{13}C-NMR of compound **2e.**

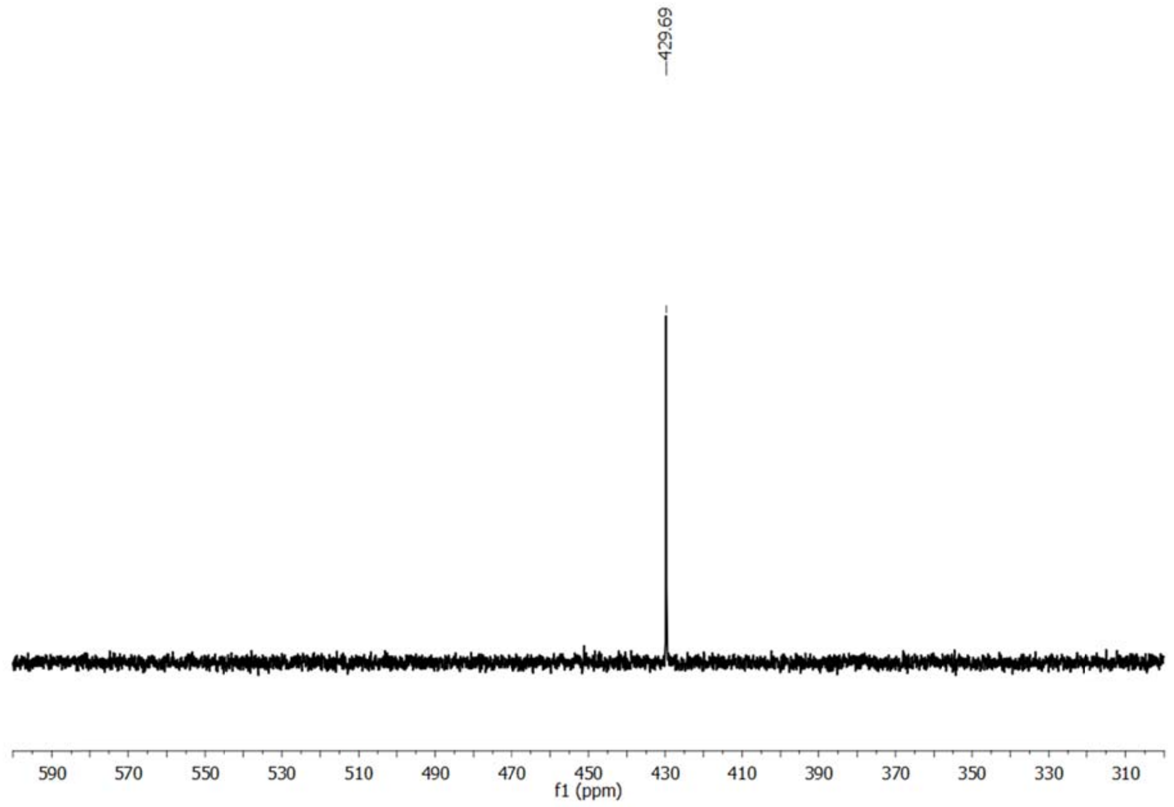

Figure S57. ^{77}Se-NMR of compound **2e**.

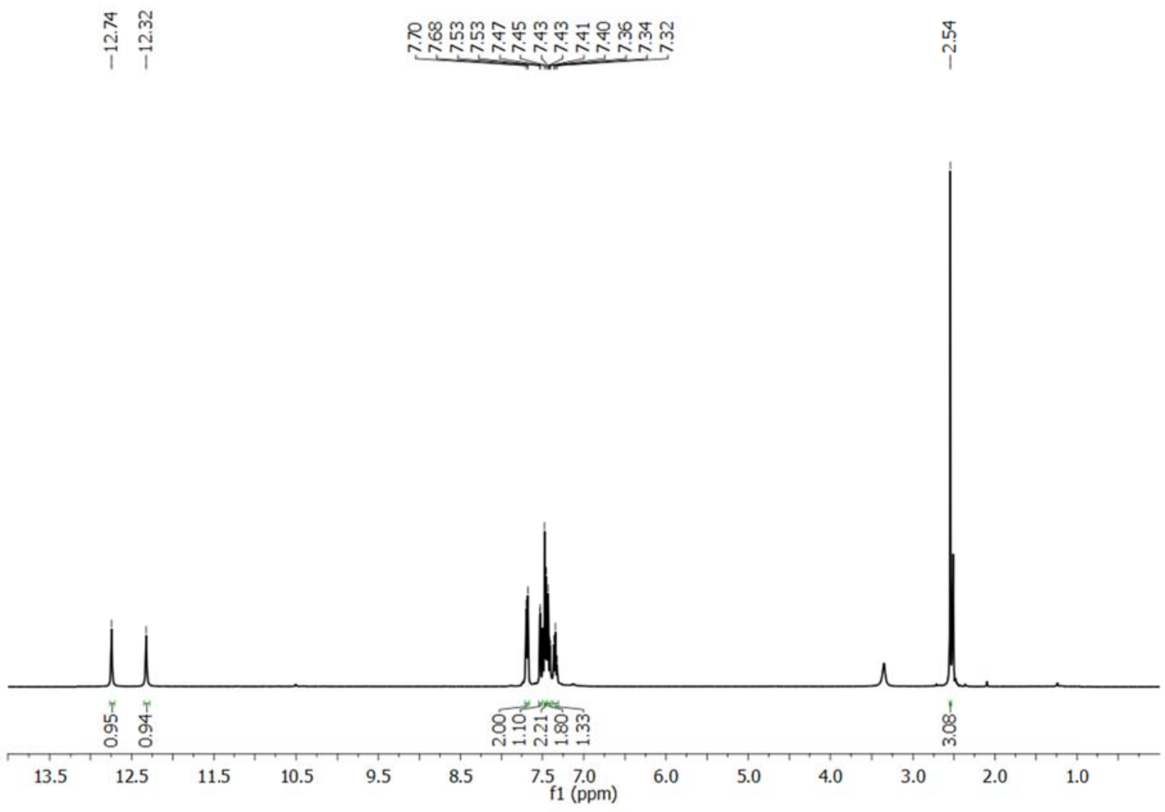

Figure S58. ^{1}H-NMR of compound **3a**.

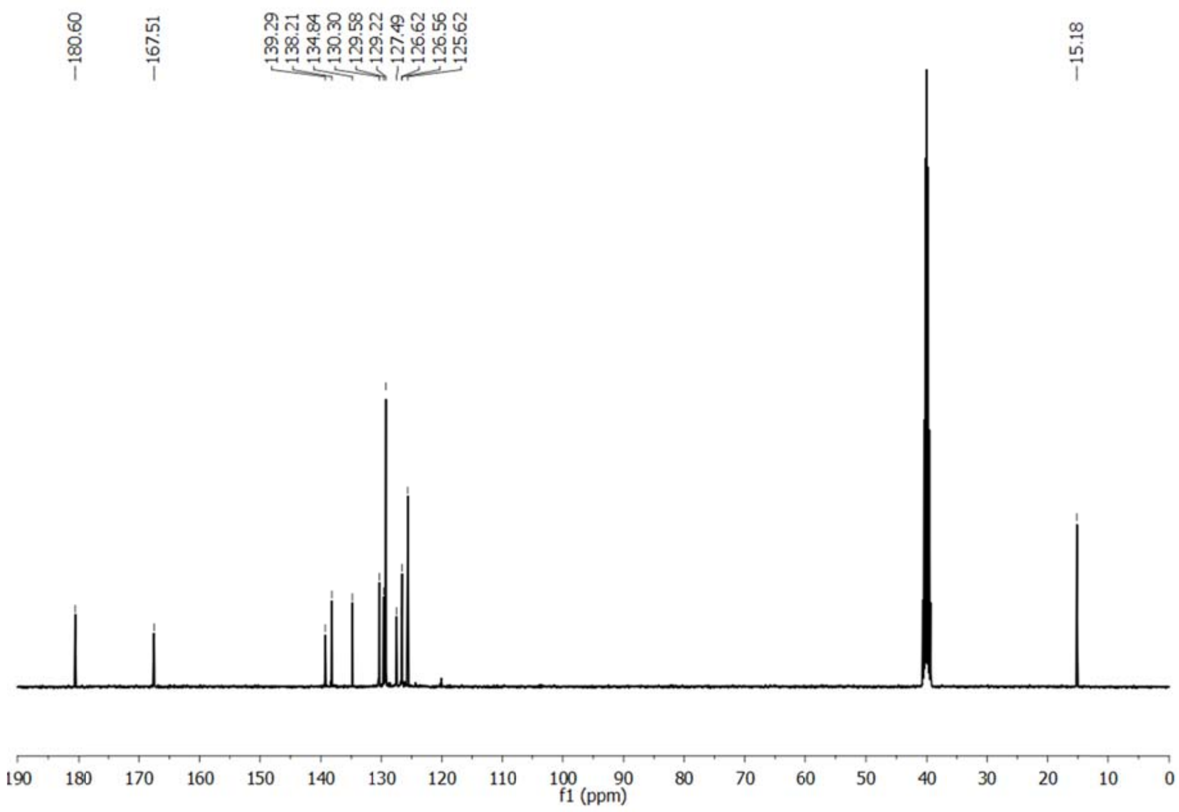

Figure S59. ^{13}C-NMR of compound **3a.**

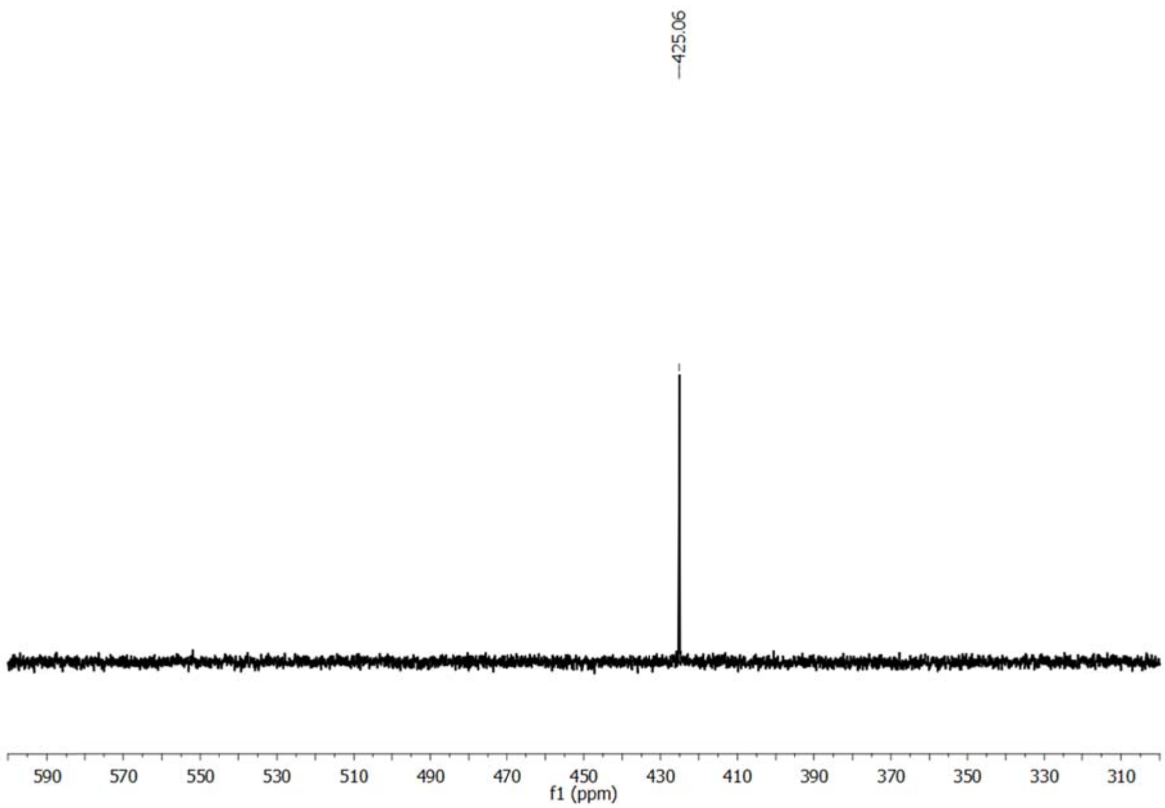

Figure S60. ^{77}Se-NMR of compound **3a**.

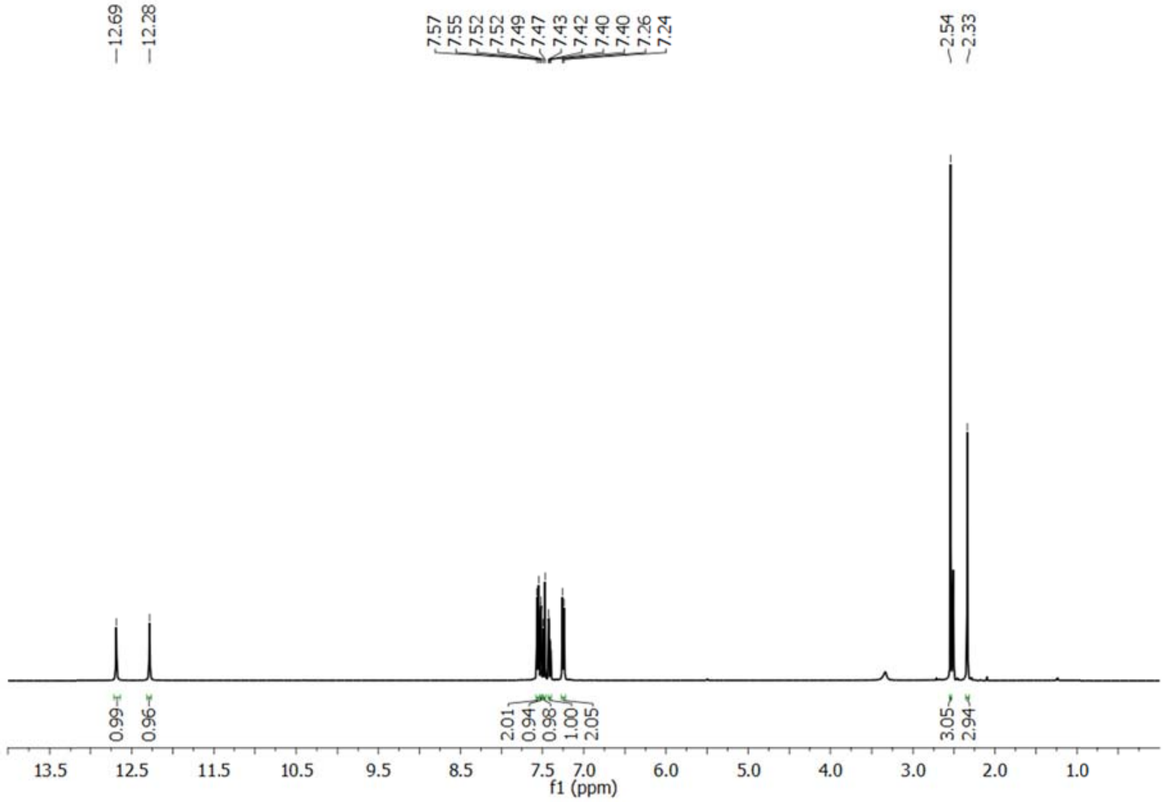

Figure S61. ^{1}H-NMR of compound **3b**.

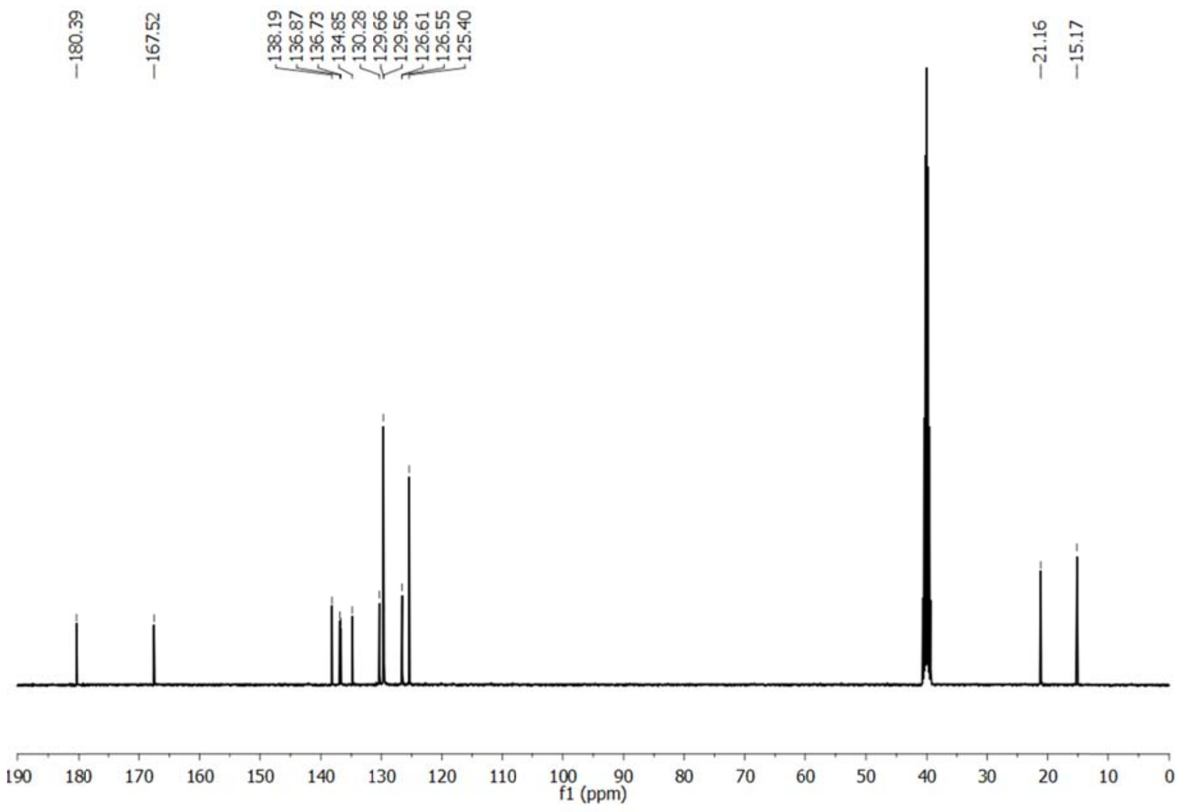

Figure S62. ^{13}C-NMR of compound **3b**.

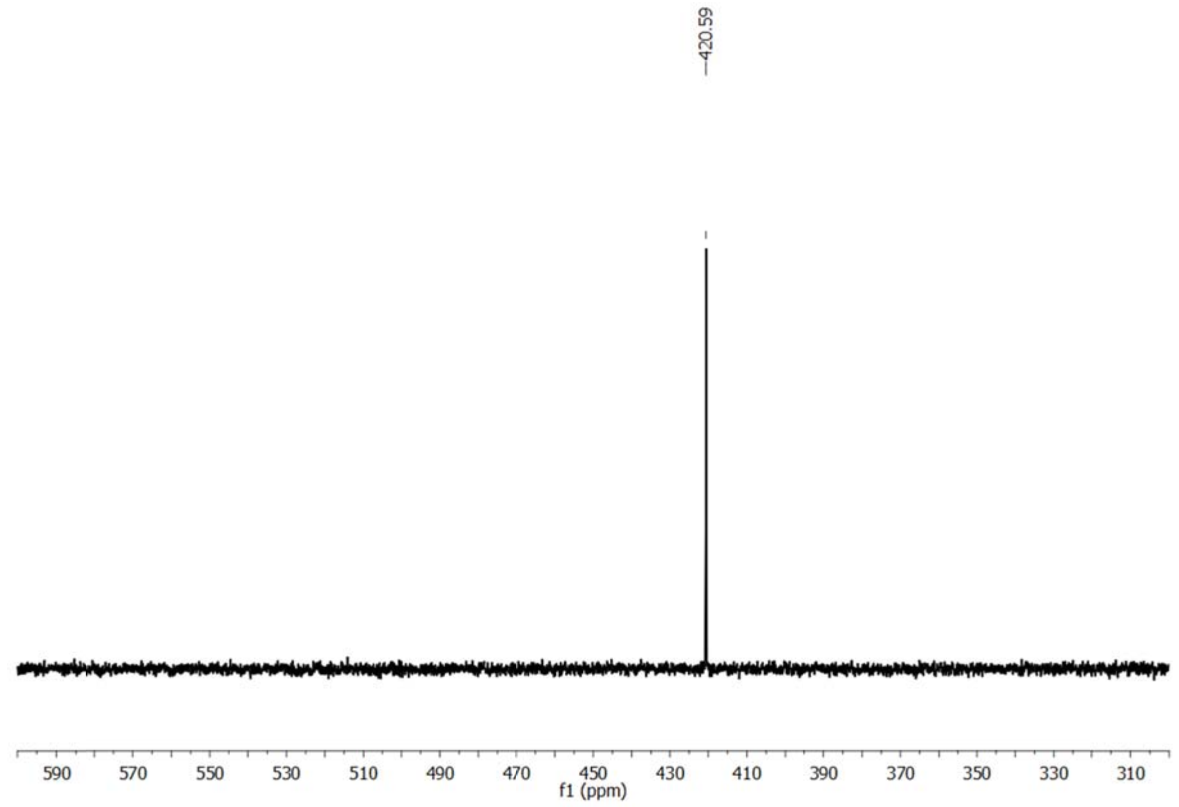

Figure S63. ^{77}Se-NMR of compound **3b**.

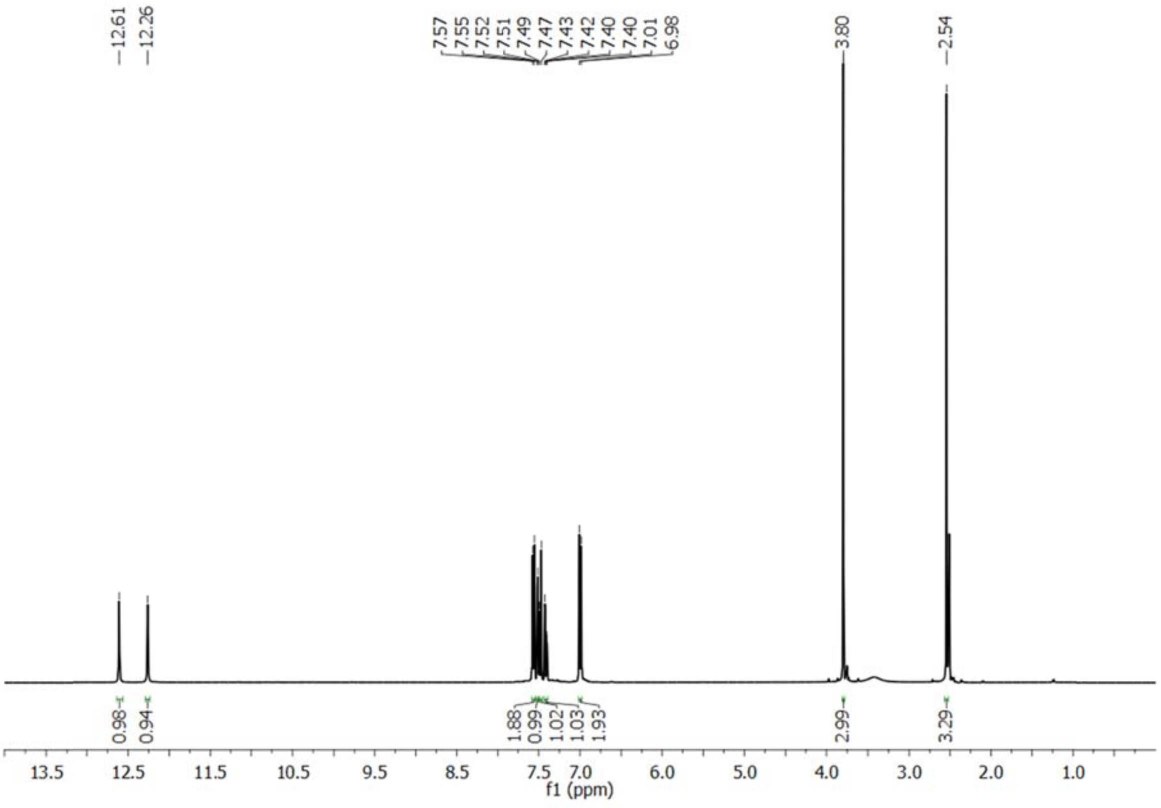

Figure S64. ^{1}H-NMR of compound **3c**.

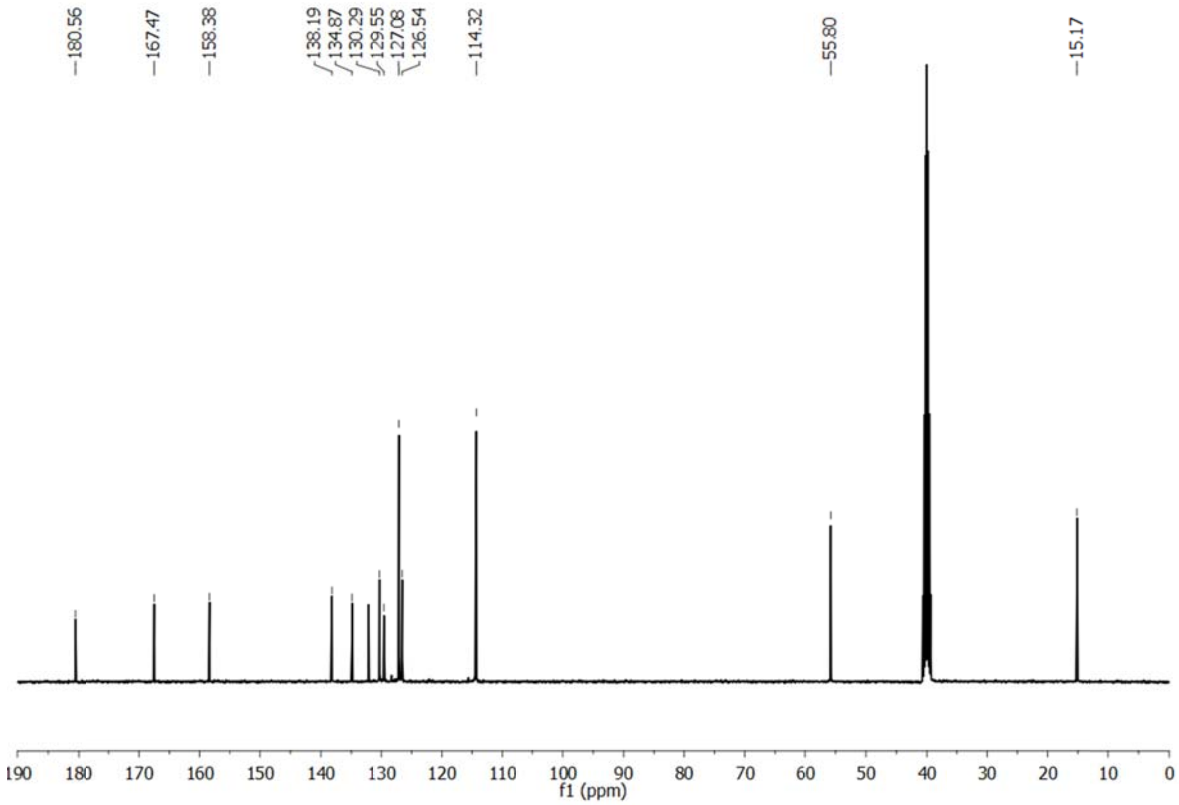

Figure S65. ^{13}C-NMR of compound **3c.**

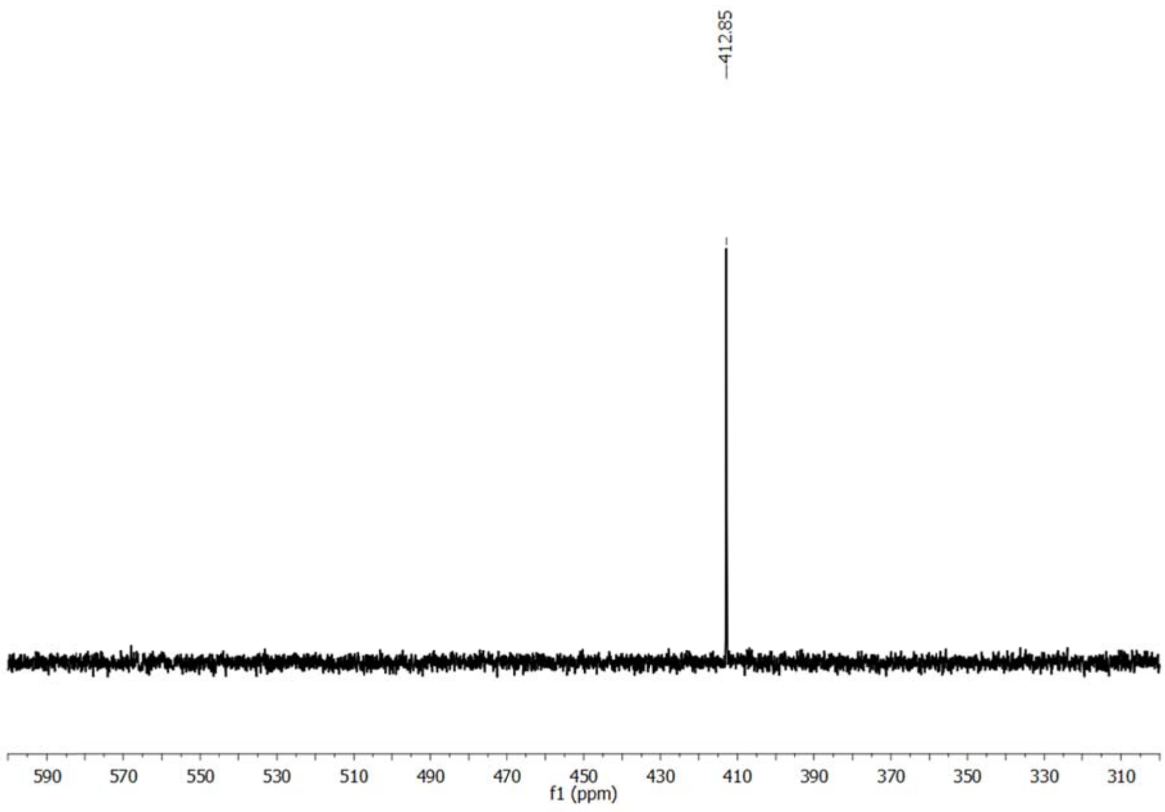

Figure S66. ^{77}Se-NMR of compound **3c**.

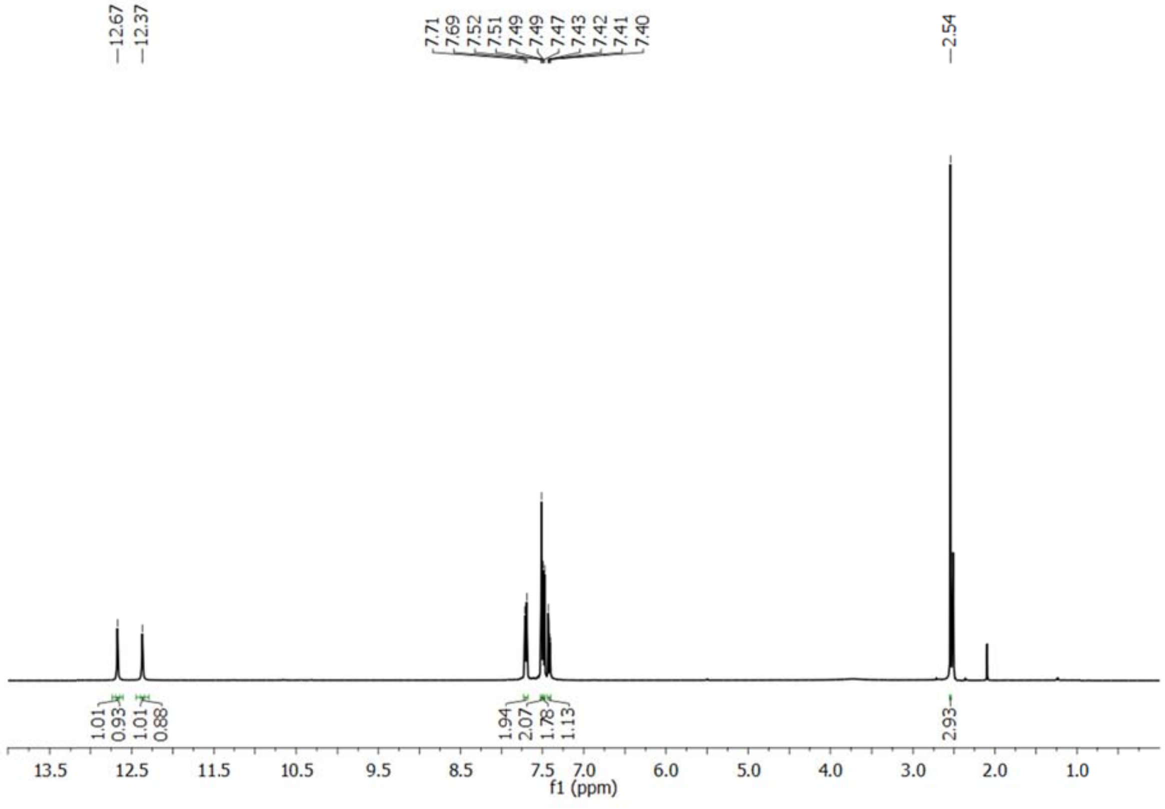

Figure S67. ^{1}H-NMR of compound **3e**.

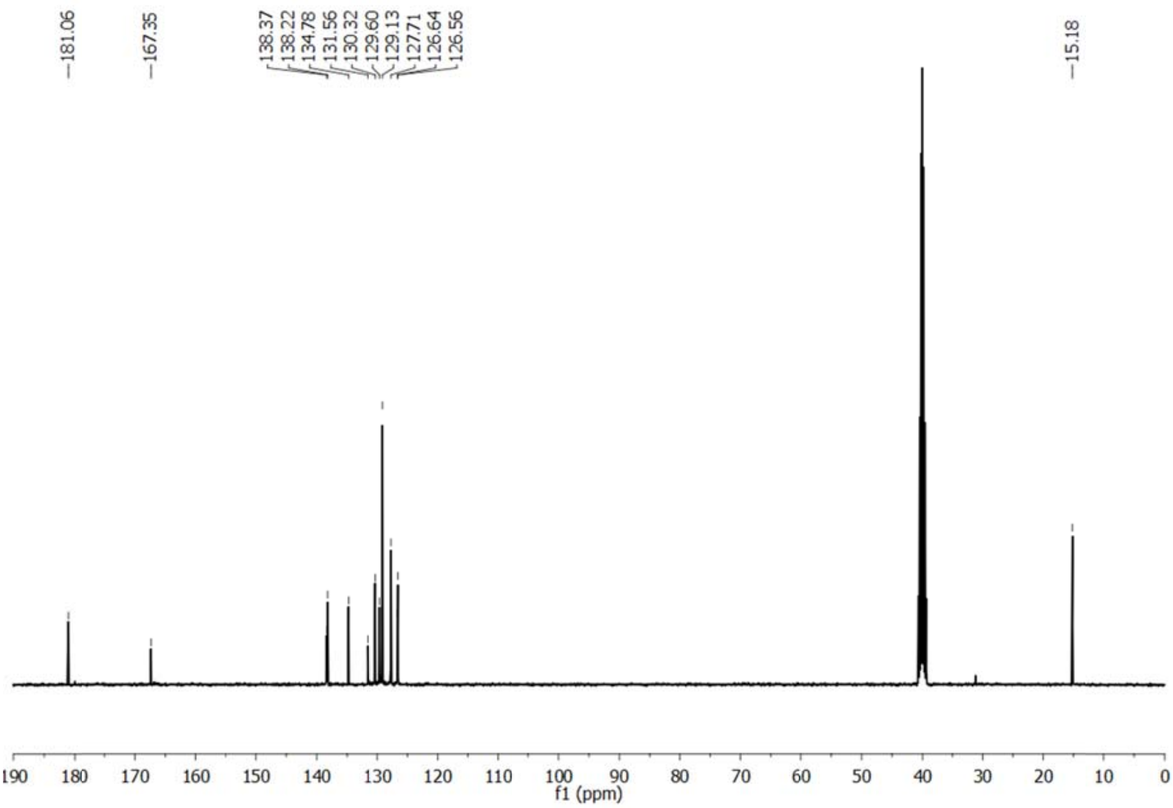

Figure S68. ^{13}C-NMR of compound **3e.**

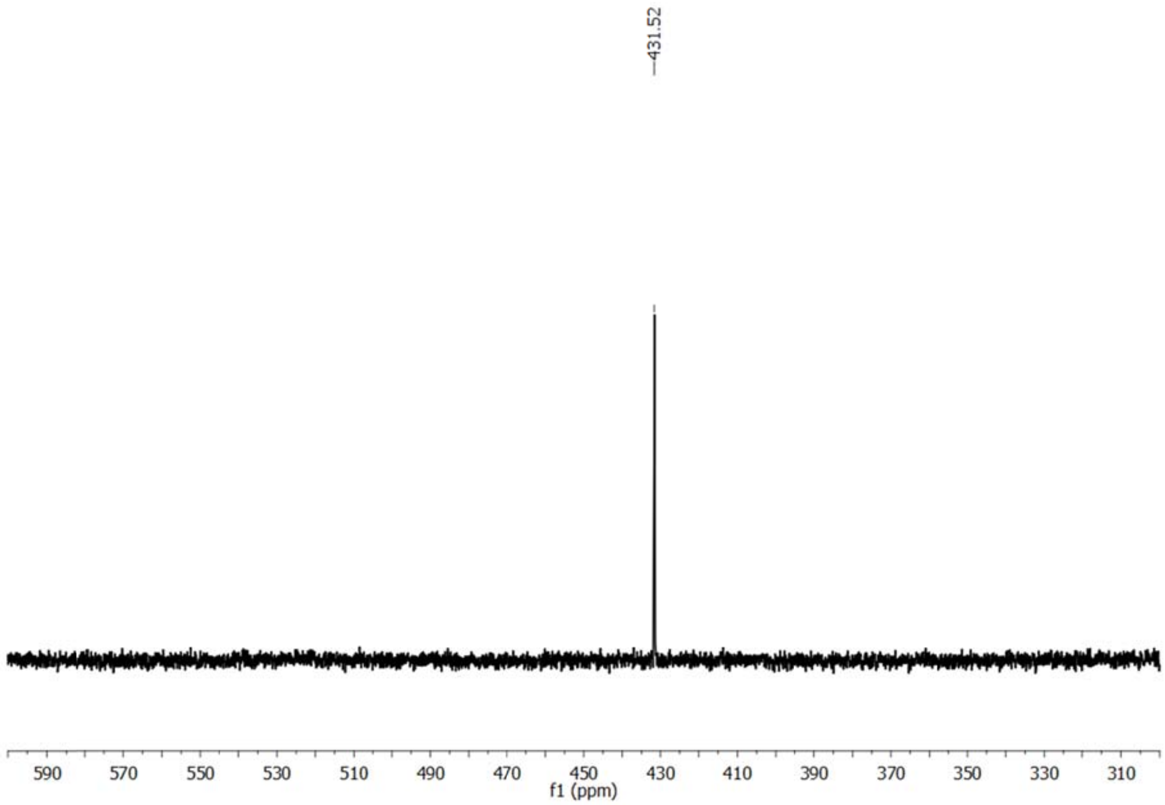

Figure S69. ^{77}Se-NMR of compound **3e**.

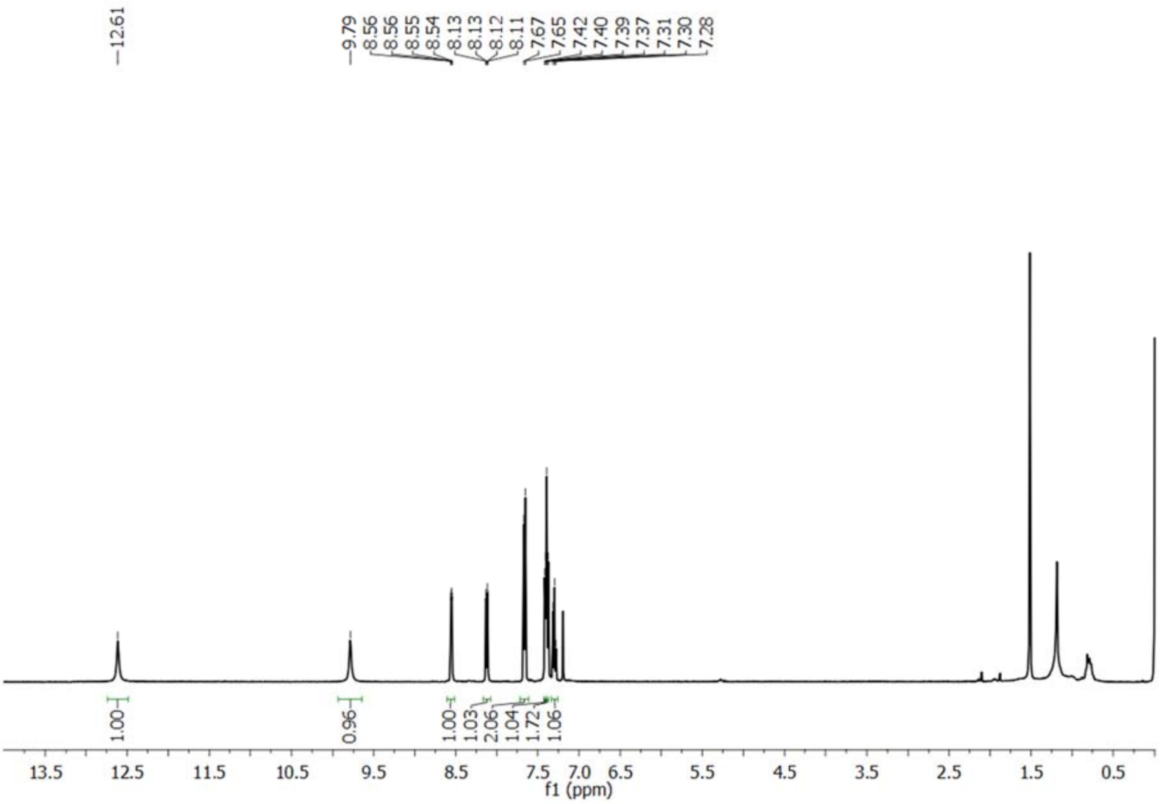

Figure S70. ^{1}H-NMR of compound **4a**.

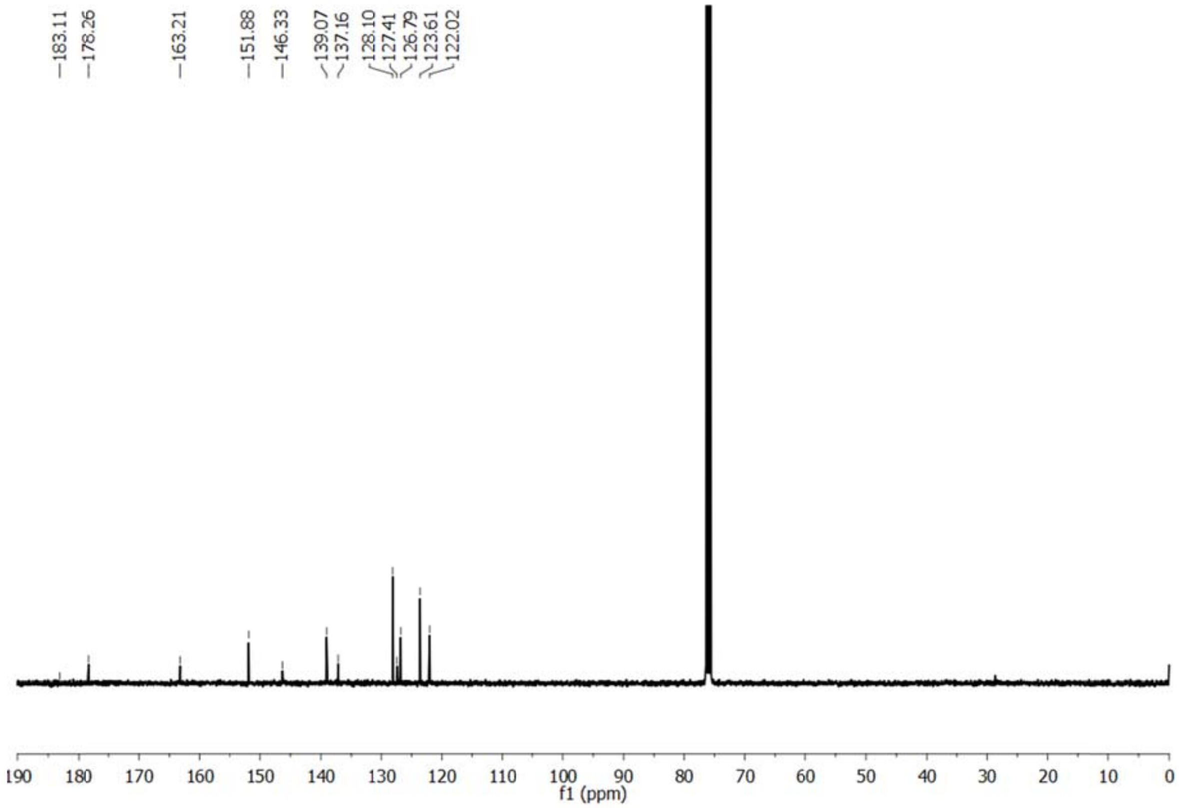

Figure S71. ^{13}C-NMR of compound **4a.**

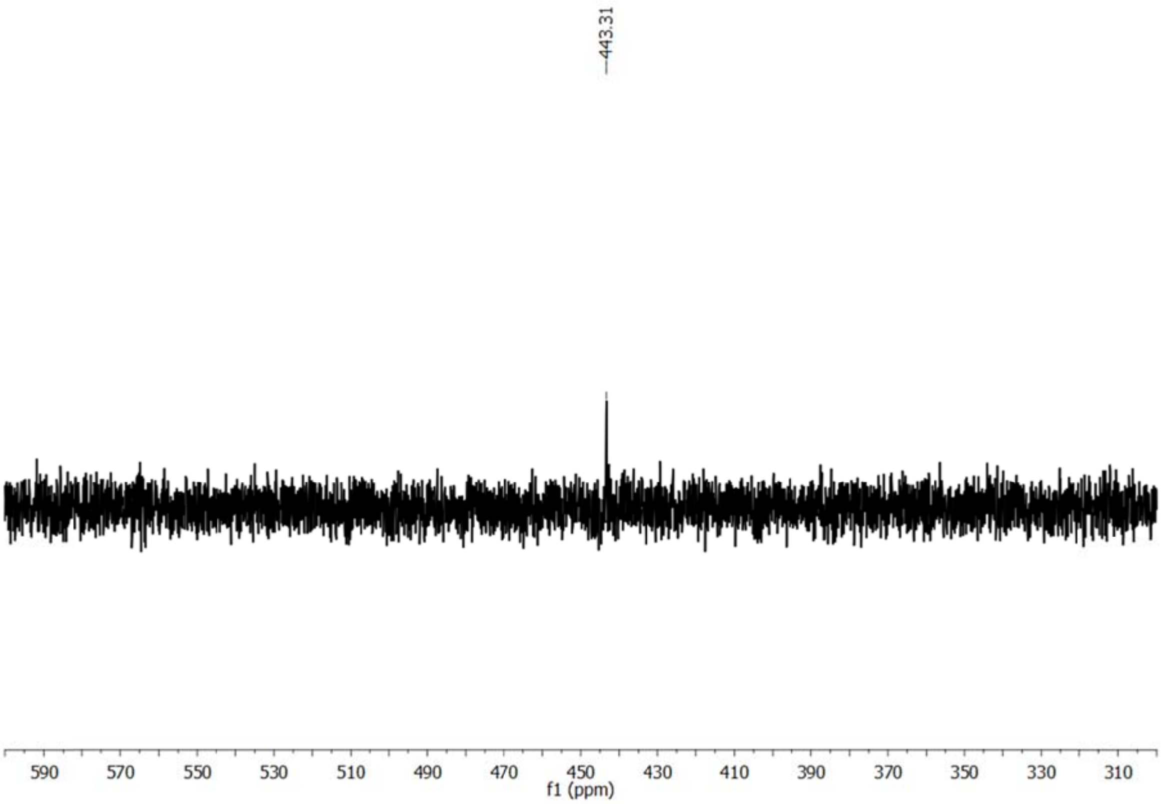

Figure S72. ^{77}Se-NMR of compound **4a**.

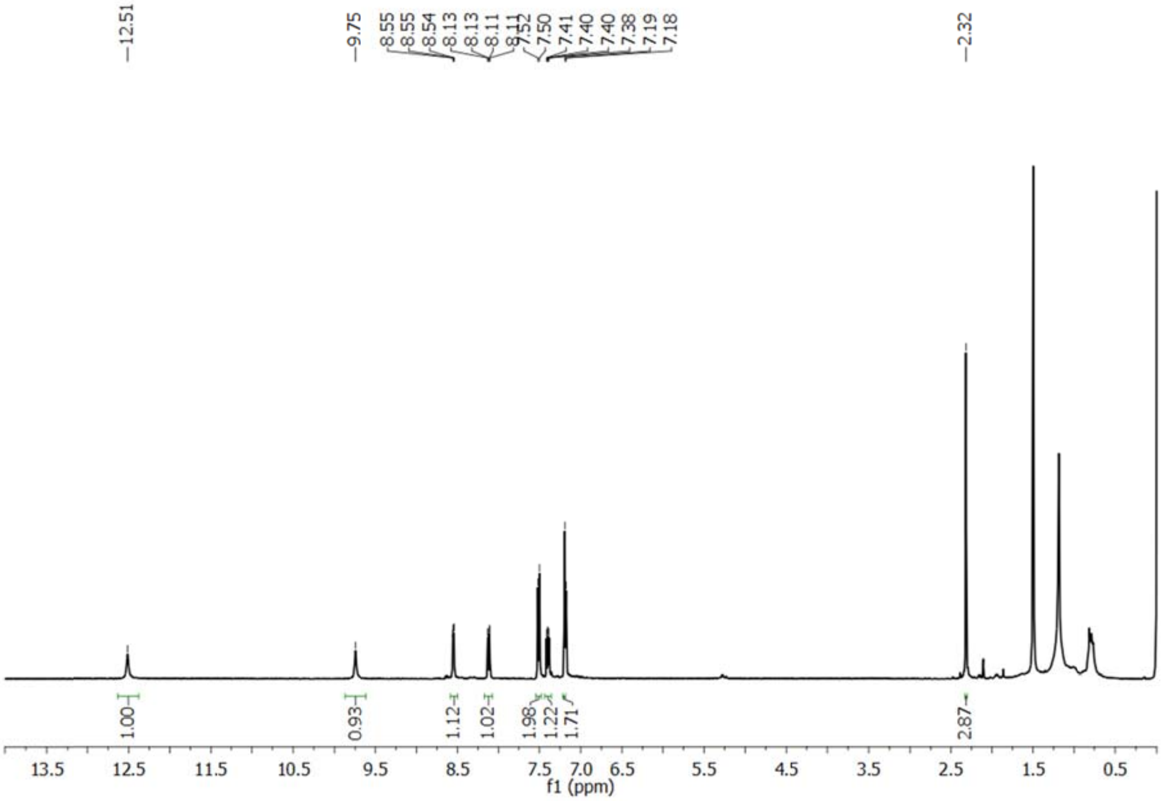

Figure S73. ^{1}H-NMR of compound **4b**.

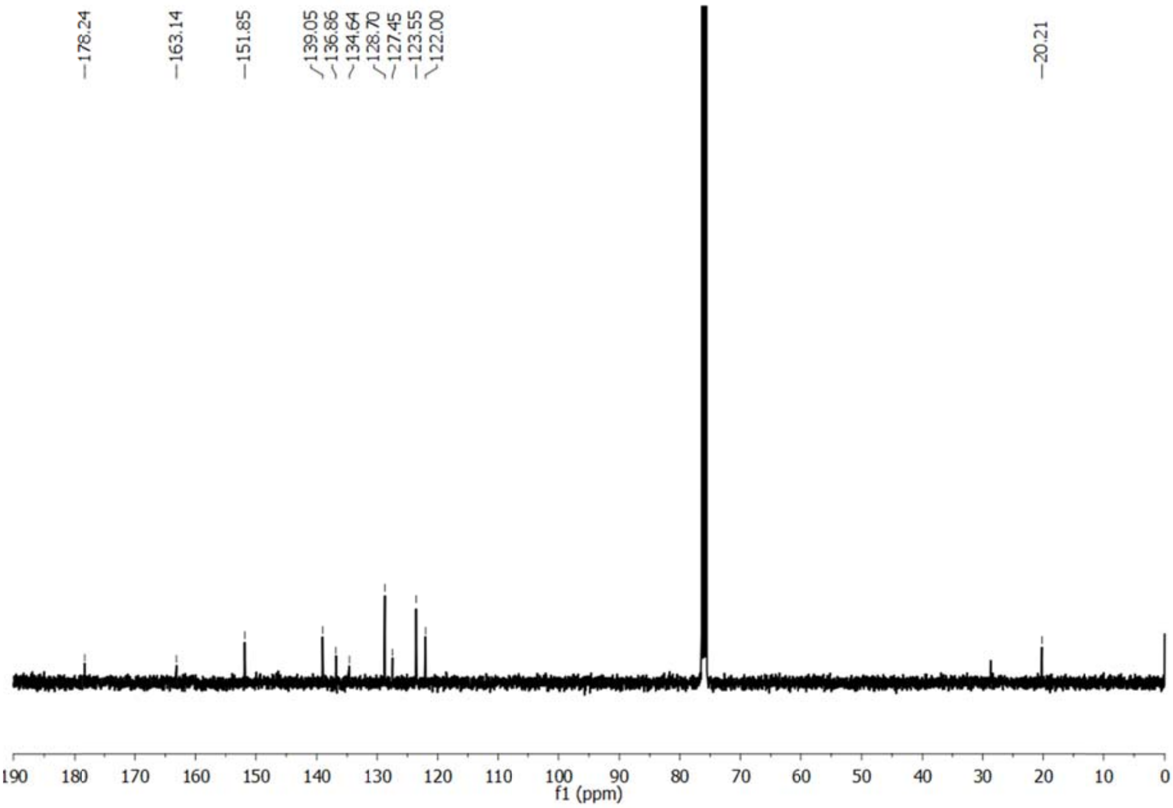

Figure S74. ^{13}C-NMR of compound **4b**.

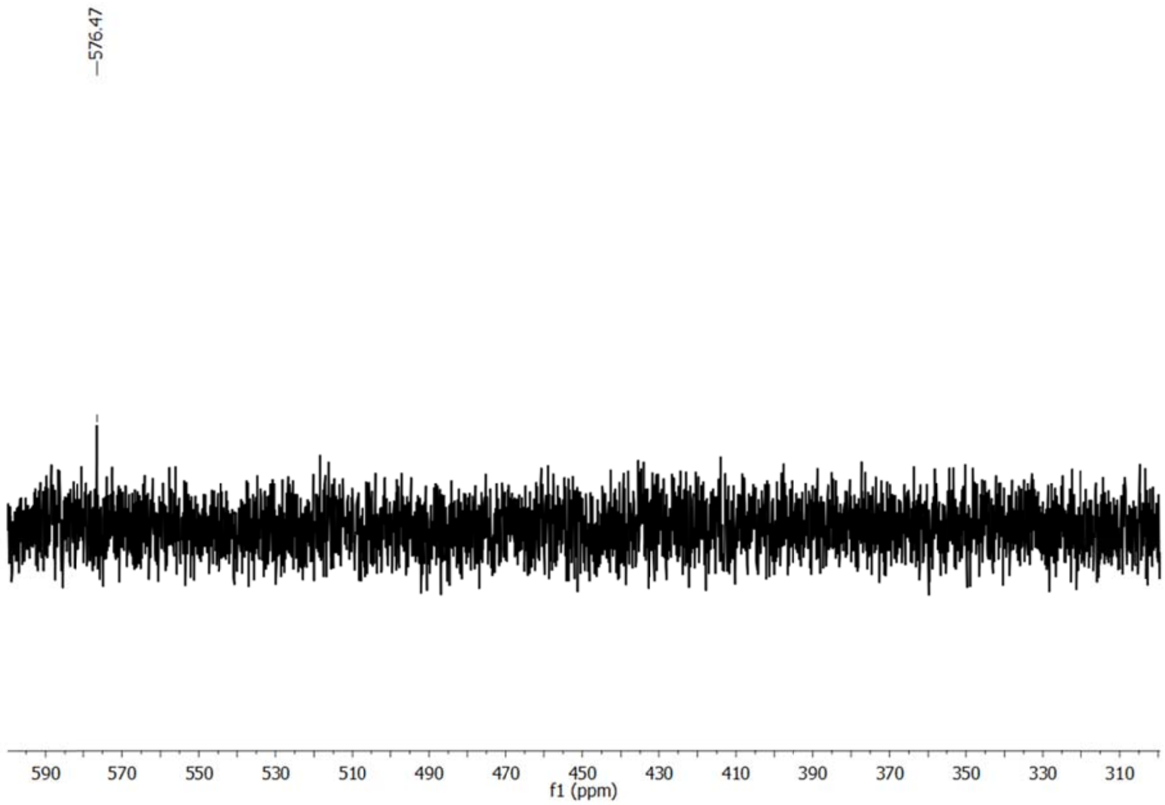

Figure S75. ^{77}Se-NMR of compound **4b**.

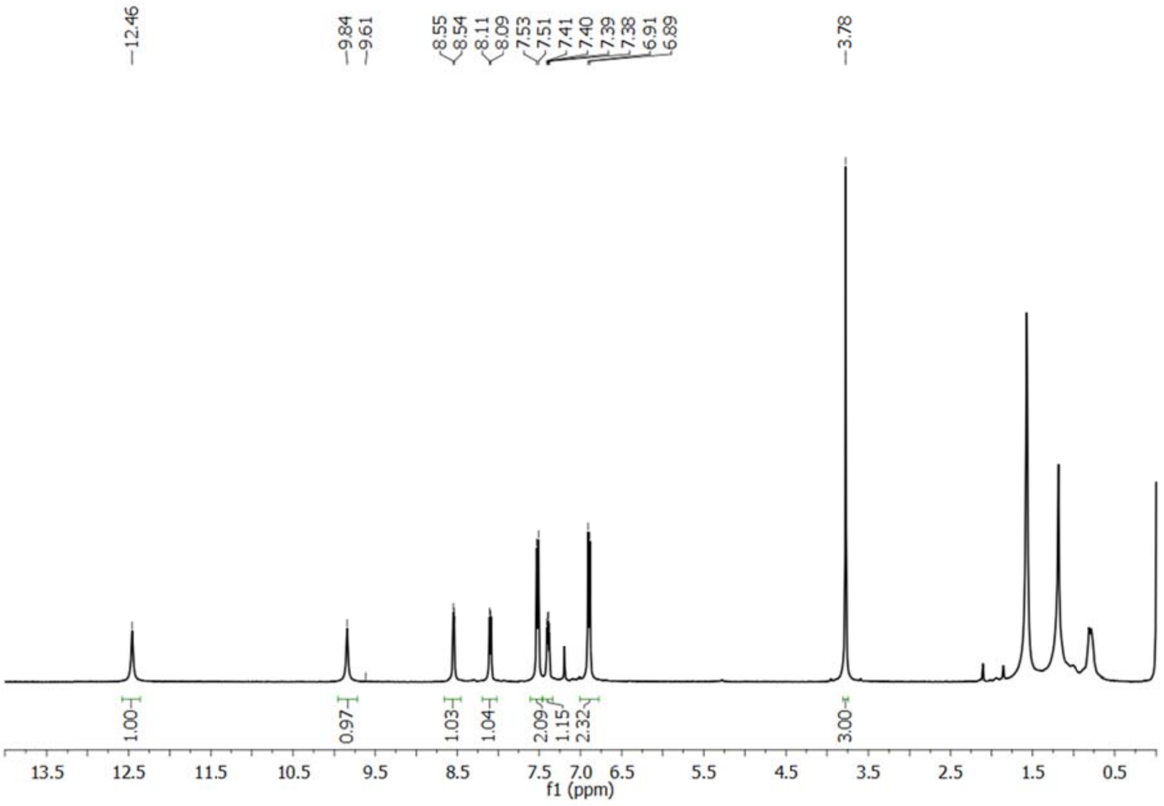

Figure S76. ^{1}H-NMR of compound **4c**.

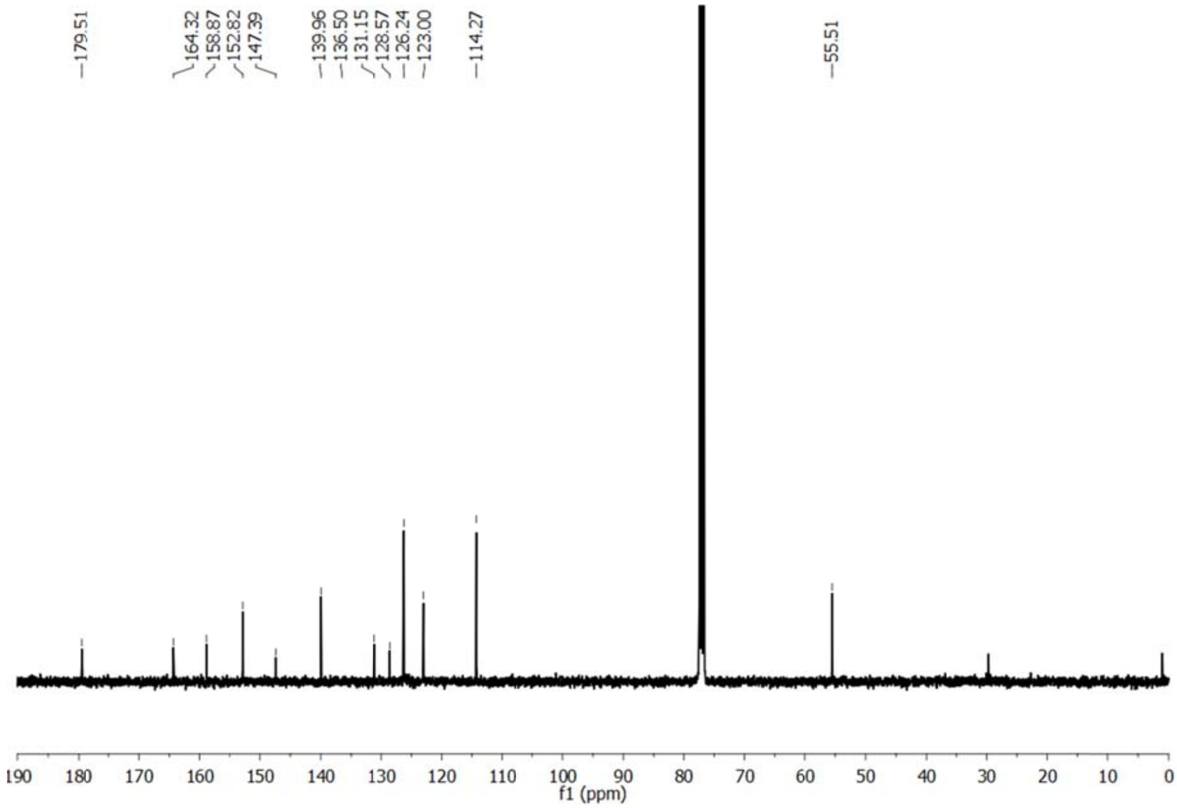

Figure S77. ^{13}C-NMR of compound **4c.**

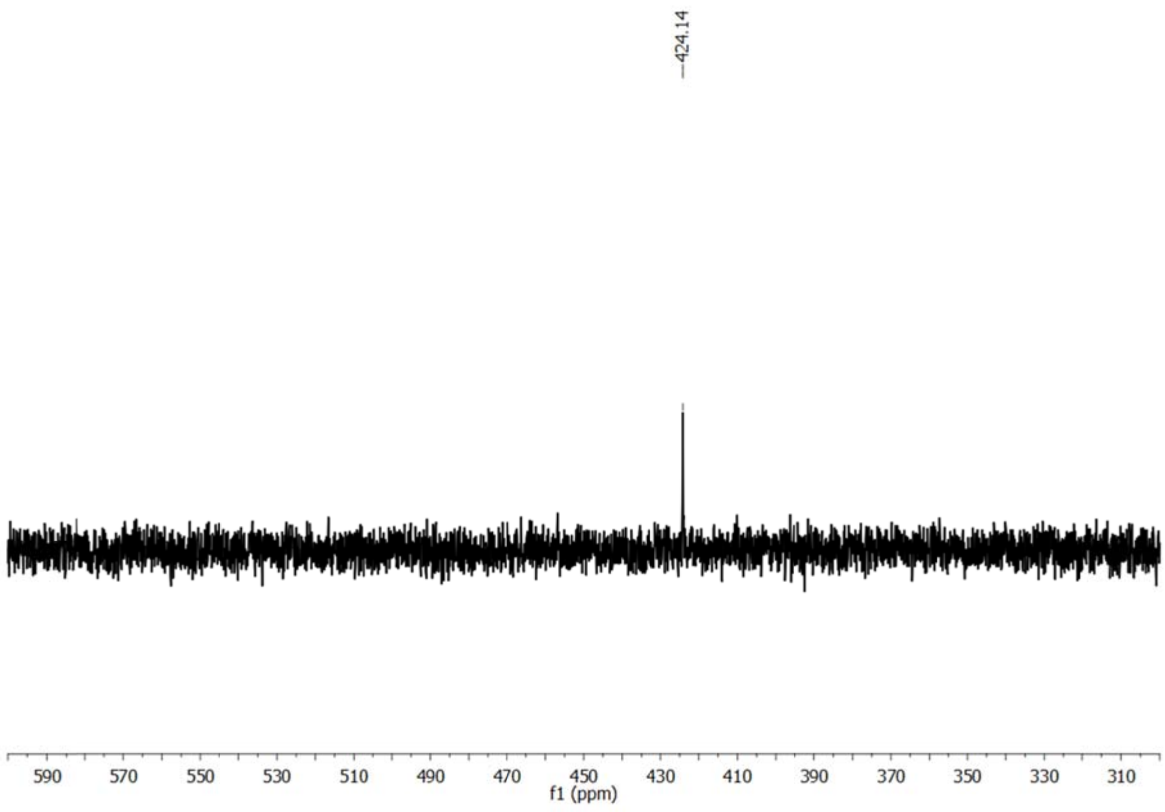

Figure S78. ^{77}Se-NMR of compound **4c**.

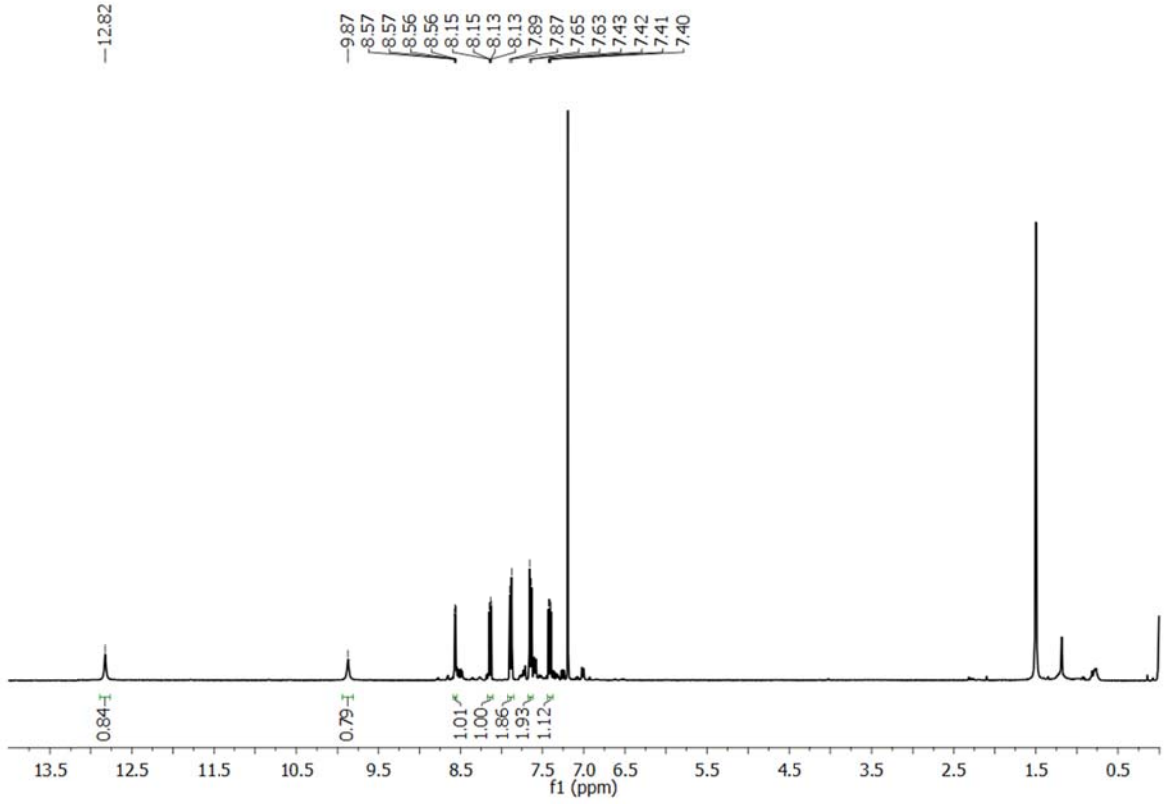

Figure S79. ^{1}H-NMR of compound **4d.**

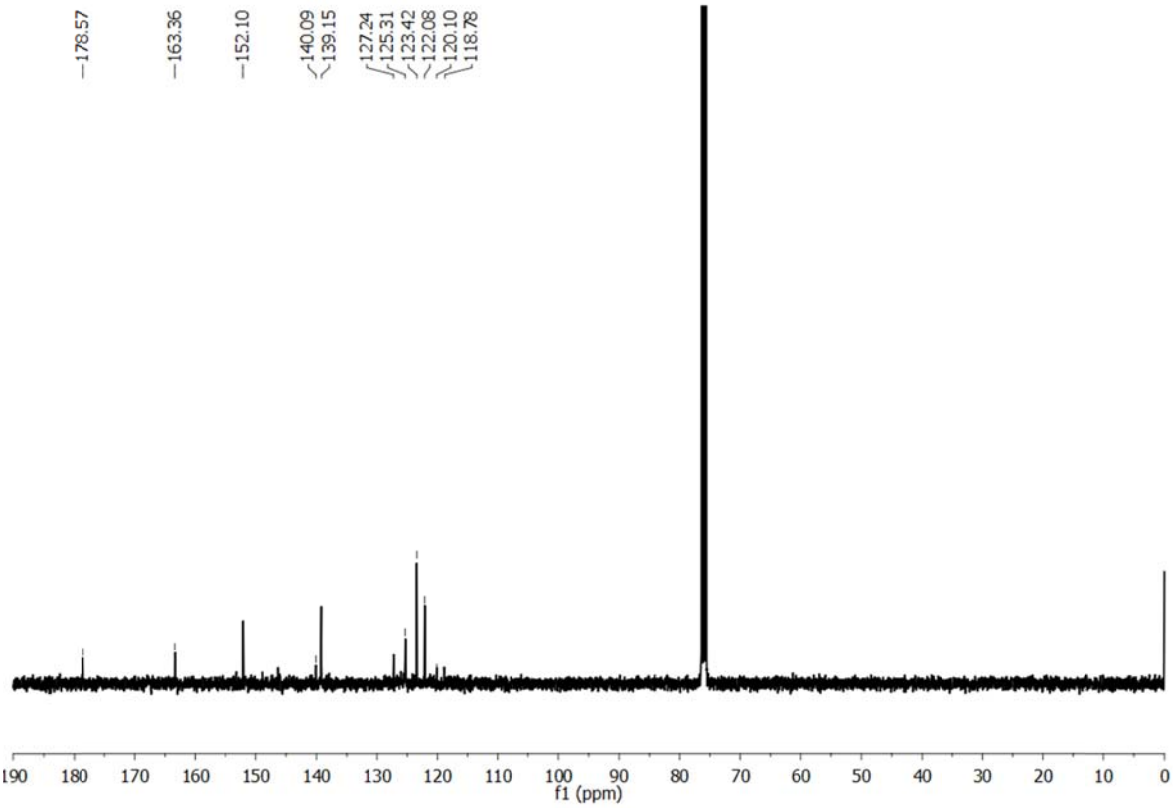

Figure S80. ^{13}C-NMR of compound **4d**.

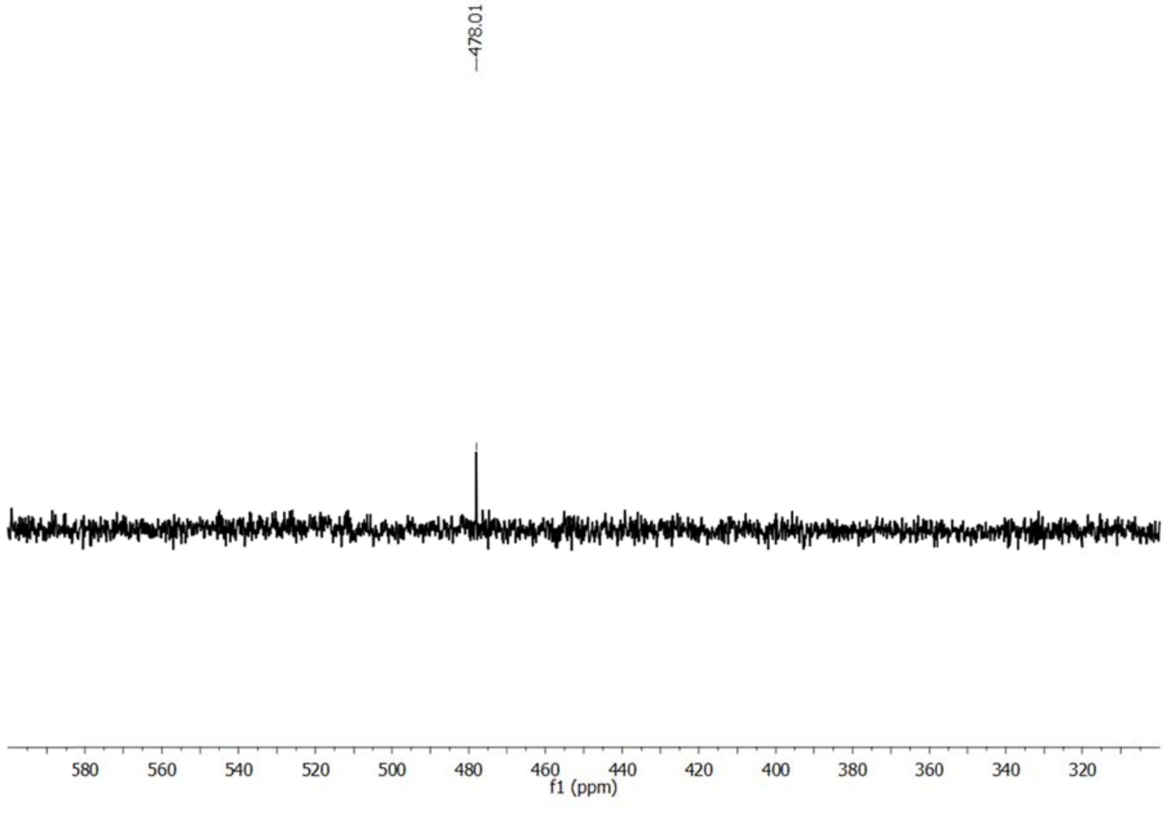

Figure S81. ^{77}Se-NMR of compound **4d**.

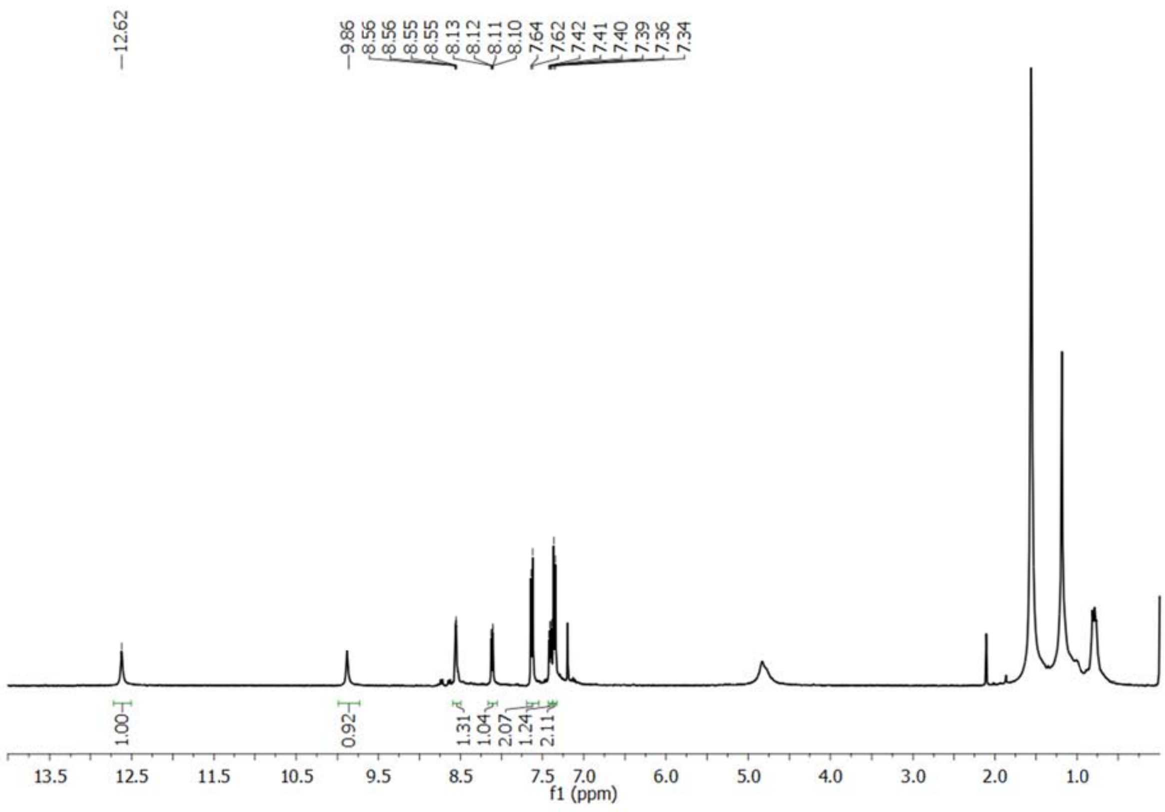

Figure S82. ^{1}H-NMR of compound **4e**.

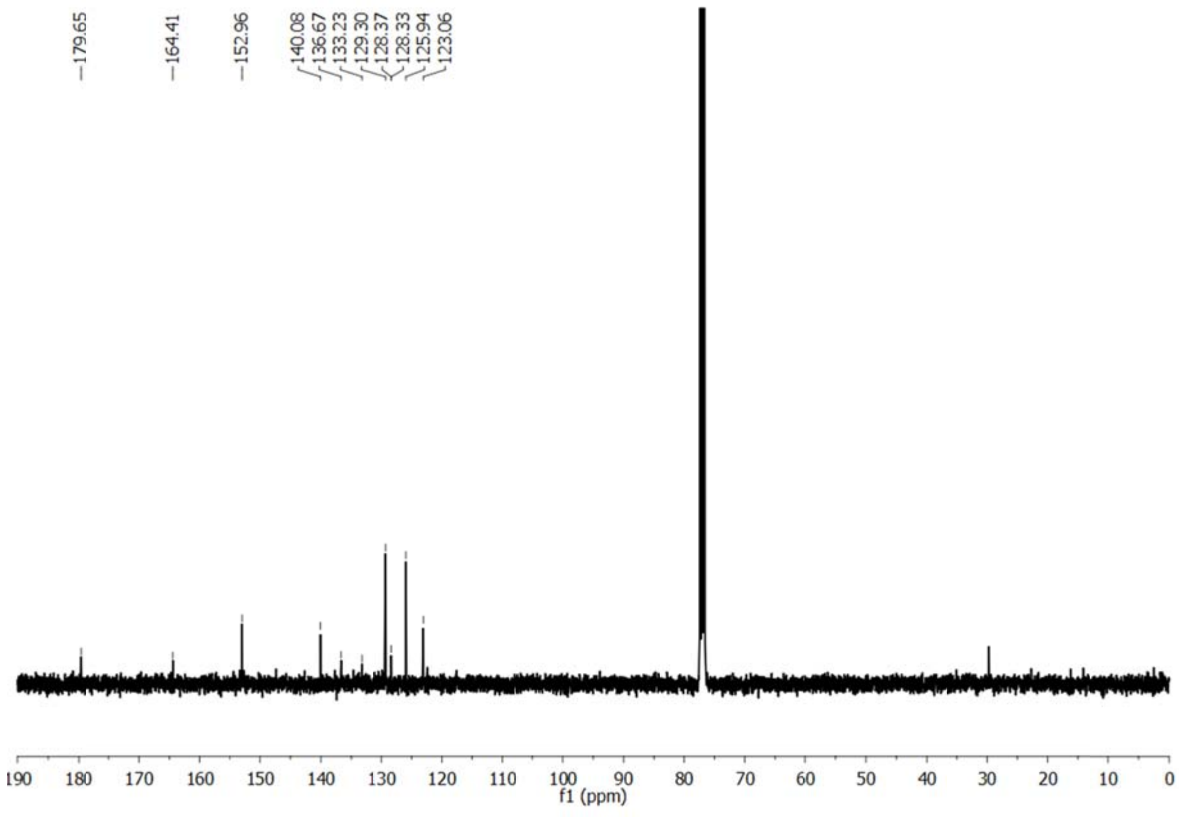

Figure S83. ^{13}C-NMR of compound **4e.**

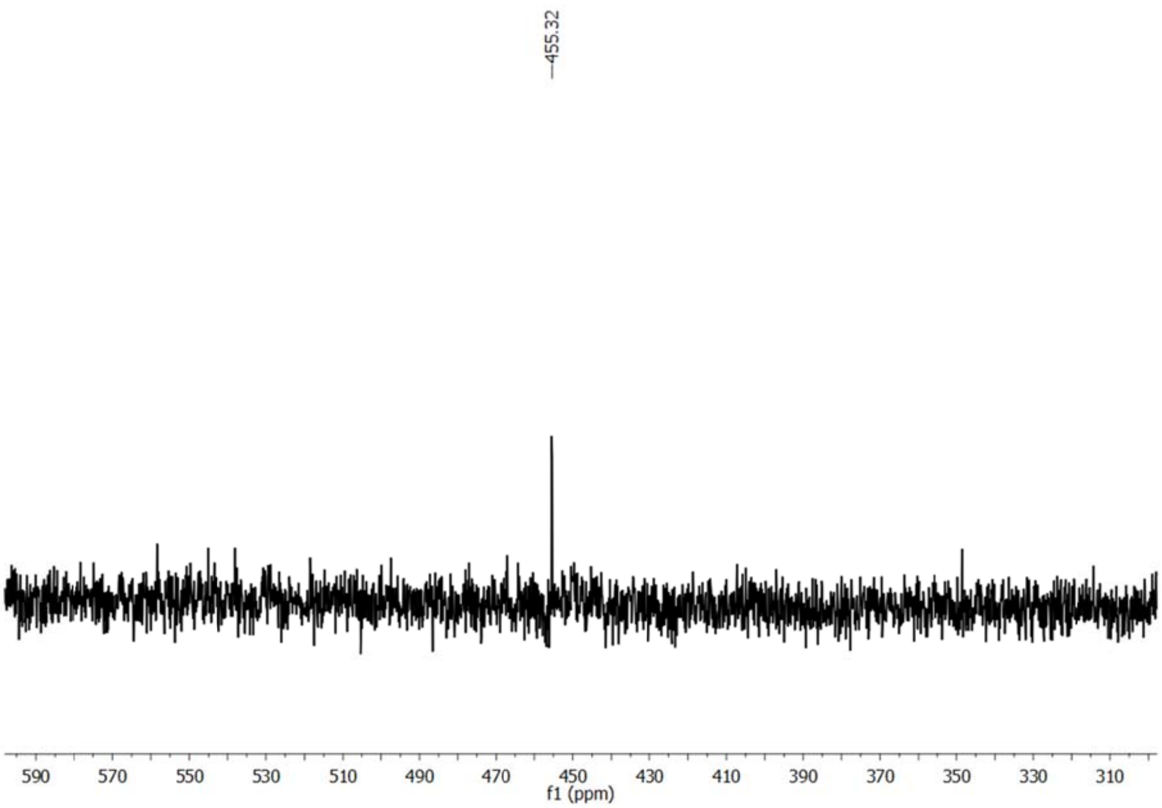

Figure S84. ^{77}Se-NMR of compound **4e**.

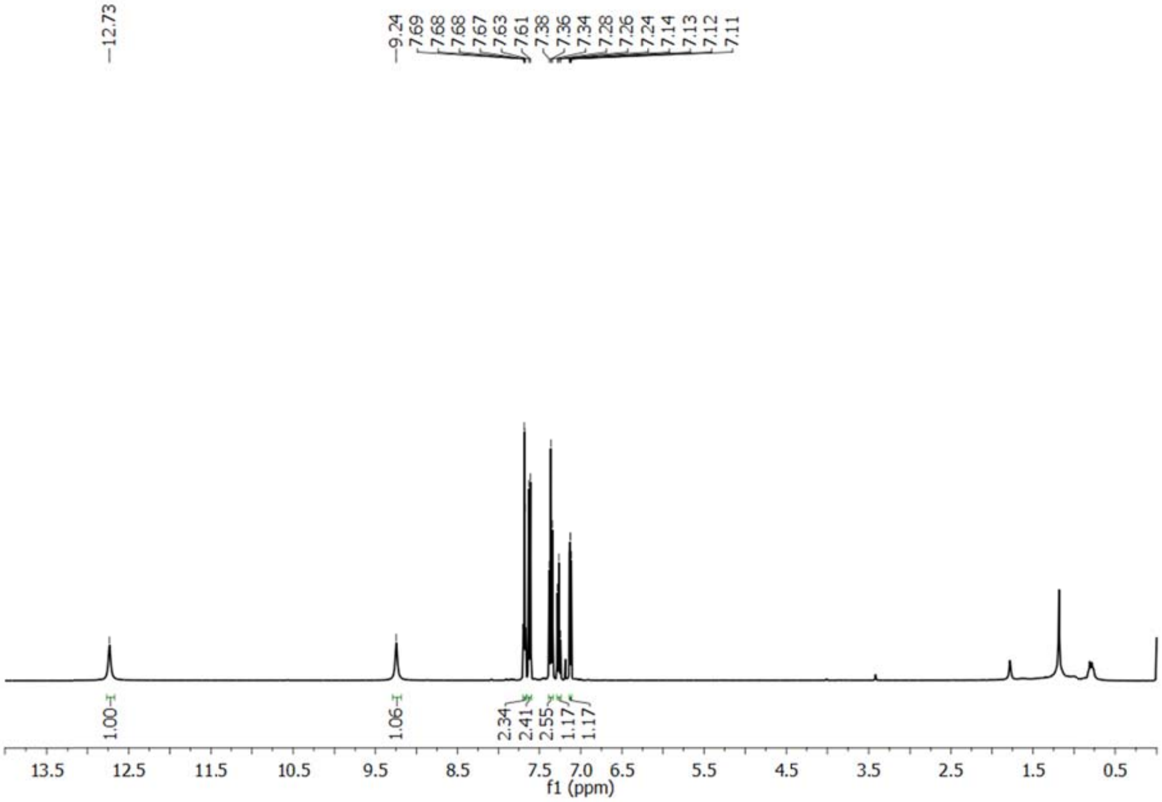

Figure S85. ^{1}H-NMR of compound **5a**.

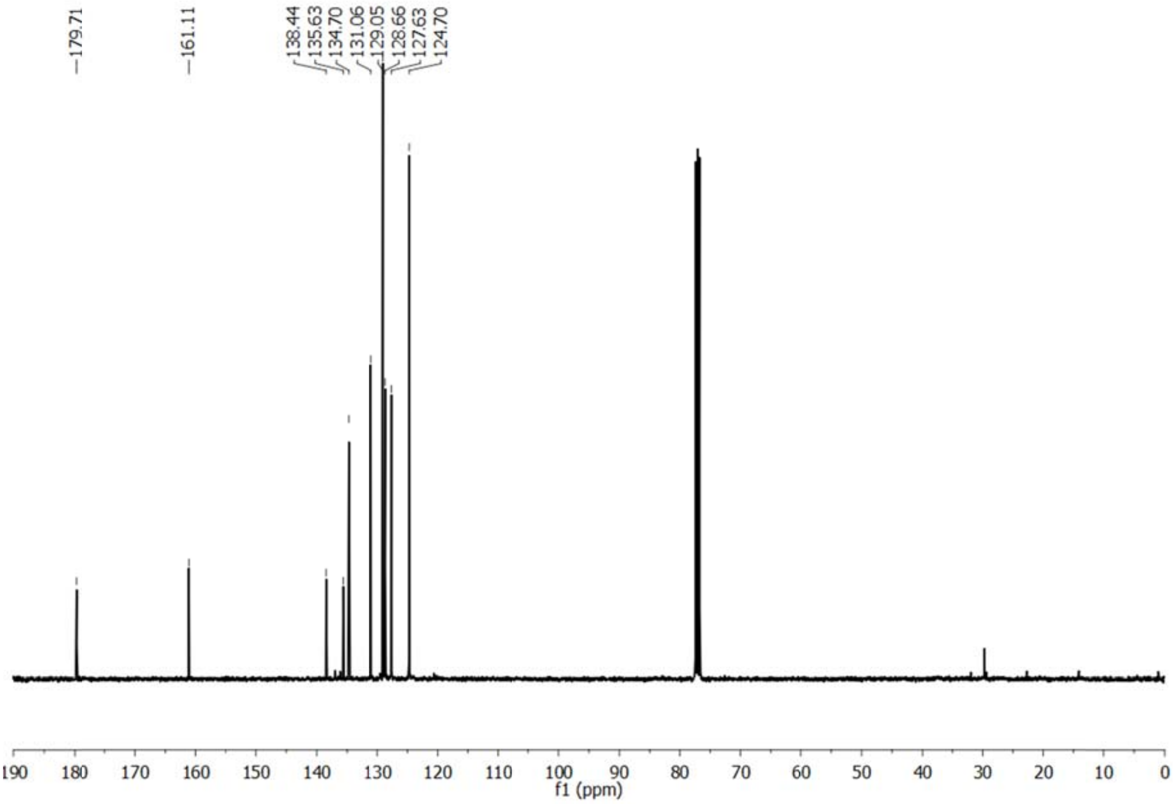

Figure S86. ^{13}C-NMR of compound **5a.**

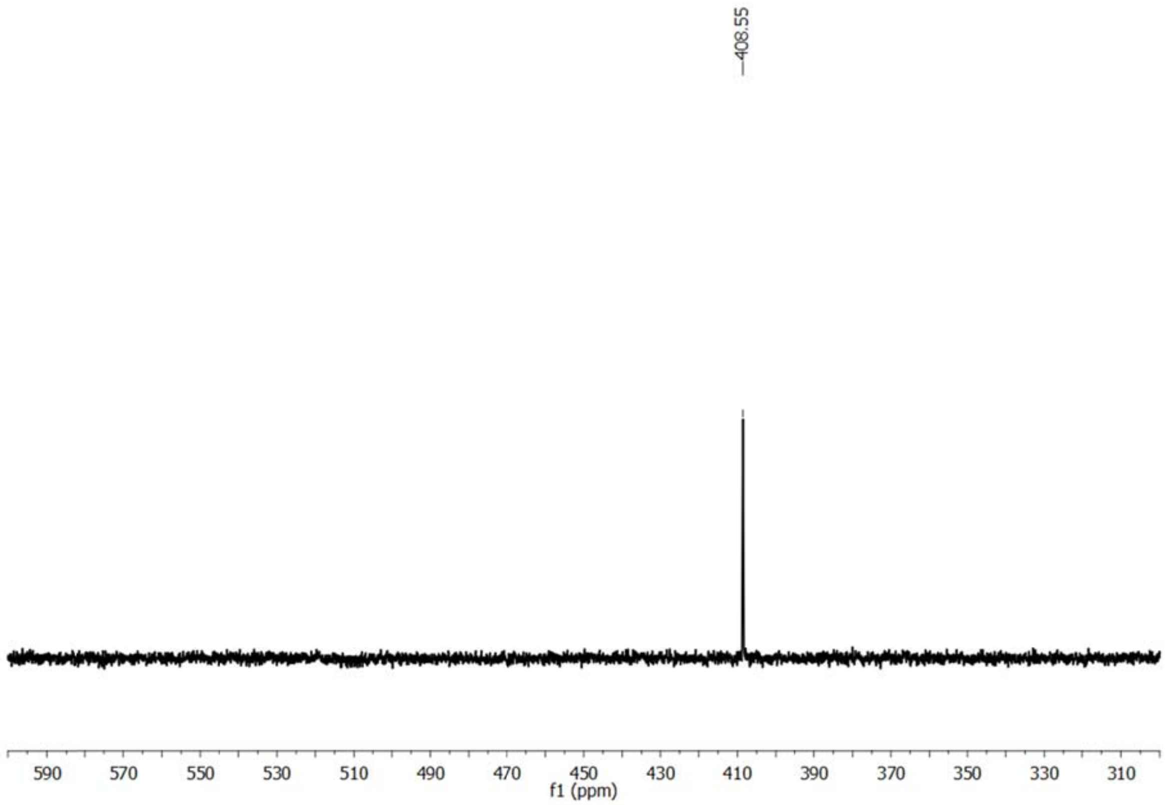

Figure S87. ^{77}Se-NMR of compound **5a**.

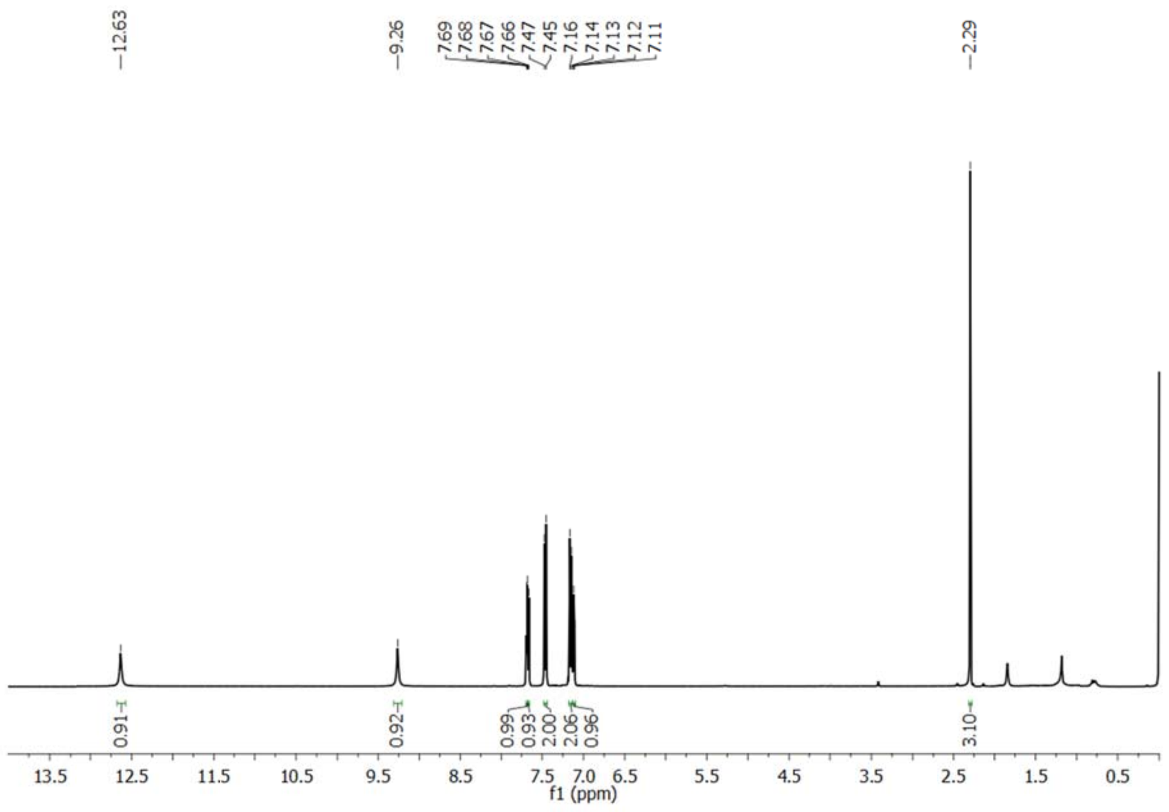

Figure S88. ^{1}H-NMR of compound **5b**.

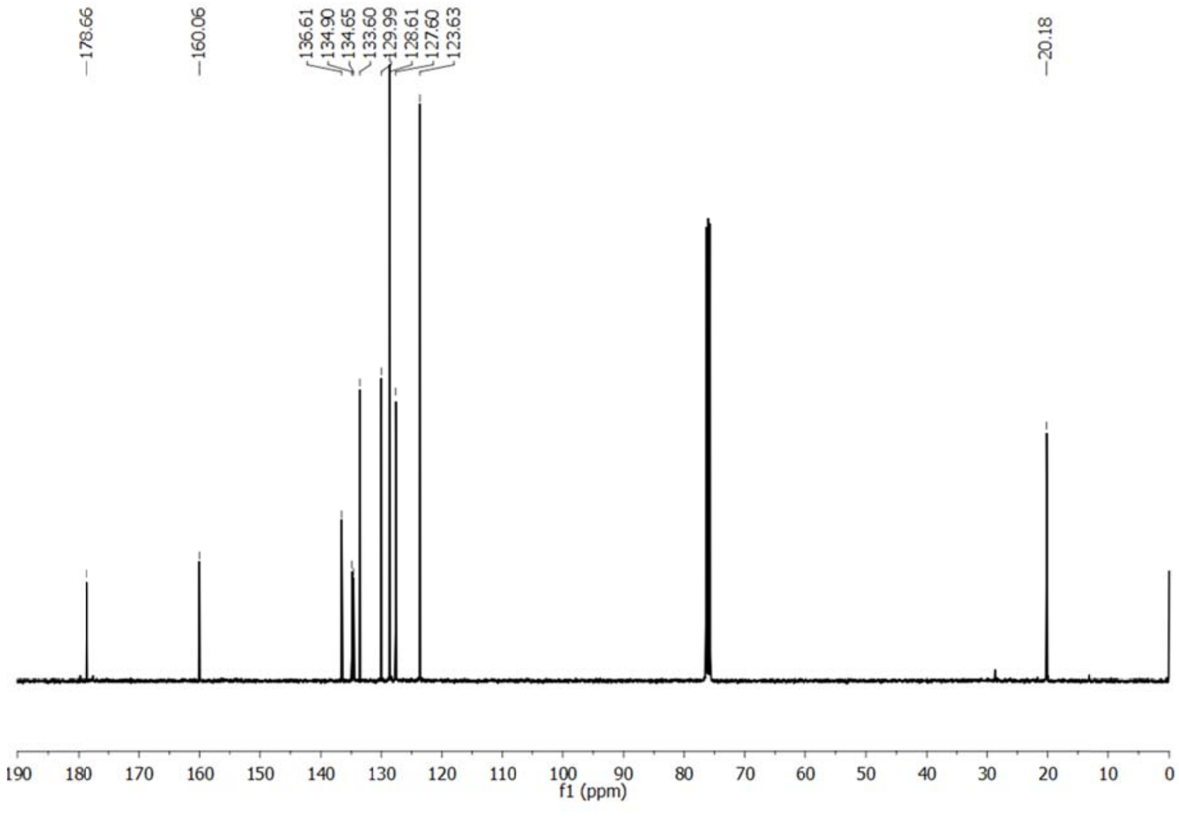

Figure S89. ^{13}C-NMR of compound **5b**.

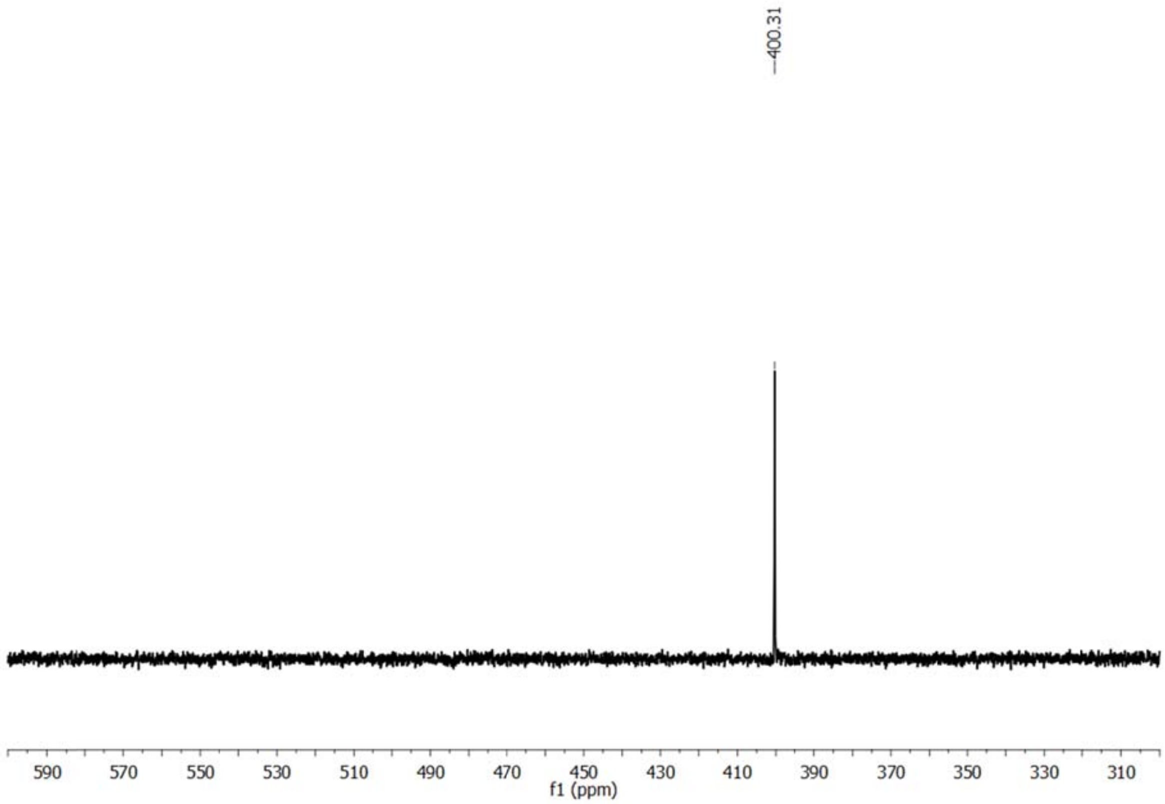

Figure S90. ^{77}Se-NMR of compound **5b**.

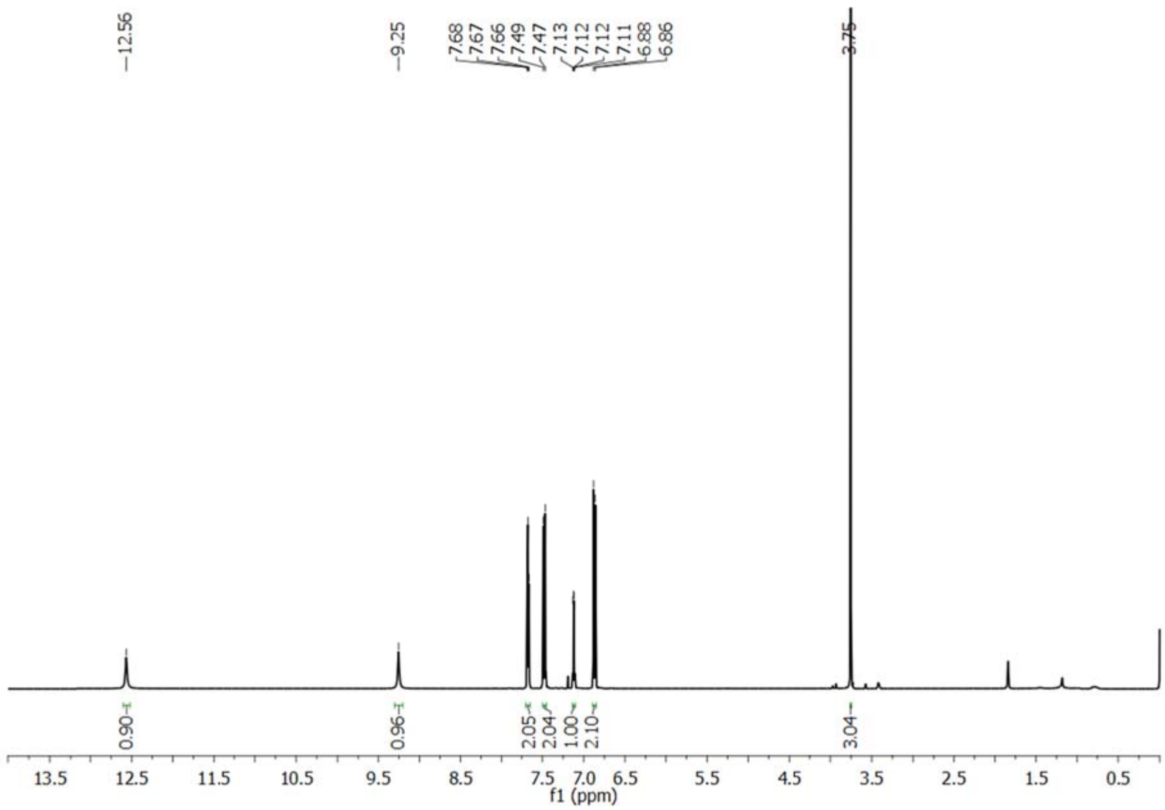

Figure S91. ^{1}H-NMR of compound **5c**.

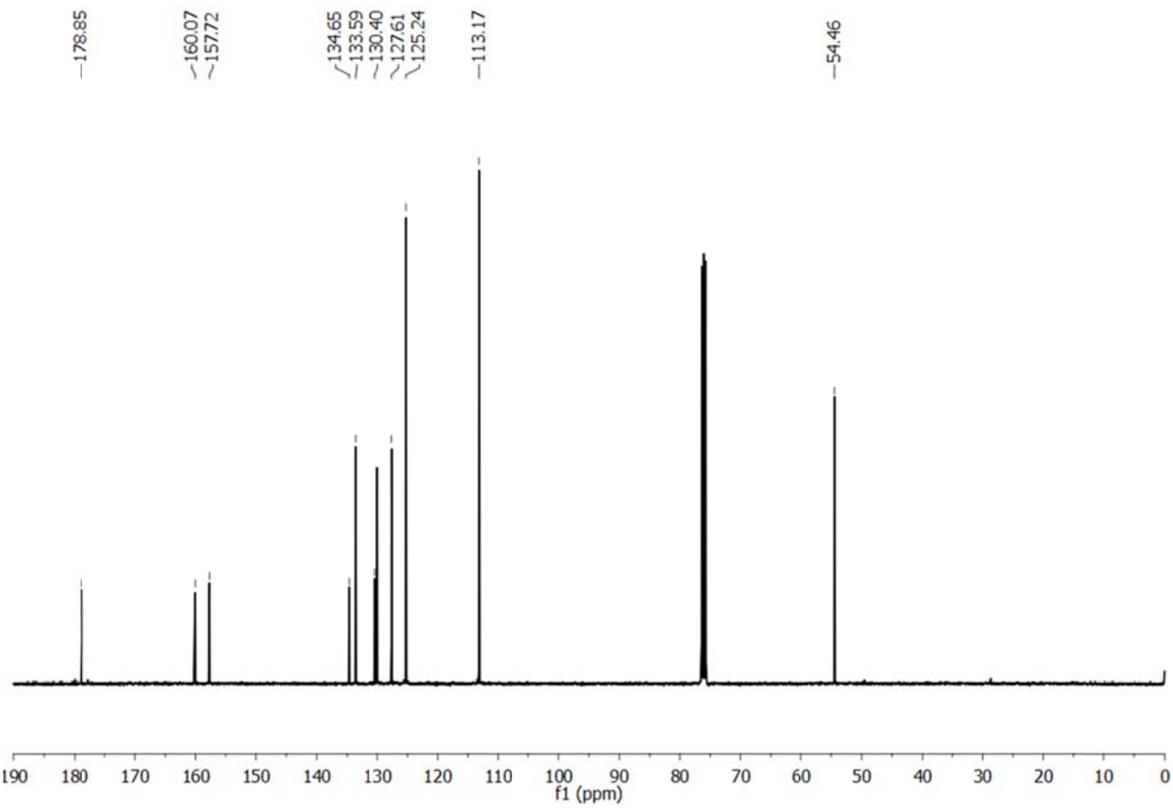

Figure S92. ^{13}C-NMR of compound **5c.**

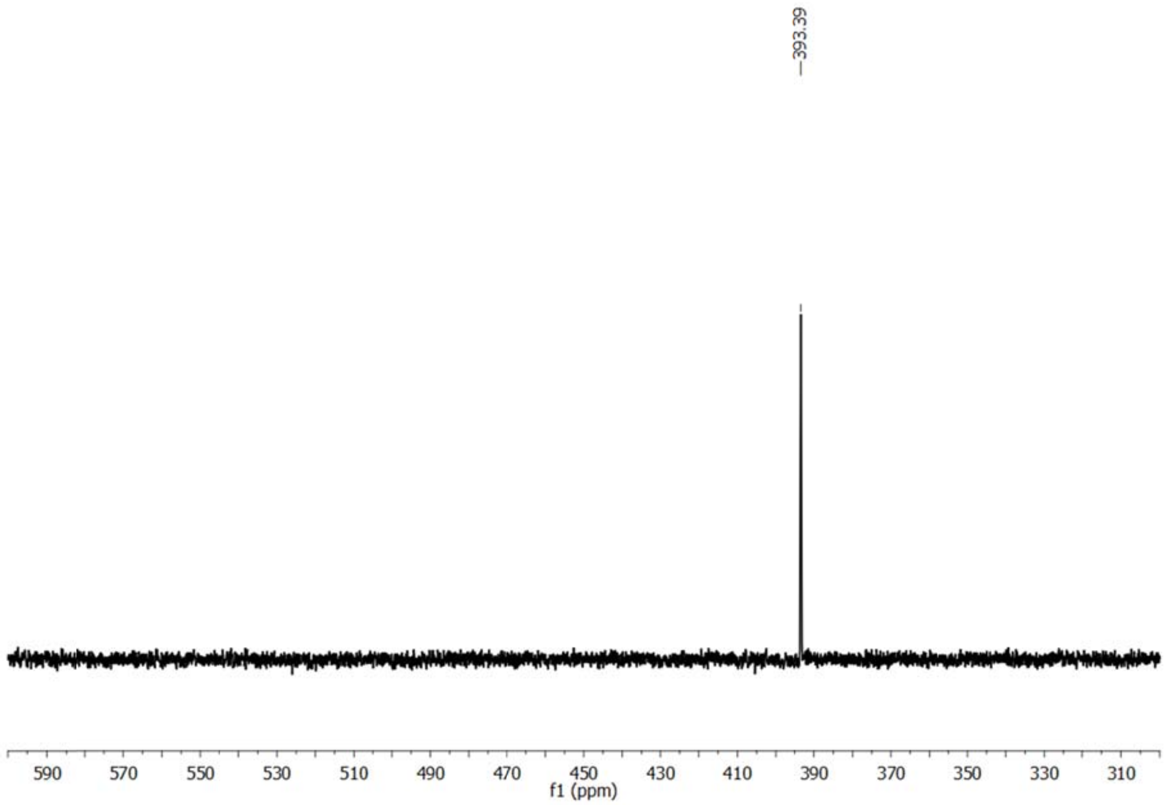

Figure S93. ^{77}Se-NMR of compound **5c**.

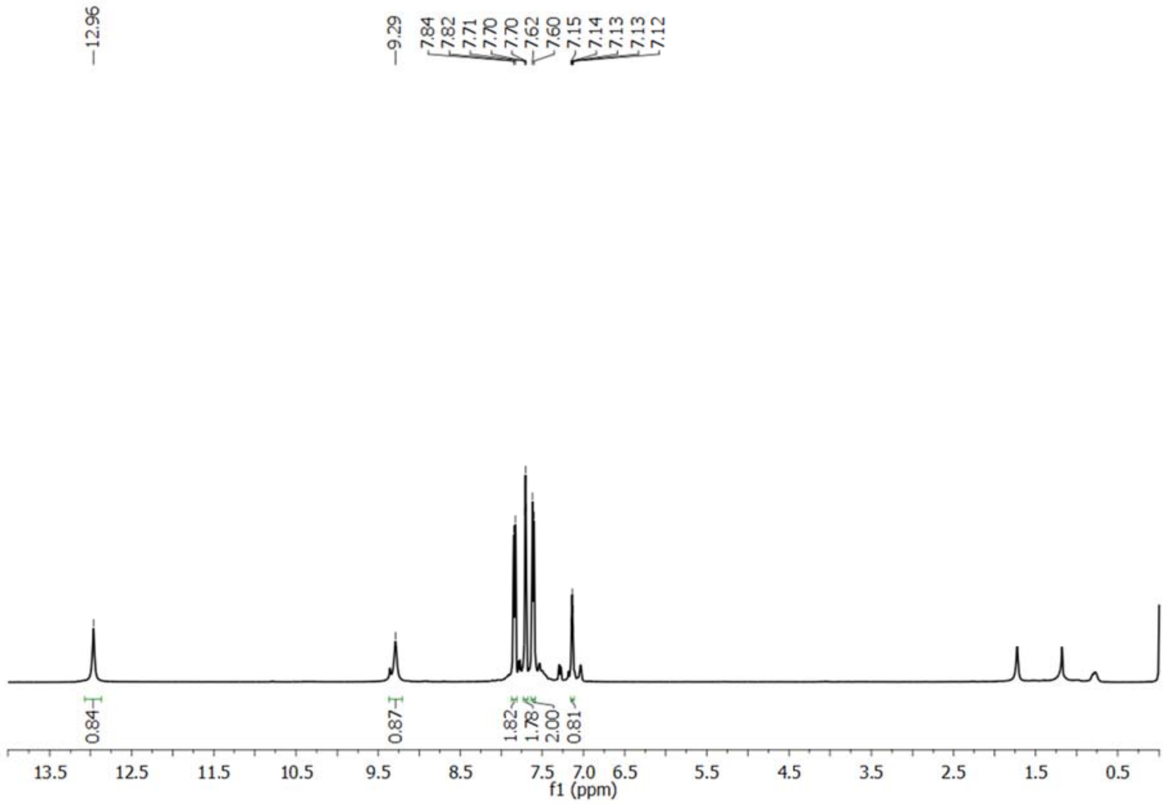

Figure S94. ^{1}H-NMR of compound **5d.**

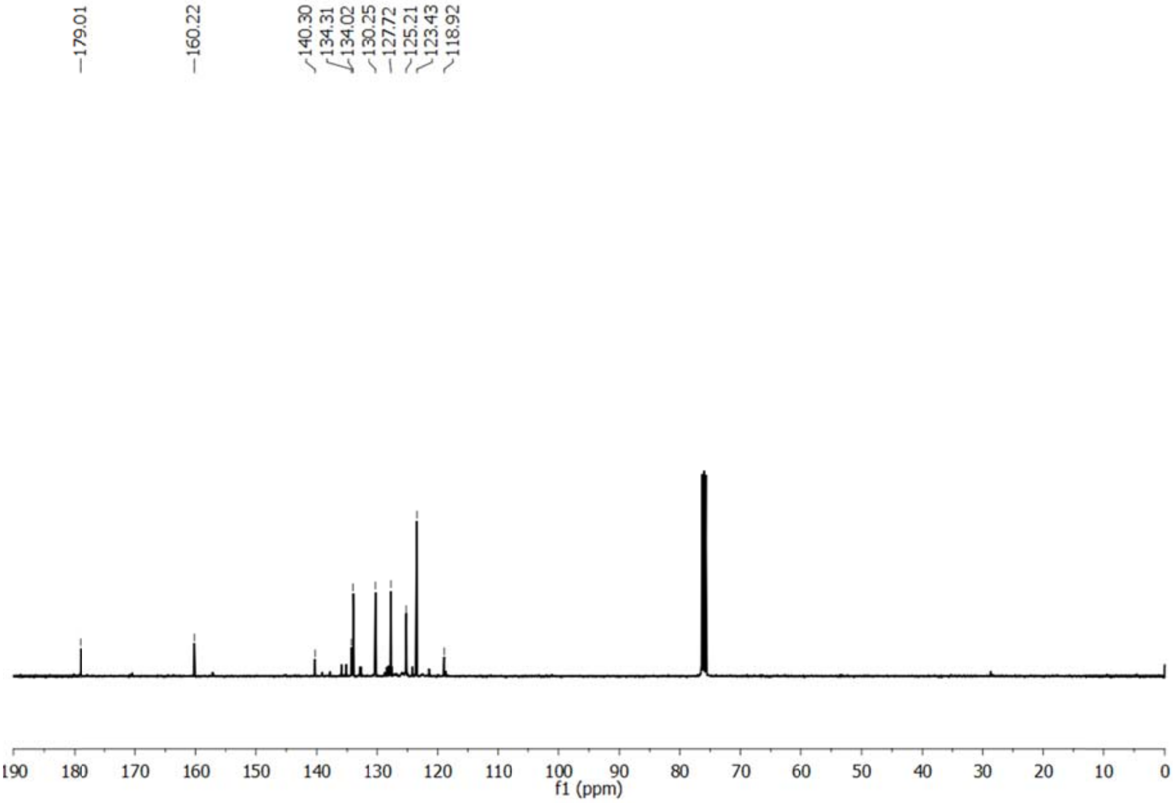

Figure S95. ^{13}C-NMR of compound **5d**.

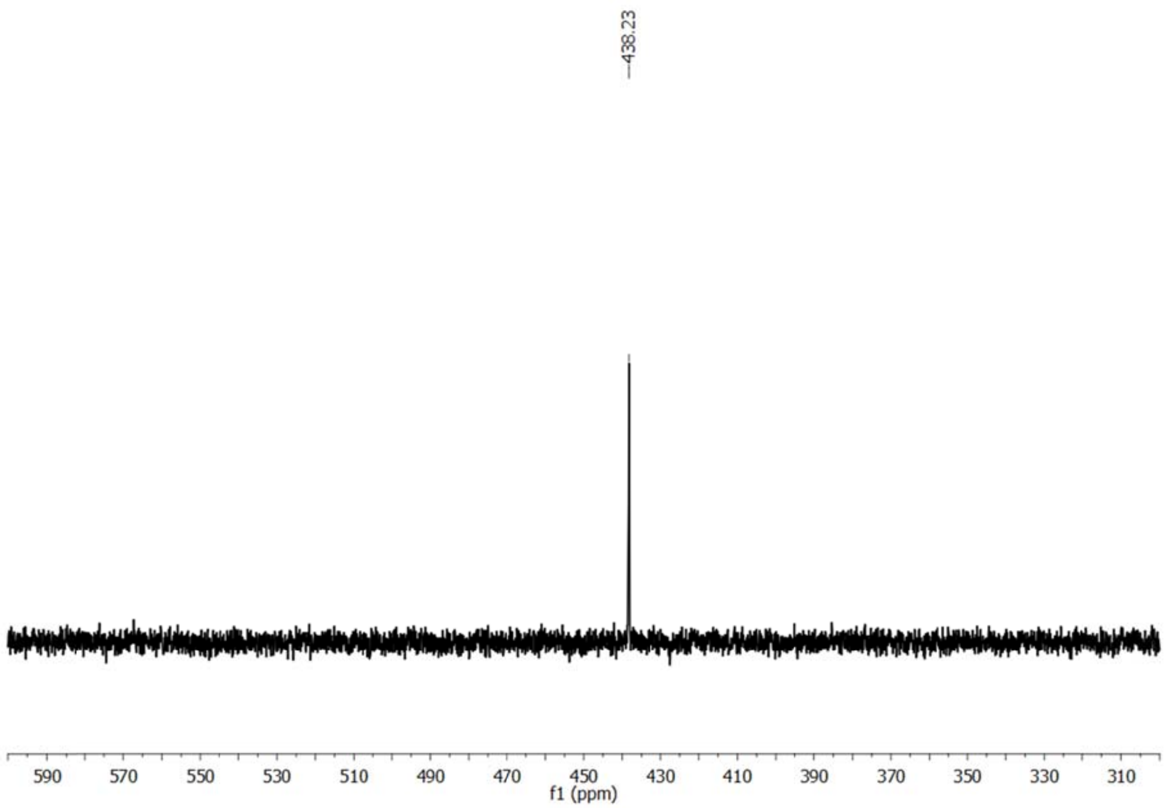

Figure S96. ^{77}Se-NMR of compound **5d**.

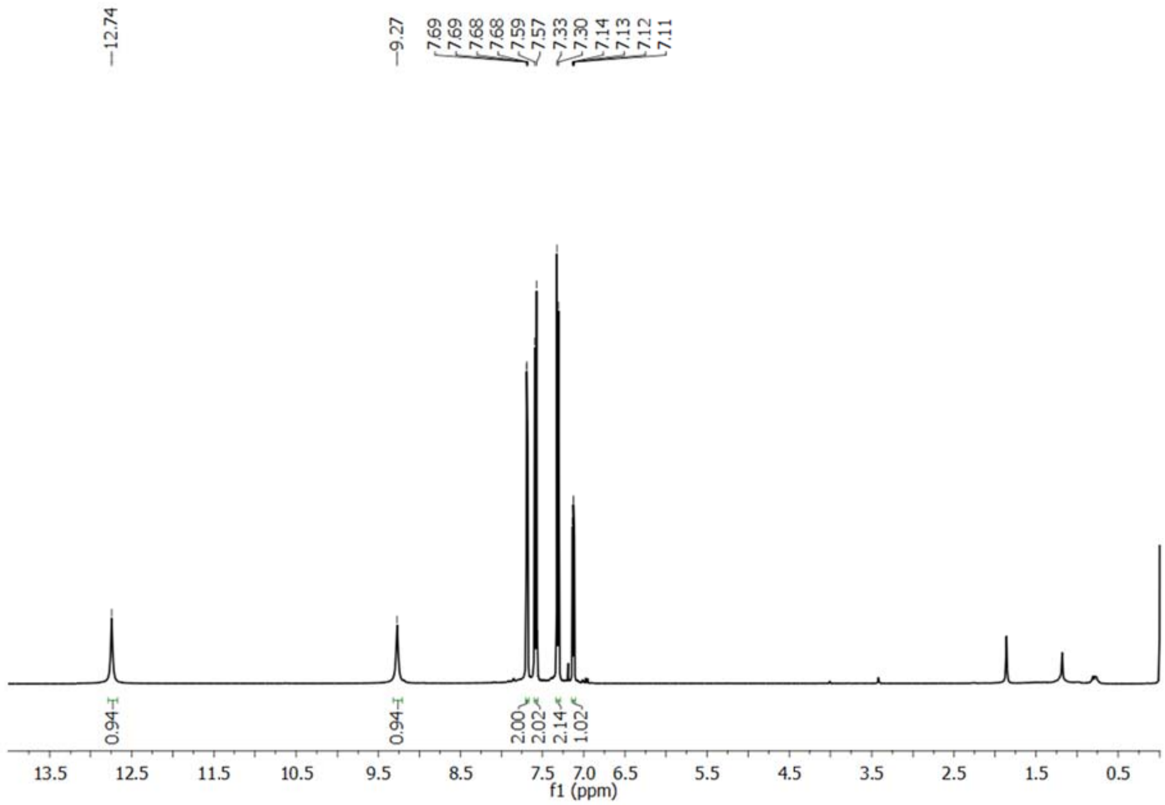

Figure S97. ^{1}H-NMR of compound **5e**.

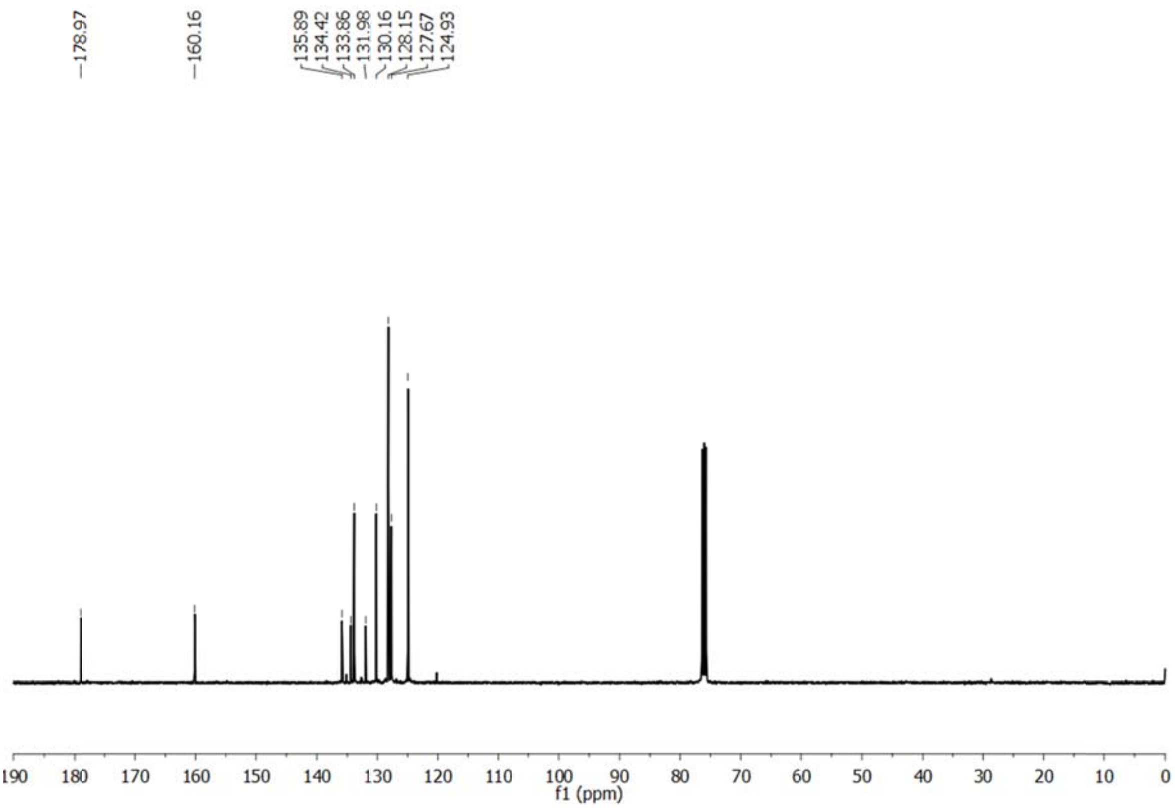

Figure S98. ^{13}C-NMR of compound **5e.**

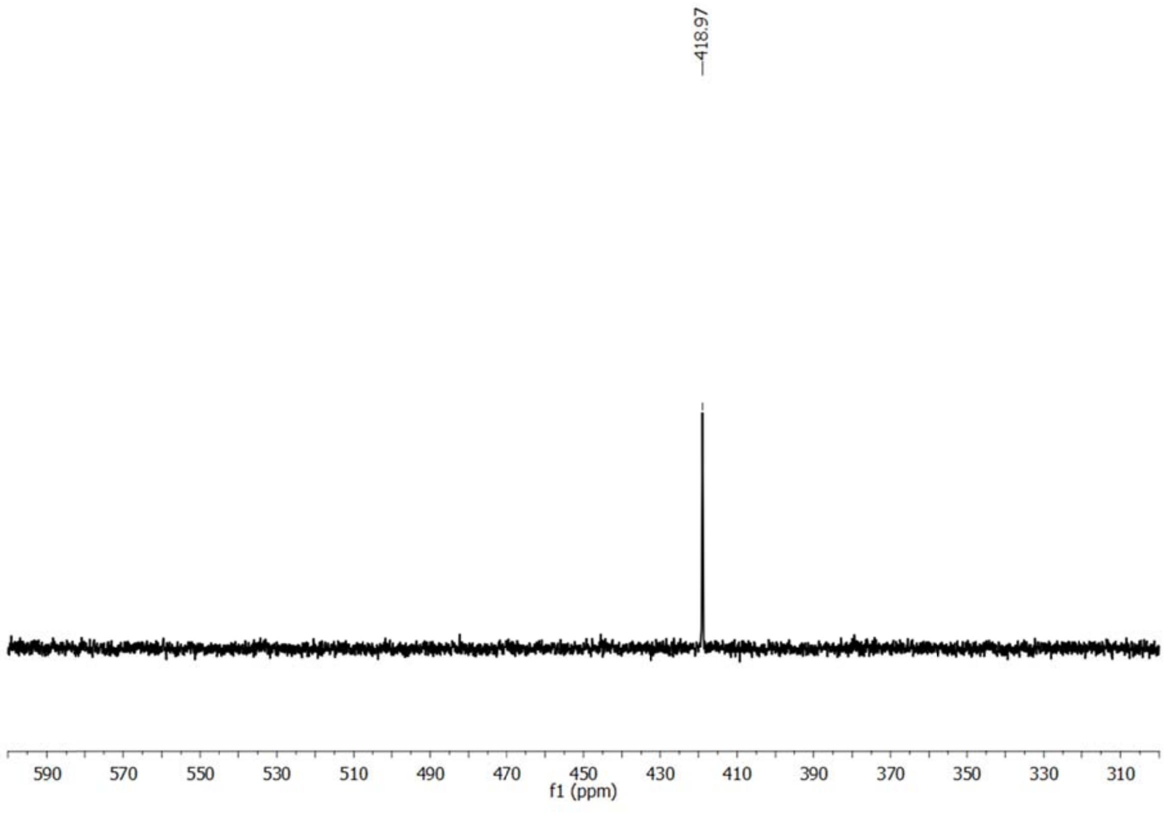

Figure S99. ^{77}Se-NMR of compound **5e**.

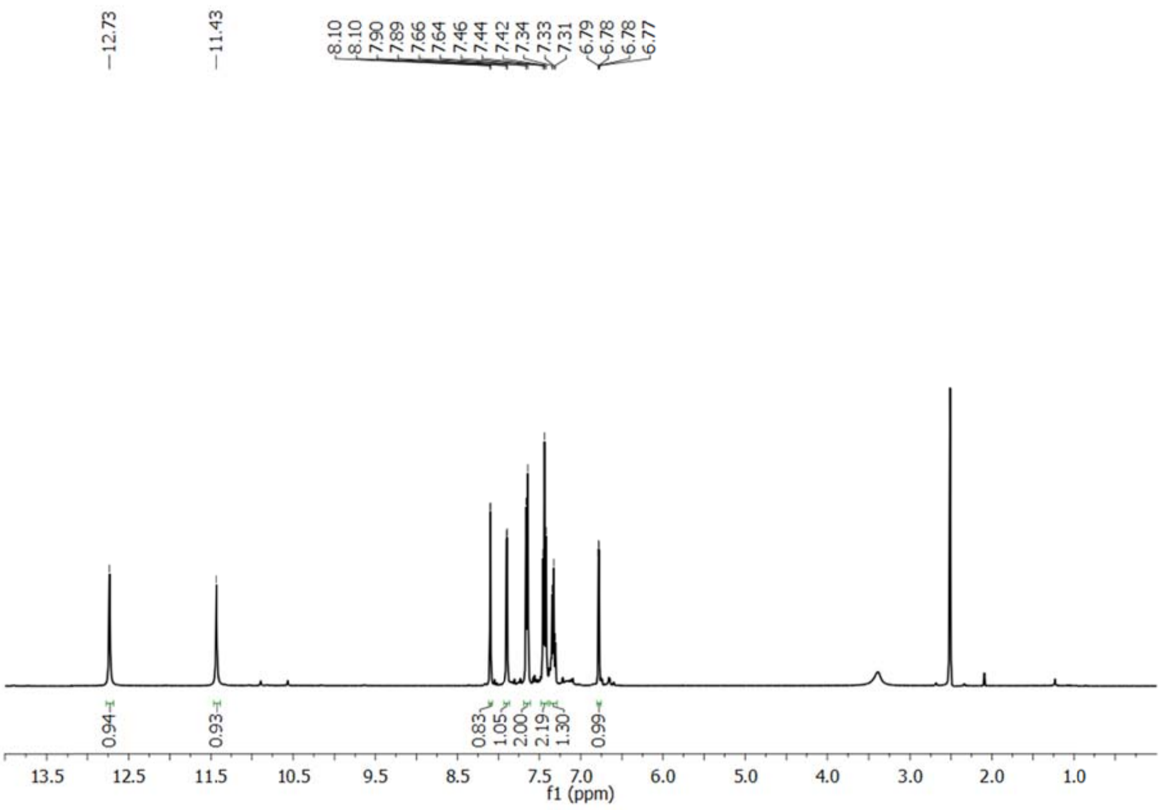

Figure S100. ^{1}H-NMR of compound **6a**.

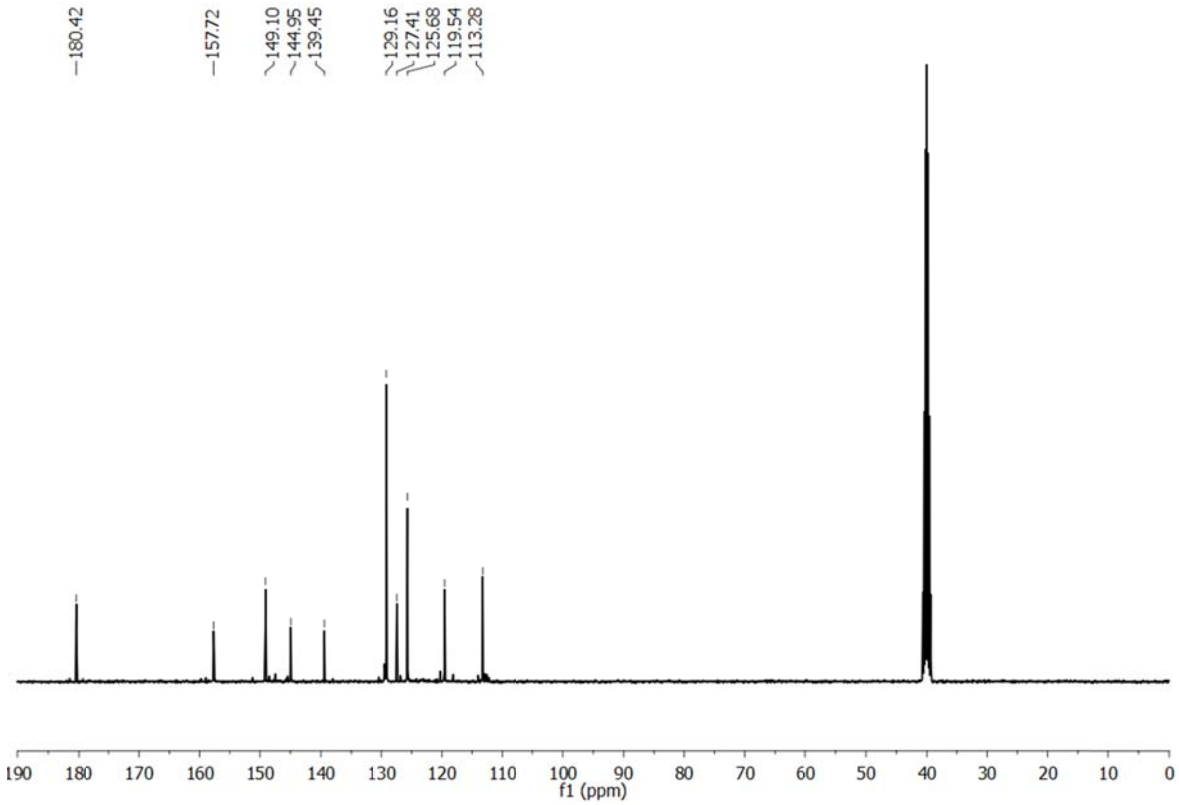

Figure S101. ^{13}C-NMR of compound **6a**.

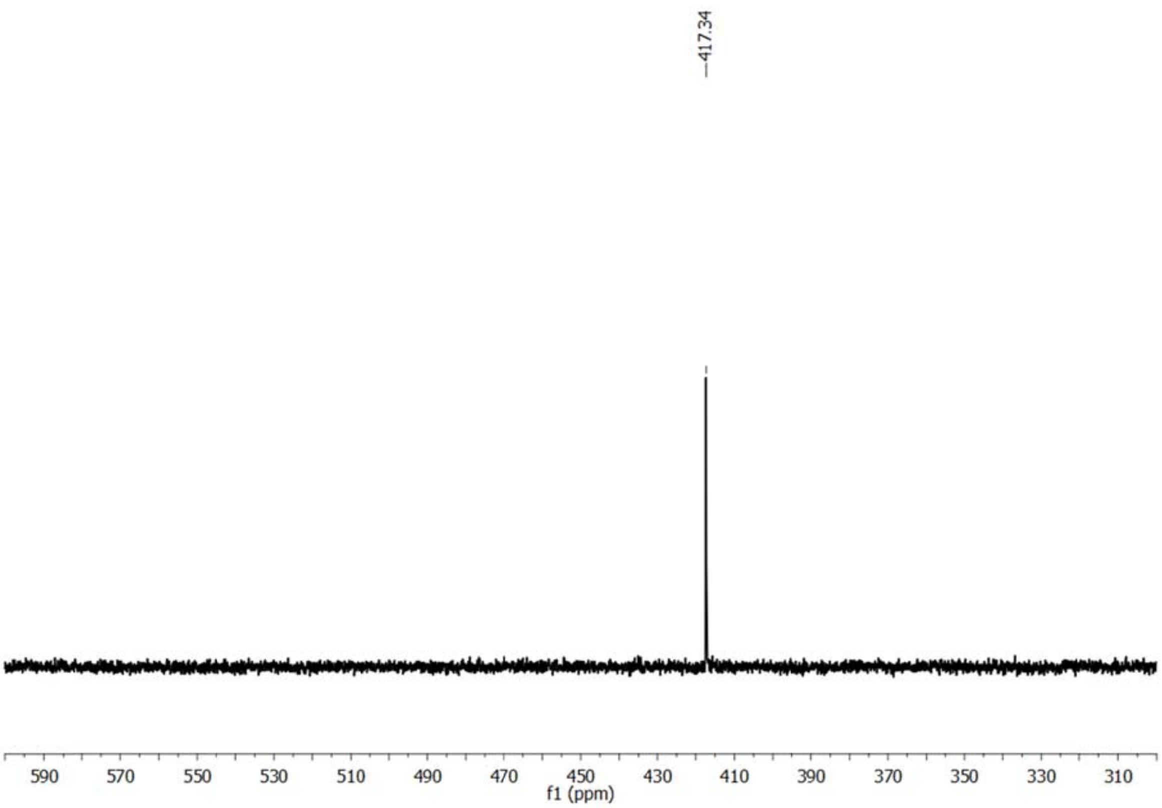

Figure S102. ^{77}Se-NMR of compound **6a**.

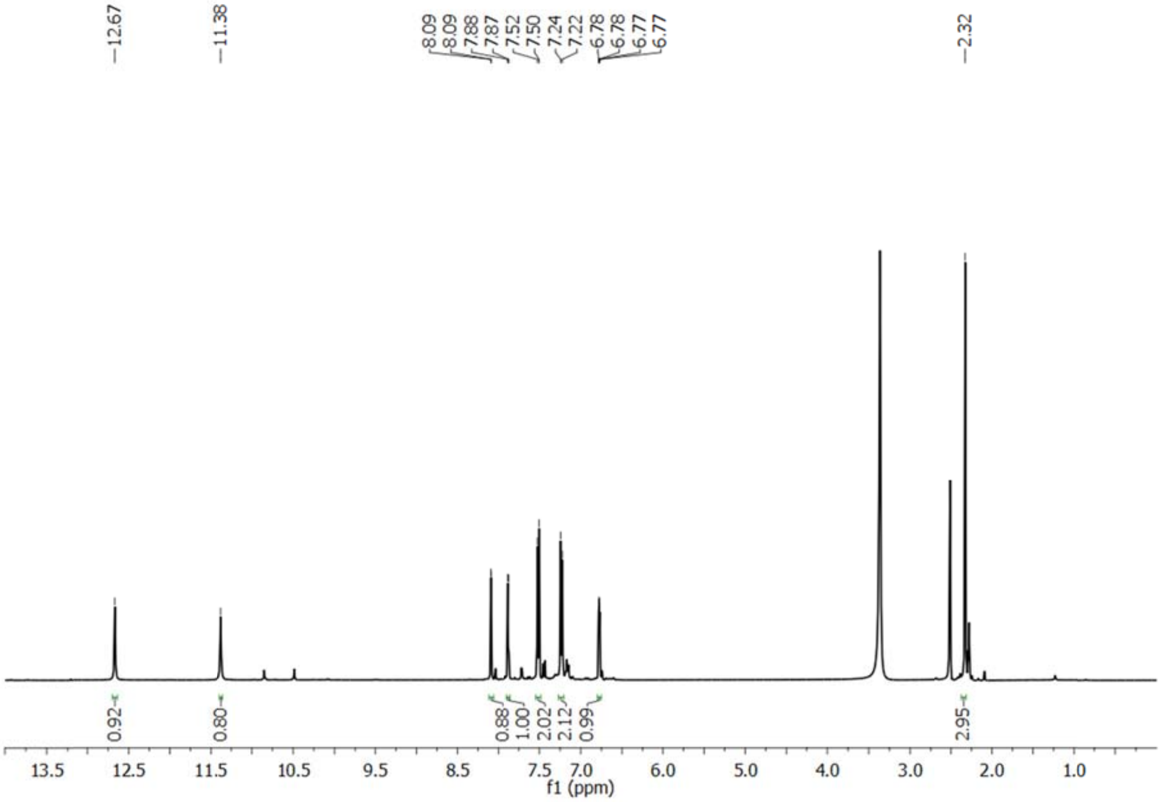

Figure S103. ^{1}H-NMR of compound **6b**.

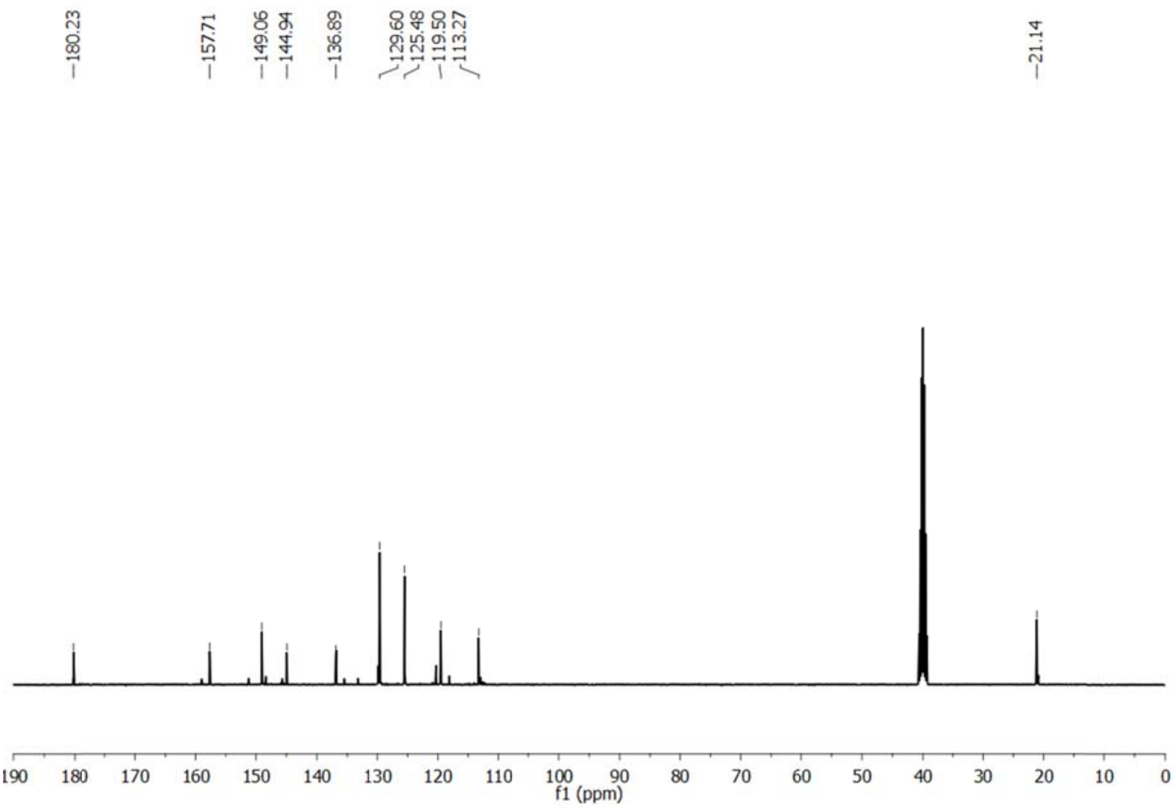

Figure S104. ^{13}C-NMR of compound **6b**.

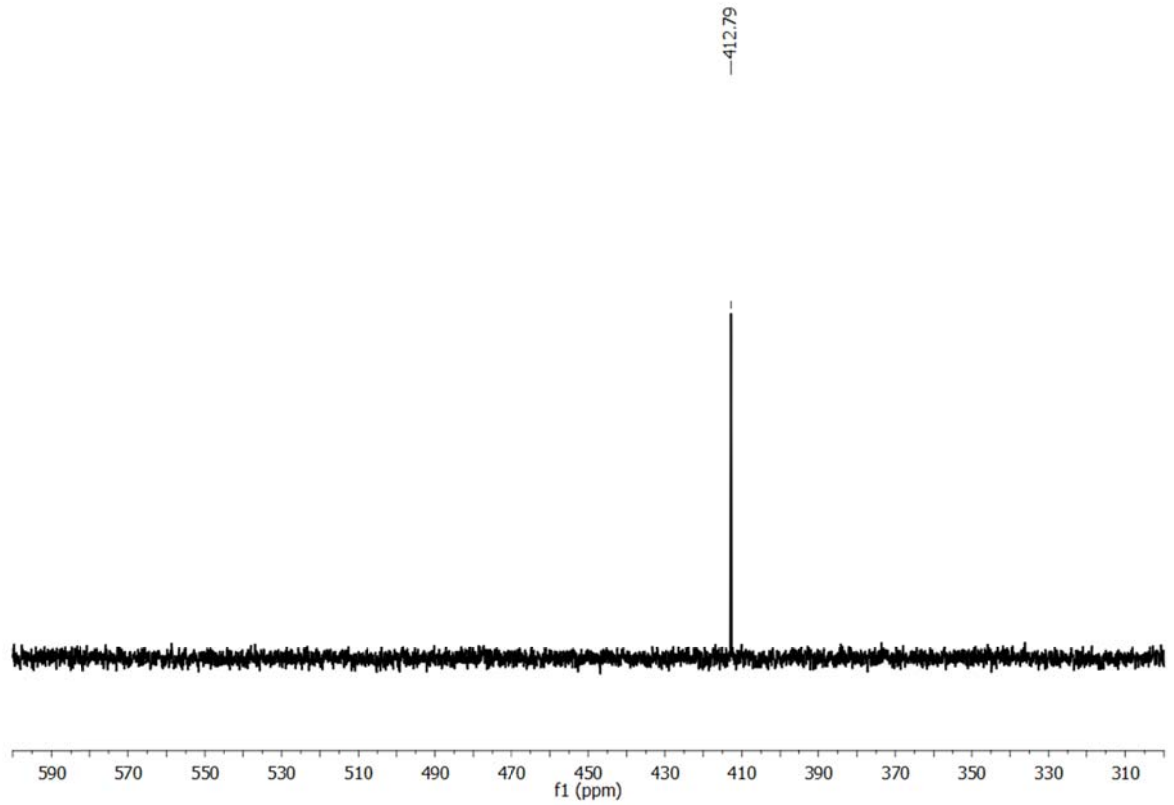

Figure S105. ^{77}Se-NMR of compound **6b**.

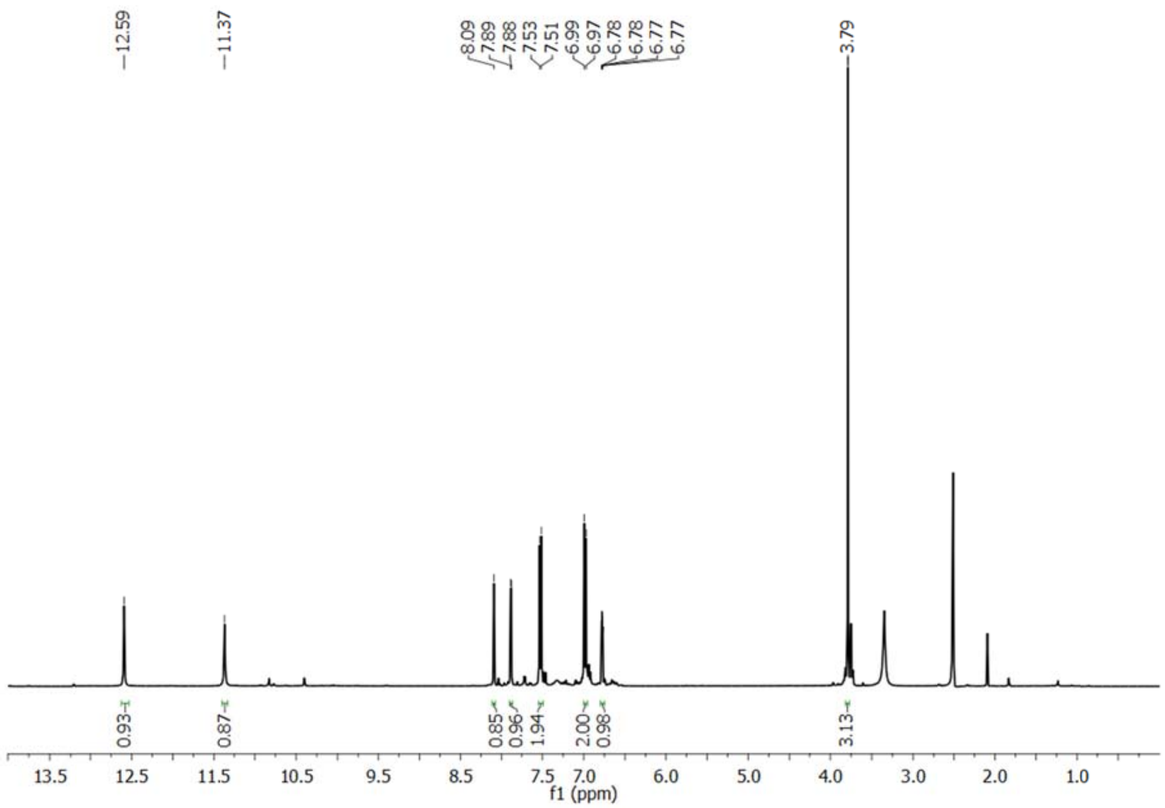

Figure S106. ^{1}H-NMR of compound **6c**.

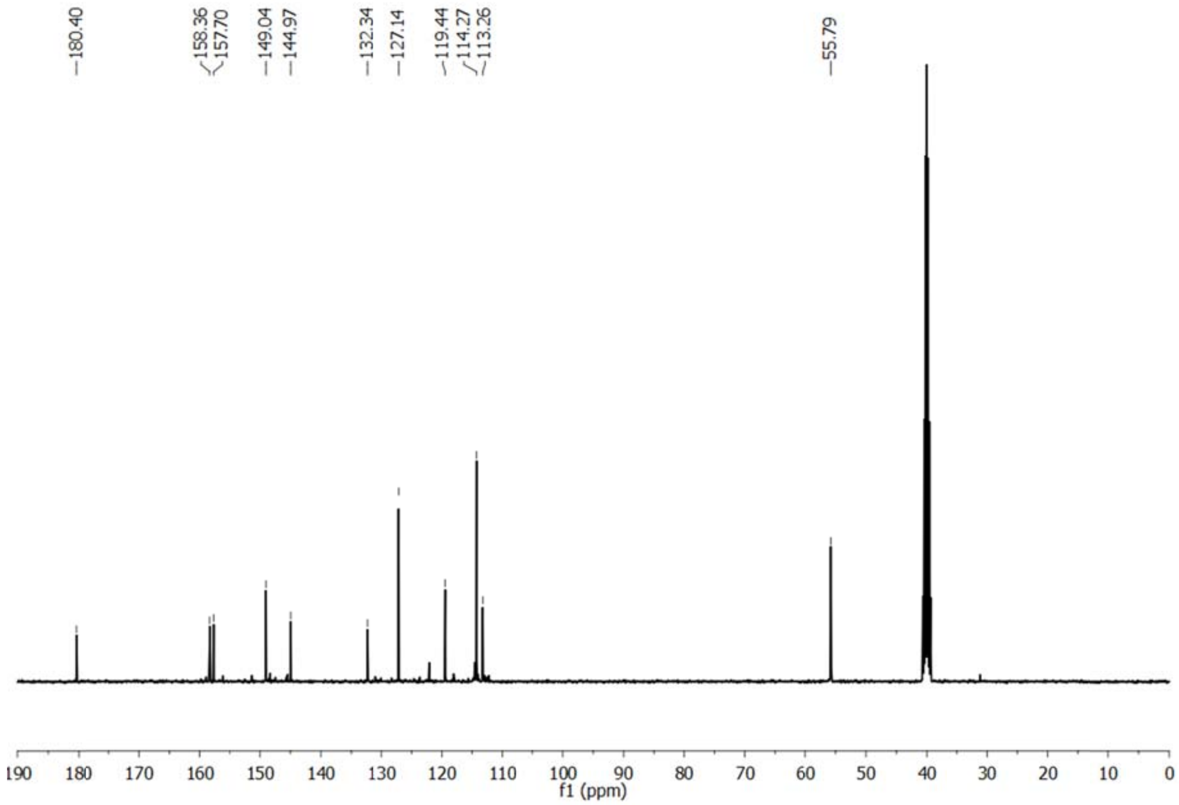

Figure S107. ^{13}C-NMR of compound **6c**.

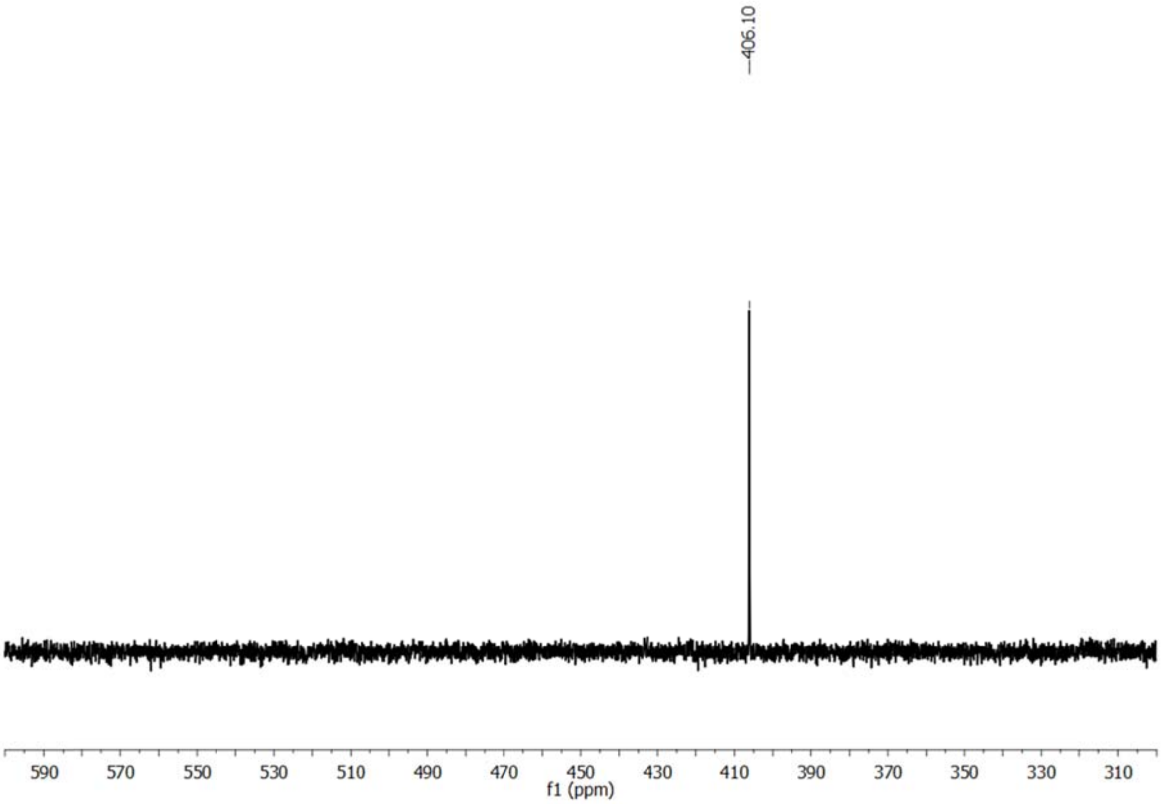

Figure S108. ^{77}Se-NMR of compound **6c**.

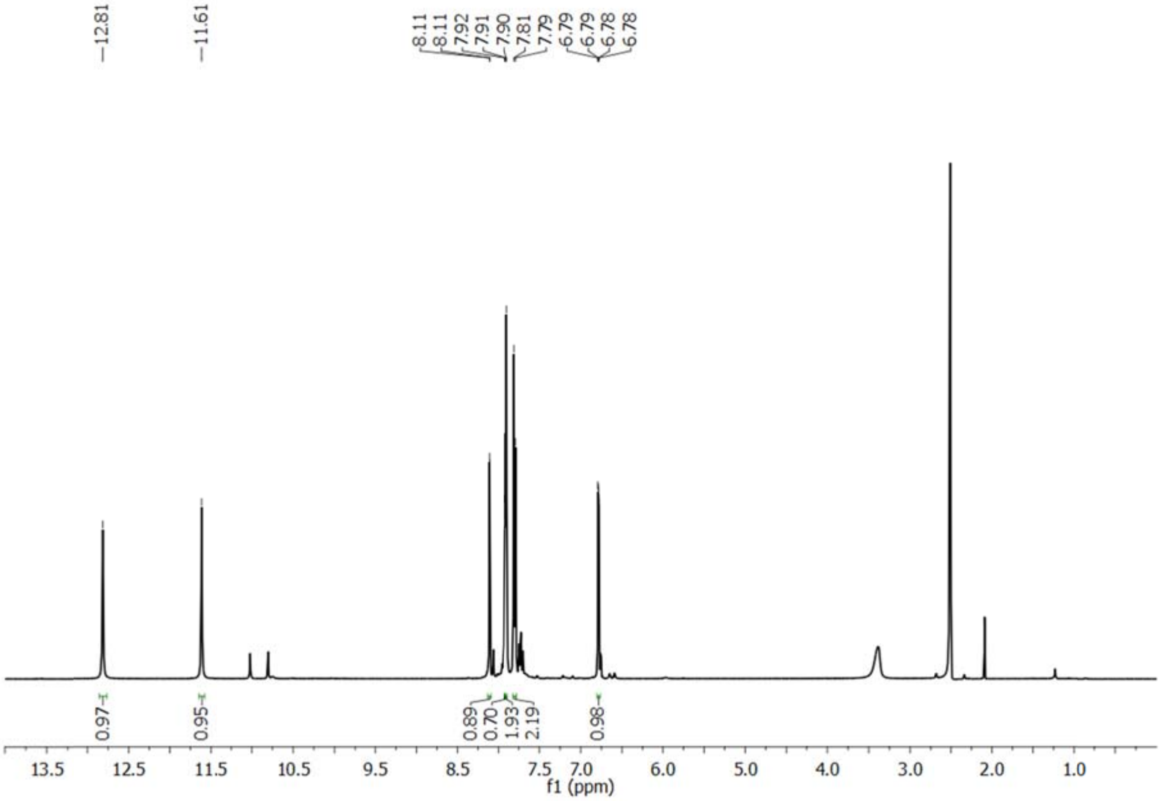

Figure S109. ^{1}H-NMR of compound **6d**.

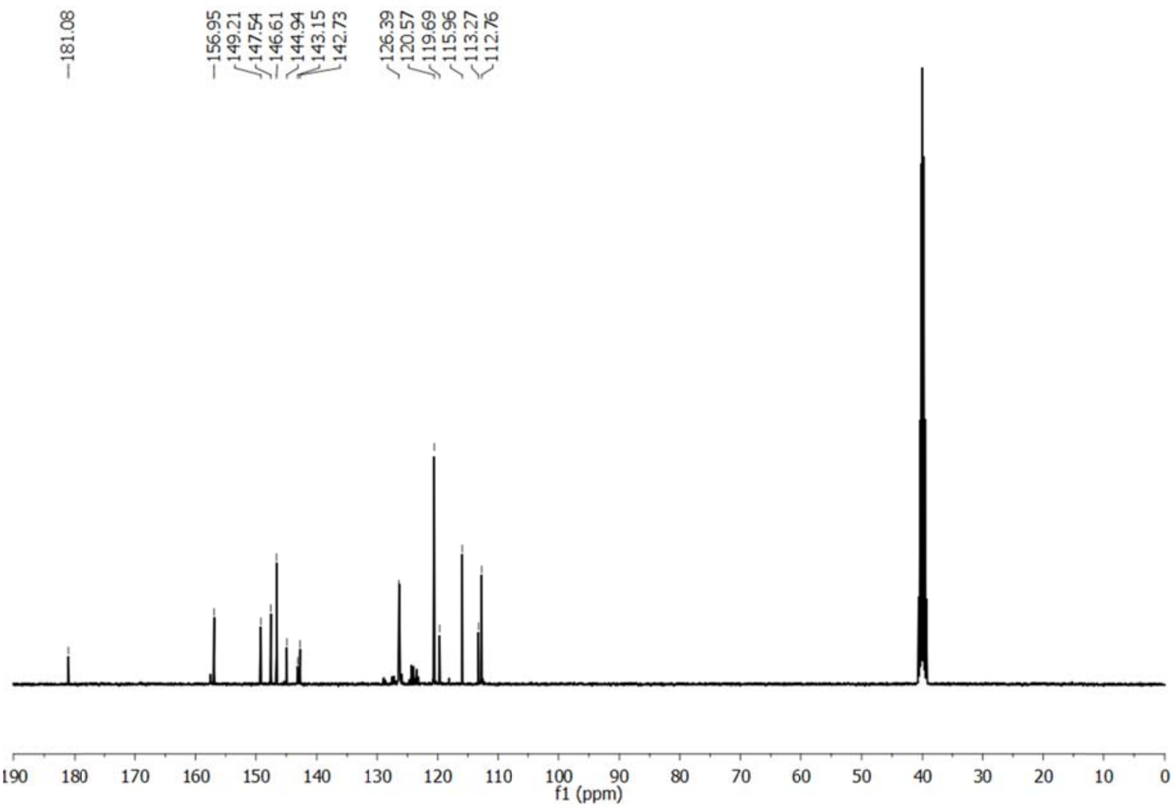

Figure S110. ^{13}C-NMR of compound **6d**.

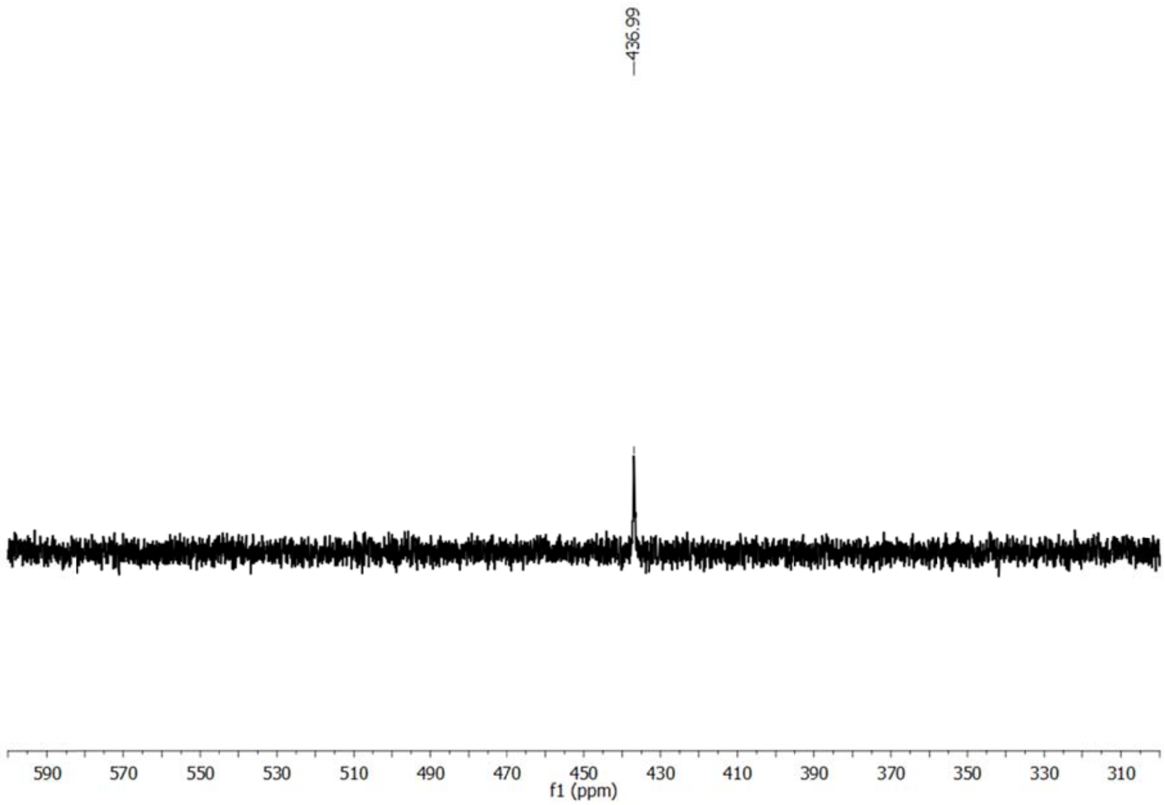

Figure S111. ^{77}Se-NMR of compound **6d**.

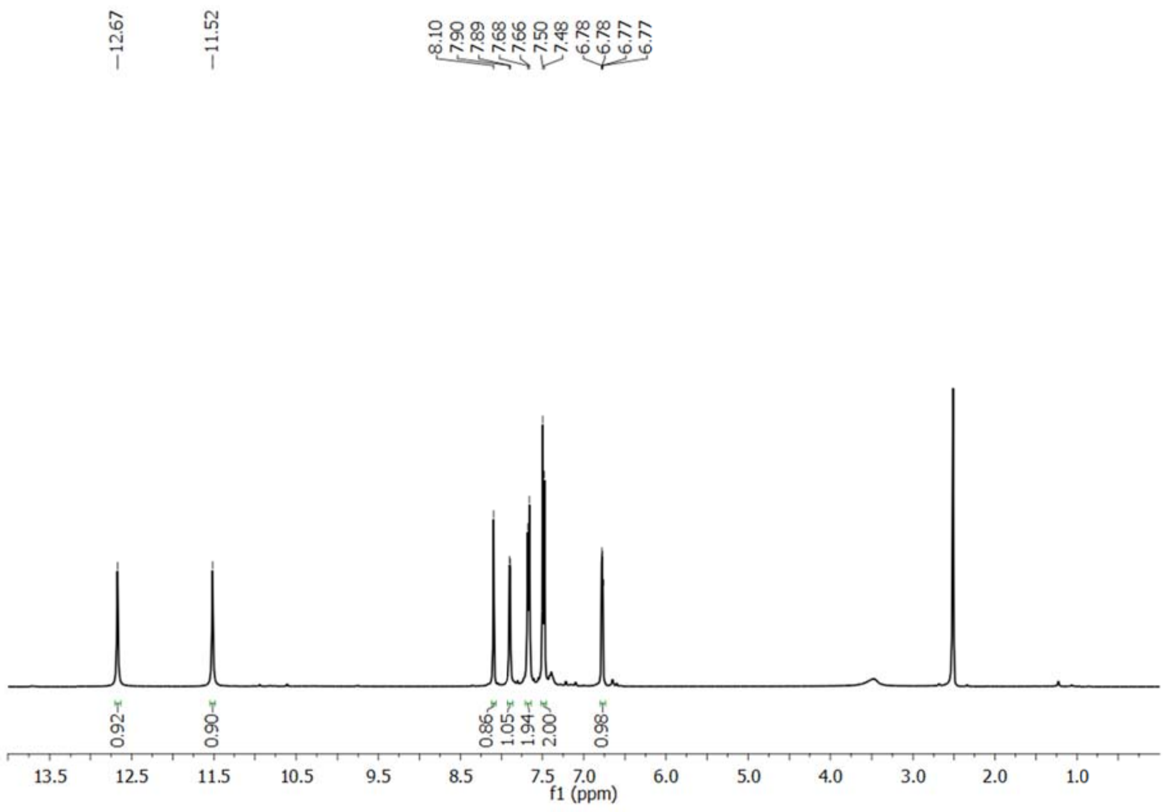

Figure S112. ^{1}H-NMR of compound **6e**.

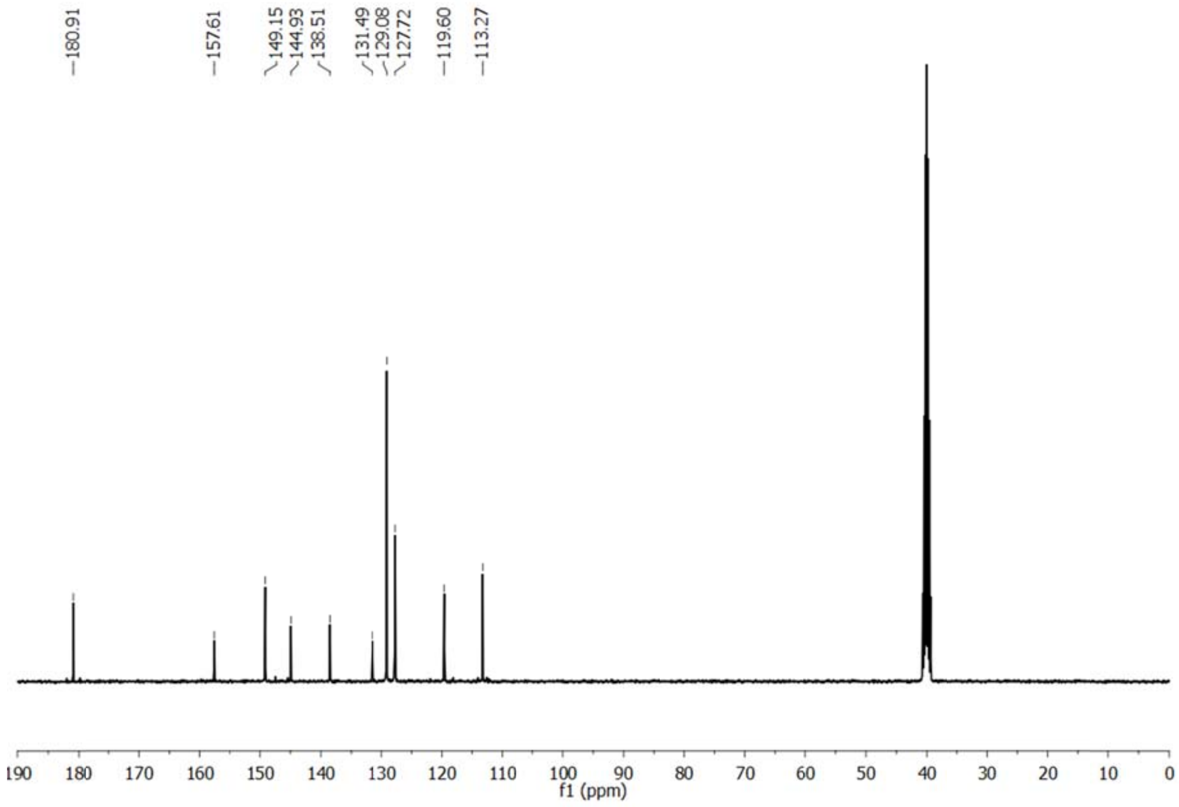

Figure S113. ^{13}C-NMR of compound **6e**.

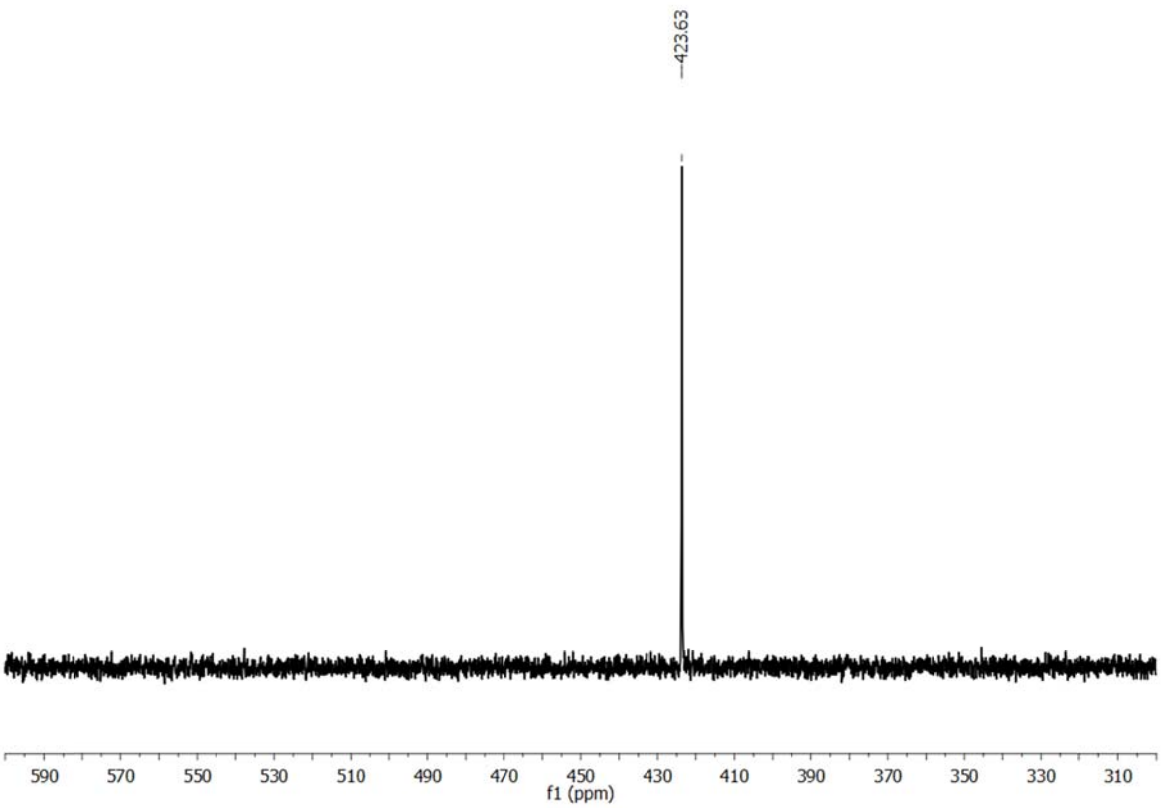

Figure S114. ^{77}Se-NMR of compound **6e**.

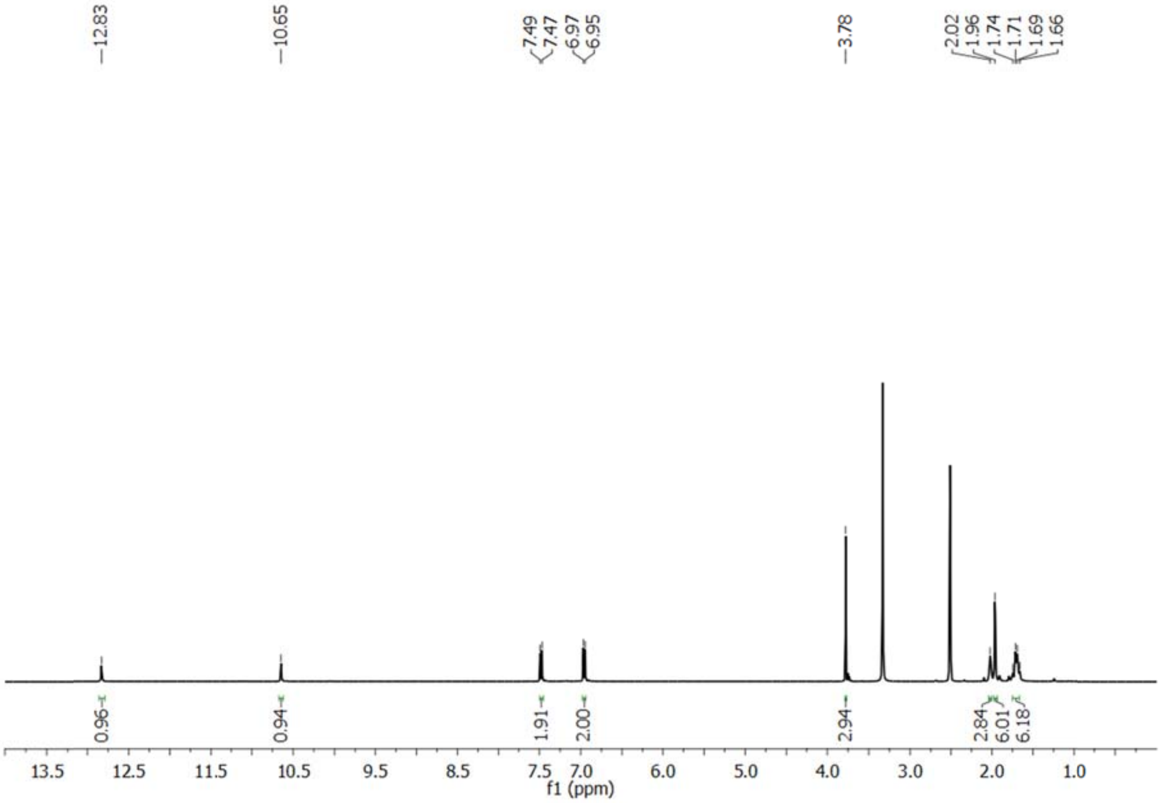

Figure S115. ^{1}H-NMR of compound **7c**.

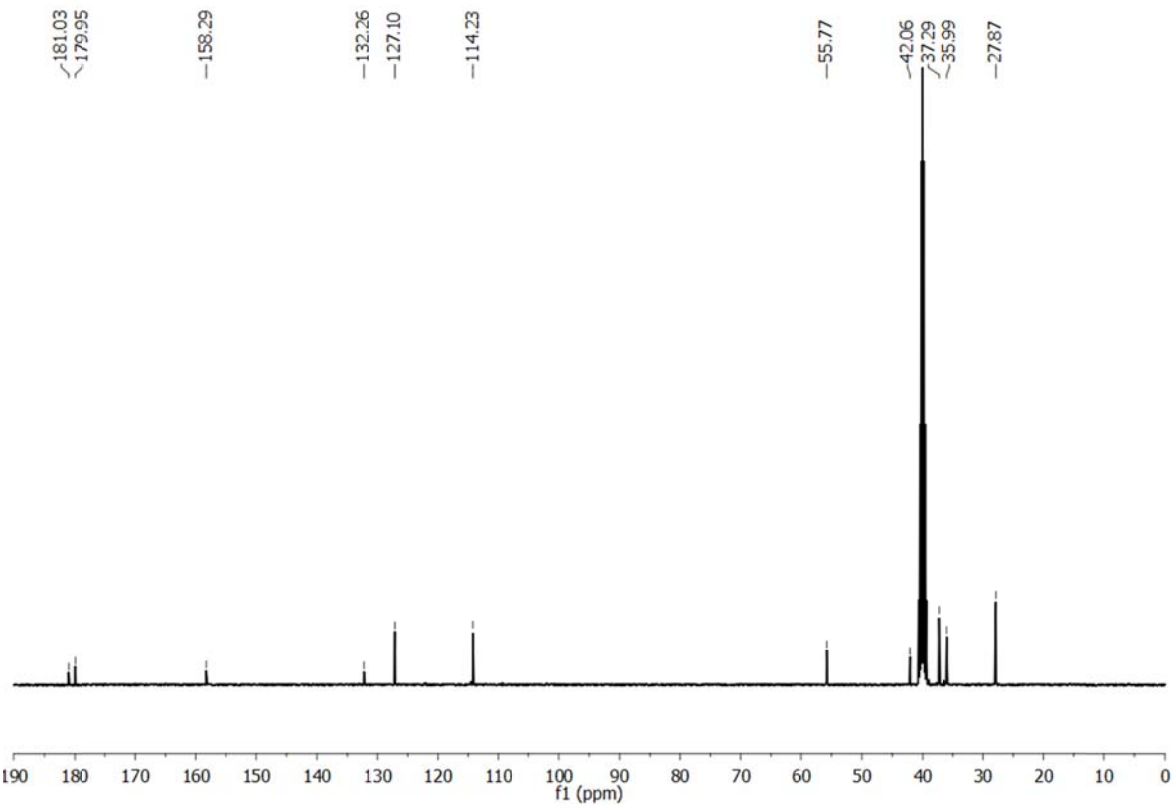

Figure S116. ^{13}C-NMR of compound **7c**.

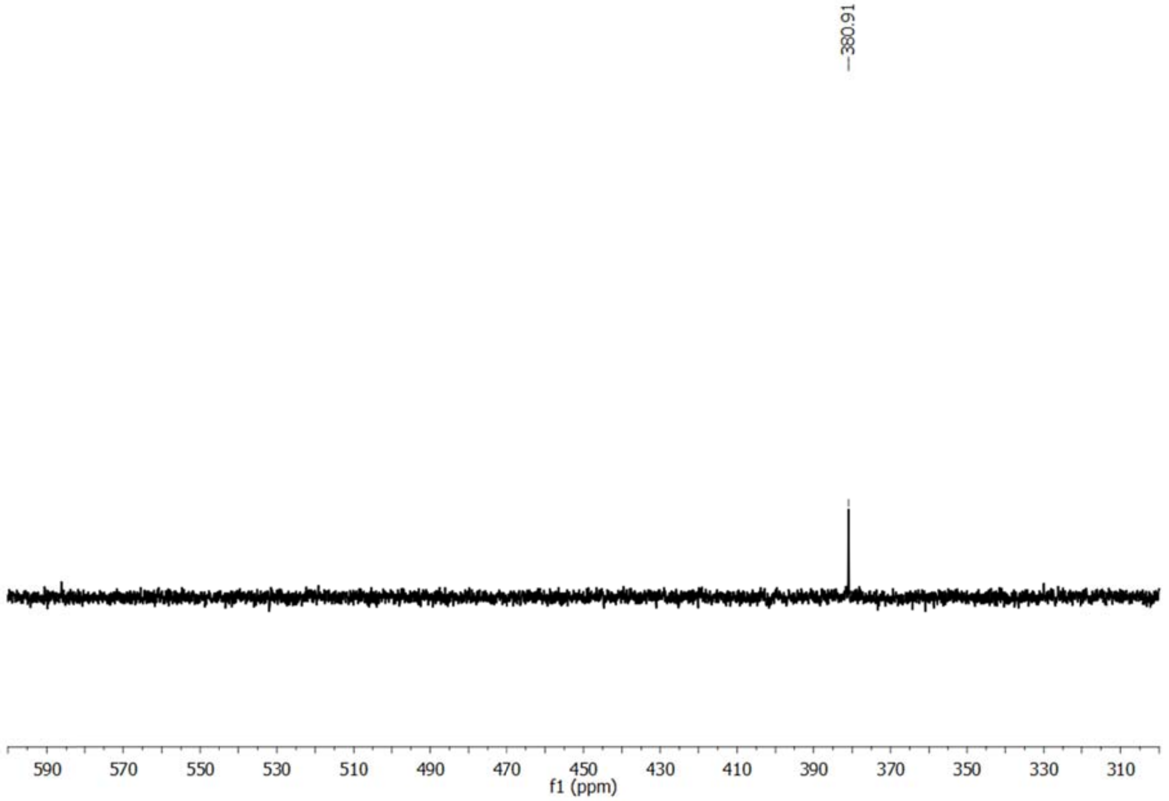

Figure S117. ^{77}Se-NMR of compound **7c**.

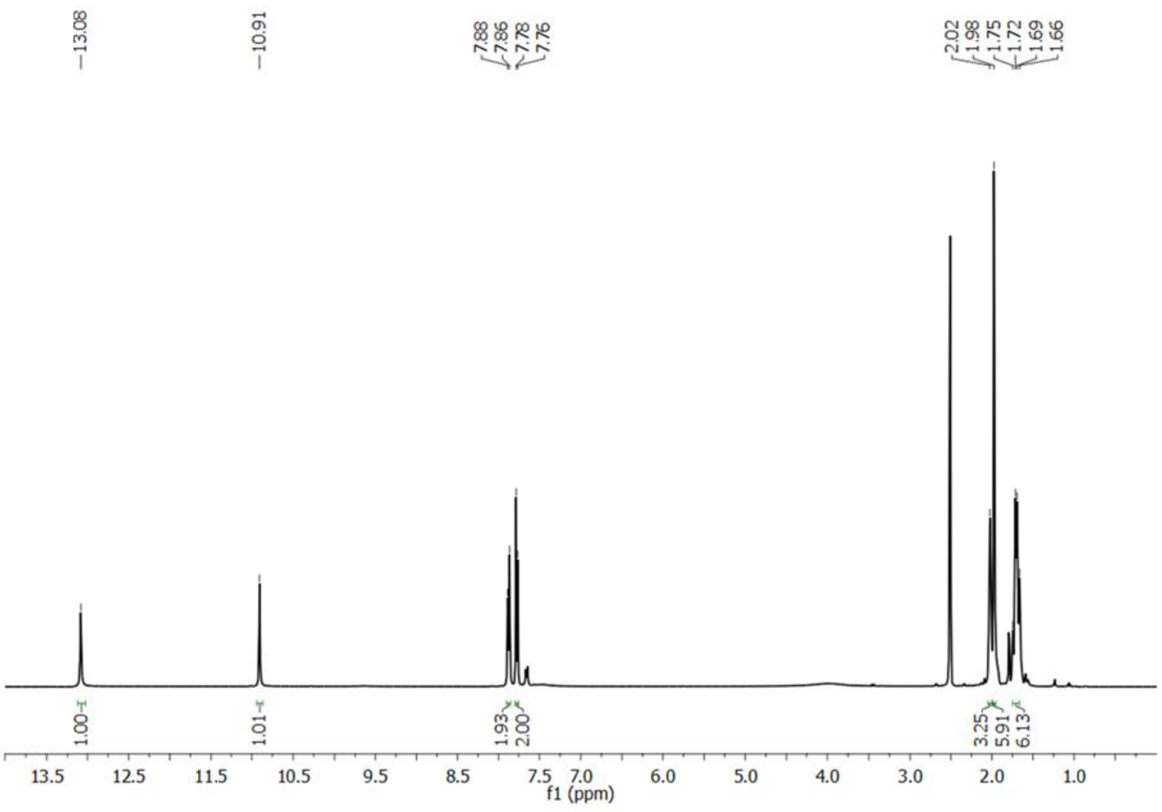

Figure S118. ^{1}H-NMR of compound **7d**.

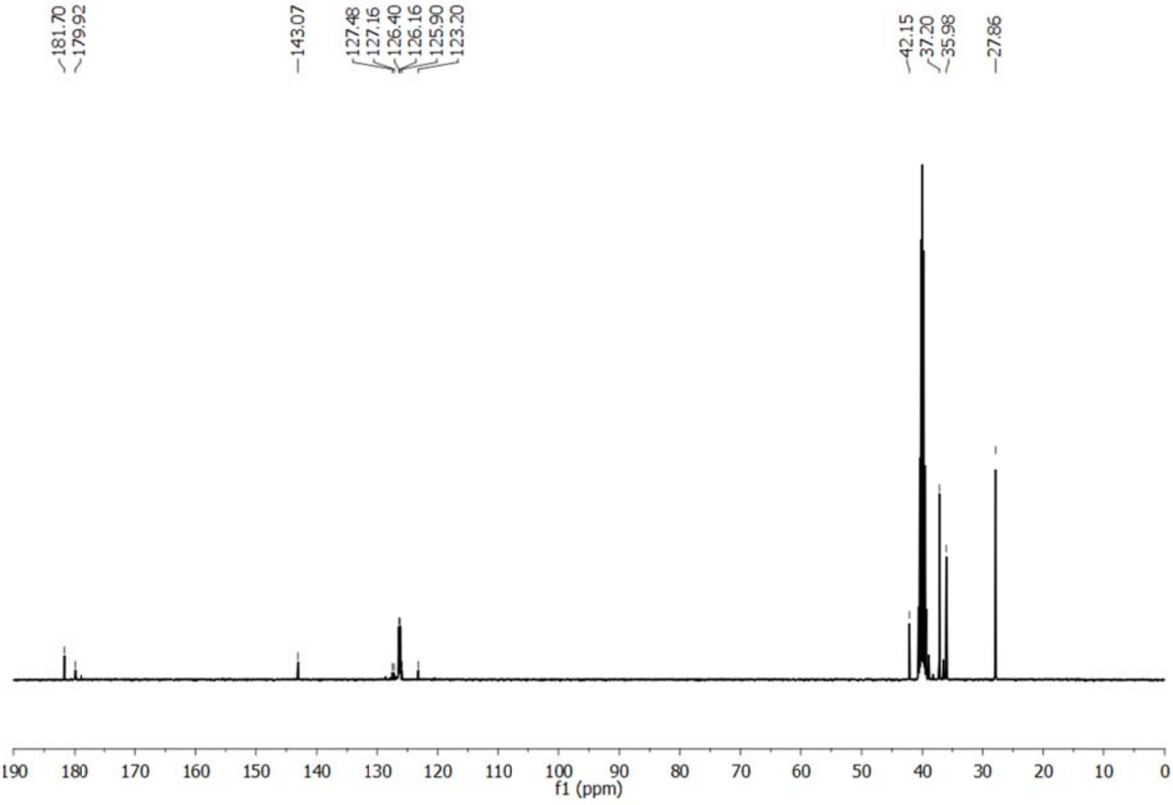

Figure S119. ^{13}C-NMR of compound **7d**.

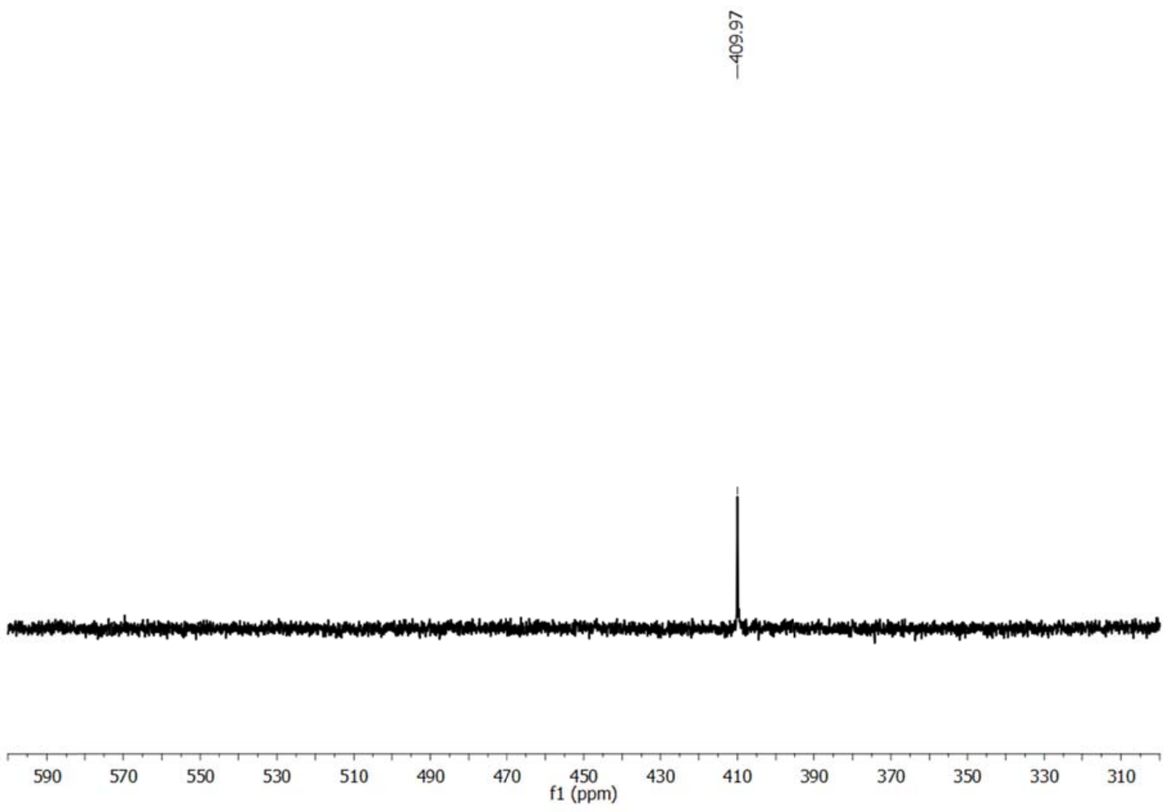

Figure S120. ^{77}Se-NMR of compound **7d**.

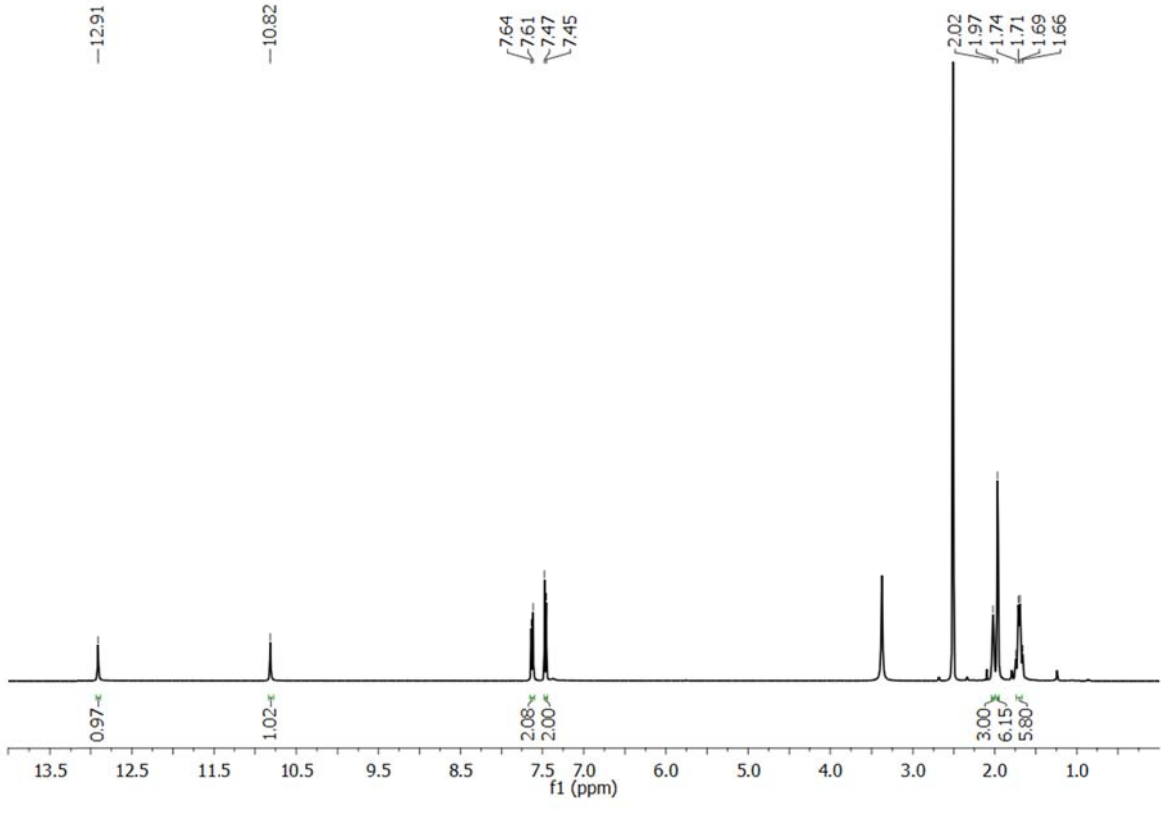

Figure S121. ^{1}H-NMR of compound **7e**.

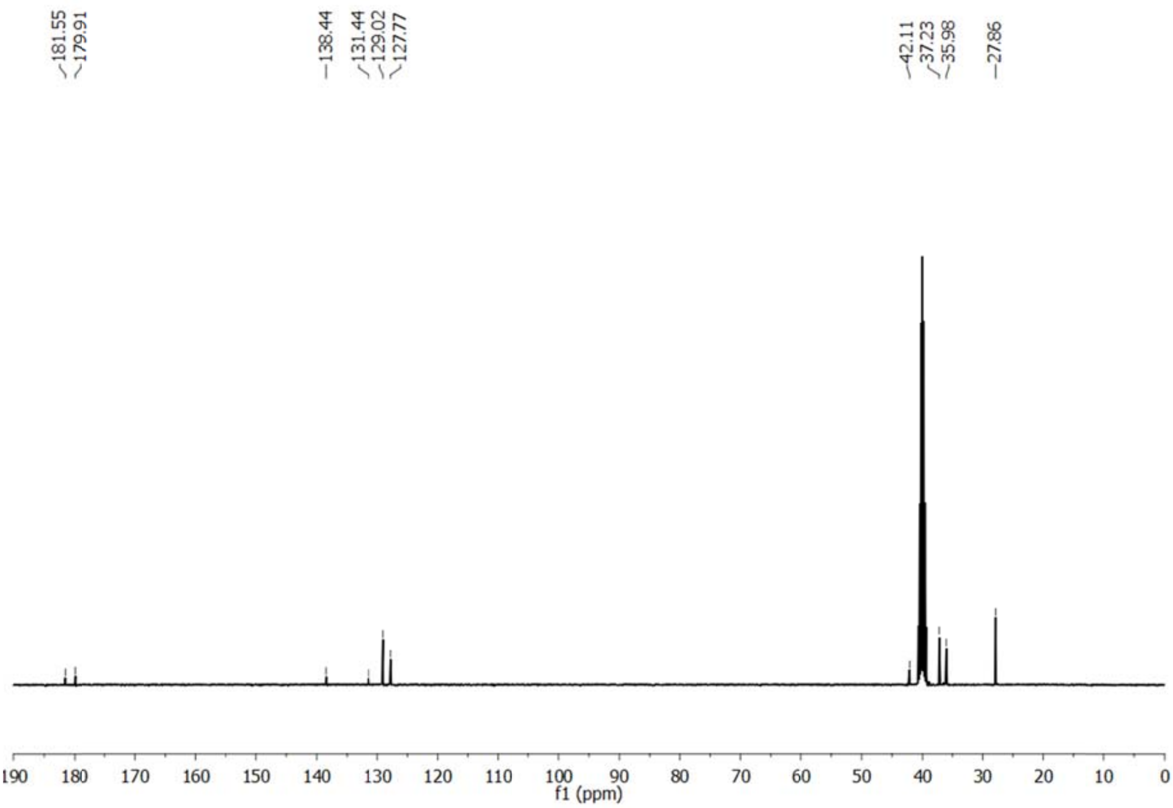

Figure S122. ^{13}C-NMR of compound **7e**.

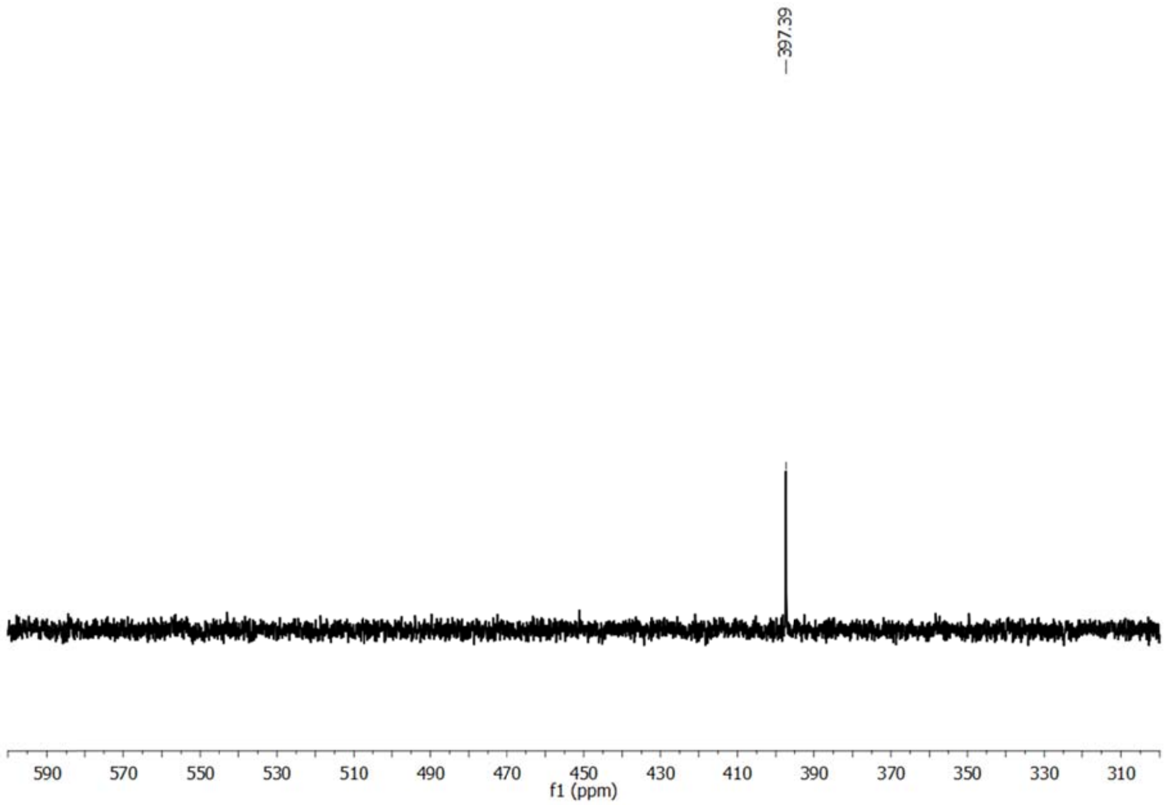

Figure S123. ^{77}Se-NMR of compound **7e**.

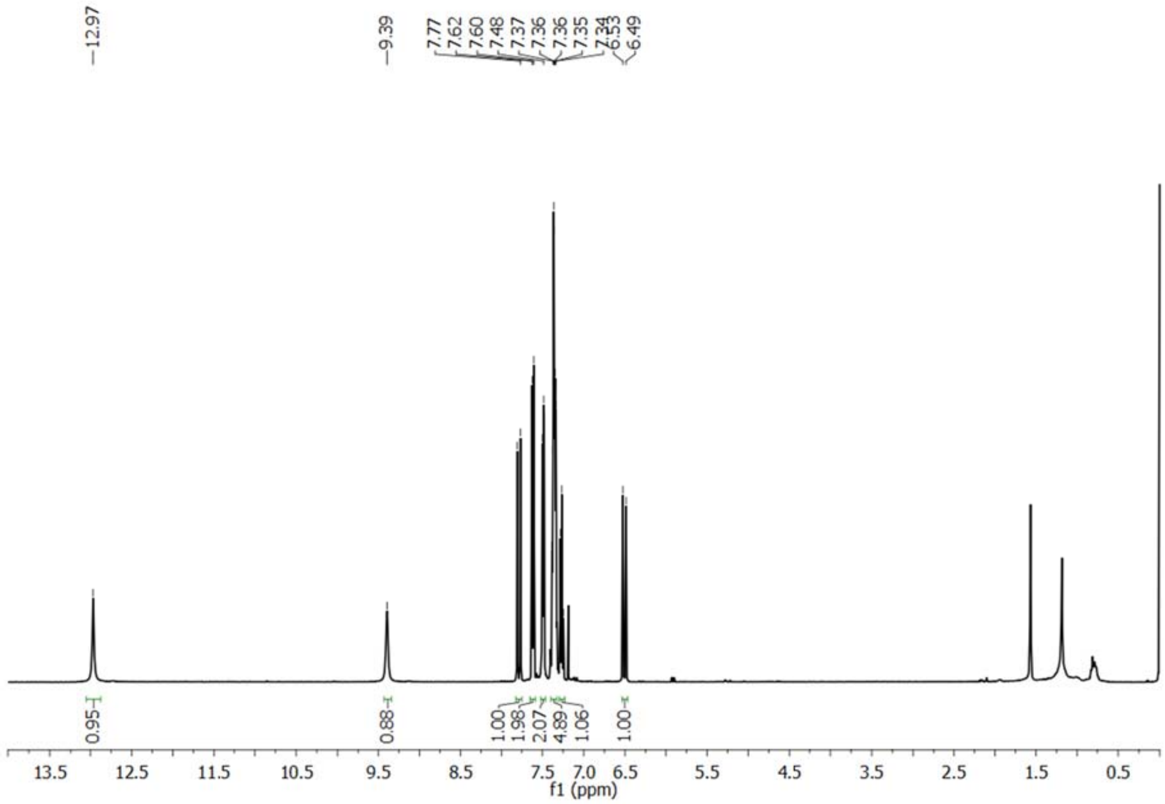

Figure S124. ^{1}H-NMR of compound **8a**.

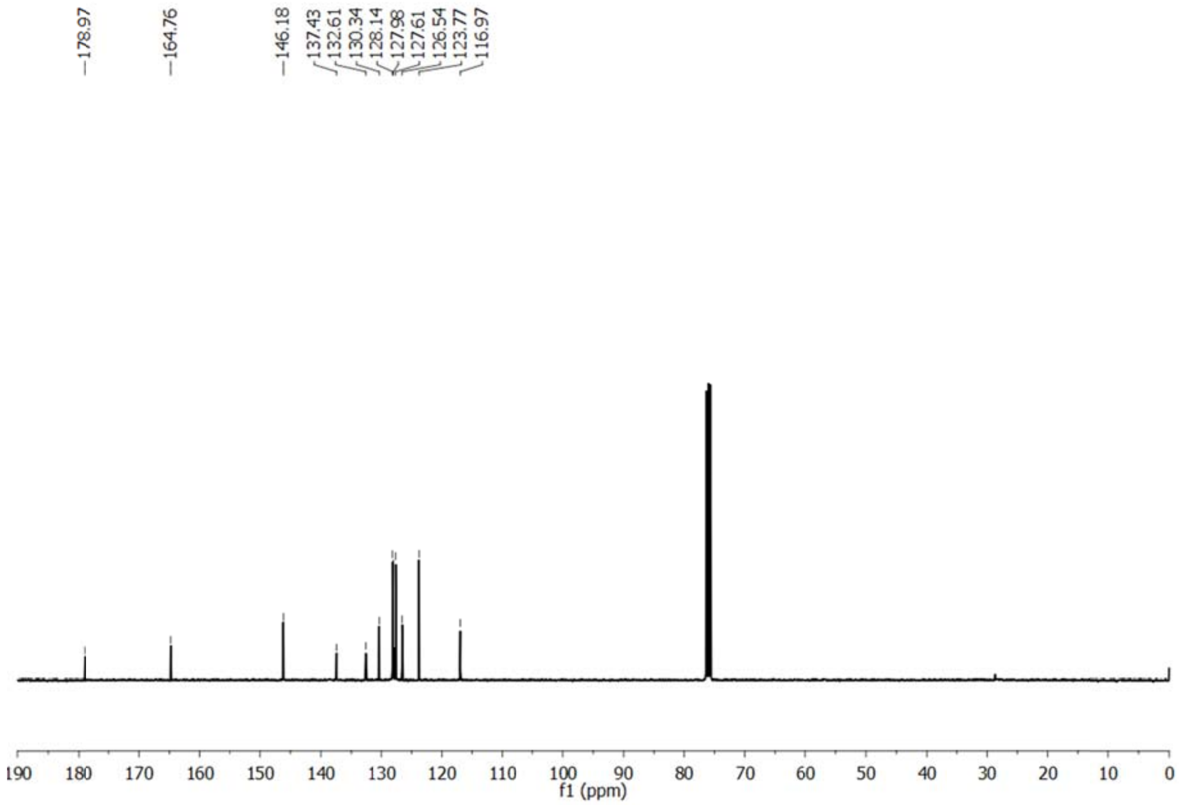

Figure S125. ^{13}C-NMR of compound **8a**.

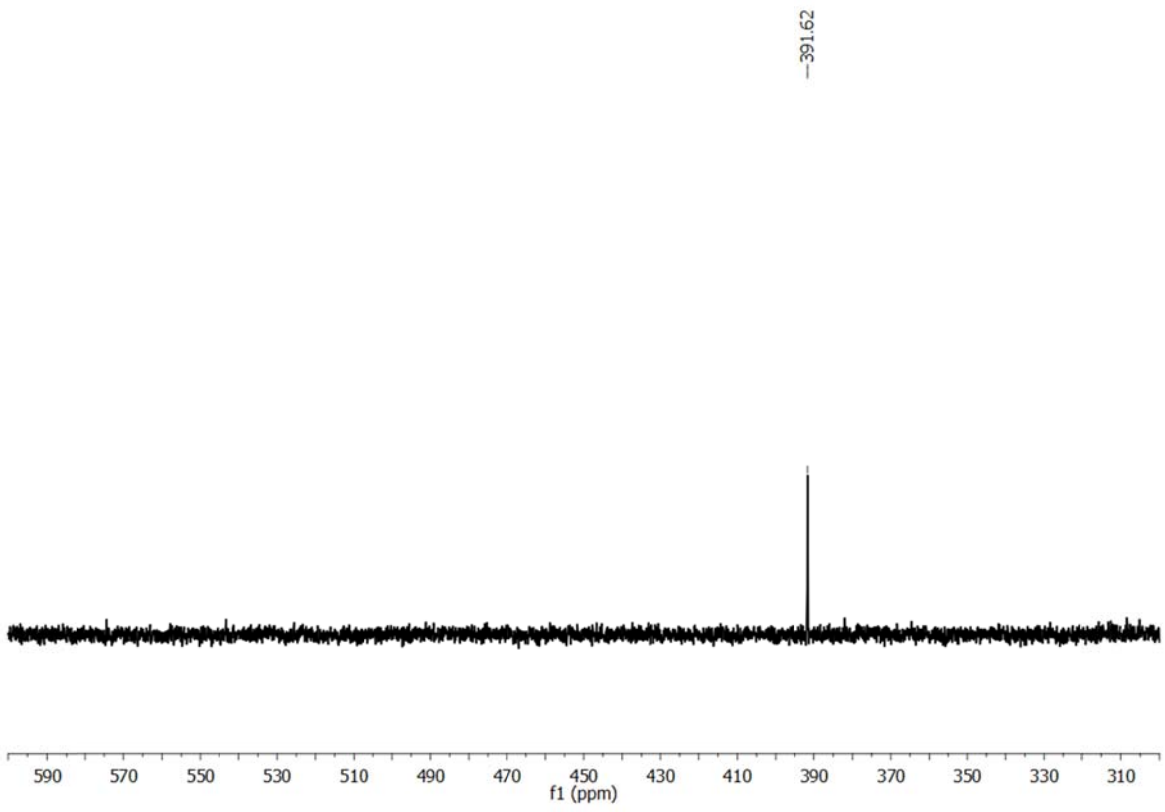

Figure S126. ^{77}Se-NMR of compound **8a**.

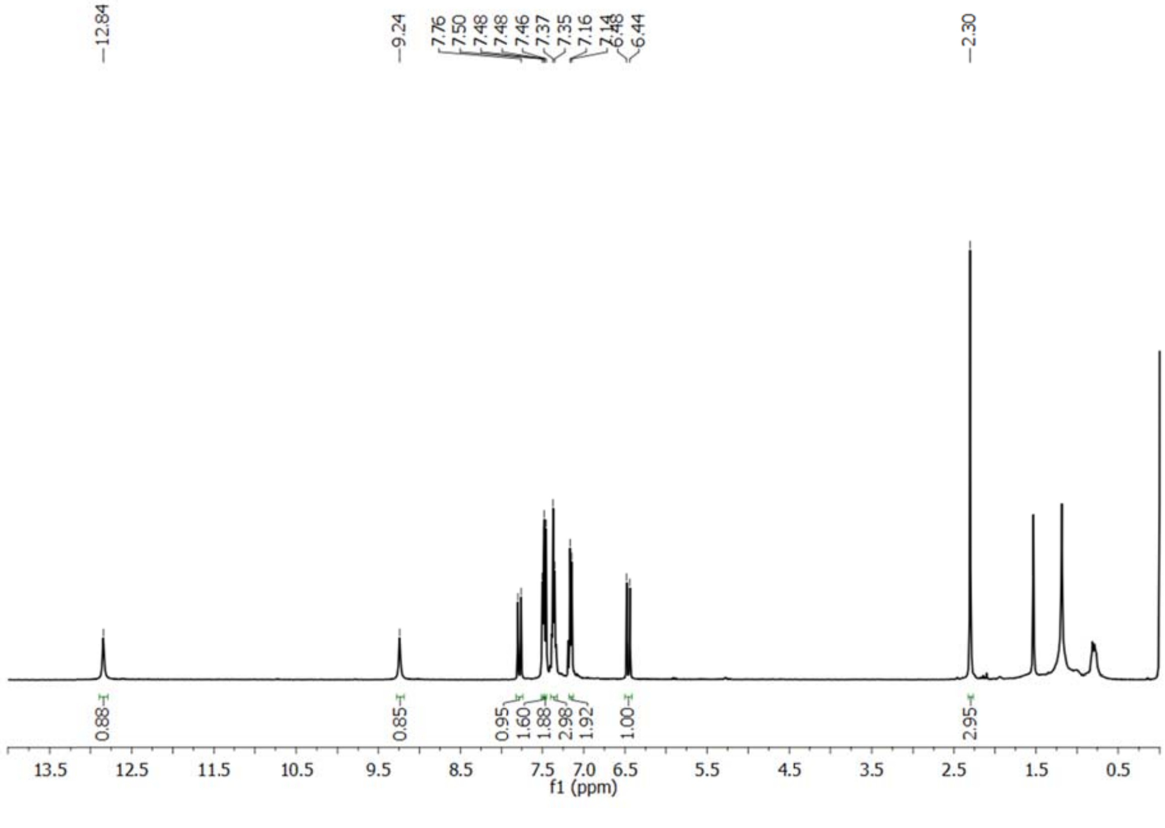

Figure S127. ^{1}H-NMR of compound **8b**.

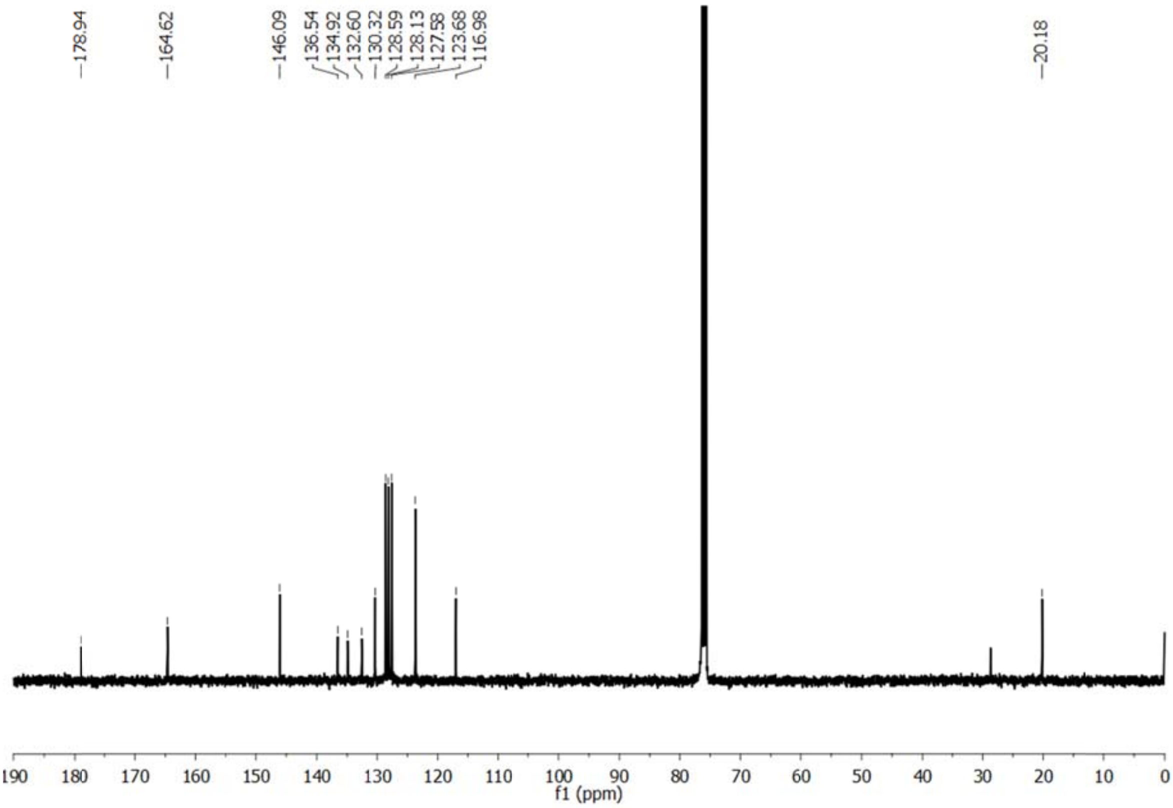

Figure S128. ^{13}C-NMR of compound **8b**.

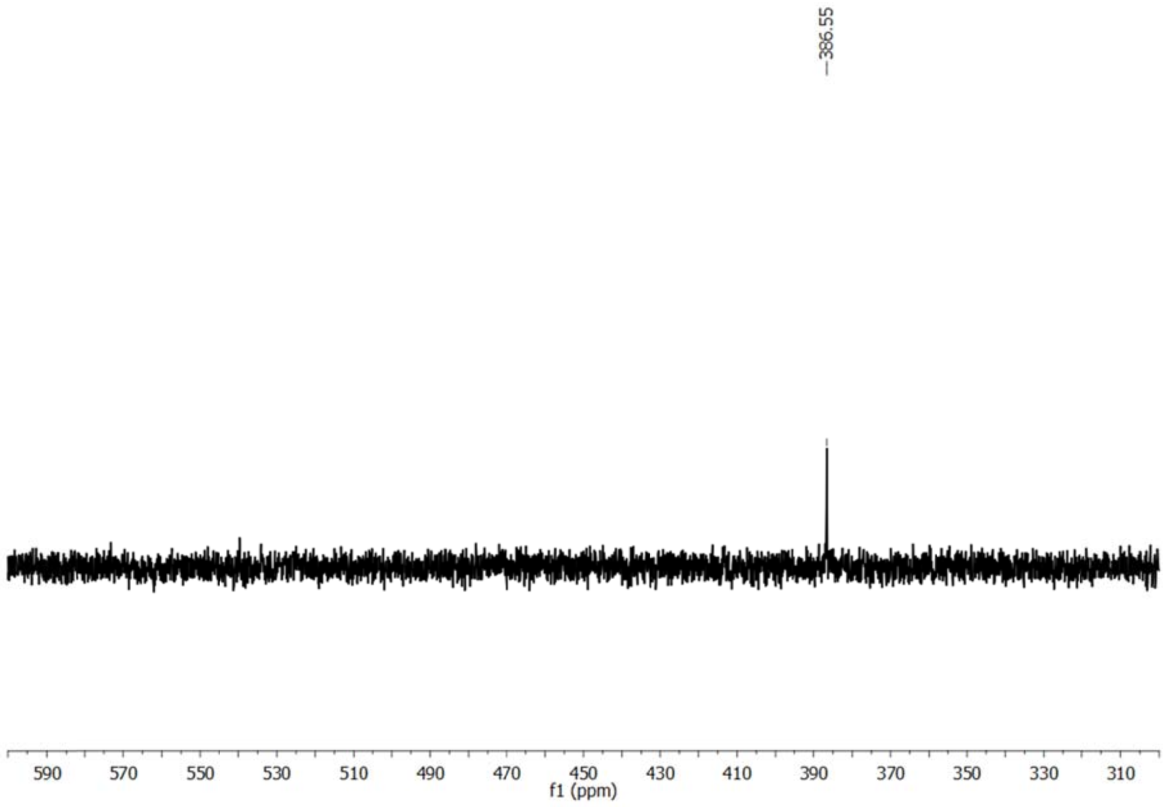

Figure S129. ^{77}Se-NMR of compound **8b**.

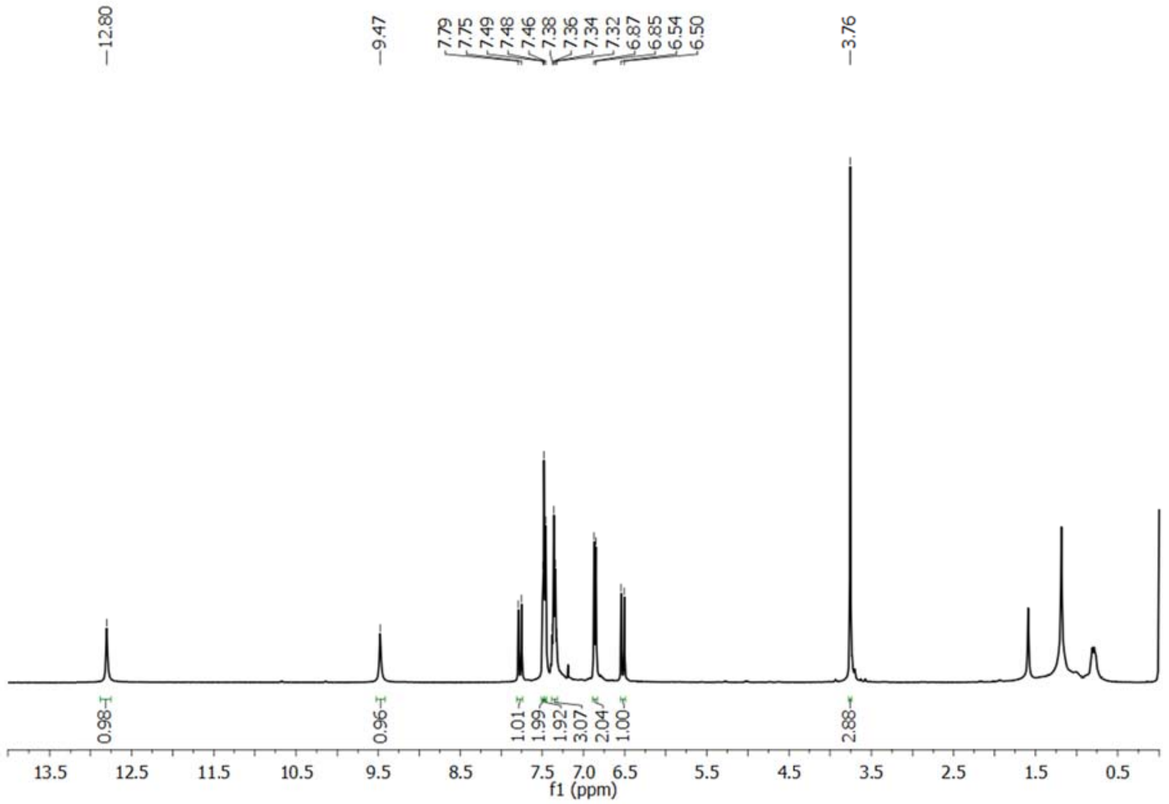

Figure S130. ^{1}H-NMR of compound **8c**.

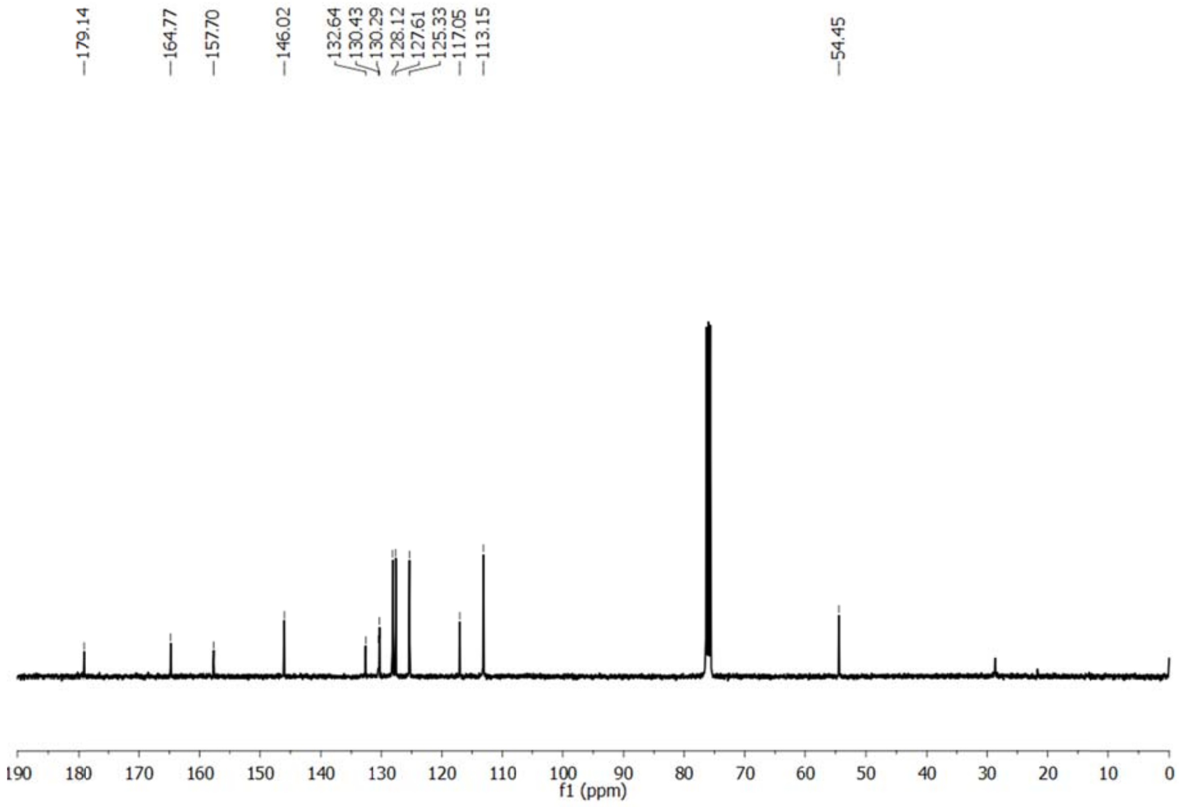

Figure S131. ^{13}C-NMR of compound **8c**.

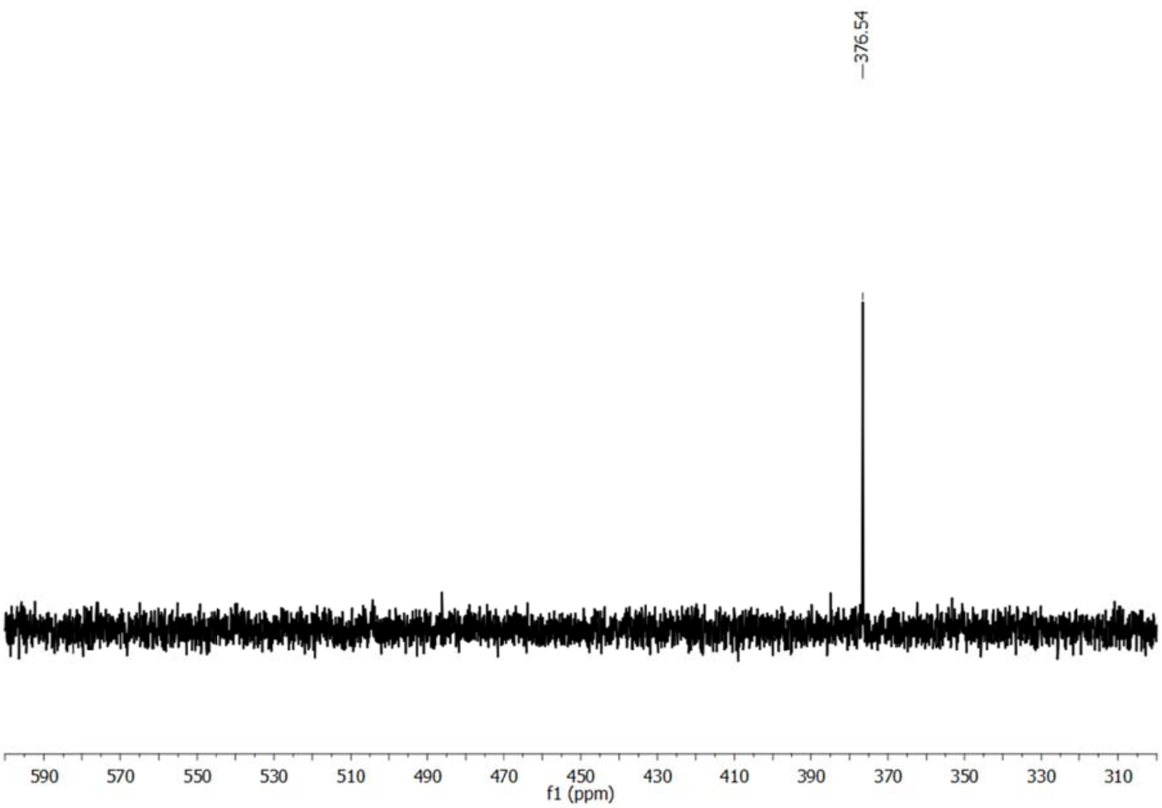

Figure S132. ^{77}Se-NMR of compound **8c**.

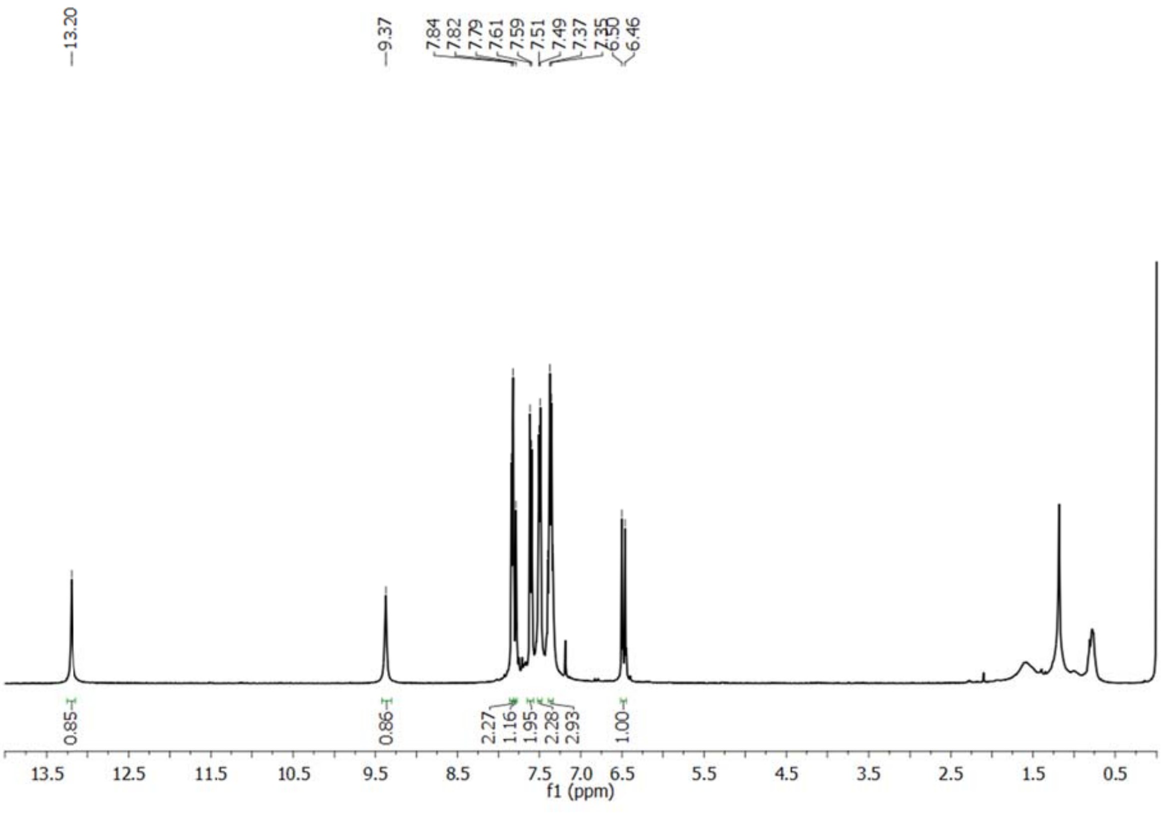

Figure S133. ^{1}H-NMR of compound **8d**.

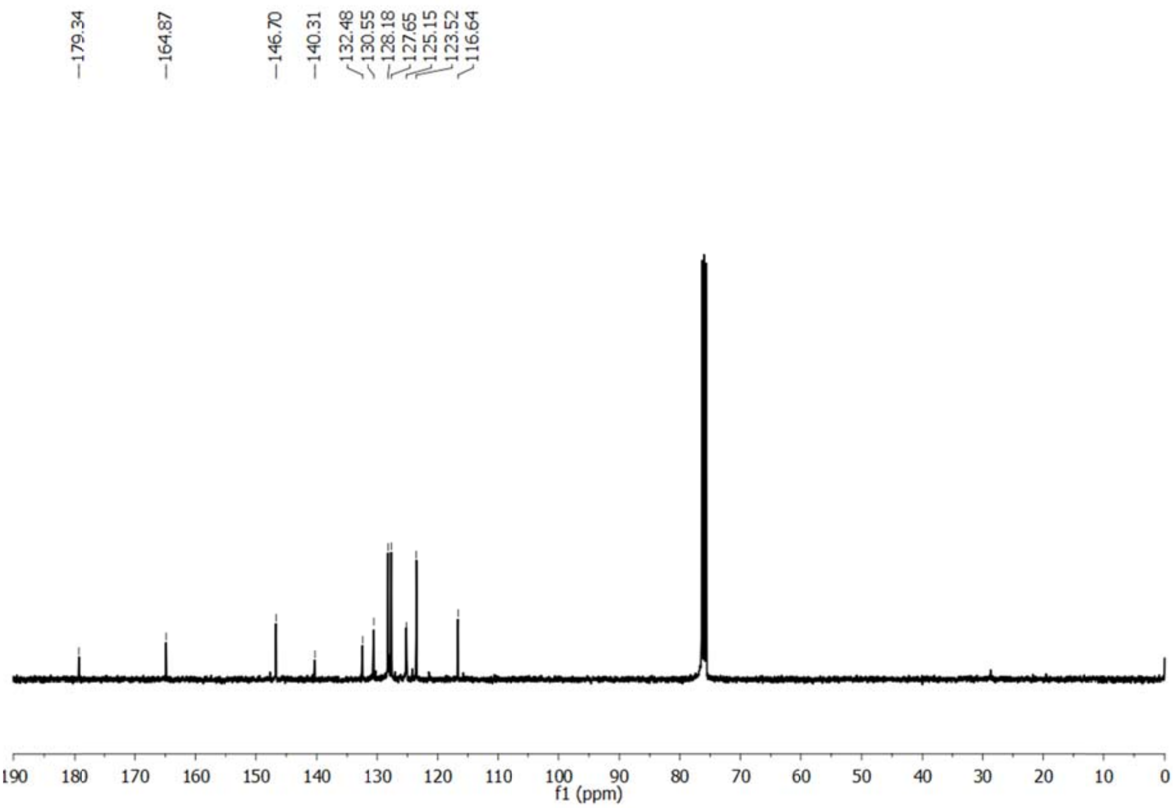

Figure S134. ^{13}C-NMR of compound **8d**.

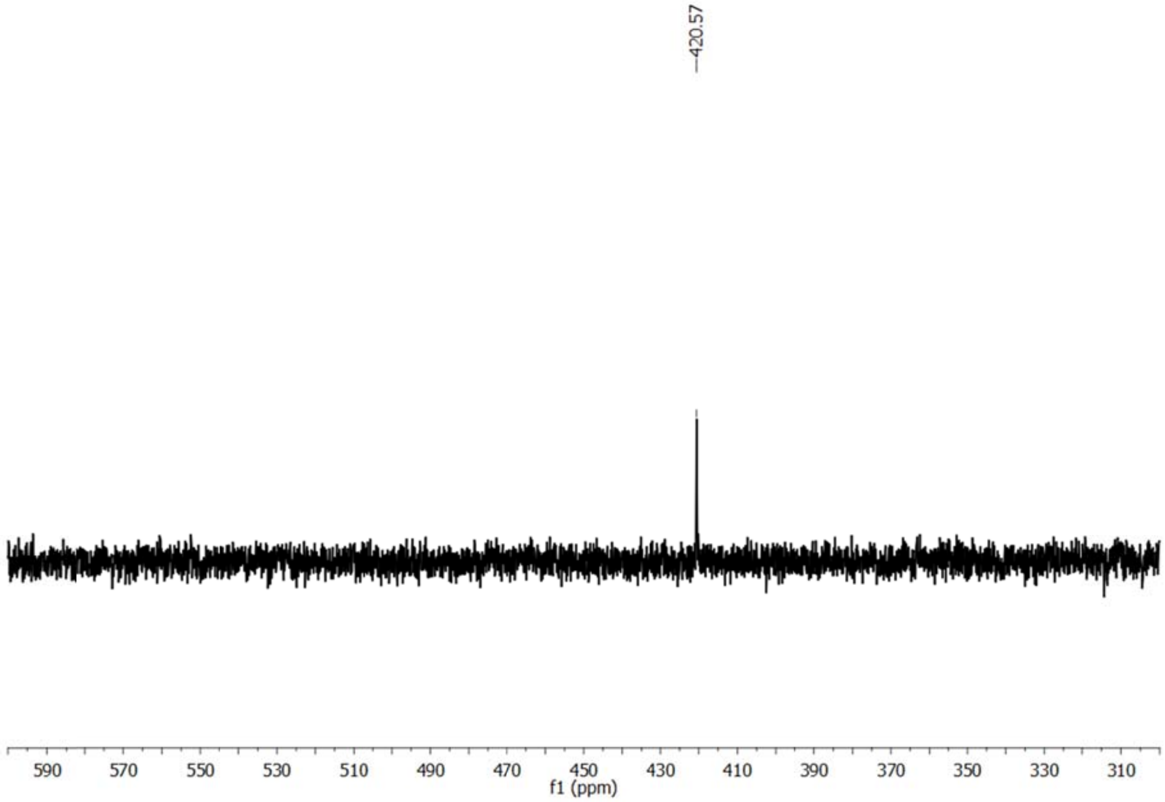

Figure S135. ^{77}Se-NMR of compound **8d**.

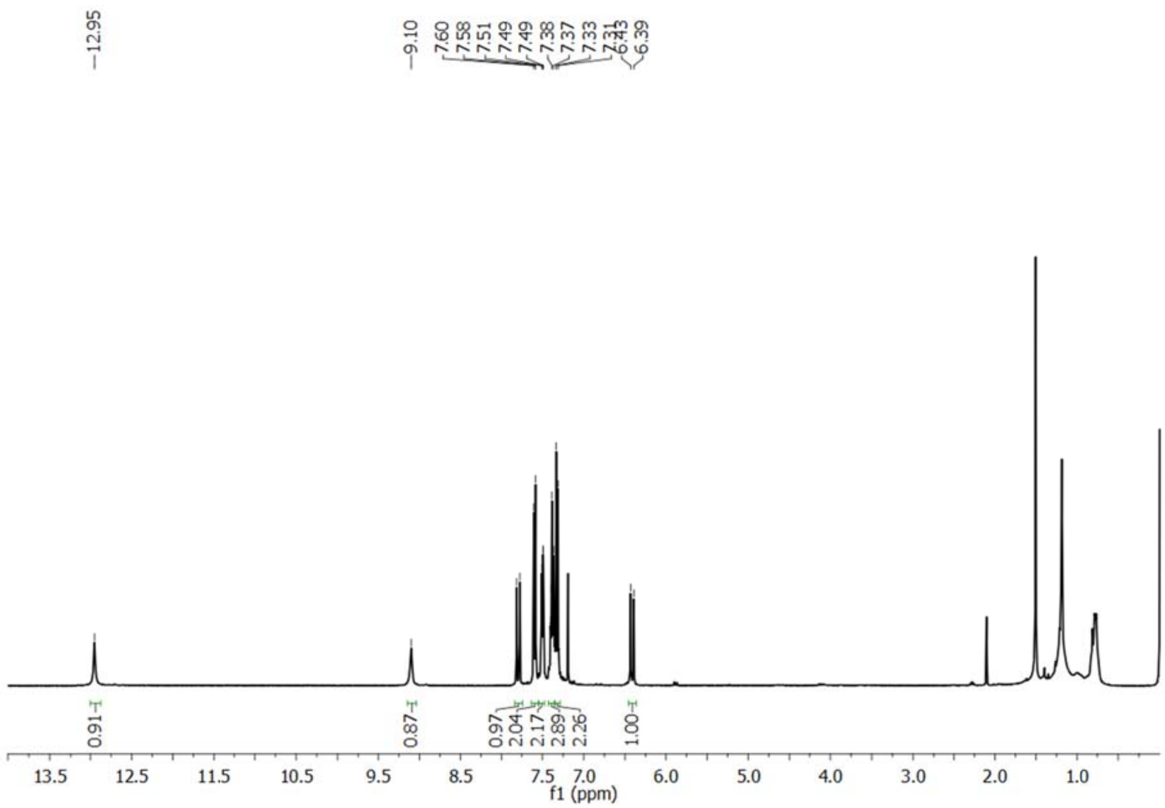

Figure S136. ^{1}H-NMR of compound **8e**.

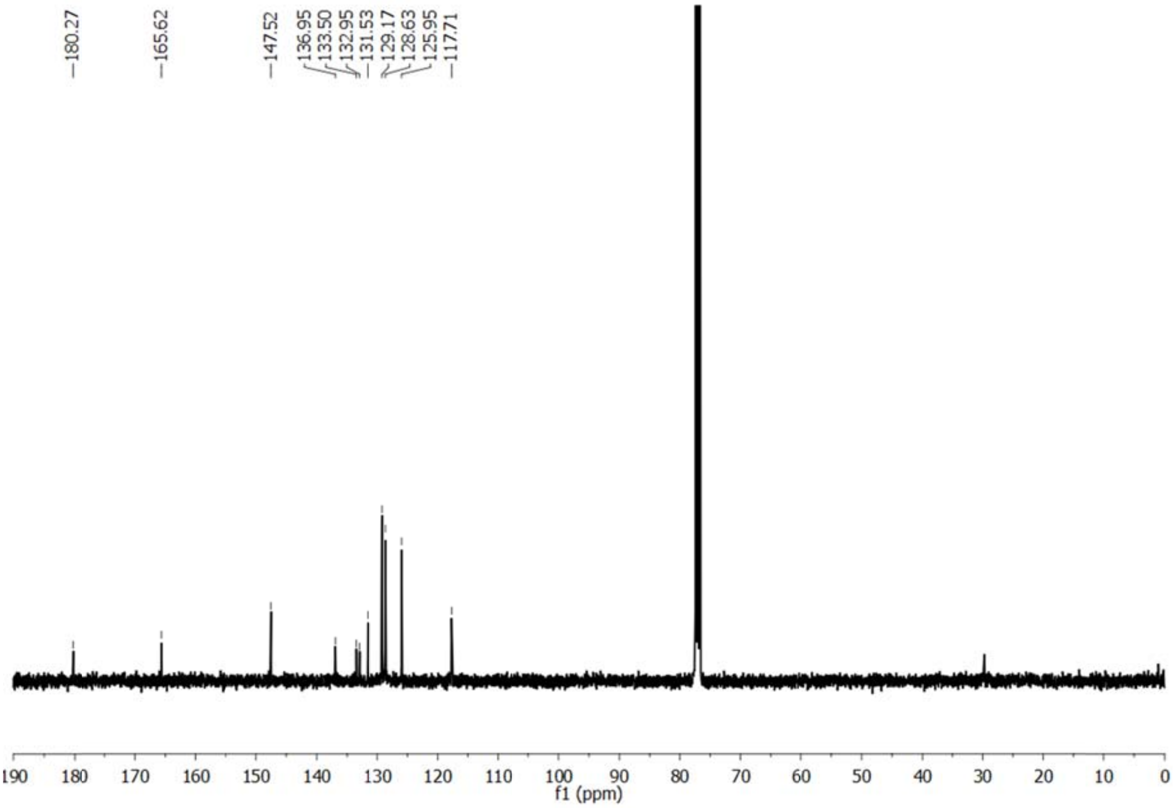

Figure S137. ^{13}C-NMR of compound **8e**.

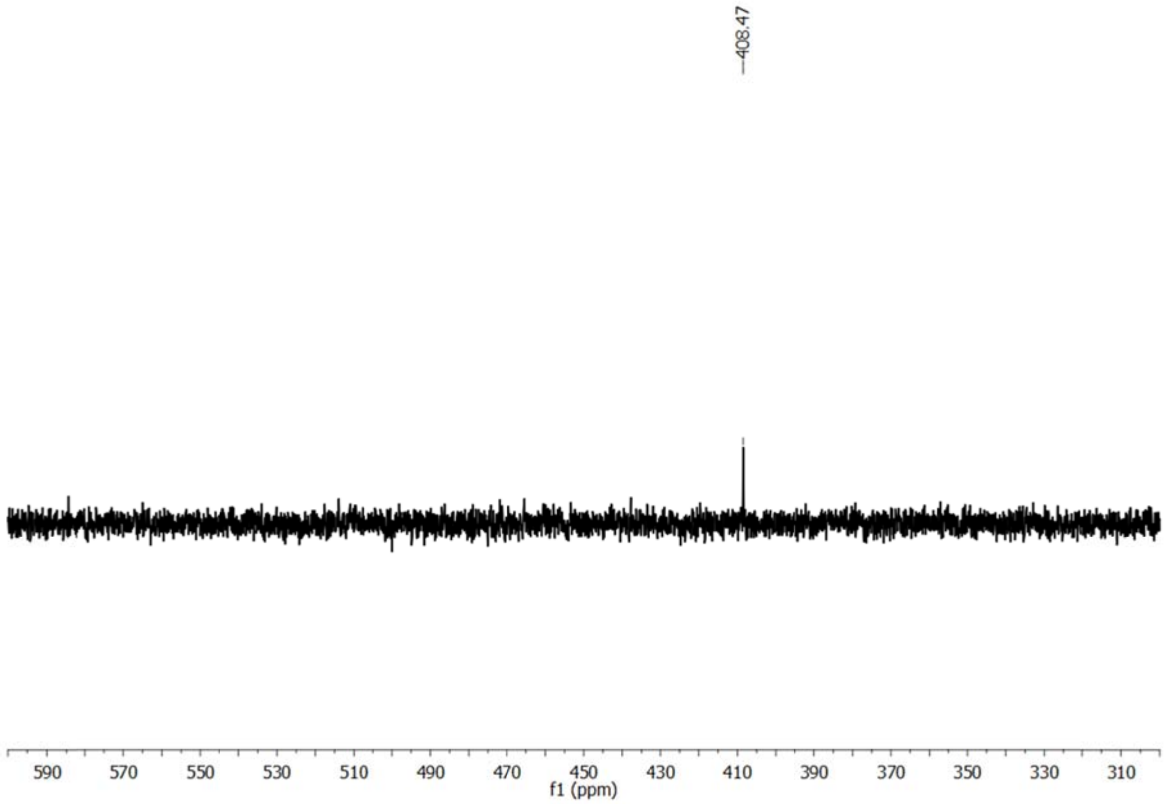

Figure S138. ^{77}Se-NMR of compound **8e**.

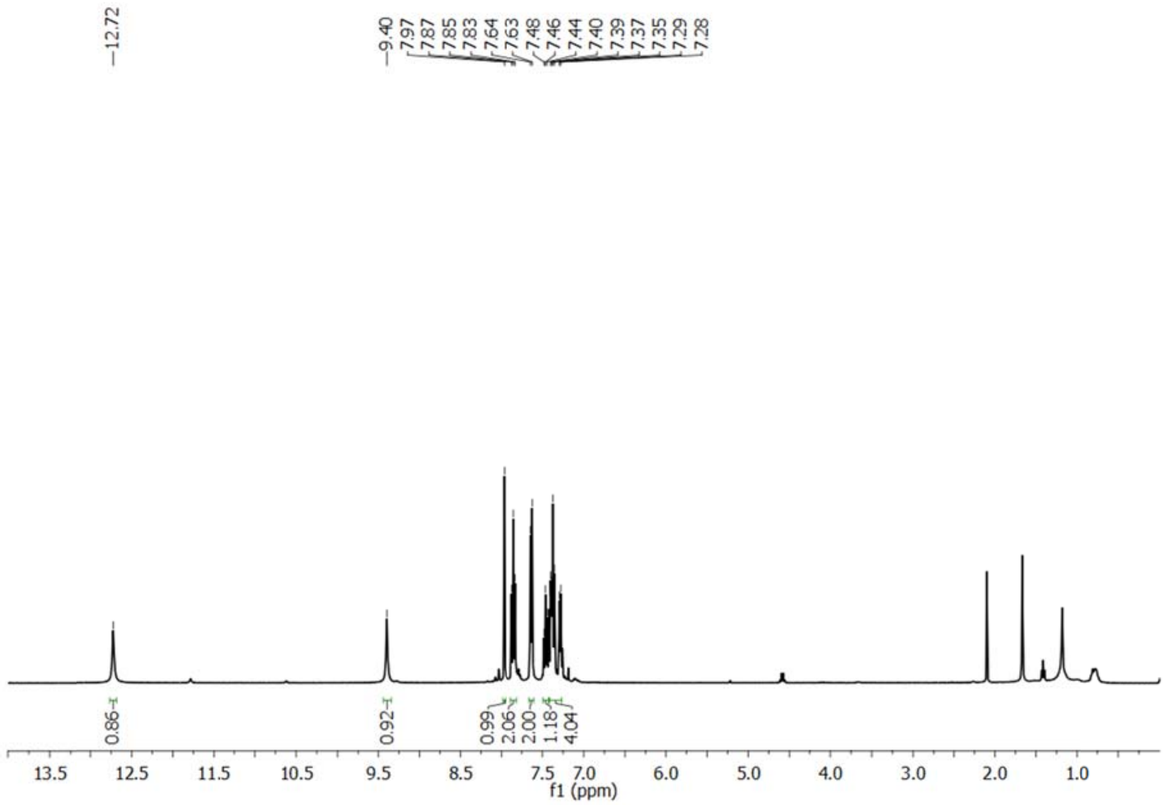

Figure S139. ^{1}H-NMR of compound **9a**.

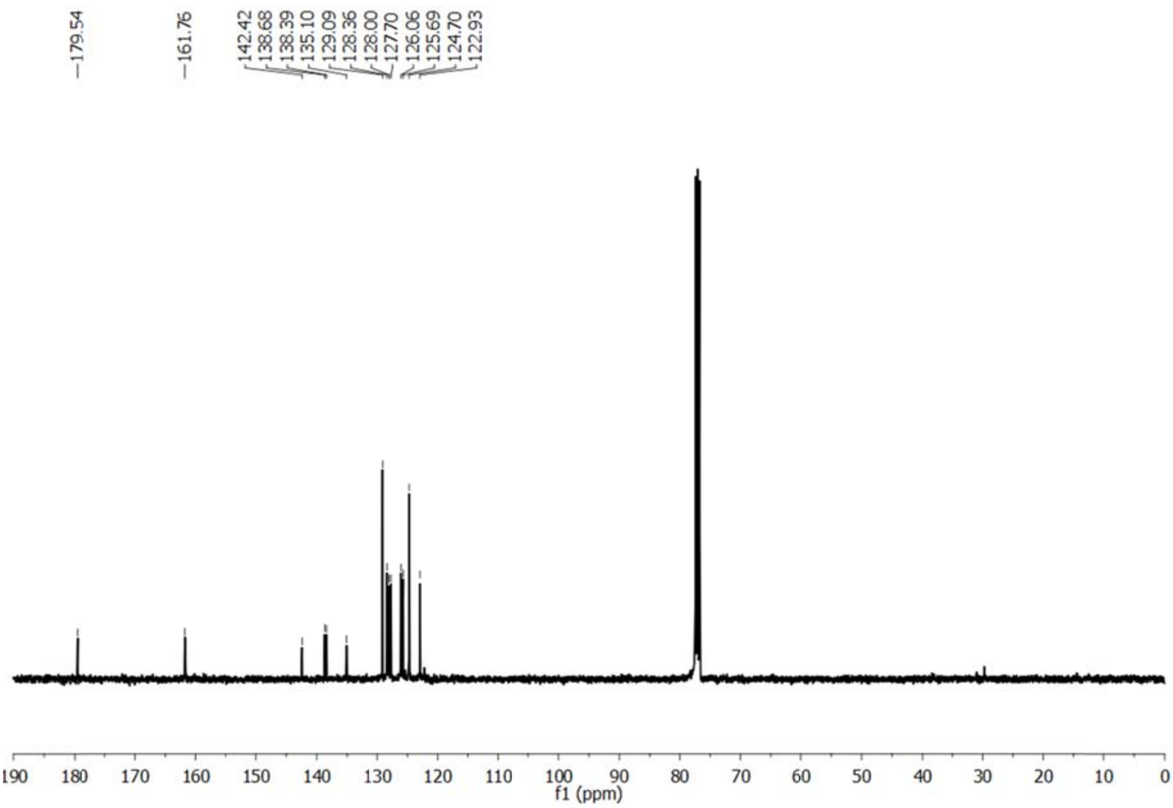

Figure S140. ^{13}C-NMR of compound **9a**.

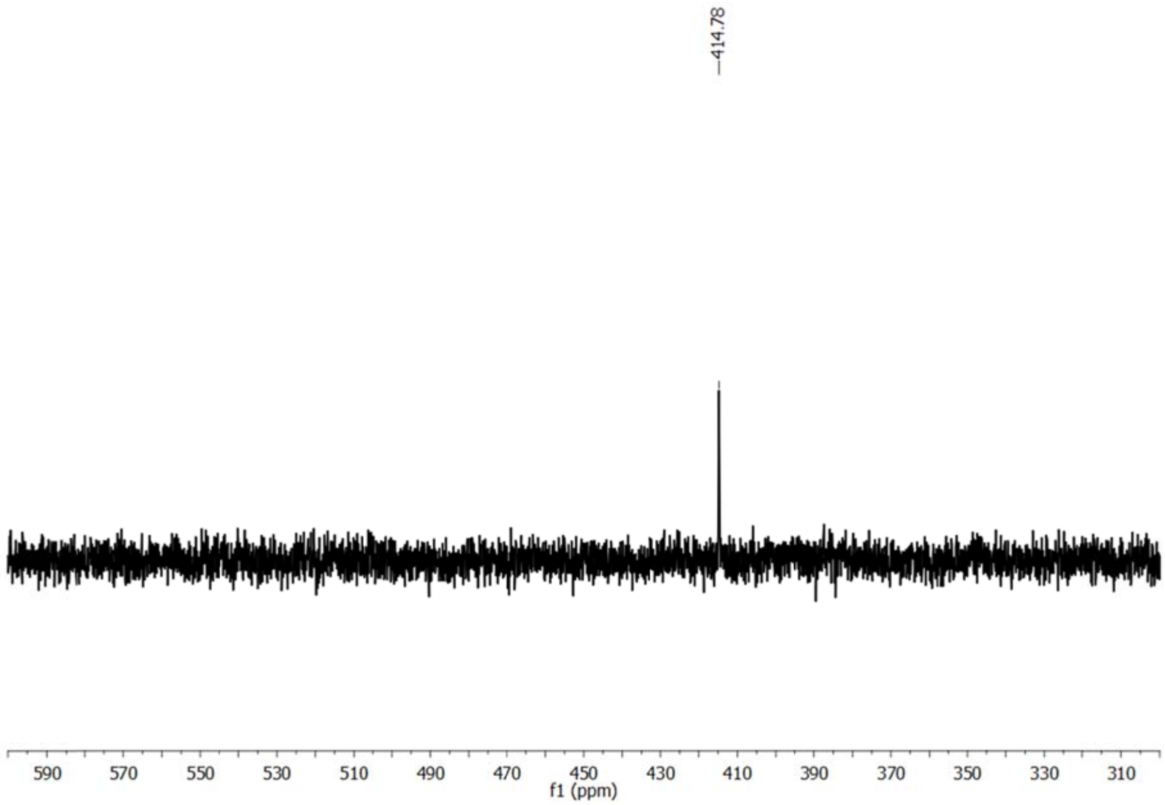

Figure S141. ^{77}Se-NMR of compound **9a**.

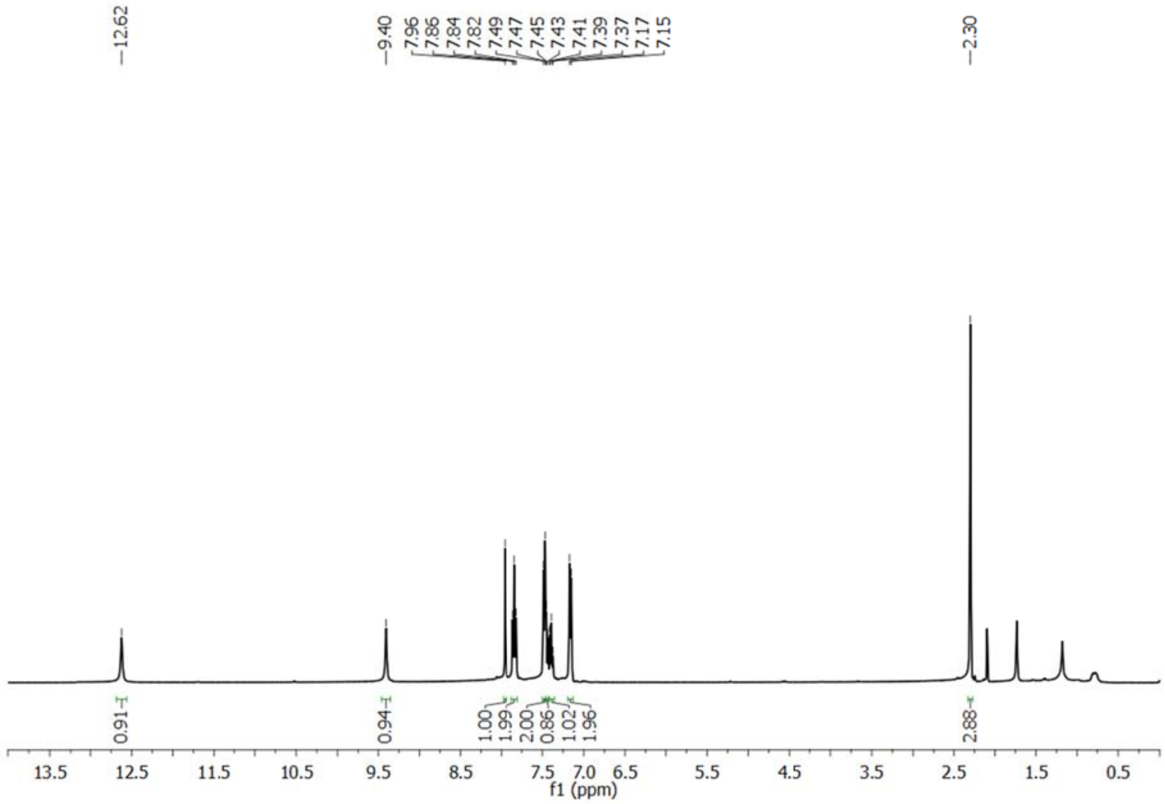

Figure S142. ^{1}H-NMR of compound **9b**.

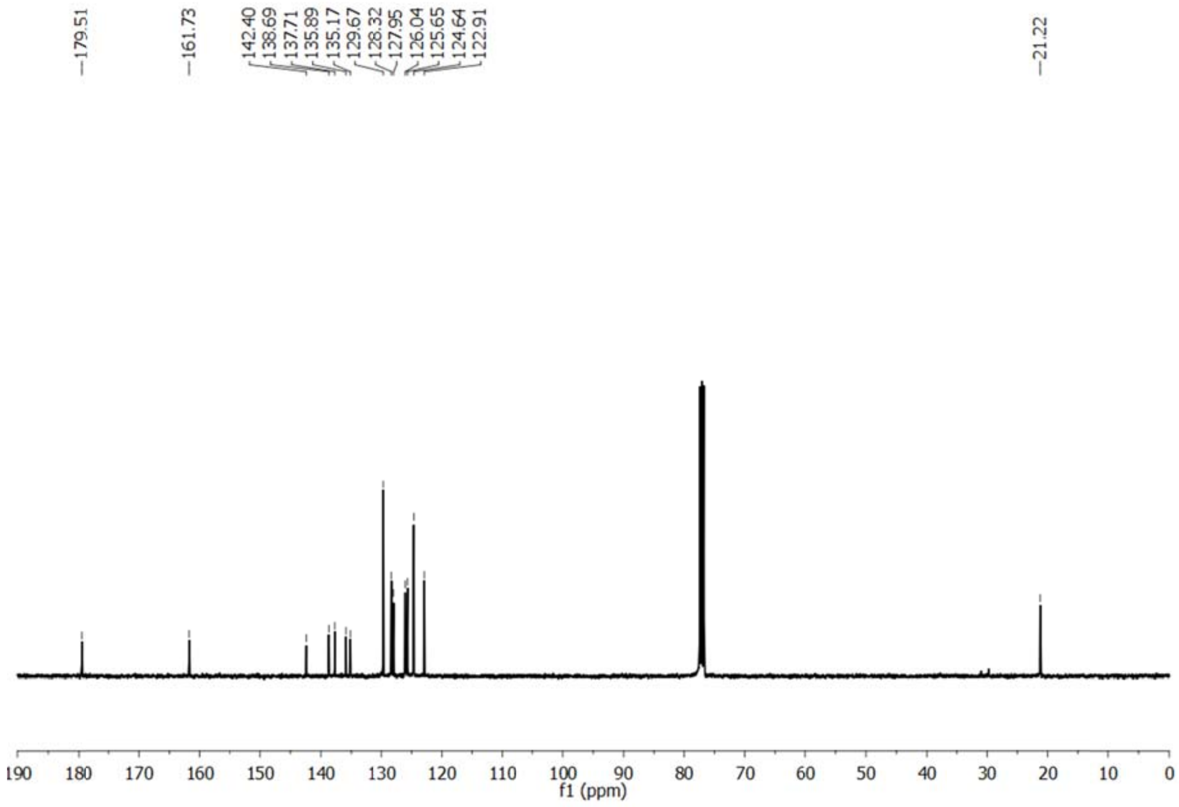

Figure S143. ^{13}C-NMR of compound **9b**.

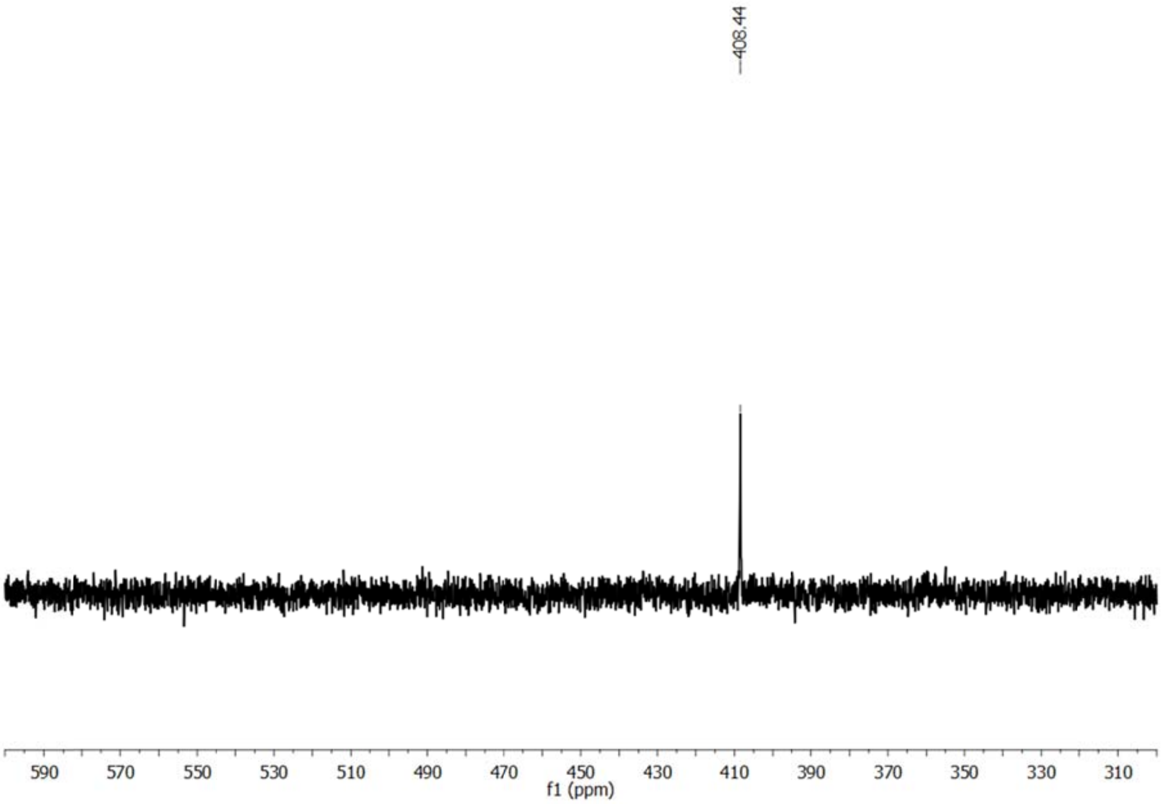

Figure S144. ^{77}Se-NMR of compound **9b**.

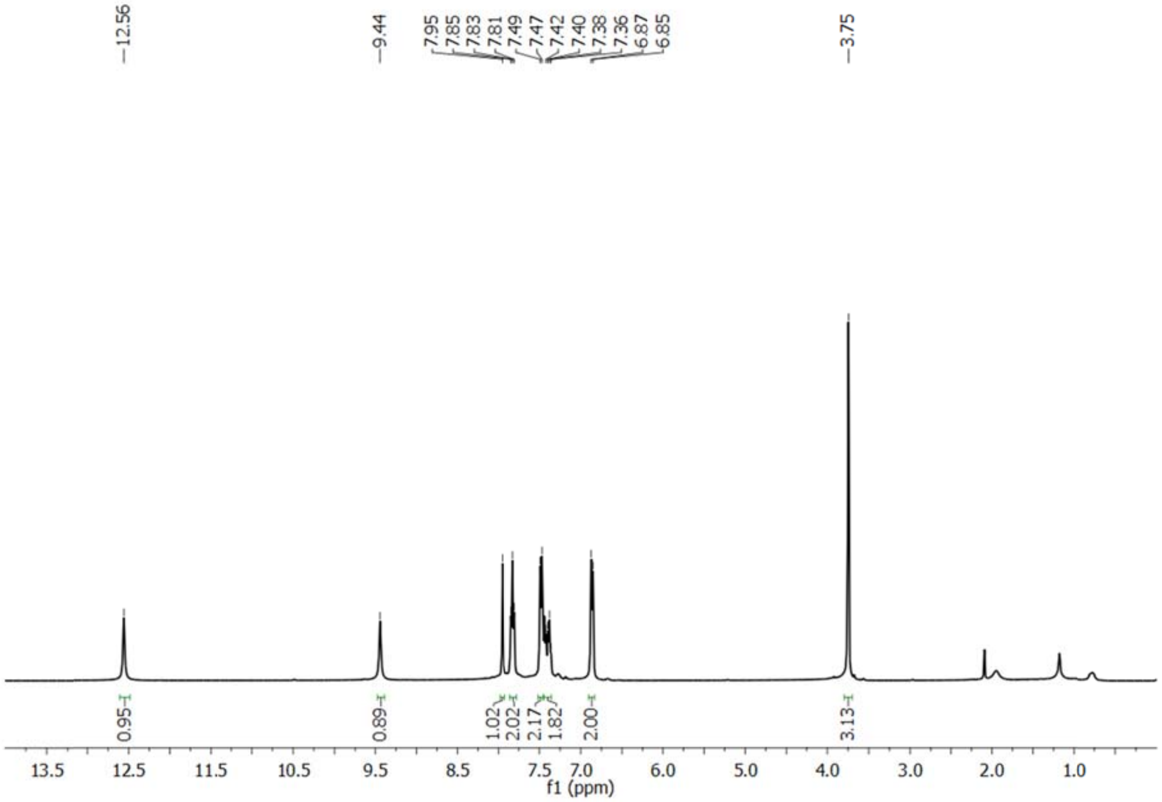

Figure S145. ^{1}H-NMR of compound **9c**.

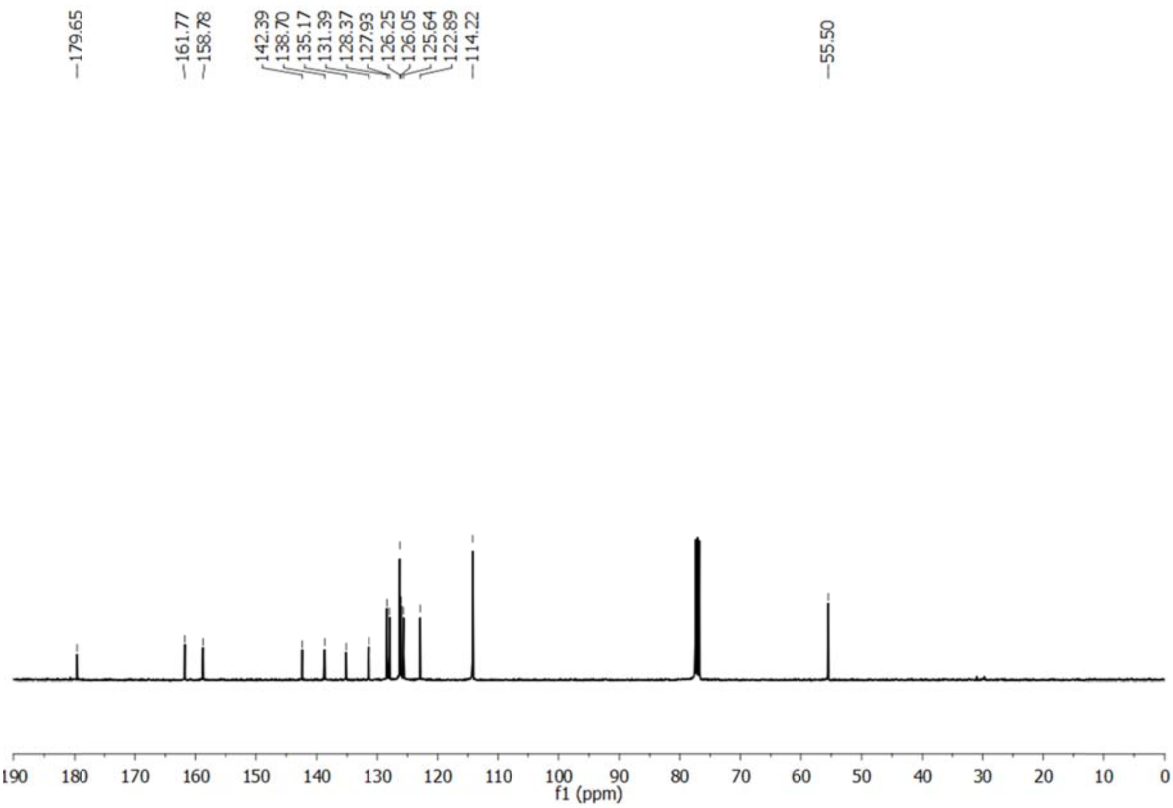

Figure S146. ^{13}C-NMR of compound **9c**.

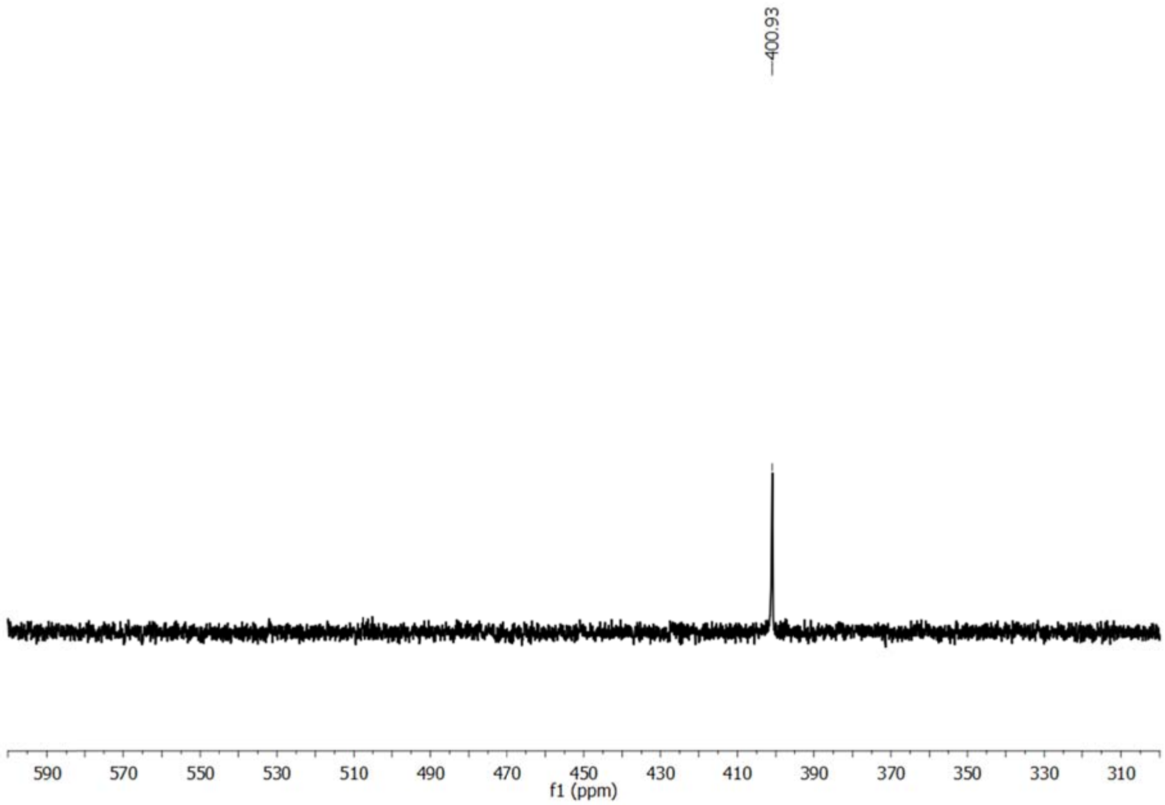

Figure S147. ^{77}Se-NMR of compound **9c**.

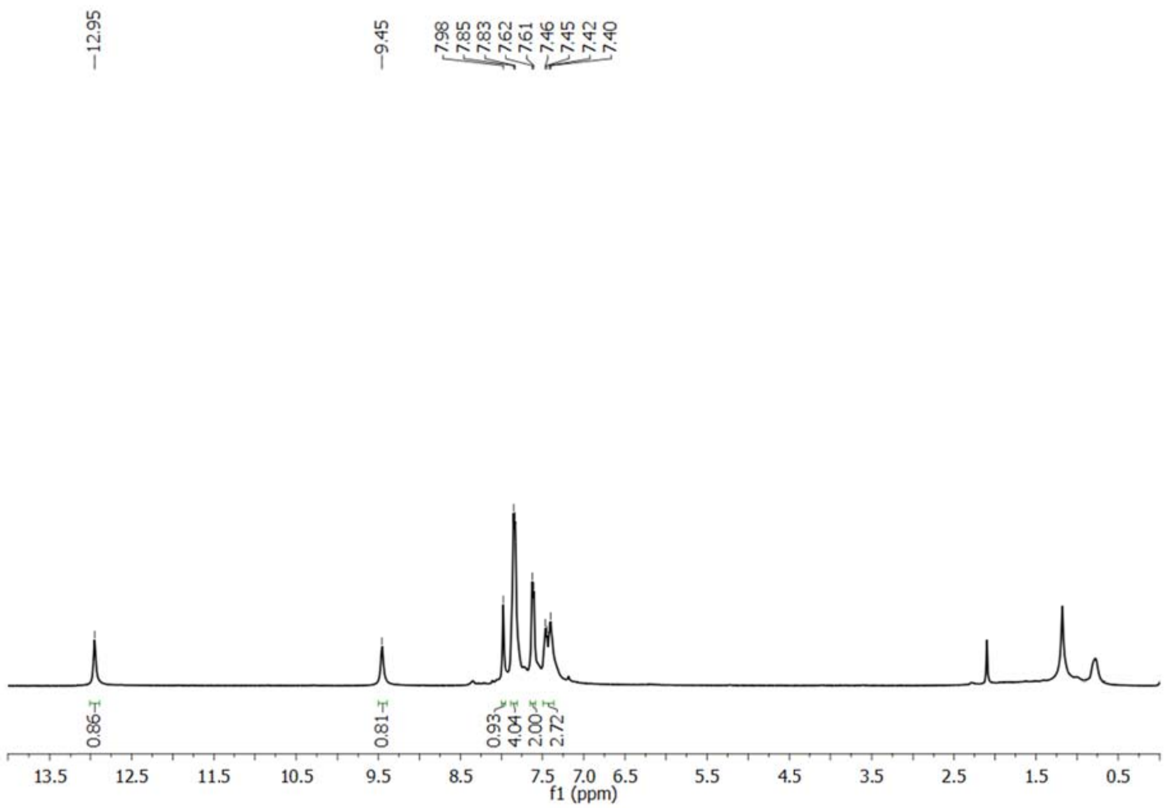

Figure S148. ^{1}H-NMR of compound **9d**.

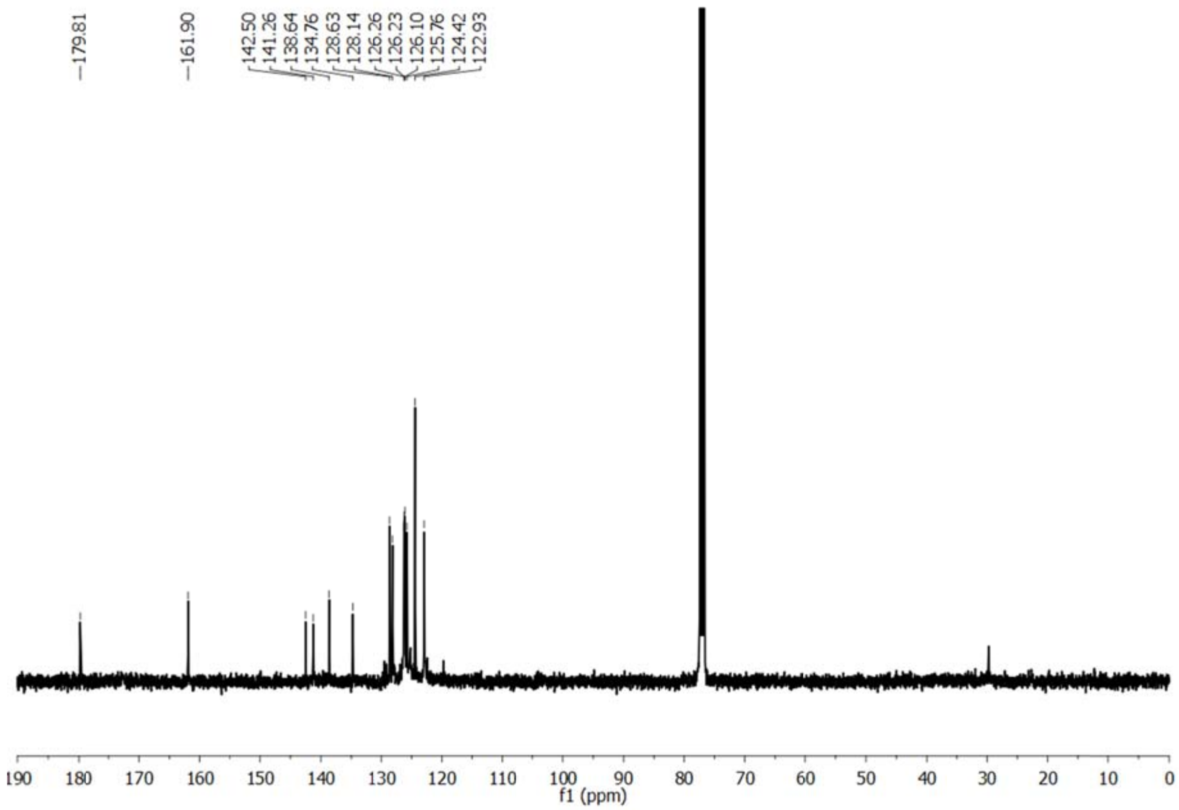

Figure S149. ^{13}C-NMR of compound **9d**.

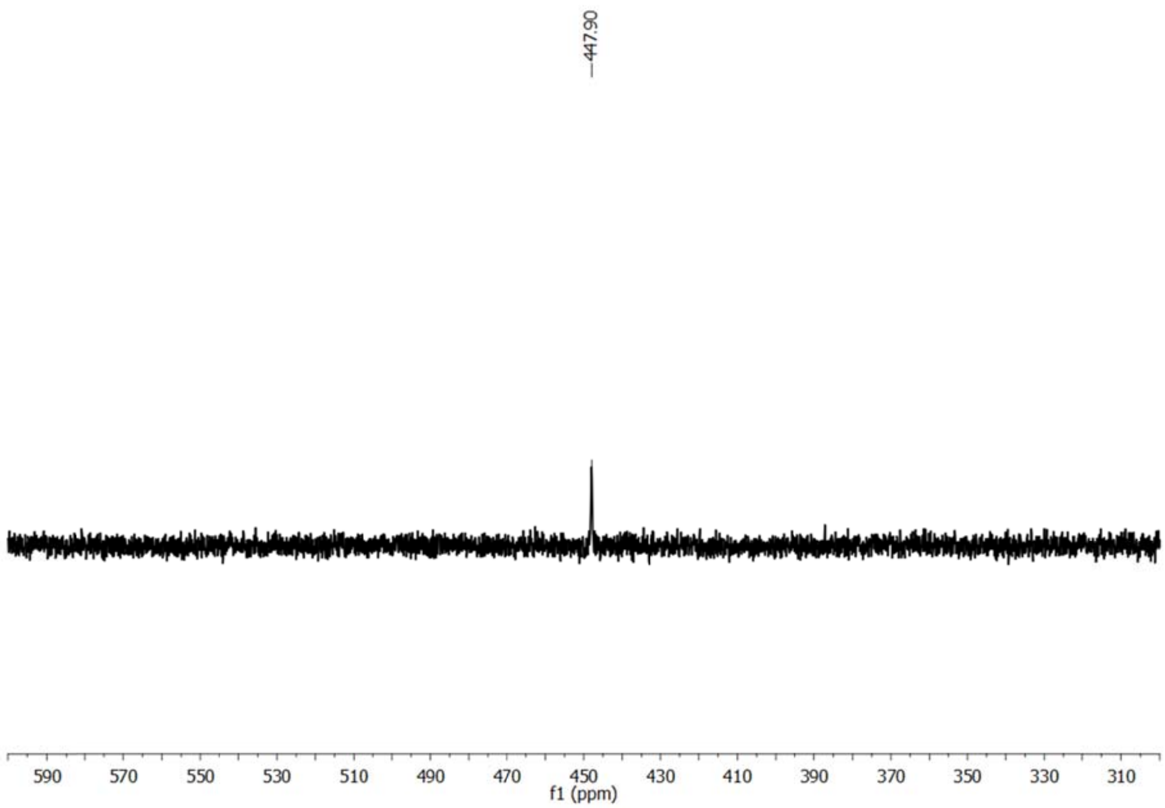

Figure S150. ^{77}Se-NMR of compound **9d**.

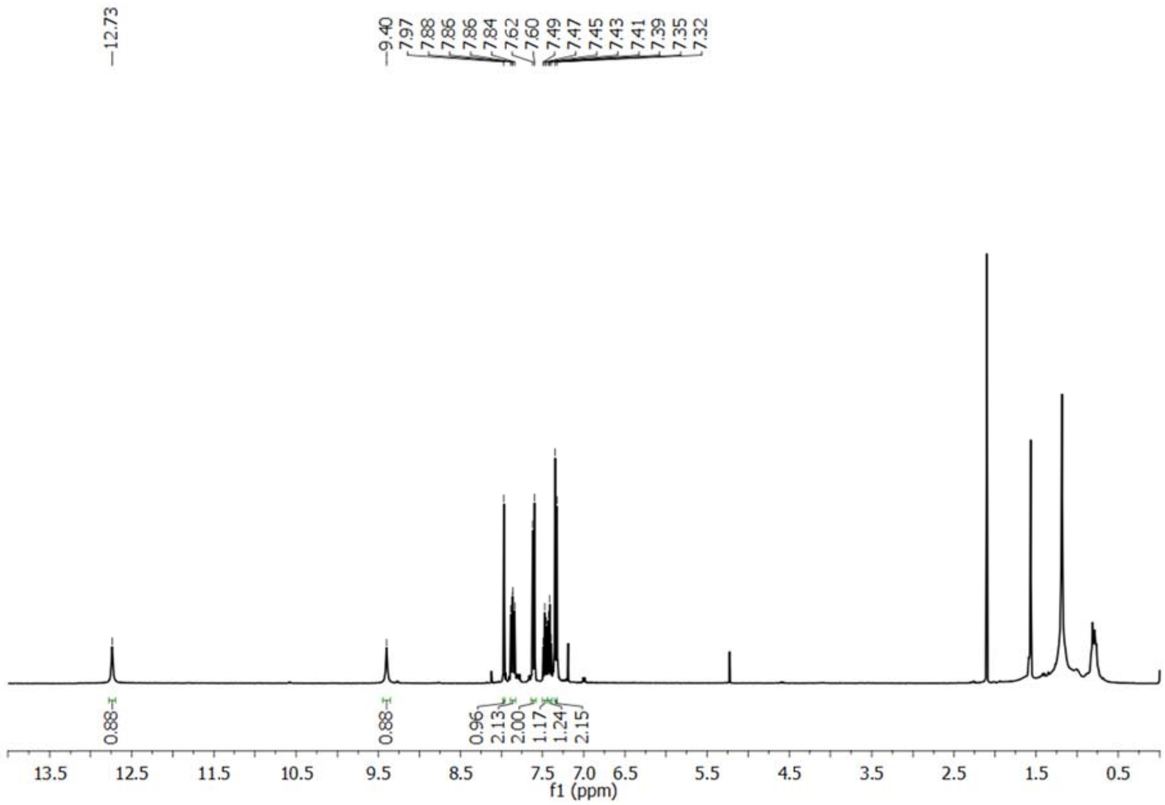

Figure S151. ^{1}H-NMR of compound **9e**.

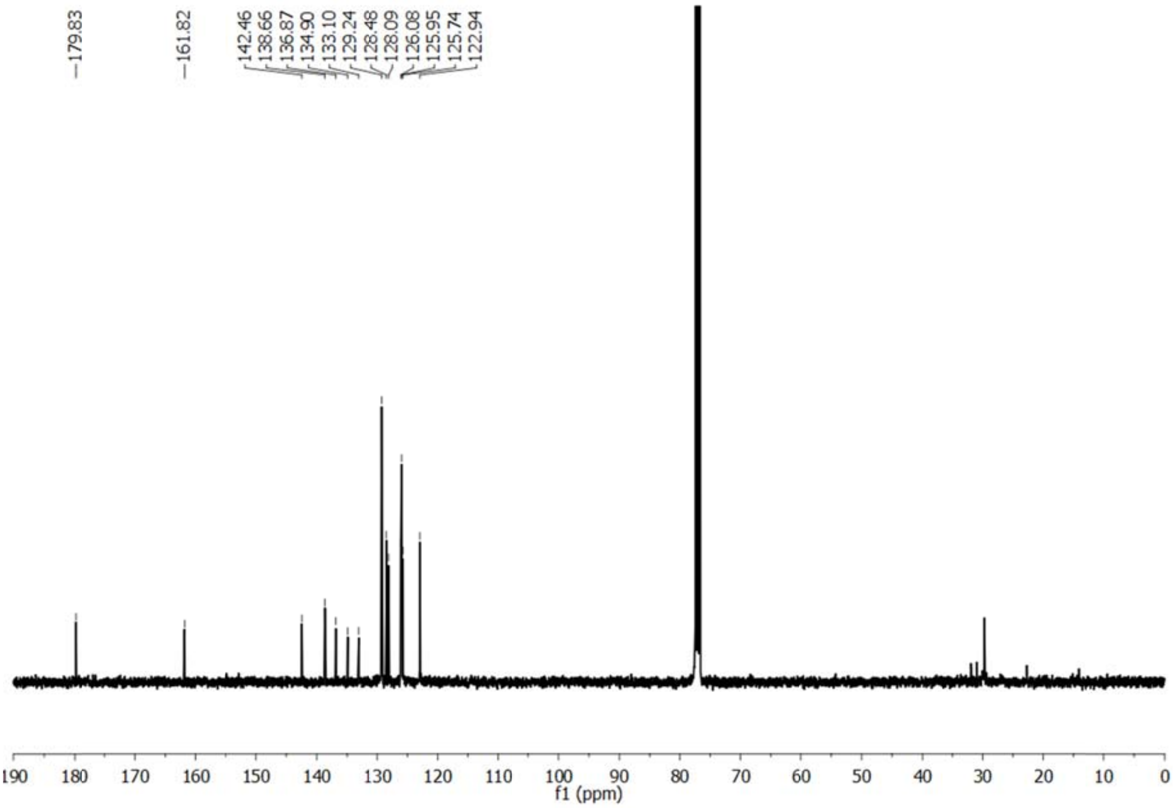

Figure S152. ^{13}C-NMR of compound **9e**.

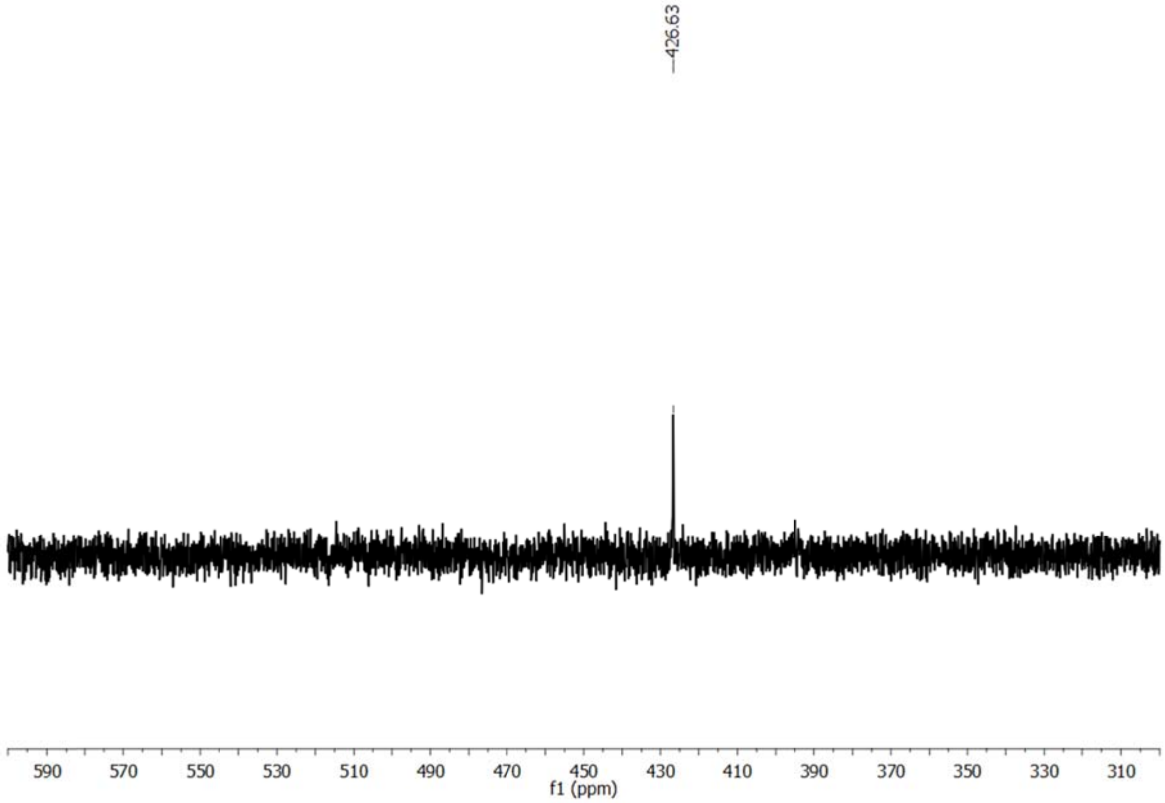

Figure S153. ^{77}Se-NMR of compound **9e**.

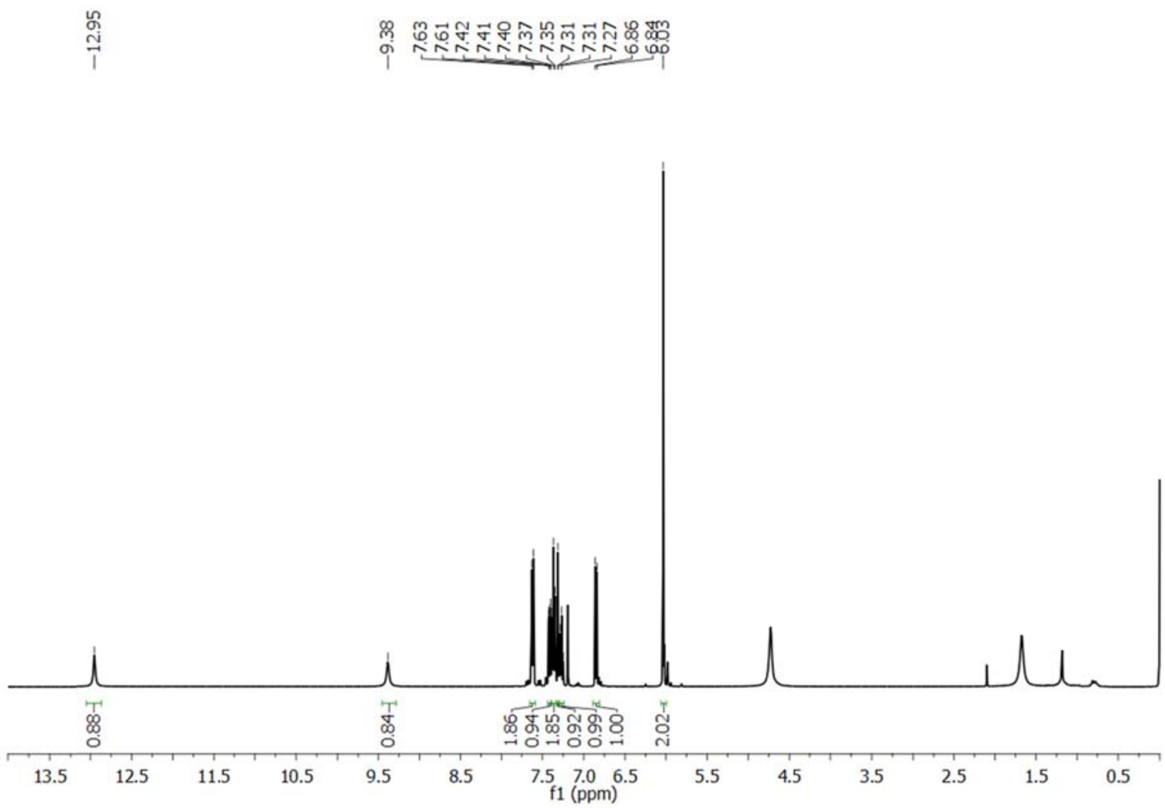

Figure S154. ¹H-NMR of compound **10a**.

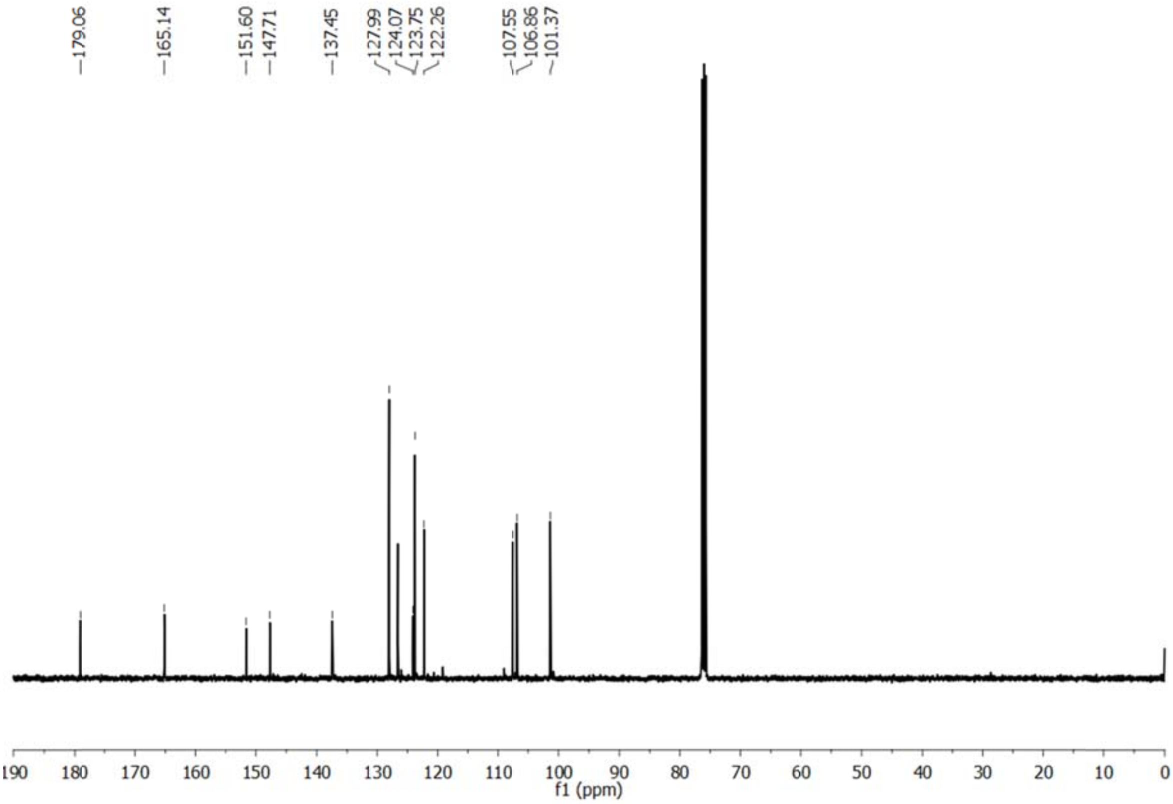

Figure S155. ¹³C-NMR of compound **10a**.

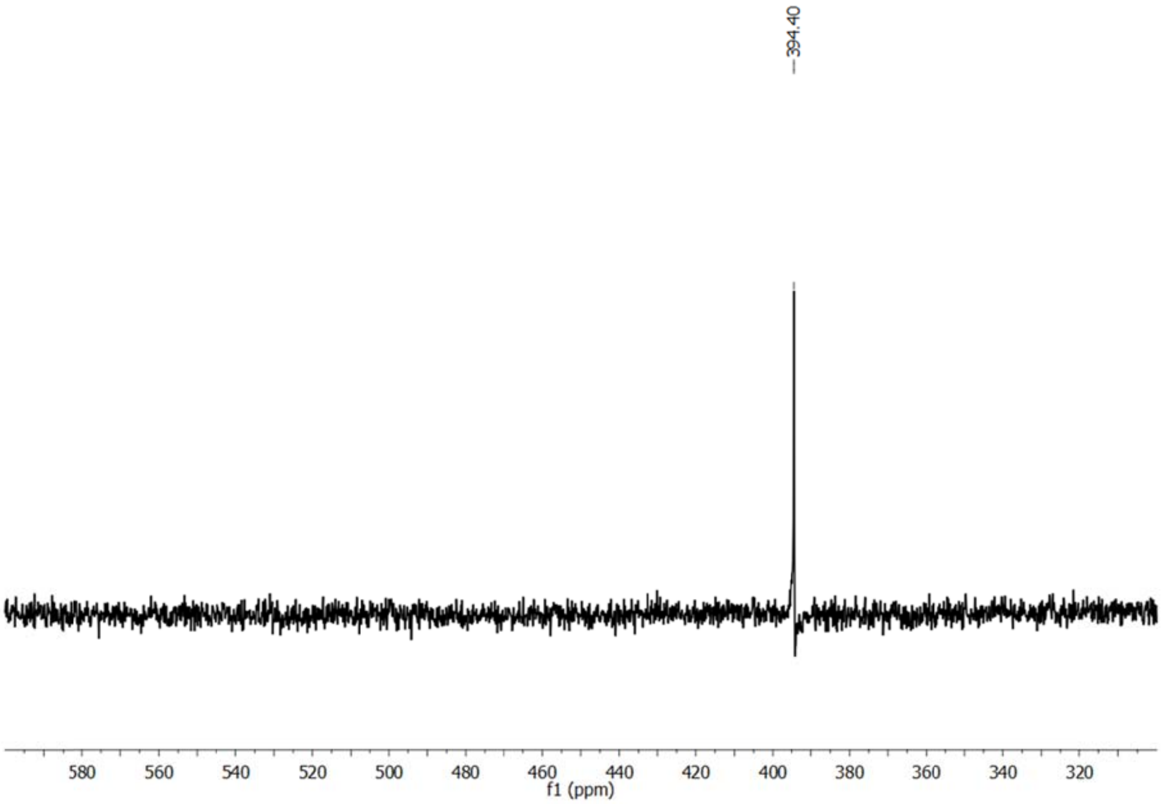

Figure S156. ^{77}Se-NMR of compound **10a**.

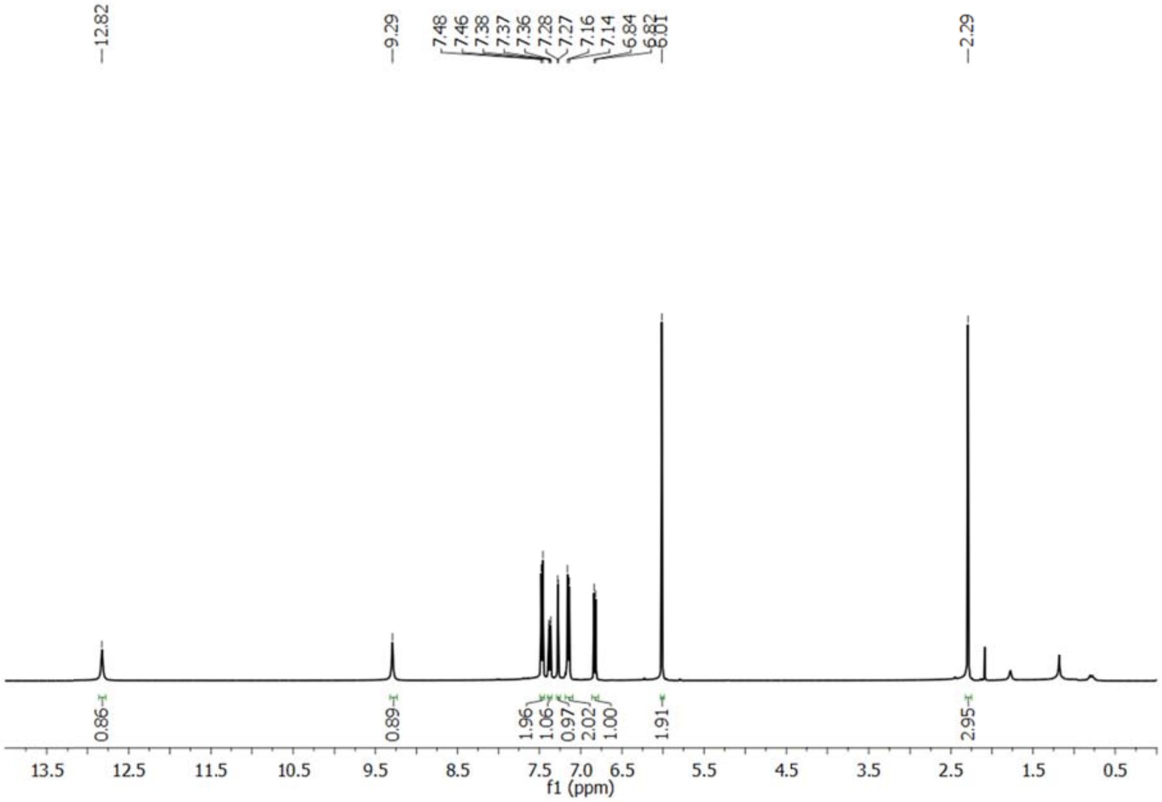

Figure S157. ^{1}H-NMR of compound **10b**.

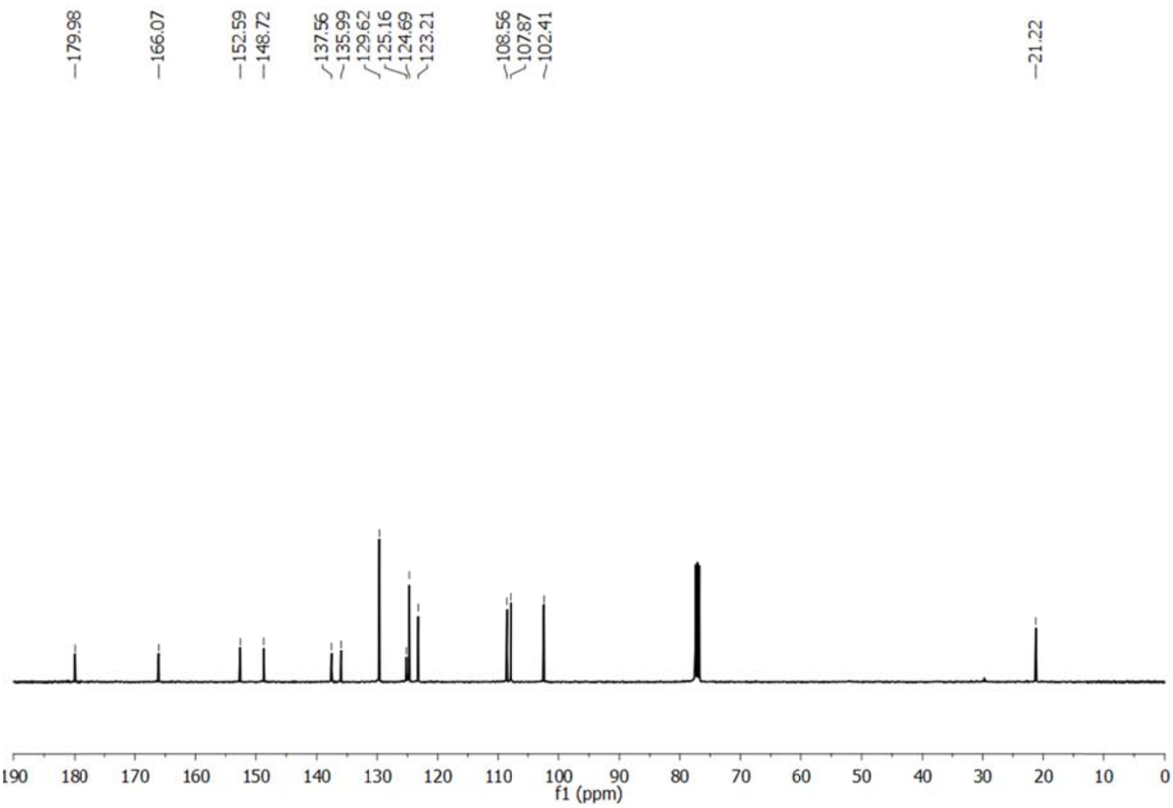

Figure S158. ^{13}C-NMR of compound **10b**.

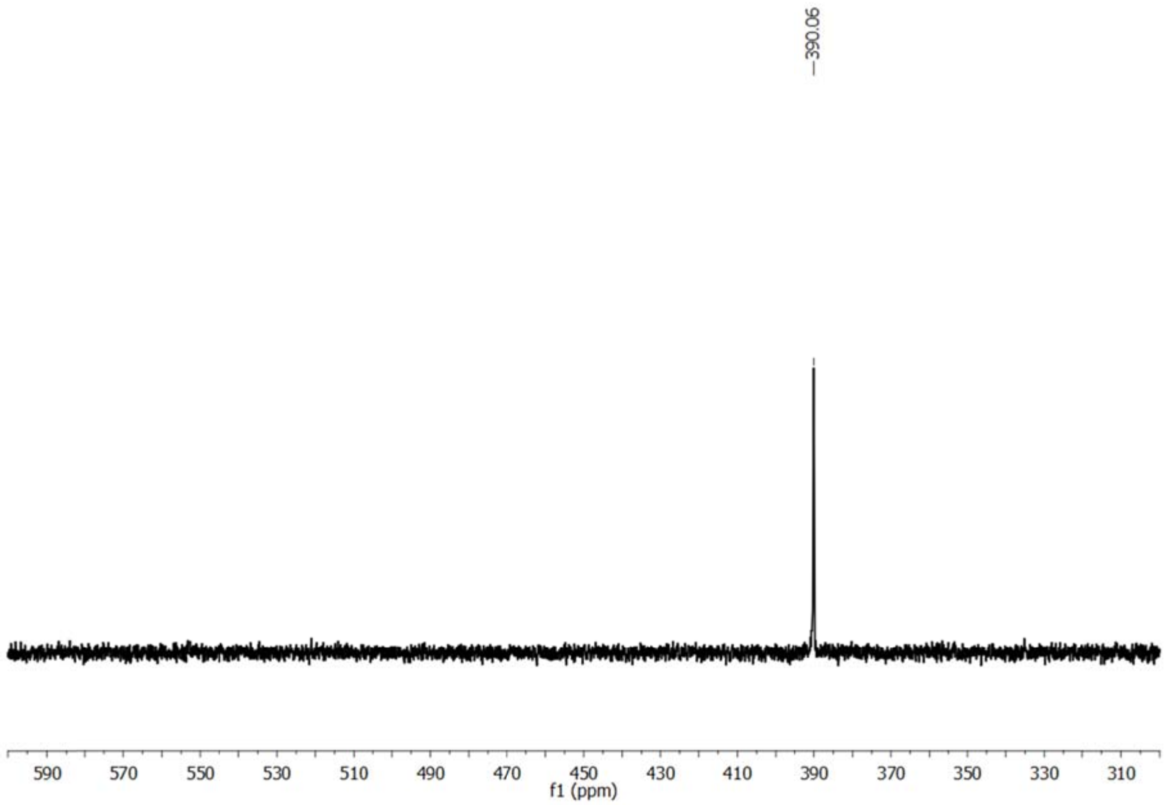

Figure S159. ^{77}Se-NMR of compound **10b**.

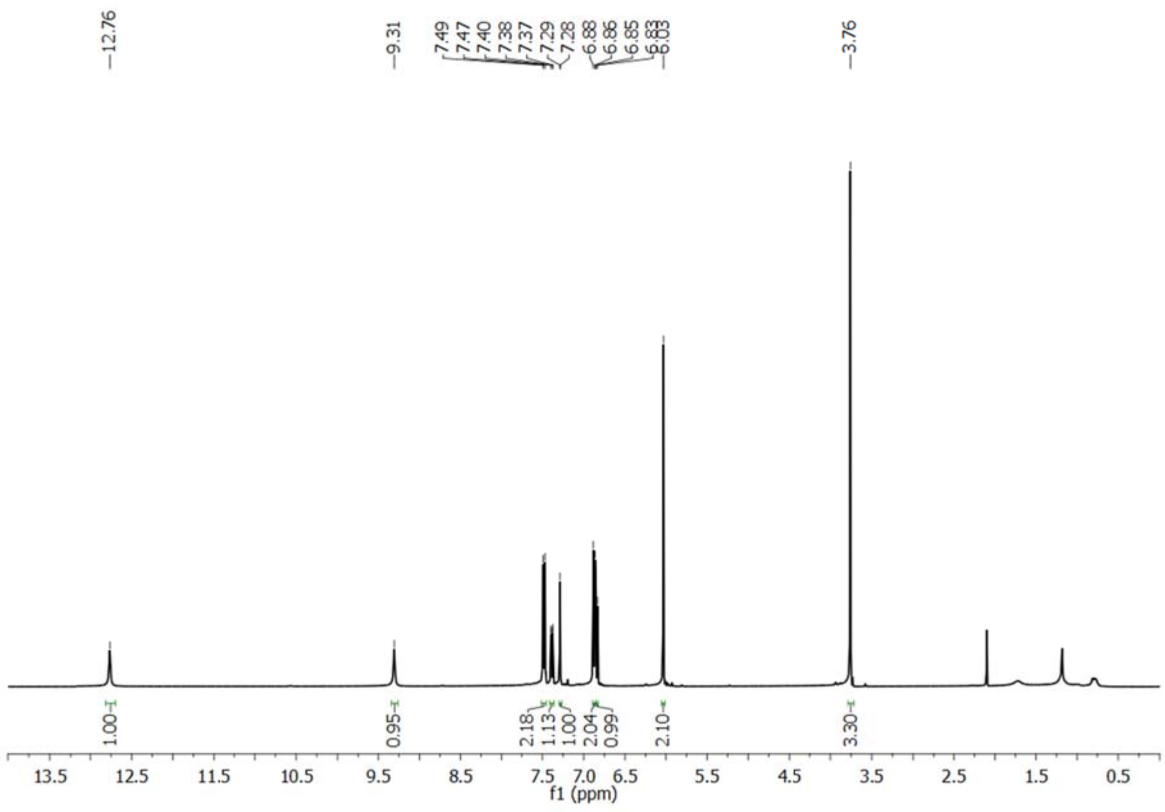

Figure S160. ^{1}H-NMR of compound **10c**.

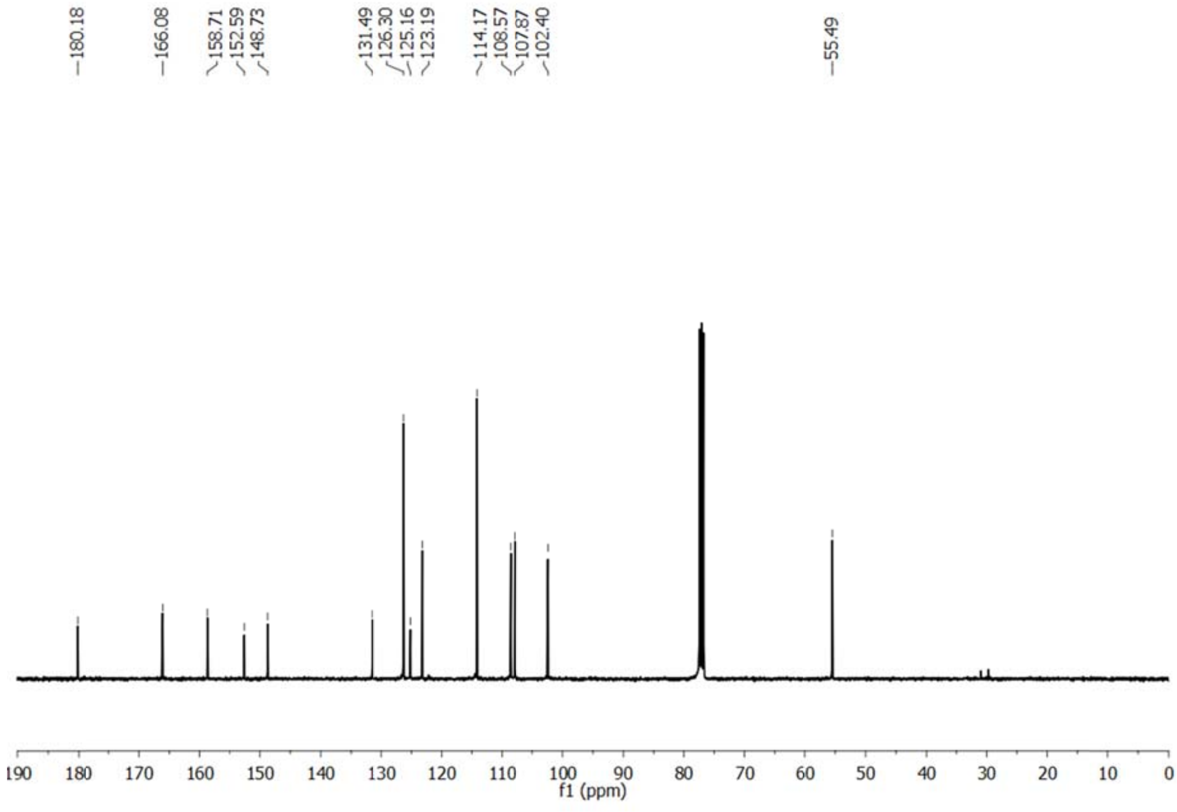

Figure S161. ^{13}C-NMR of compound **10c**.

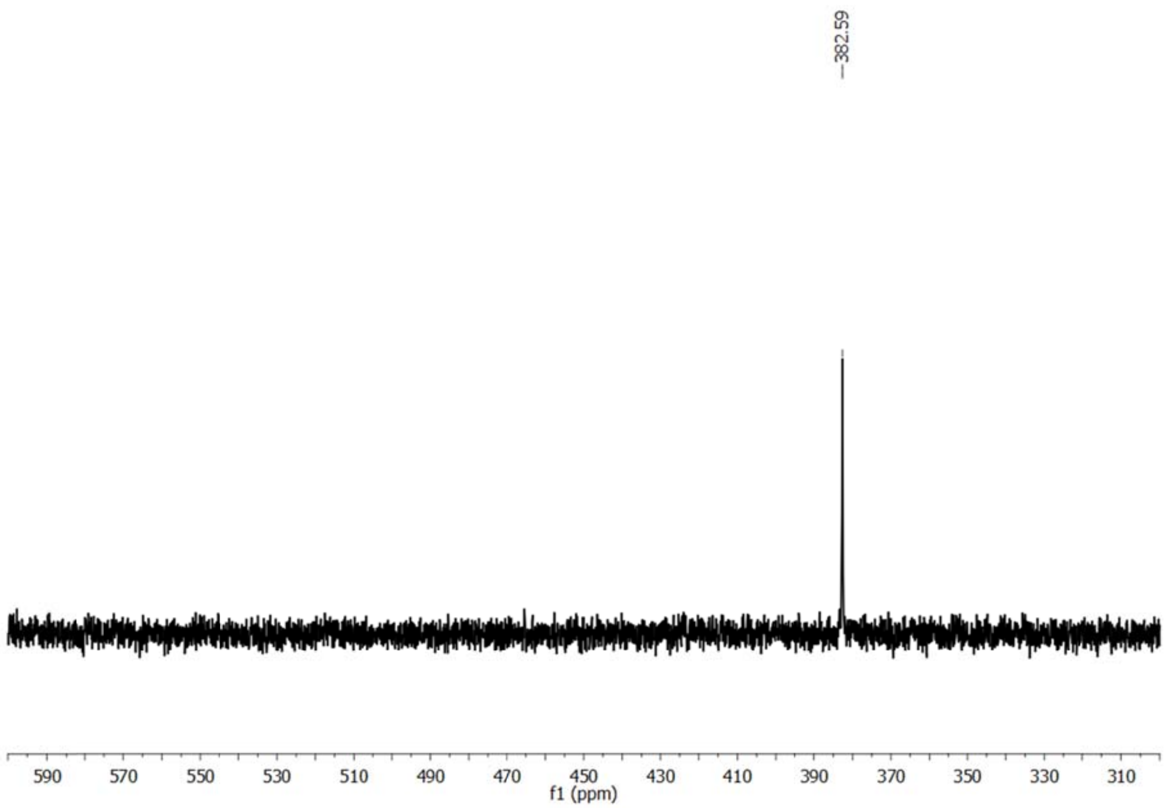

Figure S162. ^{77}Se-NMR of compound **10c**.

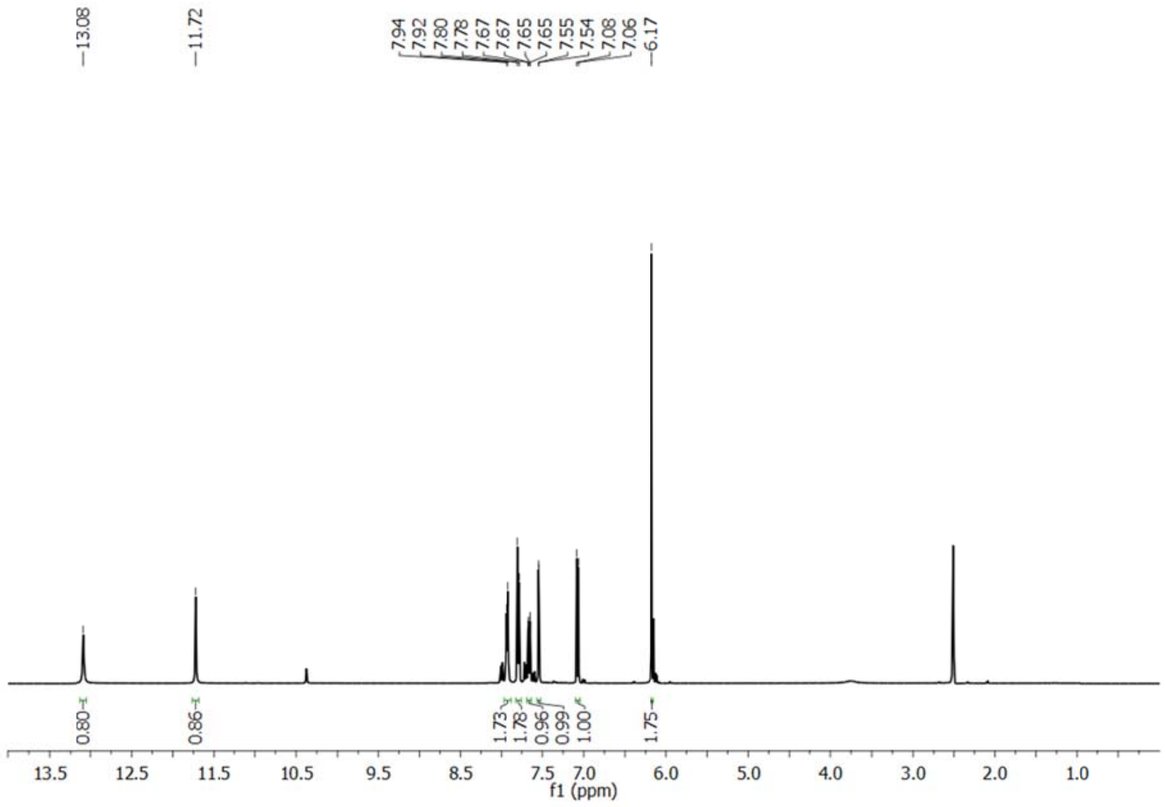

Figure S163. ^{1}H-NMR of compound **10d**.

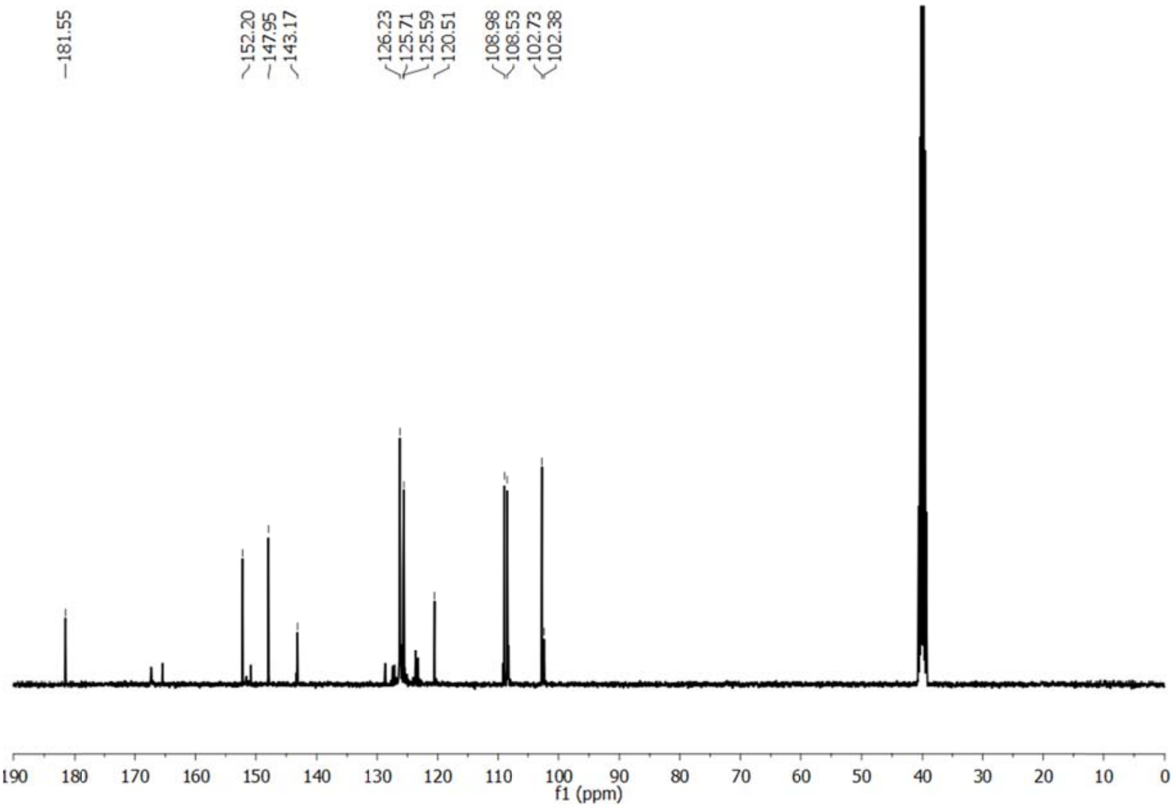

Figure S164. ^{13}C-NMR of compound **10d**.

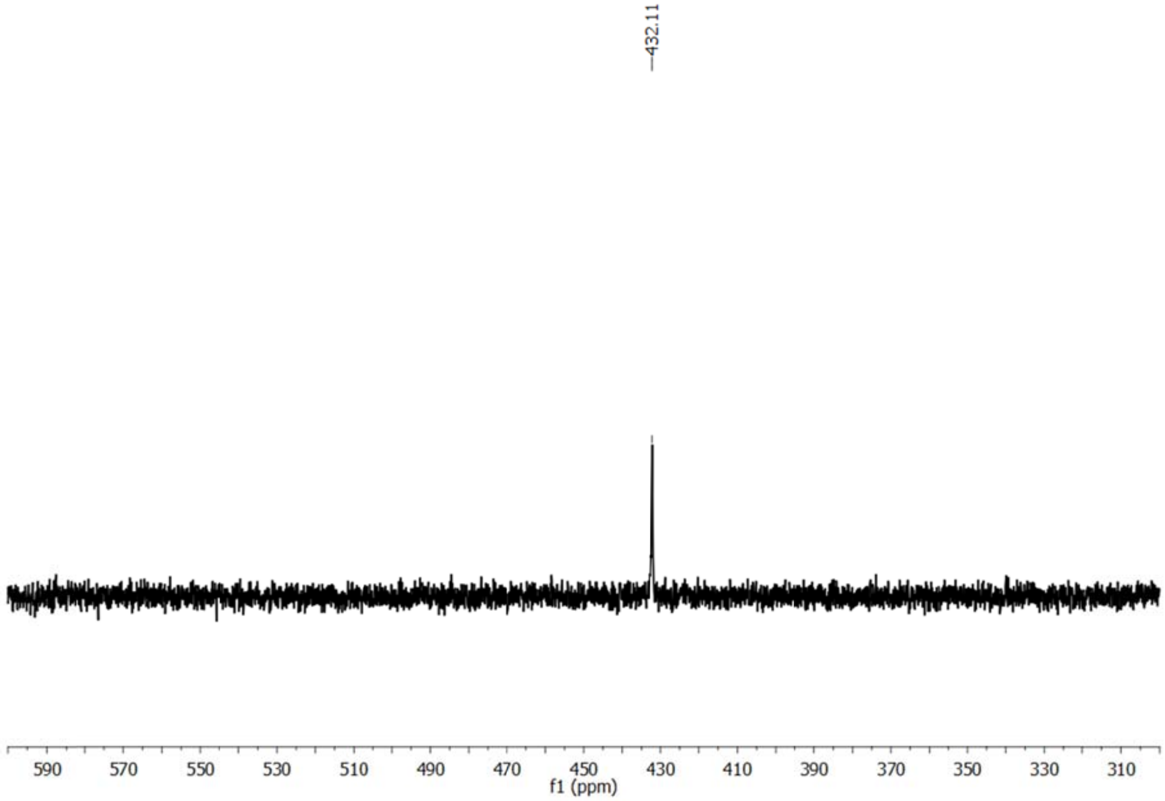

Figure S165. ^{77}Se-NMR of compound **10d**.

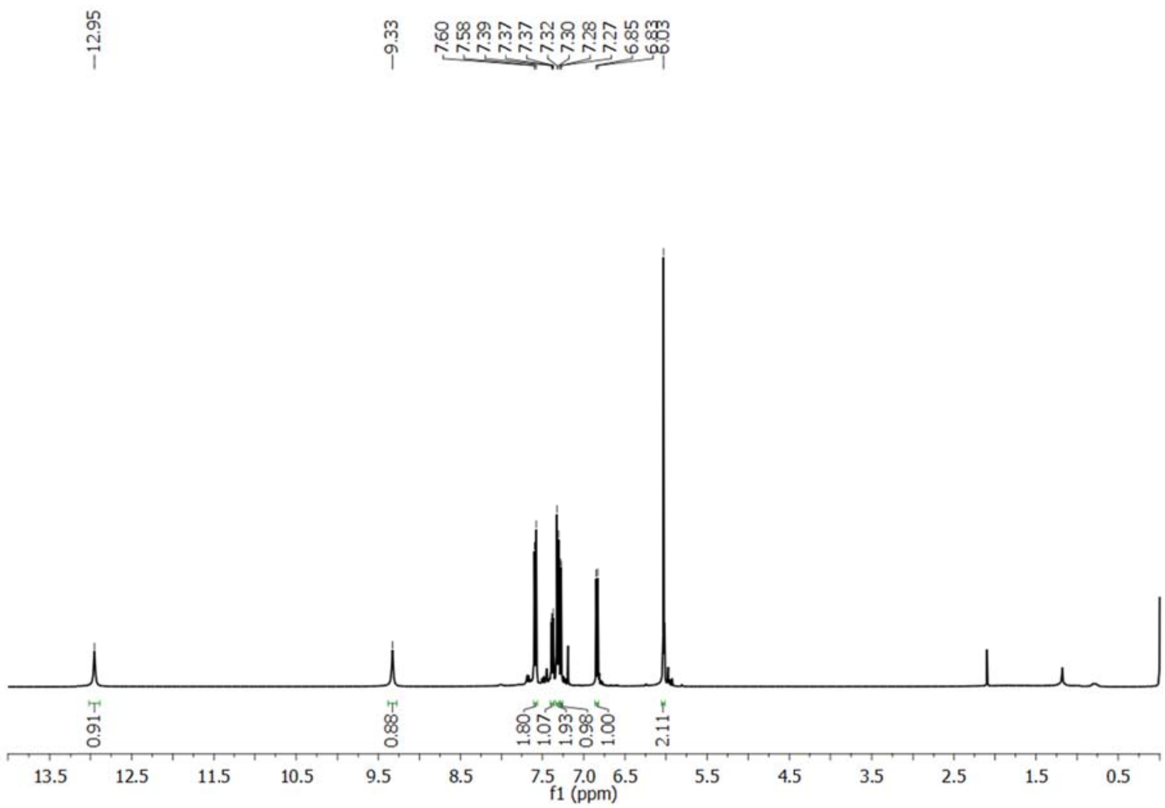

Figure S166. ^{1}H-NMR of compound **10e**.

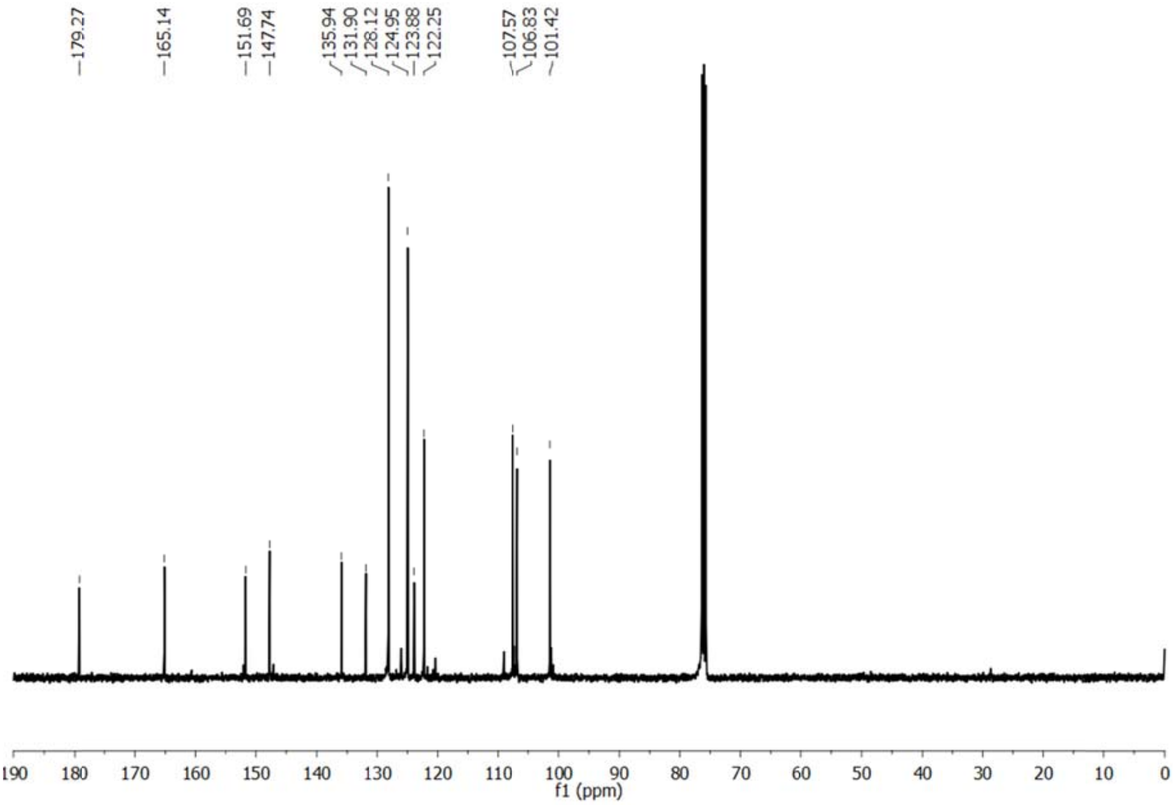

Figure S167. ^{13}C-NMR of compound **10e**.

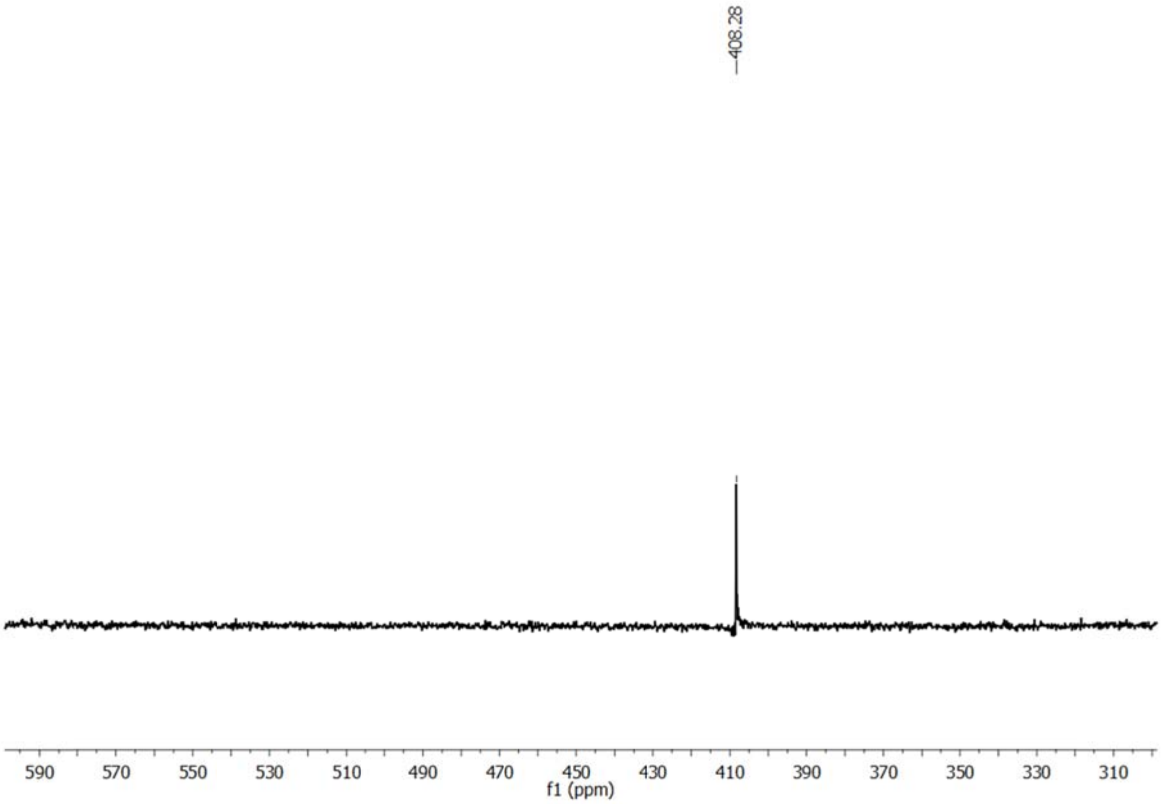

Figure S168. ^{77}Se-NMR of compound **10e**.
